# Sub-parts-per-billion CO_2_ Detection based on Dissipative Whispering Gallery Mode Microcavity Sensor

**DOI:** 10.1038/s41467-026-75218-y

**Published:** 2026-07-04

**Authors:** Shujing Ruan, Guangzhen Gao, Jianing Zhang, Haotian Wang, Dongxing Cheng, Jun Guo, Chuanyong Ren, Weidong Chen, Deyuan Shen, Tingdong Cai

**Affiliations:** 1 College of Physics and Electronic Engineering, Jiangsu Normal University, Xuzhou, 221116 China; 2 Laboratoire de Physicochimie de l’Atmosphère, Université du Littoral Côte d’Opale 189A, 59140 Dunkerque, France

**Keywords:** Optical sensors, Near-infrared spectroscopy

## Abstract

Whispering gallery mode microcavities provide strong light–matter interactions owing to their ultrahigh optical confinement, but the small gas refractive index change limits their ability to sense trace gases. Here we show that gas absorption can be detected using a dissipative sensing mechanism in a non-functionalized whispering gallery mode microcavity. Instead of tracking resonance frequency shifts used in conventional dispersive sensing, our method converts optical absorption into variations in resonance depth through thermally induced dissipation. Quantitative carbon dioxide detection was achieved over a concentration range of 1.5 to 400 parts per million with a correlation coefficients exceeding 0.99. The sensor reached a detection limit of 168 parts per trillion at an integration time of 400 seconds and an accuracy of approximately 0.4%. Continuous monitoring further demonstrated stable operation under ambient conditions. These results establish dissipative microcavity sensing as a promising approach for compact, low-cost, and highly sensitive trace gas detection.

## Abstract (web summary)

A whispering gallery mode microcavity detects CO2 through a dissipative sensing mechanism, achieving sub-part-per-billion sensitivity. The approach overcomes key limitation of conventional microcavity gas sensors while remaining compact and low cost.

## Introduction

Precise and real-time measurement of gas concentration is of great significance in various fields, including environmental monitoring^1^, industrial process control^2^, public safety^3^, and medical diagnostics^4^. With growing concerns over air pollution, greenhouse gas emissions, and toxic gas leaks, there is an increasing demand for gas sensing technologies that offer high sensitivity, high selectivity, miniaturization, and excellent stability^5^.

Currently, gas detection technologies can be broadly classified into three categories: electrochemical and semiconductor material-based sensing methods^6–8^, spectroscopic absorption-based methods^9,10^, and emerging techniques based on photoacoustic effects or optical microstructures^11–13^. Electrochemical^14^ and metal oxide semiconductor sensors^15^ are widely adopted in household safety monitoring and smart devices due to their simple structure, fast response, and low manufacturing cost; however, their poor stability, susceptibility to temperature and humidity variations, limited selectivity, and significant cross-sensitivity constrain their use in complex or high-precision scenarios. Spectroscopic absorption techniques such as Tunable Laser Absorption Spectroscopy (TLAS)^16^ and Non-Dispersive Infrared (NDIR)^17^ exploit the specific absorption lines of gas molecules at selected wavelengths, providing high sensitivity and strong selectivity for atmospheric monitoring and industrial emissions measurement. Absorption spectroscopy aims to enhance light-molecule interaction. To this end, mid-infrared light sources are preferred, since molecular absorption cross-sections in this band exceed those in the near-infrared by one to three orders of magnitude, thereby significantly boosting sensitivity. High sensitivity also depends on a long effective optical path, which is typically achieved with multi-pass cells or an integrated cavity. These configurations inevitably increase instrument size, structural complexity, and cost, all of which undermine portability. Moreover, advanced gas detection systems based on absorption spectroscopy require frequency locking loops to enhance detection performance, often relying on standard molecular absorption lines or high-finesse Fabry-Pérot (FP) cavities as frequency references.

In recent years, a number of emerging gas sensing techniques have been developed to overcome the limitations of traditional methods. These include photoacoustic^18,19^ and photothermal^20,21^ detection, fiber interferometry^22^, fiber grating^23^, and microcavity-based resonant sensing^24^. Most of them exploit interactions between gas molecules and light or sound, and offer key advantages such as compactness, low power consumption, and high sensitivity. Among these, optical microcavities based on whispering gallery modes (WGM) have shown great promise^25–30^. WGM cavities can be scaled down to the micrometer level while maintaining high Q-factors and excellent integrability. Thanks to their ultra-high intracavity field intensity, WGM sensors are exceptionally sensitive to slight changes in refractive index or absorption. Usually, WGM microcavities have a dispersive sensing mechanism that detects the wavelength shift of microcavity resonance caused by changes in external parameters^31,32^. By utilizing frequency locking technology, the detuning between the laser frequency and the microcavity resonance can be eliminated, thereby effectively improving the signal-to-noise ratio and enabling real-time detection^25,27,28^. However, when a WGM microcavity sensor is employed for gas detection, the gas-induced variation in the refractive index of the microcavity is intrinsically weak, thereby limiting the detection sensitivity of the system.

Functional coatings, such as polymers, graphene, or metal-organic frameworks (MOFs), enhance selectivity by selectively interacting with specific gases like ammonia, carbon dioxide, and volatile organic compounds (VOCs)^33–38^. It has been observed that sensing schemes based on WGM microcavities functionalized with specific coatings have achieved low limits of detection for certain gases. For instance, a WGM sensor coated with a ferrocene-containing polymer has achieved a limit of detection of 2.34 ppt for nitric oxide^33^, while a WGM sensor modified with a polyvinyl alcohol coating has reached 117 ppt for formaldehyde^34^. These studies outline multiple promising pathways for optical gas sensing toward higher sensitivity, improved selectivity, and improved integrability. Nevertheless, functional coatings in WGM-based gas sensing face several limitations. They are susceptible to environmental factors such as temperature and humidity, which can cause interference and reduce detection accuracy. Over time, coatings may degrade due to chemical interactions or environmental exposure, requiring frequent maintenance. While designed for specificity, cross-sensitivity to similar analytes in complex mixtures can compromise selectivity. Additionally, the fabrication of effective coatings involves complex processes and high costs. The finite absorption capacity of these coatings limits their performance in high-concentration scenarios. These drawbacks highlight the need for more robust materials and advanced techniques to improve reliability and long-term usability in gas sensing applications. More importantly, the surface functionalization of the WGM cavity will significantly degrade the Q factor, thereby reducing the sensor's sensitivity and detection limit. Moreover, the dispersive WGM sensor requires the laser to have good frequency stability. The frequency fluctuation of the laser itself can cause the WGM sensor to misjudge whether the resonance wavelength shift is caused by the changes that need to be detected. And after long-term operation, the frequency drift of the laser will move away from the WGM cavity resonance region, causing the sensor function to fail. In order to stabilize the laser frequency, a stable frequency reference and a complex electronic feedback control system are usually used, which will inevitably increase the complexity and cost of the laser system^39,40^.

Recently, WGM microcavities sensors using dissipative sensing mechanisms have attracted widespread attention. Dissipative WGM sensors monitor the changes in the transmitted light intensity at the microcavity resonance frequency due to the changes in the microcavity intrinsic or coupling loss caused by the external environment^41,42^. It does not need to measure the resonant wavelength shift as in the dispersive WGM sensor, thus avoiding the influence of laser frequency fluctuations on the sensor measurement results. This dissipative WGM microcavity sensor has been successfully applied to achieve high-sensitivity detection of sound waves, which relies on the mechanical vibration to cause changes in the coupling strength between the tapered fiber and the microcavity^43–45^. When the dissipative sensor is applied to gas detection, the absorption of light by the gas will cause the temperature near the microcavity to change. It will not only change the resonance wavelength of the microcavity, but also change the coupling strength of the optical mode from the cavity to the outside. Therefore, it is only necessary to measure the change in transmittance at the microcavity's resonant frequency to obtain gas concentration information, thereby overcoming the problem that the microcavity's resonant frequency is insensitive to changes in the gas state. In particular, the light intensity enhanced by the high Q factor WGM microcavity will change the coupling strength more significantly due to gas absorption, so a more sensitive dissipative sensor can be obtained.

In this article, we propose a non-functionalized dissipative WGM gas sensor and demonstrate its performance using CO_2_ gas concentration detection as an example. A silica microsphere WGM cavity is used as the sensing platform, and its resonance mode is excited by a tapered fiber. Notably, the field enhancement effect within the microcavity results in more intense heating in the coupling region. This ultimately causes a significant variation in the microcavity coupling strength, making the system highly sensitive to changes in CO_2_ gas concentration. By establishing the relationship between the transmitted light intensity at the resonance and the gas concentration, the accurate measurement of CO_2_ gas concentration can be achieved. The high Q-factor WGM microcavity not only amplifies the effect of heat generation due to gas absorption on the coupled system, but also the dissipative sensing mechanism eliminates the interference of laser frequency fluctuations on the measurement accuracy, and combines the modulation and demodulation technology to isolate the low-frequency intensity fluctuations of the laser. Our WGM microcavities have exceptionally high intracavity field enhancement; however, this compensates for the inherently weak gas absorption in this spectral region, enabling gas detection with significantly improved sensitivity. The high-sensitivity sensor demonstrated in this work can achieve performance comparable to those using mid-infrared lasers even when working in the near-infrared band (1572.3 nm), and finally the detection limit of CO_2_ gas concentration can reach the ppt level.

## Results

### System configuration

**Fig. 1:**
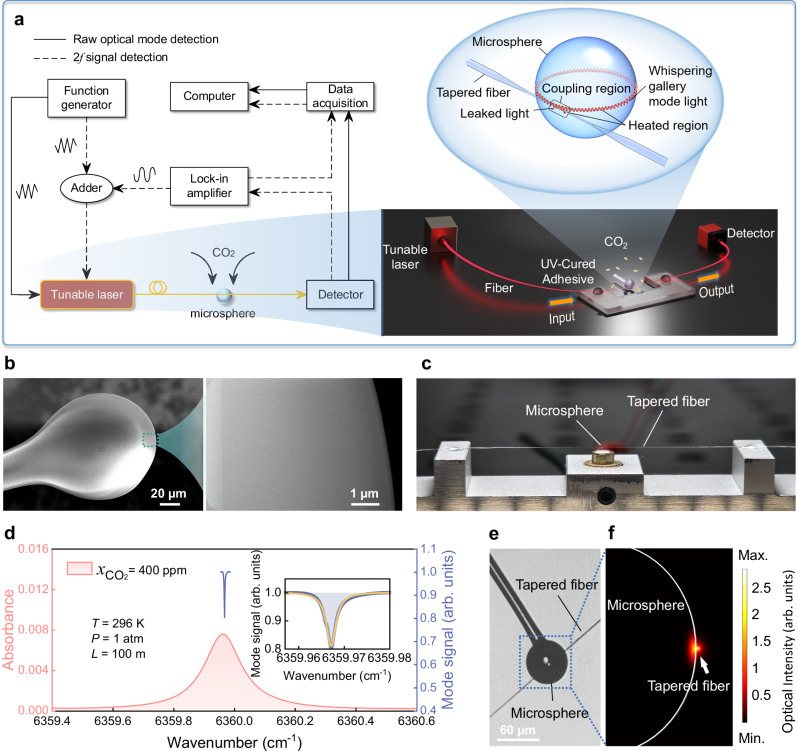
Principle and implementation of dissipative sensing in a whispering gallery mode microcavity. **a** Experimental setup of the dissipative whispering gallery mode gas sensor. The insets show the sensor principle, including the coupling of a tapered optical fiber with a microsphere whispering gallery mode gas cavity, as well as the optical mode distribution and the gas absorption-induced heating region. **b** Scanning electron microscope image of the microsphere and the zoom in of its surface morphology. **c** Installation configuration of tapered fiber and the microsphere coupling systems. **d** CO_2_ absorption line and one resonance position of the microsphere cavity. Inserted graph: Lorentz profile fitting of the resonance mode. **e** Microscopic photo of the coupling between 60 µm microsphere cavity and the tapered fiber. **f** Mode field distribution of microsphere cavity and tapered fiber at coupling cross section.

The WGM microcavity was fabricated by melting a tapered region of a single-mode silica optical fiber using localized electric discharge. First, we taper the fiber to about 10 μm in diameter, and then cut it off at the waist to form a tip. Through several electrode discharges, the fiber tip is melted to form a smooth microsphere with a diameter of about 60 μm, as illustrated in Fig. 1e. Scanning electron microscope (SEM) images of the microsphere are shown in Fig. 1b. The extremely smooth surface of the microcavity ensures a very high Q-factor.

The experimental setup for CO_2_ sensing based on the dissipative WGM sensor is shown in Fig. 1a. After optimizing the coupling position between the microsphere and the tapered fiber, they were mounted together on an aluminum bracket using UV-curable adhesive, which is shown in Fig. 1c. A microscopic image of the coupled system is shown in Fig. 1e. The tapered fiber is attached to the microsphere surface to ensure coupling stability, and this coupling method can effectively enhance the sensitivity of the proposed dissipative sensor, which has been analyzed above. To allow controlled gas exchange, the fiber-microsphere coupling system was enclosed in a sealed sample chamber. A distributed feedback (DFB) tunable diode laser (NEL, NLK1L5GAAA) with a linewidth of ~2 MHz was employed, operating near 1572.3 nm. This wavelength corresponds to the CO_2_ absorption line at 6359.967 cm^−1^, with a line strength of 1.761 × 10^−23^cm^−1^/mol·cm^2^, as shown in Fig. 1d. No significant interference from water vapor absorption is observed near this wavelength. The selected WGM resonance aligns closely with the absorption line center and has a much narrower linewidth, minimizing the impact of frequency jitter on signal strength. Lorentzian fitting of the resonance yields a linewidth of 0.00257 cm^−1^, corresponding to a *Q* factor of 2.47 × 10^6^. The optical mode in the microsphere microcavity is confined near the equatorial region by total internal reflection, where the field intensity is significantly higher than that in the tapered fiber, as illustrated by the red wavy line in Fig. 1a. The evanescent field component of the optical mode, as well as the light fields that are scattered from the tapered fiber in the coupling region, will be absorbed by the surrounding gas. The absorbed energy gives rise to localized heating, and the generated heat subsequently diffuses along the fiber, the microsphere, and the surrounding air, as indicated by the red shading in the inset of Fig. 1a. Because the molecular absorption linewidth is much broader than the cavity mode linewidth, and the microsphere supports multiple resonant modes within a given spectral range, the coupling conditions can be optimized experimentally to selectively excite the most suitable mode for sensing applications.

### Optimization of modulation frequency and modulation amplitude

**Fig. 2:**
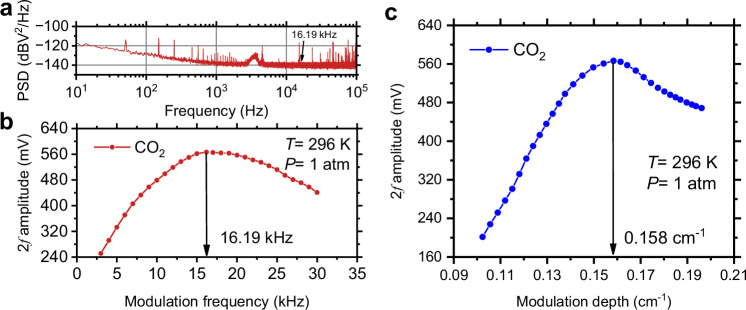
Parameter optimization for Modulation-Demodulation technology. **a** Noise power spectral density (PSD) of the laser and detection system in the 10 Hz-100 kHz frequency range. **b** Variation of 2 *f* signal amplitude with modulation frequency. **c** Variation of 2 *f* signal amplitude with modulation depth.

The noise floor of the developed sensor mainly comes from the relative intensity noise (RIN) of the laser and the noise generated by the spontaneous temperature fluctuation of the WGM microcavity. The thermal noise of the microcavity is negligible compared to the RIN of the laser, which refers to a detailed analysis in Supplementary Note 1.6. Figure 2a shows the measured noise floor in the 10Hz-100kHz frequency range, which includes the RIN of the laser and the background noise of the detection and data acquisition system. It can be found that before 5 kHz, the laser and detection system have excess noise. In order to eliminate the influence of this part of noise on the detection limit of the sensor, we will use modulation and demodulation technology to move the detection signal to 16 kHz to eliminate the low-frequency noise of the system.

During the signal modulation and demodulation process, the parameters of the sinusoidal waveform used for modulation, such as its frequency and depth, are critical to the overall signal quality. To determine the optimal values of these parameters, we conducted CO_2_ measurements in ambient air using the described system. The 2 *f* signals corresponding to the selected optical mode were analyzed as a function of modulation frequency and modulation depth. Figure 2b, c shows the dependence of the 2 *f* signal amplitude on modulation frequency and depth, respectively. Based on these results, a modulation frequency of 16.19 kHz and a modulation depth of 0.158 cm⁻¹ were selected for use in this study. As shown in the noise characterization data in Fig. 2a, the choice of 16.19 kHz effectively suppresses low-frequency noise from the laser and detection system, further validating its suitability.

**Fig. 3:**
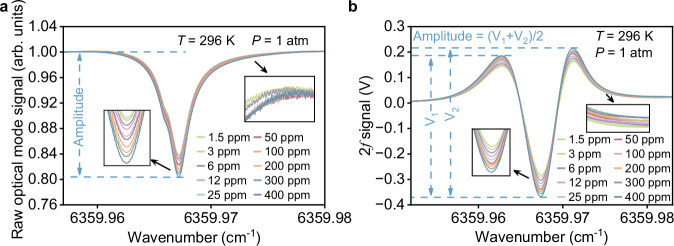
Sensor response to varying CO_2_ concentrations. **a** The raw optical mode signals measured at different CO_2_ concentrations. **b** The 2 *f* signals measured at different CO_2_ concentrations.

To assess the system calibration and measurement accuracy of the dissipative WGM gas sensor for CO_2_ detection, we tested ten CO_2_/N_2_ mixtures with concentrations ranging from 1.5 to 400 ppm. The mixtures were dynamically generated by diluting a certified 1% CO_2_ gas standard (in N_2_) using a commercial gas mixing system. The improvement in detection performance provided by the modulation-demodulation scheme was evaluated by recording both the direct optical resonance signal (without modulation) and the demodulated second-harmonic (2 *f*) signal at each concentration level. The optical resonance traces shown in Fig. 3 are raw data, without any smoothing or filtering in post-processing. For clarity, each main spectrum is accompanied by a magnified inset that highlights the detailed spectral structure and intrinsic noise characteristics. All measurements were performed at 1 atm and 296 K to ensure consistent experimental conditions. For each concentration, 20 independent signal sets were acquired within 7 minutes. Each set was the average of 200 repeated scans, improving the signal-to-noise ratio (SNR) and minimizing random errors to ensure reproducibility.

### CO_2_ concentrations measurement

**Fig. 4:**
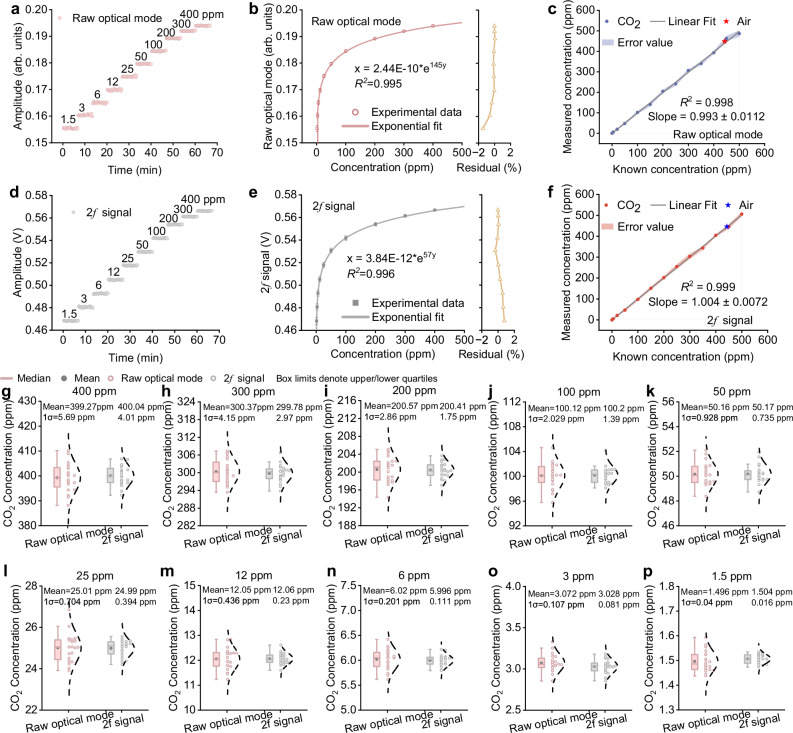
Dynamic response and calibration of CO_2_ sensing. **a** The measured optical mode amplitude versus calibration time for different CO_2_ concentration levels ranging from 1.5 to 400 ppm. **b** Experimental data points and the corresponding fitted curve of optical mode amplitude as a function of CO_2_ concentration, showing residuals within 2%. **c** Comparison between the CO_2_ concentration measured by the raw optical mode and the nominal concentration recorded during gas preparation, the shaded area is the error bar. **d** The measured 2 *f* amplitude versus calibration time for different CO_2_ concentration levels ranging from 1.5 to 400 ppm. **e** Experimental data points and corresponding fitted curve of 2 *f* amplitude as a function of CO_2_ concentration, showing residuals within 1%. **f** Comparison between the CO_2_ concentration measured by 2 *f* signal and the nominal concentration recorded during gas preparation, the shaded area is the error bar. **g**–**p** Analysis of data discrepancy at various concentrations measured in Fig. 4a, d. Data in **b** and **e** are means from twenty independent data; error bars correspond to s.d.

Figure 3a, b present the raw signals for the optical mode of the microcavity and corresponding 2 *f* signals at different CO_2_ concentrations. It can also be seen from the figure that both signals exhibit high SNRs across the entire tested range (1.5-400 ppm), with peak amplitudes increasing monotonically with CO_2_ concentration. According to Eq. 1 and the numerical simulation results in Fig. S1 in Supplementary Note 1.1, when the WGM microcavity is at under-coupled, the increase in coupling strength will lead to a decrease in the transmittance at the resonance. As the CO_2_ gas concentration increases, the light absorption increases, which causes the tapered fiber to heat up and expand, thereby increasing its coupling strength to the WGM microcavity. The amplitude of the raw optical mode and 2 *f* signals measured over a 70-minute period at different CO_2_ concentrations is shown in Fig. 4a, d, respectively. To provide a detailed assessment of measurement precision and repeatability across the entire dynamic range, statistical analysis was performed on the measured concentration data. As shown in Fig. 4g–p, box-and-scatter plots are presented for each of the ten tested concentration levels (from 1.5 ppm to 400 ppm). Each plot displays the distribution of 20 consecutive measurements obtained under stable conditions. The box indicates the interquartile range (IQR), median, and mean, while the overlaid individual points illustrate the actual scatter of the data. Both detection methods exhibit consistently high relative repeatability across all concentrations, with a relative standard deviation (RSD) of approximately ±3% in raw optical mode signal and an RSD of approximately ±1% in 2 *f* signal. This confirms the sensor's stable relative performance throughout its operational range.

To analyze the concentration-dependent behavior, the average signal amplitudes at each concentration level were plotted in Fig. 4b, e, where the vertical axis represents the amplitudes of the raw optical mode and 2 *f* signal, and the horizontal axis corresponds to the target concentrations recorded during gas mixing. The fitting residuals, shown in the figures, are within 2% for the raw optical mode and 1% for the 2 *f* signal. According to the measured data, the signals follow an approximately logarithmic relationship with CO_2_ concentration. To conveniently retrieve gas concentrations from experimentally measured signals, we applied an inverse exponential fit to them, yielding correlation coefficients of *R*^2^ = 0.995 and *R*^2^ = 0.996 for the optical mode and 2 *f* signal, respectively: *X*_*CO2*_ = 2.44E-10*e^145*PMode*^, *X*_*CO2*_ = 3.84E-12*e^57*PMode–2f*^, where *P*_*Mode*_ and *P*_*Mode-2f*_ represent the average of 100 peak values at each concentration level. The monotonic and reproducible fitting processing supports quantitative concentration retrieval over the investigated concentration range; nevertheless, the fitting should be regarded as a semi-empirical approximation rather than a strict theoretical law (detailed discussion can be found in Supplementary Note 1.3). Additional information on the dynamic range and local linearity Analyses can be found in Supplementary Note 3.

To independently validate the sensor's accuracy and avoid potential bias from using the same dataset for both calibration and validation, a separate verification experiment was conducted. A series of CO_2_ concentrations (0.001, 0.1, 5, 20, 50, 100, 150, 200, 250, 300, 350, 400, 450, and 500 ppm), distinct from those used for the initial calibration, was prepared using a precision gas mixer. Each concentration was measured independently using both the raw optical mode and 2 *f* signal, and the reported values represent the average of six consecutive measurements. Sensor performance was evaluated by comparing the measured concentrations with the corresponding reference values, as shown in Fig. 4c, f. Excellent linearity was obtained over the entire tested range. For the raw optical mode, a linear fit yields a slope of 0.993 ± 0.0112 with *R*^*2*^ = 0.998, corresponding to a measurement accuracy of approximately 0.7%. The 2 *f* signal exhibits better performance, with a fitted slope of 1.004 ± 0.0072, *R*^*2*^ = 0.999, and an accuracy of approximately 0.4%. The absolute accuracy of the concentration measurements and the practical applicability of the system were further assessed through an independent comparison experiment under ambient CO_2_ conditions. Measurements were carried out simultaneously using the raw optical mode, the demodulated 2 *f* signal, and a commercial calibrated CO_2_ analyzer (GMM-1000, specified accuracy ±1% of reading), which served as the reference instrument. The analyzer reported a concentration of 443 ppm, while the raw optical mode and 2 *f* method yielded 450 ppm and 446 ppm, respectively. The asterisks in Fig. 4c, f represent the comparison of the results measured by the two methods with the analyzer. When measuring in outdoor atmospheric environments, the absolute deviations from the reference value were approximately 6-8 ppm for the raw optical mode and 3-4 ppm for the 2 *f* signal, corresponding to relative accuracies of about ±2% and ±1% of the reading, respectively. The main sources of uncertainty include gas mixing errors, peak extraction inaccuracies, laser frequency fluctuations, and limited precision in pressure and temperature control. Overall, these results confirm the superior accuracy of the 2*f*-WMS approach, which benefits from enhanced noise suppression and reduced sensitivity to baseline drift.

### Measurement Precision and Detection Sensitivity

**Fig. 5:**
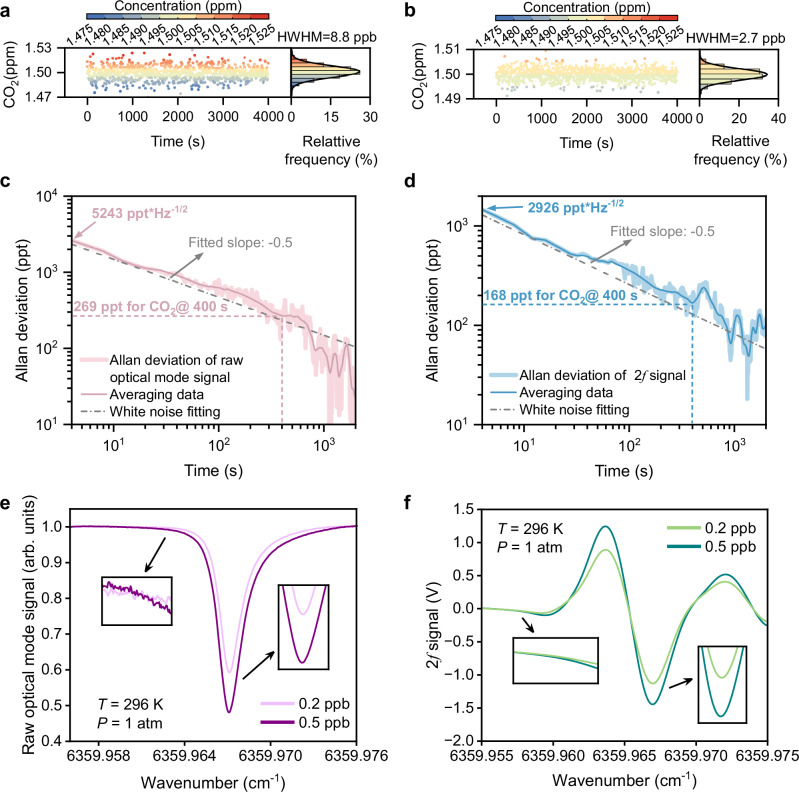
Sensor stability and sub-ppb detection capability. **a, b** Distribution of CO_2_ concentration measured continuously for 4000 s for 1.5 ppm CO_2_-N_2_ sample using raw optical mode and 2 *f* signal (*T* = 296 K, *P* = 1 atm), as well as the frequency distribution and Gaussian profile fitting of measured concentration. **c**, **d** Allan deviation plot of the dissipative whispering gallery mode gas sensor based on the data shown in Fig. 5a, b. **e**, **f** Raw optical mode signals and 2 *f* signals for CO_2_-N_2_ mixtures at 0.2 ppb and 0.5 ppb concentrations with 400 s integration time.

To evaluate the stability of the WGM microcavity-based sensor, continuous measurements a 1.5 ppm CO_2_/N_2_ mixture were performed over a period of approximately 66 min with a temporal resolution of 4 s, as shown in Fig. 5a, b. The corresponding raw optical mode and 2 *f* signals are partially presented in Fig. S9 of the supplementary material. The measurement precision was analyzed separately for the raw optical mode and the 2 *f* signal. For the raw optical mode (Fig. 5a), the histogram of the retrieved concentrations follows a normal distribution centered around the mean value. Gaussian fitting yields an *R*² value of 0.983. The precision, defined as the standard deviation (1*σ*) of the distribution, is 7.51 ppb, corresponding to 8.8 ppb in terms of the half width at half maximum (HWHM). For the 2 *f* signal (Fig. 5b), the histogram also exhibits a normal distribution, with a Gaussian fit *R*² of 0.987. The corresponding precision is significantly improved to 2.28 ppb (1*σ*) and 2.7 ppb (HWHM). Compared with the raw optical mode, the modulation-demodulation technique effectively suppresses low-frequency noise, resulting in a ~ 3.3-fold improvement in measurement precision compared to the raw optical mode.

The minimum detection limit (MDL, i.e. LOD) was further quantified through Allan deviation analysis. To ensure a reliable estimation, the time-series data were preprocessed with a 50-point moving-average window prior to calculating the Allan deviation. For the raw optical mode (Fig. 5c), the Allan deviation exhibits a *τ*^−1/2^ dependence, indicating white-noise-limited performance, with a bandwidth-normalized sensitivity of 5243 ppt Hz^−1/2^. This behavior persists up to an averaging time of 400 s, at which a MDL of approximately 269 ppt is achieved. For the 2 *f* signal (Fig. 5d), a similar *τ*^−1/2^ trend is observed, with an improved bandwidth-normalized sensitivity of 2926 ppt Hz^−1/2^. At an averaging time of 400 s, the MDL reaches approximately 168 ppt, confirming the superior noise performance of the 2*f*-based measurement. To verify the reliability of the Allan-derived detection limits, additional measurements were performed on CO_2_/N_2_ mixtures with concentrations of 0.2 ppb and 0.5 ppb, prepared through multiple dilution steps, using an integration time of 400 s. The corresponding raw optical mode and 2 *f* responses are shown in Fig. 5e, f, respectively. The magnified insets reveal clear signal separation between the two concentration levels, with well-resolved features above the intrinsic noise floor. To clearly resolve the weak signal variations at low concentrations, the detector gain and lock-in amplifier sensitivity were increased for the measurements in Fig. 5e, f. Therefore, the absolute signal amplitude and noise level are not directly comparable with those in Fig. 3. This adjustment does not affect the sensing mechanism, but is used solely to optimize signal visualization across different concentration ranges. These results demonstrate that the sensor maintains high sensitivity and stable performance even near its detection limit.

### Real-time monitoring of ambient CO_2_

**Fig. 6:**
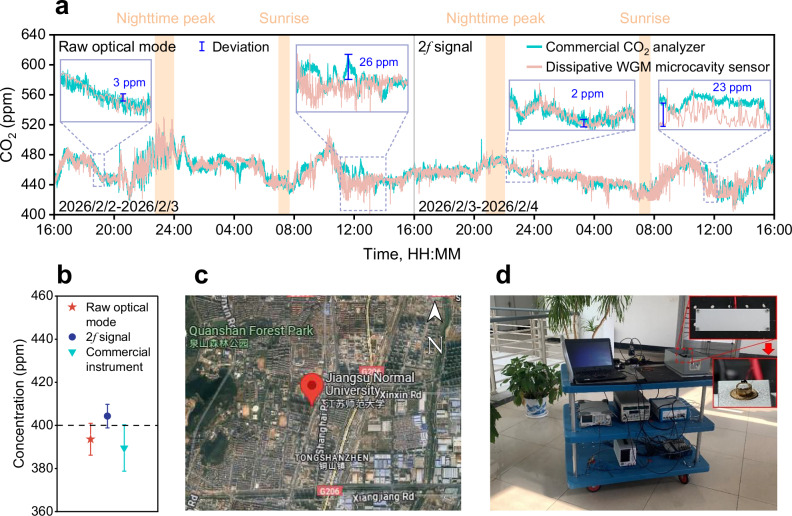
Continuous monitoring of CO_2_ in the actual atmosphere. **a** Comparative time series of ambient CO_2_ concentration over ~24 h, measured simultaneously by the dissipative whispering gallery mode-based sensor (raw optical mode and 2 *f* signal) and a calibrated commercial reference analyzer. **b** Comparison of three measurement methods at a known CO_2_ concentration of 400 ppm. **c** Detailed locations of dissipative whispering gallery mode-based CO_2_ sensor measurement site and its surroundings in Xuzhou. Map provided by Google Earth. **d** The whole CO_2_ gas sensing system on a mobile cart. The inset shows the packaged coupling system of the microsphere microcavity and the tapered optical fiber.

To evaluate the practical applicability of the system, long-term ambient CO_2_ monitoring was conducted on the main campus of Jiangsu Normal University (JSNU, 34.19°N, 117.18°E, 10 m above ground level (a.g.l.)), as shown in Fig. 6c. The dissipative WGM-based sensor was deployed alongside the calibrated commercial CO_2_ analyzer (GMM-1000) to perform simultaneous and parallel measurements. Both instruments sampled ambient air at the same location. The dissipative WGM-based CO_2_ sensor was implemented on a compact mobile cart, as shown in Fig. 6d. In this stage of the study, commercial instruments-including the laser controller, lock-in amplifier, function generator, and photodetector-were employed to provide a stable platform for validating the sensing mechanism and benchmarking system performance under well-controlled laboratory conditions. The use of discrete instruments was intended for performance evaluation rather than to represent the system's prototype architecture. The core functionalities can be consolidated through custom-designed electronics and packaged optical components, enabling substantial miniaturization and improved portability. A prototype implementation of the integrated system is described in Supplementary Note 8. For real-time ambient monitoring, encapsulated devices comprising microspheres and tapered fibers are placed directly in the natural environment. This microcavity enclosure has not undergone airtight encapsulation, allowing ambient CO_2_ to diffuse into the enclosure through the fiber feedthrough openings.

Figure 6a displays 24 h of CO_2_ measurements obtained from the raw optical mode, the 2 *f* signals and the calibrated commercial analyzer. Most discrepancies between the proposed sensor and the commercial analyzer are within 0-5 ppm for the majority of the measurement period. Larger deviations occur only during limited time intervals, with the maximum deviation reaching approximately 26 ppm for concentrations retrieved from the raw optical mode and approximately 23 ppm for those obtained from the 2 *f* signal. A comparative measurement was carried out in the laboratory for a 400 ppm CO_2_-N_2_ standard gas sample prepared using a gas mixing system, with simultaneous detection by our sensor and a commercial instrument. The results are presented in Fig. 6b. The deviations between the concentrations determined by the raw optical mode and the 2 *f* signal of our device and the standard value are approximately 6.4 ppm and 4.3 ppm, respectively, whereas the deviation of the concentration measured by the commercial instrument from the standard value is approximately 10.5 ppm. All three datasets exhibit a pronounced diurnal variation characteristic of a campus environment. From 00:00 to 06:00, CO_2_ concentrations increase gradually due to continuous plant respiration, minimal human activity, and relatively weak atmospheric mixing. A more rapid rise is observed between 06:00 and 08:00, corresponding to the onset of morning activities. Between 08:00 and 15:00, enhanced solar radiation strengthens photosynthetic uptake, leading to a progressive decline in CO_2_ concentration and a daytime minimum around noon. After 15:00, as solar intensity decreases, photosynthetic activity weakens, and CO_2_ levels begin to rise again. From 18:00 to 24:00, the combined effects of ceased photosynthesis, ongoing plant respiration, human presence, and traffic emissions produce a distinct nighttime peak. Two independent monitoring campaigns were conducted. In both cases, the raw optical mode and 2 *f* signals from the dissipative WGM-based sensor were benchmarked against the calibrated commercial analyzer. The measured time series exhibit highly consistent temporal trends and concentration levels across all instruments, demonstrating the reliability, repeatability, and field applicability of the proposed dissipative WGM-based CO_2_ sensing system.

Our device-packaged sensor incorporates a dual-stage temperature control system. This system effectively mitigates the impact of temperature fluctuations in the ambient environment on the sensor’s readouts (see Supplementary Notes 4 and 6). Within the thermally stabilized microcavity house, during sensor operation, the heat generated as the laser frequency scans across the WGM resonance is mainly localized to the region surrounding the microcavity and the tapered fiber. Consequently, the WGM coupling strength primarily responds to this localized temperature changing, demonstrating low susceptibility to temperature variations occurring in the broader surroundings. Additionally, some desiccants are placed inside the microcavity housing to filter out water vapor from the environment, thereby ensuring the long-term stability and working life of the sensor. Figure S11 in the supplementary materials presents the raw mode signals measured for the same 400 ppm CO_2_-N_2_ sample by varying the temperature of the sealed chamber containing the WGM microcavity. As shown, the signals remain highly consistent across different temperatures, with deviations within 1%. Further details on the system’s environmental robustness and long-term stability are provided in Supplementary Notes 9 and 10.

### Comparison with other methods

**Fig. 7:**
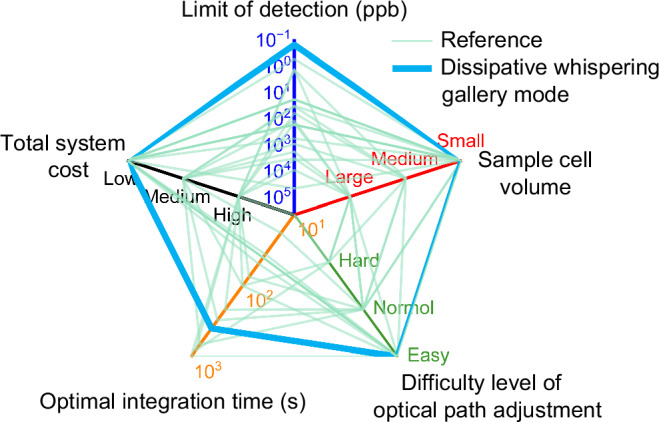
Comparison of Sensor Performance. Performance comparison of the dissipative whispering gallery mode gas sensor with state-of-the-art CO_2_ sensors from the literatures (Table 1 with Supporting References).

The measurement performance of the sensor is systematically evaluated by comparison with several representative optical systems of similar functionality whose excitation sources span the near-infrared to mid-infrared regions. The comparison focuses on key parameters including sample cell volume, system cost, difficulty level of optical path adjustment, optimal integration time, and limit of detection, as summarized in Fig. 7 and Table 1. The results indicate that the developed sensor achieves a limit of detection as low as 168 ppt, representing an order of magnitude improvement over current mainstream mature systems. In terms of sample cell volume, optical alignment complexity, and overall system cost, the developed sensor exhibits significant advantages. Specifically, the sample cell volume is only 20% of that of the smallest conventional design, and the cell's construction cost is merely USD 0.1. Moreover, the system enables highly sensitive measurements under ambient pressure, thereby eliminating the need for low-pressure operation typically required by conventional spectroscopic techniques. This feature simplifies system sealing and reduces the associated engineering complexity and potential measurement inaccuracies. For a detailed comparison, refer to Table 1.

**Table 1:** Comparison of gas sensing performance of state-of-the-art CO_2_ sensors

Spectral technology	LOD^a^	Sample cell volume	Difficulty level of optical path adjustment	TSO^b^	Optimal integration time	Wavelength
Multi-pass Cell^46^	130 ppb	Large	Normal	Medium	18 s	2.004 μm
OA-CEAS^47^	1.4 ppb	Large	Hard	High	100 s	1.6 μm
CRDS^48^	160 ppb	Large	Hard	High	816 s	1.6 μm
On-chip Slot Waveguide^49^	627 ppb	Small	Easy	Low	36 s	4.23 μm
On-chip Suspended Waveguide^50^	20 ppb	Small	Easy	Low	40 s	4.345 μm
SEIRA^51^	1 ppm	Medium	Hard	Medium	N/A	6-8 μm
PAS^52^	37 ppb	Medium	Normal	Medium	307 s	4.3 μm
QEPAS^53^	35 ppb	Small	Normal	Medium	670 s	2.0 μm
MEMS^54^	580 ppt	Small	Easy	Low	49 s	4.323 μm
CEPAS^55^	4 ppm	Small	Normal	High	75 s	1.57 μm
Photothermal Spectroscopy^56^	1.5 ppb	Medium	Easy	High	1000 s	2.003 μm
LITES^57^	3.59 ppm	Large	Easy	Medium	100 s	2.00401 μm
Dispersion Spectroscopy^58^	65.78 ppb	Medium	Normal	High	218 s	4.329 μm
NDIR^59^	2 ppm	Large	Normal	Medium	N/A	4.3 μm
FTIR^60^	10 ppm	Large	Hard	High	5 s	4.25 μm
Fiber FP Interferometer^61^	480 ppb	Small	Easy	Medium	N/A	4.266 μm
Fiber Grating^62^	401 ppm	Medium	Easy	Medium	20 s	0.7 μm
Silicon Microring Resonator^63^	370 ppm	Small	Easy	High	N/A	1.56 μm
Antiresonant Fiber^64^	144 ppm	Small	Normal	Medium	1.5 s	1.574 μm
Photonic Crystals^65^	2000 ppm	Small	Easy	Medium	N/A	0.463-0.668 μm
Functionalized Dispersive WGM^66^	50 ppm	Small	Easy	Low	N/A	1.525-1.57 μm
Dispersive WGM^67^	184 ppb	Small	Easy	Low	N/A	1.55 μm
Dissipative WGM (This work)	168 ppt	Small	Easy	Low	400 s	1.572 μm

^a^LOD represents the limit of detection.

^b^TSO represents total system cost.

Compared with typical dispersive-based WGM microcavity CO_2_ sensor systems, the present system demonstrates a roughly three orders of magnitude improvement in detection limit. Furthermore, it utilizes the modal peak height as the retrieval parameter for concentration, effectively mitigating the influence of mode drift induced by environmental temperature or pressure fluctuations, which is a common issue in dispersive systems. This approach significantly enhances long-term monitoring stability.

The developed sensor not only maintains high measurement performance but also features a compact structure, low cost, and strong anti-interference capability. Additionally, it benefits from the communication-band light sources and detectors, which offer high commercial maturity, stable performance, low cost, and ease of integration. These advantages contribute to the system’s strong potential for engineering implementation and broad applicability in field measurements.

## Discussion

In this work, we developed a gas sensor based on a non-functionalized dissipative WGM microcavity and demonstrated its performance using CO_2_ detection in the 1.5 μm band as a representative example. A silica microsphere served as the sensing cavity. By selecting a resonant mode spectrally aligned with a CO_2_ absorption transition, system performance was evaluated using both the raw optical mode and the 2 *f* signal obtained through modulation-demodulation techniques. Allan deviation analysis revealed that, with a 400 s integration time, the MDLs are 269 ppt and 168 ppt for the raw optical mode and 2 *f* signal, respectively. The 2 *f* approach delivers superior sensitivity and noise performance, primarily because it suppresses low-frequency noise components associated with laser intensity drift and environmental perturbations. Comparison with representative state-of-the-art optical gas sensors confirms the excellent sensitivity and compactness of the proposed sensor. The high intrinsic field intensity sustained within the WGM microcavity, together with the dissipative sensing mechanism, enables strong light–matter interaction without the need for functional coatings. As a result, the sensor combines high sensitivity with structural simplicity, low cost, and good environmental robustness. Moreover, the system operates at ambient pressure, eliminating the need for a vacuum and facilitating practical deployment. Furthermore, although the system demonstrates good agreement with a commercial instrument in ambient-air CO_2_ monitoring, thereby validating its reliable sensing capability under atmospheric-pressure conditions, the proposed dissipative WGM sensor exhibits superior sensitivity in the low-concentration regime (from sub-ppb levels to approximately 10-20 ppm). Within this range, the microcavity resonance response maintains a high concentration-dependent slope, enabling enhanced concentration resolution and improved sensitivity to trace-level variations. Consequently, the sensor is particularly well suited for trace CO_2_ detection applications, including semiconductor manufacturing and electronic specialty gas quality control, high-purity industrial gas monitoring, low-background carbon-cycle and trace carbon-release studies, isotope-resolved CO_2_ sensing, and trace gas analysis in low-pressure or rarefied environments, where its high sensitivity and resolution offer significant practical value.

Owing to the high Q factor of silica microspheres in the 1-2 μm spectral range and the use of a non-functionalized configuration, the proposed dissipative WGM sensing scheme is inherently versatile. It can be extended to a variety of trace gases exhibiting near-infrared absorption features, such as CO_2_, CH_4_, NH_3_, H_2_S, C_2_H_2_, and HF, by selecting appropriate laser wavelengths; for example, measurements of CH_4_ and C_2_H_2_ are presented in Supplementary Notes 5 and 7. Furthermore, implementation of frequency-locking techniques to stabilize the laser to the cavity resonance would further enhance long-term stability and enable continuous real-time monitoring. Combined with the maturity and availability of telecom-grade light sources and detectors, these attributes position the dissipative WGM-based sensor as a promising platform for high-sensitivity, compact, and cost-effective trace gas detection in both laboratory and field applications.

## Methods

### Mechanism for the dissipative whispering gallery mode microcavity gas sensor

When a tapered fiber is used to couple laser into a WGM microcavity, the intensity transmittance of the tapered fiber at resonance can be described as:

1 $${\varGamma }_{0}={\left(\frac{{\kappa }_{ext}-{\kappa }_{int}}{{\kappa }_{ext}+{\kappa }_{int}}\right)}^{2}$$

where *κ*_*int*_ and *κ*_*ext*_ represent the loss rates due to the intrinsic loss in the WGM microcavity and the loss rate introduced by coupling (coupling strength), respectively (details in Supplementary Note 1.1). Due to weak gas absorption, the change in *Γ*_0_ is mainly due to the contribution of *κ*_*ext*_ rather than *κ*_*int*_. This is because the intrinsic loss rate *κ*_*int*_ caused by gas absorption is negligible.

The gas sensing mechanism of the proposed dissipative WGM microcavity is attributed to the photothermal effect: the absorption of the optical field by the gas induces a temperature rise, leading to thermal expansion and refractive index modulation of the tapered fiber and microsphere, which ultimately results in the variation in coupling strength *κ*_*ext*_ in Eq. (1). According to the Beer-Lambert law, the heat generated by gas absorption per unit time and per unit volume is (details in Supplementary Note 1.4):

2 $$Q=B\varGamma \frac{{P}_{{{{\rm{in}}}}}}{{A}_{eff}}Ng(\nu -{\nu }_{0})S$$

*B*, *Γ*, and *A*_*eff*_ are the enhancement factor of the intracavity light intensity, its evanescent field ratio, and the effective mode area, respectively. *P*_*in*_ is the input power from the tapered fiber. Since the optical intensity inside the high-Q WGM microcavity is much greater than the incident light, the microcavity as well as the tapered fiber in the coupling region (attached to the microcavity surface in the following experiment) can more sensitively sense the heating effect. *N*, *S* and *g*(*ν*-*ν*_0_) are the gas molecular number density, the absorption line strength, and the gas absorption line shape function, respectively. At the gas absorption peak (*v* = *ν*_0_), *g* reaches its maximum value *g*_0_. The heat generated is rapidly transferred to the microsphere, the tapered fiber, and the ambient air through thermal conduction, leading to a localized temperature increase. In the weak absorption regime, the temperature rise scales linearly with the gas concentration.

According to Eq. (1), we can relate *δκ*_*ext*_ to temperature change *δT* through thermal expansion and the thermo-optic coefficient:

3 $$\frac{\delta {\varGamma }_{0}}{{\varGamma }_{0}}=\frac{4{\kappa }_{int}{\kappa }_{ext}}{|{\kappa }_{ext}^{2}-{\kappa }_{int}^{2}|}\left[\frac{\partial {\kappa }_{ext}}{\partial n}n{\alpha }_{n}\delta T+\frac{\partial {\kappa }_{ext}}{\partial l}(\delta {l}_{1}+\delta {l}_{2})\right]$$

The first term in the parenthesis on the right side corresponds to the thermo-optic effect, where *n* and *α*_*n*_ = 1*/n·∂n/ ∂T* denote the refractive index and the thermo-optic coefficient of silica, respectively. *δl*_1_ and *δl*_2_ represent the variations in coupling length induced by the thermal expansion of the tapered fiber and the microsphere, respectively. The variation in coupling length *δl*_2_ induced by the change in microsphere radius *δR* is given by (details in Supplementary Note 1.3):

4 $$\delta {l}_{2}=2(R+\delta R)\cdot \arccos \frac{R-\varDelta }{R+\delta R}-{l}_{0}$$

where *R* is the microcavity radius, *l*_0_ is the initial coupling length, and *Δ* is the small correction term. Since *δl*_2_ is highly sensitive to small *δR*, a logarithmic function provides an excellent fit for Eq. (4) (Fig. S5). According to the theoretical analysis and numerical simulation results presented in Supplementary Note 1.3, the primary contribution to the right-hand side of Eq. (3) originates from *δl*_2_. Consequently, it can be derived that *δl* ≈ *δl*_2_, where *δl* denotes the variation in coupling length. Furthermore, in the experimental range of variation for *δl*, it has a linear relationship with *Γ*_0_. Using the finite element method (FEM), we calculated the mode field distributions of the WGM cavity and the tapered fiber within the coupling region, as illustrated in Fig. 1f. This subsequently allowed us to determine *δκ*_*ext*_ induced by *δl* (see Fig. S3). A linear dependence between *δκ*_*ext*_ and *δl* is also observed. In the experiment, we define *A* = 1-*Γ*_0_ as the sensor readout. As a result, it can be deduced that *δl* has a linear relationship with *A*. On the other hand, since *δR* = *Rα*_*l*_*δT* (where *α*_*l*_ is the thermal expansion coefficient) and the *δT* is proportional to the gas concentration *N* as analyzed above, *δR* will subsequently be proportional to *N*. Although Eq. (4) does not analytically impose a strictly logarithmic law, the calculated *δl*-*δR* dependence over the small-expansion range relevant to our experiments can be reasonably approximated by a logarithmic-type function. Therefore, an approximately logarithmic dependence of *A* on *N* is obtained, consistent with experimental observations (Fig. 4b).

When the WGM microcavity is set at the under-coupled state (*κ*_*ext*_ <*κ*_*int*_), and the increase in *κ*_*ext*_ will increase *A*. This is consistent with the experimental results (Fig. 3a). It is worth noting that if the WGM microcavity is at the over-coupled state (*κ*_*ext*_ >*κ*_*int*_), the increase in *κ*_*ext*_ will reduce *A*, but the sensitivity will also be reduced greatly. Therefore, in the experiment, the coupling position of the tapered fiber is adjusted to make the microcavity at the under-coupled state, thereby improving the sensitivity for our dissipative WGM sensor. For more theoretical analysis, see details in the Supplementary Note 1.

### Experimental details

A laser driver (Stanford Research Systems, LDC501) was used to precisely control the injection current and temperature of a butterfly-packaged diode laser, providing an output power of approximately 10 mW. CO_2_ concentration measurements were performed using both the direct optical transmission (raw optical mode) and the second-harmonic (2 *f*) signal obtained via modulation–demodulation. For raw signal acquisition, the laser frequency was scanned by applying a 100 Hz triangular waveform with an amplitude of 300 mV from a function generator (Rigol DG1000Z). For 2 *f* signal acquisition, this triangular scan was superimposed with a sinusoidal modulation signal generated by a digital lock-in amplifier (Zurich Instruments MFLI) using an analog summing circuit. The combined waveform was applied to the laser current to achieve wavelength modulation spectroscopy. The laser output was coupled into the tapered fiber through a fiber flange connector, and the transmitted signal was detected by a photodetector (Thorlabs, PDA20CS2). In the raw optical mode configuration, the photodetector output was directly recorded by a data acquisition (DAQ) system (National Instruments, USB-6356). In the 2 *f* configuration, the photodetector signal was first demodulated by the lock-in amplifier and then recorded by the DAQ system. All signal acquisition, fitting, and peak extraction were automated via a LabVIEW-based program. The sample chamber was connected to an integrated gas handling system consisting of a stainless-steel mixing tank, a multicomponent gas blender (Environics S4000), and high-purity gas cylinders containing 1% CO_2_ and 99.9999% N_2_. Gas flows were precisely controlled by the multicomponent blender, ensuring complete homogenization within the mixing tank prior to delivery into the sample chamber for accurate and reproducible measurements. Additional experimental details are available in Supplementary Note 2.

## Supplementary information

Supplementary information

Transparent Peer Review file

## Source data

Source Data

## Data Availability

The data generated in this study are provided as a Source Data file. Extra data that support the findings of this study are available from the corresponding authors upon request. Source data are provided with this paper.
