# Supplementary material for: Sub-parts-per-billion CO2 Detection based on Dissipative Whispering Gallery Mode Microcavity Sensor
Source: Nat Commun. 2026 Jul 4;17:8341. doi: 10.1038/s41467-026-75218-y (PMC13473595; doi:10.1038/s41467-026-75218-y)
Supplement: Supplementary file 1 [file 41467_2026_75218_MOESM1_ESM.pdf]

Supplementary Information for Sub-parts-per-billion CO_2 Detection based on Dissipative Whispering Gallery Mode Microcavity Sensor

Shujing Ruan[1#], Guangzhen Gao[1#], Jianing Zhang[1#], Haotian Wang[1*], Dongxing Cheng[1], Jun Guo[1], Chuanyong Ren[1], Weidong Chen[2], Deyuan Shen[1] & Tingdong Cai[1*]

[1]College of Physics and Electronic Engineering, Jiangsu Normal University, Xuzhou, 221116, China.

[2]Laboratoire de Physicochimie de l'Atmosphère, Université du Littoral Côte d'Opale 189A, 59140 Dunkerque, France.

#These authors contributed equally to this work.

*Corresponding author: e-mail: wanghaotian@jsnu.edu.cn (Haotian Wang), caitingdong@126.com (Tingdong Cai).

Supplementary Note 1：

The readout A of the dissipative WGM sensor is first defined, and its relationship with the coupling strength κ_{ext} is established based on coupled-mode theory. Subsequently, the factors affecting the coupling strength κ_{ext} are analyzed, and it is found that the main contribution comes from the increase in coupling length due to the expansion of the microspheres induced by gas absorption. A model integrating the thermo-optic absorption of CO_2 gas at varying concentrations with heat conduction is developed. This model derives the relationship between the sensor's temperature increase and the coupling strength κ_{ext}, thereby ultimately obtaining a logarithmic functional relationship between the sensor readout A and the gas concentration N. Finally, a quantitative assessment is conducted to validate the consistency between the theoretical predictions and the experimental results.

1.1 Coupled mode equations

The amplitude a of the light field in the WGM microcavity changes with time:

$$\frac{d}{dt}a = -i\omega_0 a - \frac{\kappa_{int}}{2}a - \frac{\kappa_{ext}}{2}a + \sqrt{\kappa_{ext}}a_{in} \tag{S1}$$

where ω_0 is the resonance frequency of the microcavity, a_{in} is the amplitude of the incident laser field from the tapered fiber. κ_{ext} and κ_{int} represent the loss rates due to the inherent loss in the WGM microcavity and the loss rate introduced by coupling, respectively. Assuming that the incident light oscillates at angular frequency ω, we extract the fast-varying terms and analyze only the time evolution of the slow-varying amplitude:

$$a \to \tilde{a}e^{-i\omega t}, \quad a_{in} \to \tilde{a}_{in}e^{-i\omega t} \tag{S2}$$

$$\frac{d\tilde{a}}{dt} = \left[-i\left(\omega_0 - \omega\right) - \frac{\kappa_{int}}{2} - \frac{\kappa_{ext}}{2}\right]\tilde{a} + \sqrt{\kappa_{ext}}\tilde{a}_{in} \tag{S3}$$

When the system is in steady state, i.e., $d\tilde{a}/dt = 0$

$$\tilde{a} = \frac{\sqrt{\kappa_{ext}}\tilde{a}_{in}}{\frac{\kappa_{ext}+\kappa_{int}}{2} - i\left(\omega_0 - \omega\right)} \tag{S4}$$

We can obtain the light intensity transmittance $\Gamma(\omega)$ of the WGM microcavity:

$$\Gamma\left(\omega\right) = \left|\frac{a_{out}}{a_{in}}\right|^2 = \left|\frac{\frac{\kappa_{ext}-\kappa_{int}}{2} + i\Delta}{\frac{\kappa_{ext}+\kappa_{int}}{2} - i\Delta}\right|^2 \tag{S5}$$

where $a_{out} = \sqrt{\kappa_{ext}}a - a_{in}$ is the amplitude of the light field output from the WGM microcavity $\Delta = \omega - \omega_0$ is the frequency detuning between incident laser and the resonance of the microcavity. When the incident light is at the microcavity resonant frequency, the transmittance of the light intensity is:

$$\Gamma_0 = \left(\frac{\kappa_{ext}-\kappa_{int}}{\kappa_{ext}+\kappa_{int}}\right)^2 \tag{S6}$$

For our dissipative WGM sensors, the change in light intensity transmittance at the resonance can be used as the sensor readout A=1-Γ_0, as shown in Fig. 3a of the manuscript. Since the change in κ_{int} caused by light absorption by low-concentration gases is negligible, its main contribution comes from the change in κ_{ext}.

According to Eqs. S5 and S6, assuming κ_{int} is a constant, we calculated the

transmission spectrum of the WGM microcavity at different coupling strengths κ_{ext}, and the relationship between the sensor readout A and κ_{ext}, as shown in Fig. S1. In the under-coupled ($\kappa_{ext} < \kappa_{int}$) and over-coupled ($\kappa_{ext} > \kappa_{int}$) states, the sensor reading A increases and decreases, respectively, with increasing κ_{ext}. Furthermore, the rate of change of A with respect to $\kappa_{ext}/\kappa_{int}$ is much higher in the under-coupled state than in the over-coupled state, at 3.3 and -0.136, respectively. Therefore, in the experiment, setting the dissipative WGM microcavity sensor in the under-coupled state can significantly improve sensitivity.

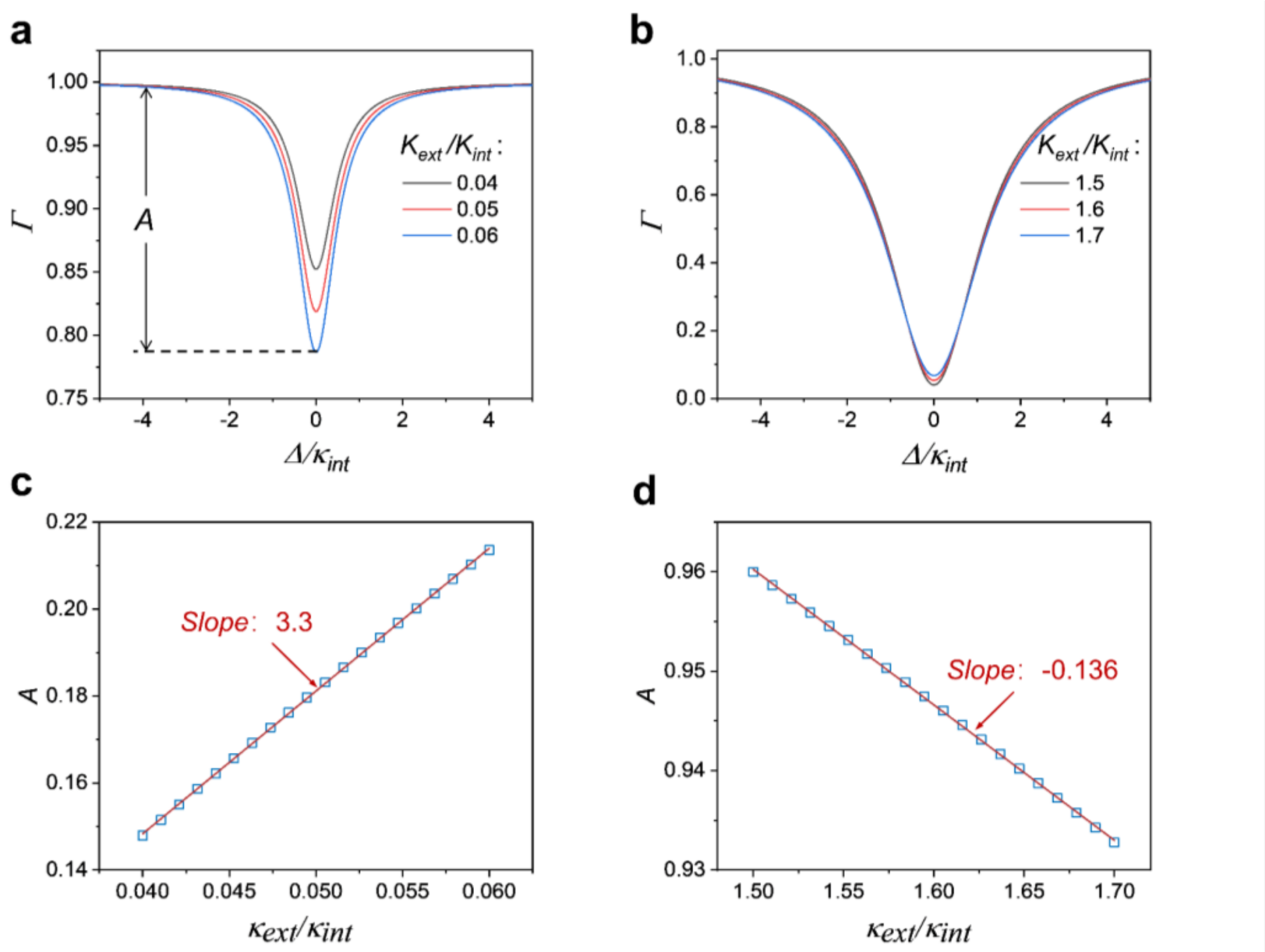

Fig. S1 | The WGM transmission lineshapes of the microsphere cavity under different κ_{ext} in under-coupling **a** and over-coupling **b** states. **c** and **d,** The linear relationship between sensor readout A and κ_{ext} under these two coupling states, respectively.

1.2 Coupling strength

The coupling strength between the tapered fiber and the WGM microcavity depends on the mode field overlap factor and the length of the coupling region:

$$\eta = \frac{\omega\varepsilon_0}{4}\left(n_r^2 - n_0^2\right) N_f N_r \times \iint \left(\boldsymbol{E}_f \cdot \boldsymbol{E}_r^*\right) dxdy \tag{S7}$$

$$\kappa(l)=\eta\frac{\sin\left(\Delta\beta l/2\right)}{\Delta\beta/2} \tag{S8}$$

$$\kappa_{ext}=\left|\kappa\right|^{2}\frac{c}{2\pi n_{r}R} \tag{S9}$$

η is the coupling coefficient between a certain point on the tapered fiber and the WGM microcavity, $\boldsymbol{E}_f$ and $\boldsymbol{E}_r$ are the electric field distributions of the tapered fiber and the WGM microcavity, and N_f and N_r are the corresponding field normalization coefficients. n_r and n_0 are the refractive indices of the tapered fiber and the environment. In practice, a section of tapered fiber is attached to the WGM microcavity surface. Assuming that the point coupling coefficient in the coupling region remains unchanged, a line coupling coefficient κ is generated (Eq. S8), where $\Delta\beta=\beta_f-\beta_r$ is the difference between the propagation constants of the mode for tapered fiber and the microsphere cavity. l is the length of the coupling region. The relationship between the line coupling coefficient κ and κ_{ext} is as shown in Eq. S9, where c and R are the speed of light and the radius of the microsphere WGM cavity.

The mode field distributions of the WGM microcavity and the tapered fiber at the cross-section of the coupling region were calculated using the finite element numerical method. To more clearly illustrate the overlap ratio of the mode field in Eq. S7, we calculated the modes of the microcavity and the tapered fiber separately and plotted them in the same coordinate system. Figs. S2a and S2b show the superimposed mode fields: that is, the distribution of $|\boldsymbol{E}_f|^2/N_f+|\boldsymbol{E}_r|^2/N_r$ in the coupling region. The diameters of the microcavity and the tapered fiber are 60 μm and 0.6 μm, respectively. Fig. S2c shows the distribution of the mode field intensities of the microcavity and the tapered fiber along the white curve in Fig. S2b. When the diameter of the tapered fiber is <1 μm, a large amount of evanescent field will leak from the fiber, thus enhancing its coupling strength with the microcavity.

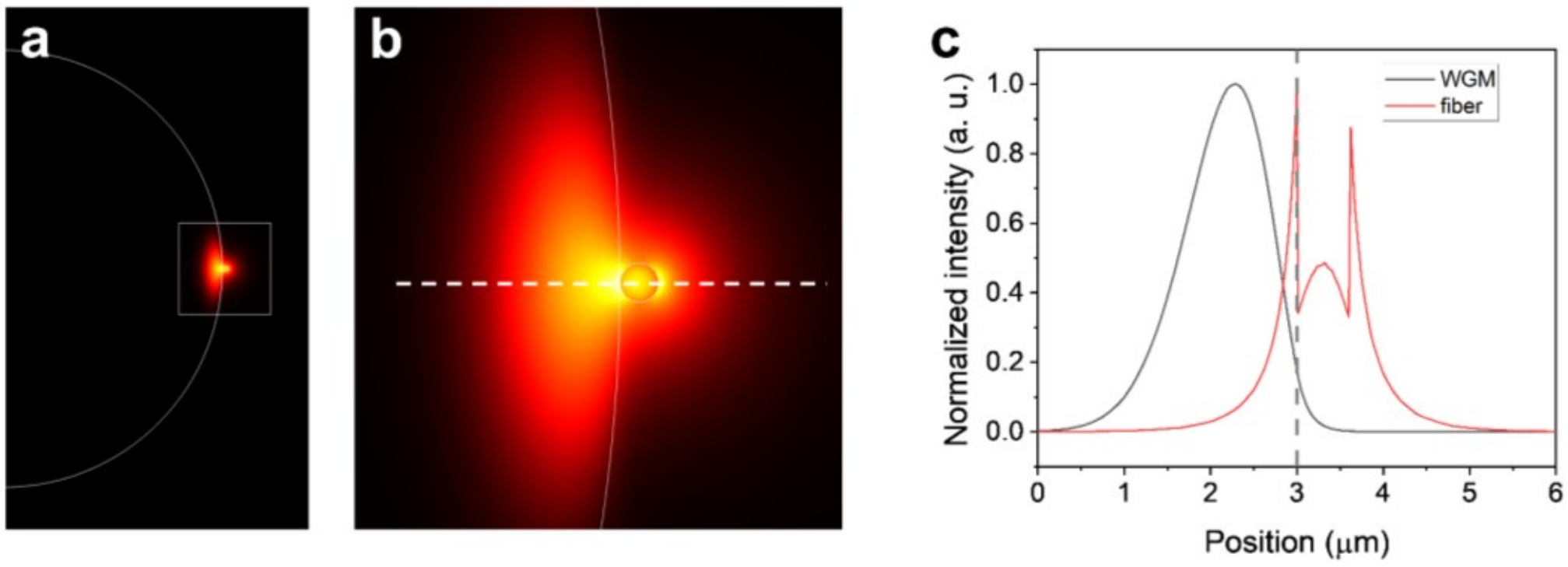

Fig. S2 | **a**, Mode field distribution ($|\boldsymbol{E}_f|^2/N_f+|\boldsymbol{E}_r|^2/N_r$) of microsphere cavity and tapered fiber in the coupling region. **b**, Zoom within the white square in **a**. **c**, The black and red curves represent the mode field distributions of the WGM mode ($|\boldsymbol{E}_r|^2/N_r$) and the tapered fiber mode ($|\boldsymbol{E}_f|^2/N_f$) along the white curve in **b**, respectively. The black vertical curve marks the boundary of the microsphere.

According to Eqs. S7-S9, the relationship between the coupling strength κ_{ext} and the coupling length, fiber diameter, and refractive index was calculated, as shown in Fig. S3. Fig. S3a shows a 2D contour plot of the coupling strength as a function of coupling length and fiber diameter. Initially, the coupling strength increases rapidly with increasing coupling length, but with decreasing fiber diameter, it shows a trend of increasing first and then decreasing. This is because the initial decrease in fiber diameter increases the proportion of the evanescent field, thus increasing the overlap between the mode field of the fiber and the microcavity mode. However, further reducing the diameter causes the fiber mode to deviate from paraxial propagation, i.e., the axial electric field component increases, which significantly reduces its overlap with the microcavity mode, specifically the numerator of the integrand in Eq. S7. Fig. S3b shows a 2D contour plot of the coupling strength as a function of coupling length and refractive index. At a fixed coupling length, the change in coupling strength is only on the order of kHz within a refractive index change of δn = 0.0003. Fig. S3d shows the sensitivity of the coupling strength to the refractive index: -2.1 MHz/RIU. Since the refractive index n changes only by an order of 10^{-5} for a 1 K temperature variation, the influence of the thermo-optic effect can be ignored in our sensor.

Because the coupling strength κ_{ext} varies only by a few MHz in the experiment, it exhibits a linear relationship with the coupling length within this range, as shown in Fig. S3c. At a tapered fiber diameter of 0.6 μm, the sensitivity of the coupling strength to the coupling length is 1.24 MHz/nm.

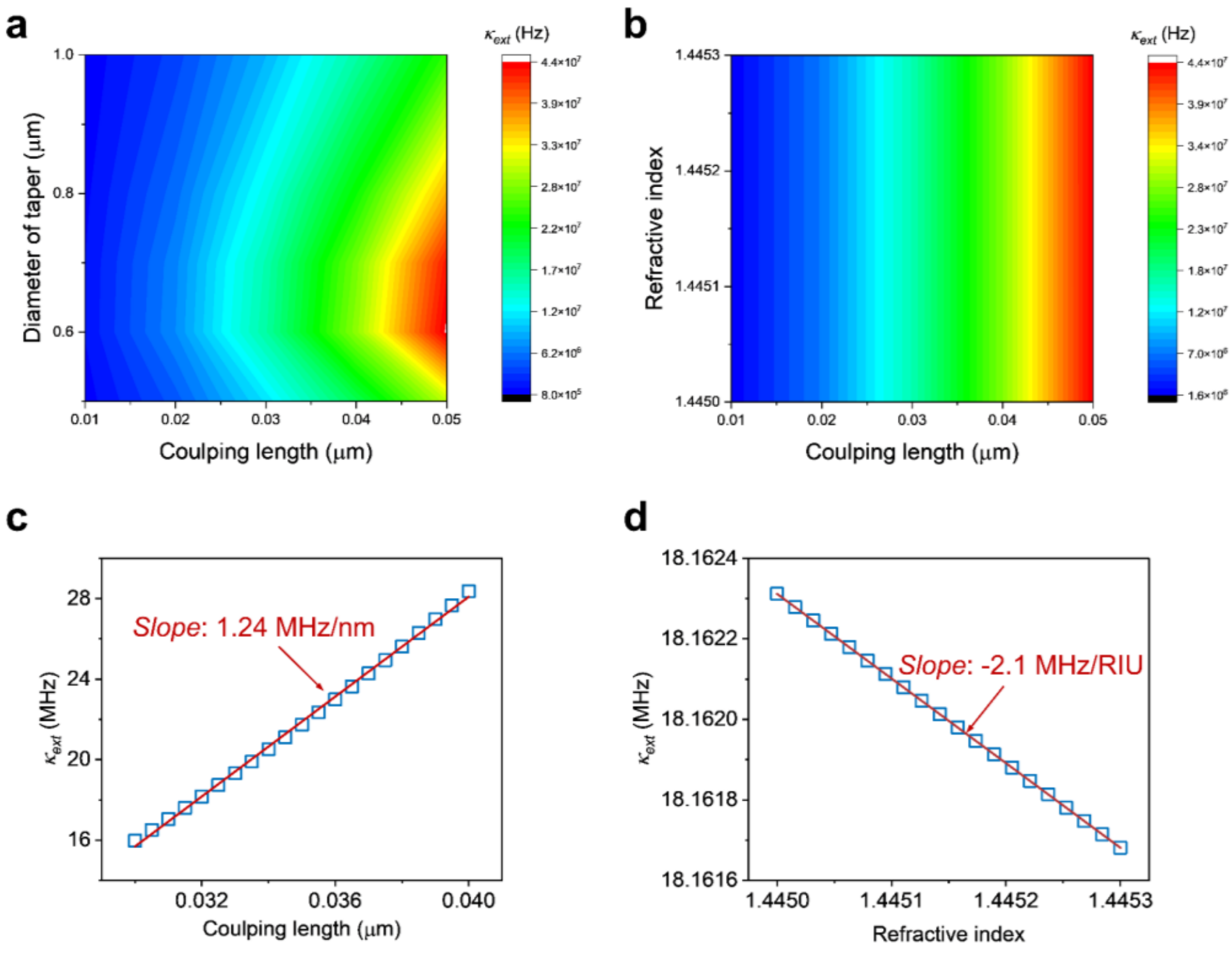

Fig. S3 | **a**, Two-dimensional contour plot of coupling strength varying with coupling length and tapered fiber diameter. **b**, Two-dimensional contour plot of coupling strength varying with coupling length and refractive index. **c**, The linear relationship between coupling strength and coupling length when the diameter of the tapered fiber is 0.6μm. **d**, The linear relationship between coupling strength and refractive index when the tapered fiber diameter is 0.6μm and the coupling length is 30 nm.

1.3 Temperature-induced change in coupling strength

In this section, we will analyze the effect of temperature changes on κ_{ext}. The change of κ_{ext} is mainly caused by the heat generated by the gas absorbing light, which will be written as:

$$\delta\kappa_{ext} = \frac{\partial\kappa_{ext}}{\partial n} n\alpha_n \delta T + \frac{\partial\kappa_{ext}}{\partial l}\delta l \qquad \text{(S10)}$$

The first term on the right-hand side of the above equation represents the contribution due to the thermo-optic effect, where $\alpha_n = (1/n)\cdot(\partial n/\partial T)$ is the thermo-optic coefficient of silica.

The change in coupling length δl due to temperature has two main contributions. Firstly, the heat generated by the absorption of light by the gas is conducted along the tapered fiber, causing the tapered fiber to heat up and expand within a certain range of the coupling region, resulting in a change of δl_1:

$$\delta l_1 = l_T \alpha_l \delta T \tag{S11}$$

where $\alpha_l = 1/l\cdot\partial l/\partial T$ is the thermal expansion coefficient of quartz, and l_T is the heated tapered fiber region.

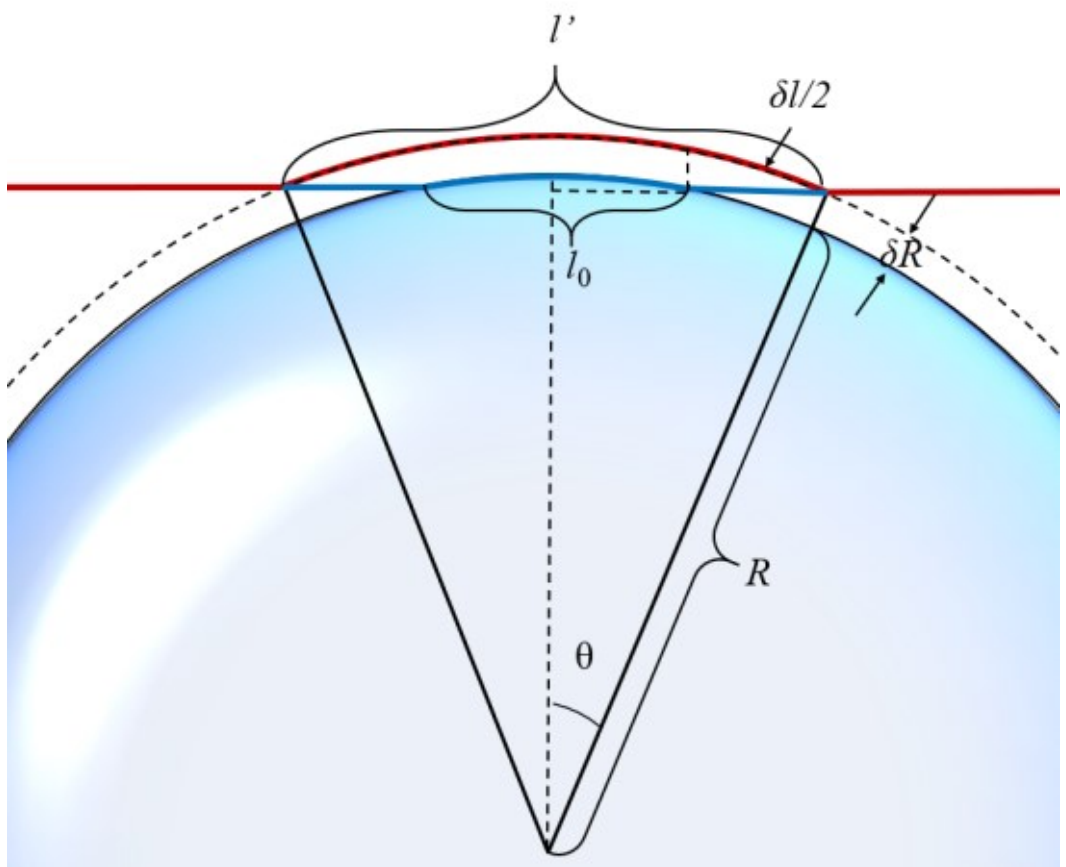

Fig. S4 | Changes in coupling length caused by microsphere expansion before (blue curve) and after (red curve) temperature increase. The black dashed line represents the edge of the microsphere after expansion.

On the other hand, light absorption by the gas causes thermal expansion of the microsphere microcavity, which in turn extends the length of the tapered optical fiber attached to the surface of the microcavity. As shown in Fig. S4, when the tapered fiber is coupled to the microsphere cavity, a section of the fiber is in contact with the microsphere surface, and the coupling region appears as an arc. The blue and red arcs represent the coupling length before and after expansion, respectively, and the solid and dashed black lines represent the edge contours of the microsphere before and after

expansion. Based on the geometric relationship in the figure, the change in coupling length δl_2 caused by the change in microcavity radius can be approximately expressed as:

$$\delta l_2 = l' - l_0 = 2\left(R + \delta R\right) \cdot \arccos\frac{R - \varDelta}{R + \delta R} - l_0 \tag{S12}$$

where R is the microcavity radius, δR is the expansion of the microcavity radius, and l_0 is the initial coupling length. In the above derivation, we assumed that the angle θ is very small, which is reasonable because the microsphere radius is on the order of tens of micrometers, while the coupling length l_0 is only on the order of tens of nanometers. The value of the correction term $\varDelta$ can be determined by ensuring that when $\delta R = 0$, δl_2 must be zero in the numerical calculation of Eq. S12.

According to Eqs. S10-S12, the change in coupling strength due to temperature variations can be derived:

$$\begin{aligned} \delta\kappa_{ext} &= \frac{\partial\kappa_{ext}}{\partial n} n\alpha_n \delta T + \frac{\partial\kappa_{ext}}{\partial l}\left(\delta l_1 + \delta l_2\right) \\ &= \frac{\partial\kappa_{ext}}{\partial n} n\alpha_n \delta T + \frac{\partial\kappa_{ext}}{\partial l}\left[l_T \alpha_l \delta T + 2\left(R + \delta R\right) \cdot \arccos\frac{R - \varDelta}{R + \delta R} - l_0 \right] \end{aligned} \tag{S13}$$

where $\delta R = R\alpha_l \delta T$.

According to Eqs. S6, S10, and S13, the relationship between the sensor readout A and the temperature change δT can be derived. The first, second, and third terms on the right side of Eq. S13 represent the changes in coupling strength due to the thermo-optic effect, the expansion of the tapered fiber, and the expansion of the microsphere, respectively. Based on the calculation results in Fig. S3d, the change in coupling strength caused by the thermo-optic effect in the experiment is negligible. Furthermore, according to the heat conduction model (see the analysis in Supplementary Note 1.4), the heated region of the tapered fiber is only tens of micrometers, and its contribution to the change in coupling strength is also negligible. Therefore, the change in coupling strength mainly depends on the expansion of the microsphere WGM cavity, i.e., $\delta l \approx \delta l_2$.

As shown in Fig. S5, the blue squares represent the numerical results using Eq.

S12. In the calculation, l_0, R, and Δ were set to 20 nm, 30 μm, and 1.65 pm, respectively. Clearly, there is a nonlinear relationship between δl_2 and δR. In Fig. S5, we performed both a 5th-order polynomial fit and a logarithmic fit to Eq. S12, yielding correlation coefficients of R^2 = 0.9998 and R^2 = 0.9947, respectively. Although Eq. S12 does not analytically impose a strictly logarithmic law and does not exhibit lower fitting residuals compared to the 5th-order polynomial, the logarithmic function provides a more intuitive and useful physical picture. The fitted logarithmic function is:

$$\delta l = 268 + 64\ln\left(\delta R + 0.015\right) \tag{S14}$$

According to Figs. S1c and S3c, we find that the sensor readout A is proportional to the change in coupling length δl. On the other hand, due to the expansion of the microcavity caused by gas absorption, at low concentrations, the gas concentration N is proportional to the temperature increase of the microcavity (see Supplementary Note 1.4), and consequently proportional to the change in microsphere radius δR. According to Fig. S5, δl has a logarithmic-type relationship with δR; therefore, it can be deduced that the sensor readout A has a logarithmic-type relationship with the gas concentration N, which is consistent with the experimental results shown in Fig. 4 of the manuscript. Compared to higher-order polynomials, logarithmic functions possess fewer fitting parameters, enabling the rapid retrieval of gas concentrations.

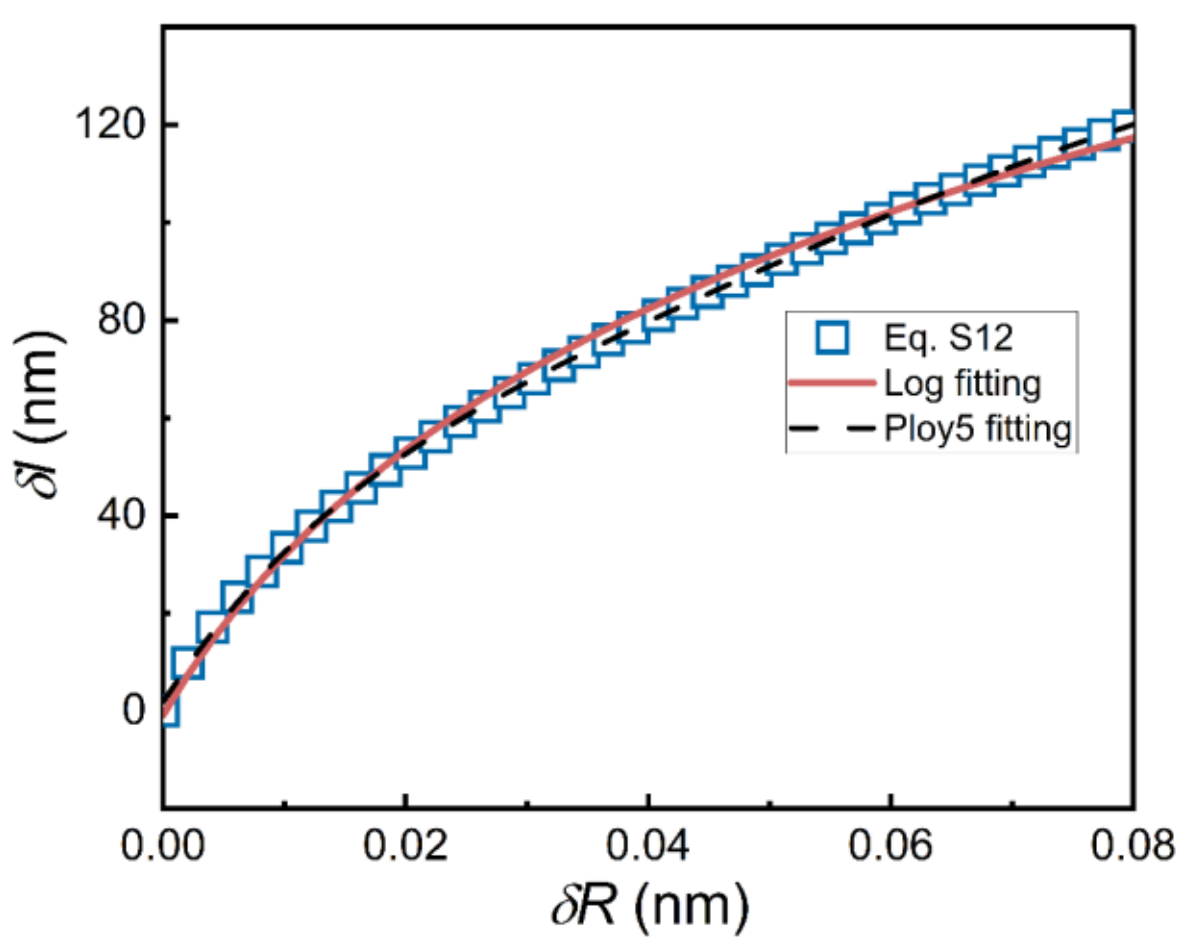

Fig. S5 | The relationship between the change in coupling length δl and the change in microsphere radius δR. Blue squares: Calculated from Eq. 12; Red curve: Logarithmic

fit; Black dashed line: 5th-order polynomial fit.

Based on Eq. S12, δl exhibits an approximately linear dependence on δR at sufficiently low gas concentrations. Consequently, the sensitivity does not increase indefinitely as the concentration decreases, establishing a definitive limit of detection (LOD). Therefore, the sensitivity of the sensor increases as the concentration decreases, reaching a maximum at the LOD.

1.4 Gas absorption induces temperature rise

According to the Beer-Lambert law, the intensity of light absorbed by a gas is:

$$\Delta I = I_0\left(1-e^{-\alpha(\nu)L}\right) \tag{S15}$$

where I_0 is the initial light intensity, L is the interaction distance in the gas, and α is the absorption coefficient, which is related to the laser frequency ν:

$$\alpha(\nu) = Ng(\nu-\nu_0)S \tag{S16}$$

where N represents the gas molecular number density, S is the absorption line strength, and $g(\nu\text{-}\nu_0)$ is the gas absorption line shape function. In the experiment, the linewidth of the laser used is much smaller than the absorption linewidth, and the laser frequency is set at the absorption peak; therefore, the line shape function only needs to consider g_0 at ν_0. For a laser wavenumber of 6359.967 cm^{-1}, g_0 and S are 4 cm and 1.76×10^{-23} $cm^{-1}/mol\cdot cm^{-2}$, respectively. The relationship between the absorption coefficient and gas concentration is $\alpha = k\cdot N$, where $k = 7.04 \times 10^{-23}$ cm^2/mol. Therefore, for weak absorption, i.e., $\alpha L << 1$, Eq. S15 can be written as:

$$\Delta I = I_0\alpha(\nu)L \tag{S17}$$

Therefore, the heating power is:

$$P_Q = I_0\alpha L A_Q = I_0\alpha V \tag{S18}$$

A_Q and V represent the heating area and volume, respectively. Under the adiabatic approximation (i.e., neglecting heat conduction), the temperature increase per unit time in the heated region is:

$$\Delta T = \frac{P_Q}{\rho c_v V} = \frac{I_0 \alpha}{\rho c_v} \tag{S19}$$

where ρ and c_v are the density and specific heat capacity of quartz, respectively. In our experiment, I_0 is the light intensity density inside the microsphere WGM cavity, which is significantly enhanced when the laser frequency approaches the microcavity resonance.

$$I_0 = B\Gamma \frac{P_{in}}{A_{eff}} \tag{S20}$$

P_{in} is the incident power in the tapered fiber, A_{eff} is the mode field area of the WGM in the microcavity, and B is intracavity intensity enhancement factor for the WGM microcavity. The inclusion of the Γ factor accounts for the evanescent field portion of the optical mode interacting with the gas, which leaks outside the microcavity. Furthermore, since the tapered fiber is in close contact with the microcavity surface, strong scattering occurs at the coupling point, significantly increasing the leakage of the light from the microcavity into the surrounding air. Combining Eqs. S19 and S20, the heating rate of the microcavity due to gas absorption at a concentration of 1 ppm can be estimated to be approximately 0.04 K/s, with the parameters used shown in Table S1.

Table S1 The parameter values used for numerical estimation

Parameter	Symbol	Value
Incident power	P_{in}	10 mW
Mode field area	A_{eff}	3 μm^2
Absorption peak	g_0	4 cm
Absorption line strength	S	1.76×10^{-23}cm^{-1}/mol·cm^{-2}
Gas molecular number density	N (1 ppm)	1.83×10^{13} mol/cm^3
Thermal expansion coefficient	α_l	0.55×10^{-6} K^{-1}

Thermo-optic coefficient	α_n	10×10^{-6} K^{-1}
Silica density	ρ	2.2 g/cm^3
Temperature	T	300 K
Specific heat capacity	c_V	0.67 J/(g·K)
Constant pressure heat capacity	C_p	0.703 J/(g·K)
Thermal conductivity	k	1.38 W/(m*K)
Light intensity enhancement factor	$B{\cdot}\Gamma$	~100

Considering that the generated heat diffuses into the microsphere microcavity, the tapered fiber, and the surrounding air, the heat conduction equation is needed:

$$\rho C_p \frac{\partial T}{\partial t} - \nabla \cdot (k\nabla) = Q \tag{S21}$$

where T represents temperature, and C_p represents the constant-pressure heat capacity, which for silica is approximately equal to the specific heat capacity c_V. k and Q represent thermal conductivity and generated heat, respectively. Since the composite structure of the tapered fiber and microsphere microcavity is an irregular geometry, a numerical method is required to solve Eq. S21. As described in Fig. 1b in the manuscript, we used a heating ring surrounding the equator of the microsphere to simulate gas absorption-induced heating. The microsphere and tapered fiber were enclosed within a large, air-filled cube. The boundaries of the cube are set to a constant ambient temperature to simulate the large heat capacity of the surrounding environment. The steady-state temperature distribution is shown in Fig. S6. Fig. S6a shows the temperature distribution at a heat generation per unit volume of $Q = 0.6\times10^{6}$ W/m^3 (corresponding to 3 ppm gas absorption), and Fig. S6b shows the temperature variation along the white curve in Fig. S6a. It can be seen that due to heat conduction, the temperature rise is only 0.0012 K compared to the adiabatic condition described in Eq. S19, and the temperature distribution is relatively uniform within the microsphere cavity. The heat does not propagate specifically along the tapered fiber, but rather diffuses into the surrounding

air over a range of tens of micrometers. This is because the thermal conductivity cross-sectional area of the tapered fiber is much smaller than that of the surrounding air, even though its thermal conductivity is much greater than that of air.

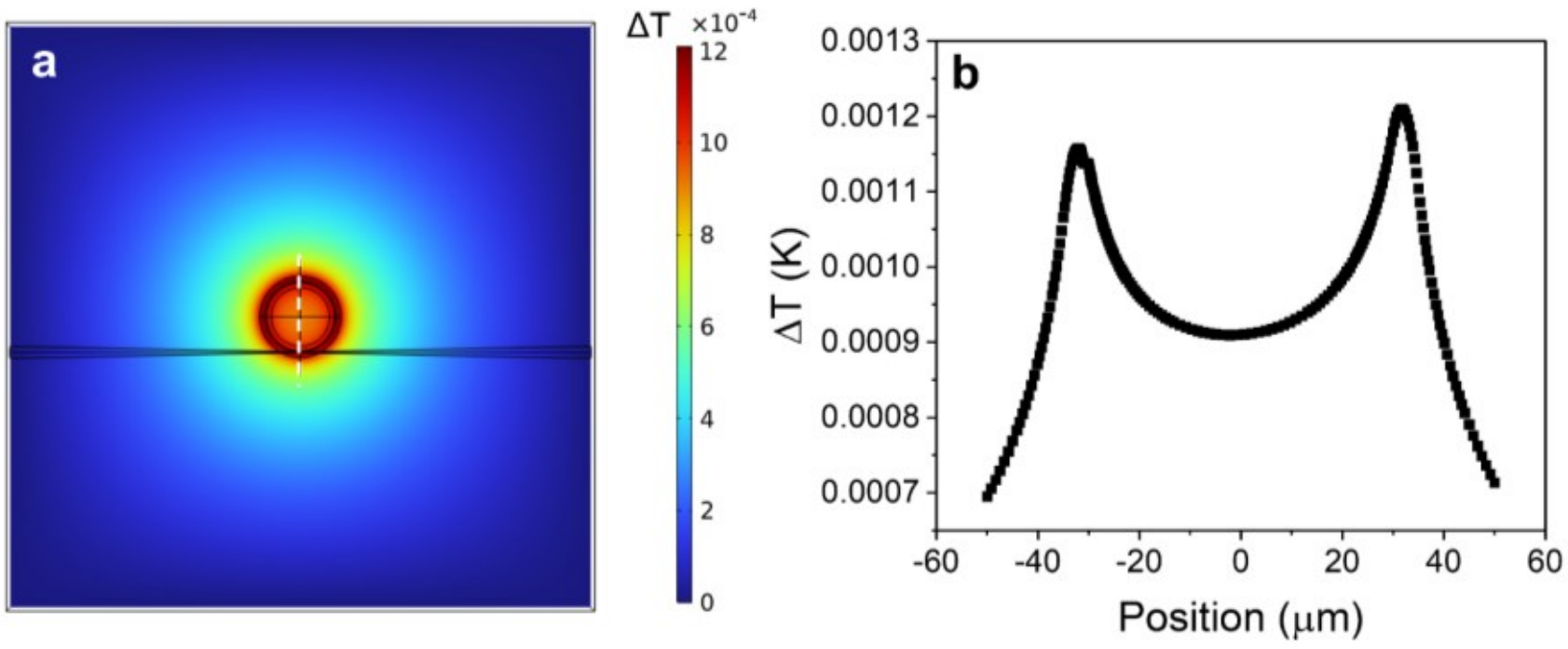

Fig. S6 | **a**, Temperature variation distribution on the equatorial plane of the microsphere. **b**, Temperature variation along the white dashed line in **a**.

1.5 Comparison between theoretical estimation and experimental results

In this section, we use the theoretical model described above to perform numerical estimations and validate the model against experimental results. According to Fig. 4a in the manuscript, the experimentally measured signal change is 0.005 (normalized) for a gas concentration change of 1.5 ppm (from 1.5 ppm to 3 ppm). Based on the sensitivity of the coupling strength to the sensor readout (~3.3) shown in Fig. S2c of Supplementary Note 1.1, this signal change requires a change in coupling strength κ_{ext} of approximately 0.45 MHz. Here, we assume the intrinsic loss of the microsphere is 300 MHz, corresponding to an intrinsic Q factor Q_{int} ~ 4 × 10^6. Then, using the sensitivity of κ_{ext} to the coupling length l obtained from Fig. S4 in Supplementary Note 1.2: ~1.24 MHz/nm, we can deduce that the coupling length change δl is 0.36 nm. From Eq. S12 and the logarithmic fitting curve in Fig. S5 of Supplementary Note 1.3, we obtain the slope near the zero point, $\delta l/\delta R \sim 5 \times 10^3$. Therefore, a δl change of 0.36 nm corresponds to a δR change of 0.07 pm. Using the thermal expansion formula: $\delta R = R{\cdot}\alpha_l{\cdot}\delta T$, and substituting the microsphere radius of 30 μm and the thermal expansion coefficient $\alpha_l = 0.55 \times 10^{-6}$, we obtain a required temperature change of 0.004 K. This result agrees well with the calculated temperature increase due to gas absorption at 3

ppm shown in Fig. S6 (0.0012 K). Therefore, the validity of our theoretical model can be proven.

1.6 Sensor noise floor analysis

In this section, we will estimate the noise floor of our WGM sensor. Even if the temperature remains constant when the gas concentration is fixed, the WGM microcavity will produce spontaneous temperature fluctuations due to Brownian thermal motion, and the intensity noise of the laser itself will also introduce noise to the sensor:

$$I_{out} = \Gamma\left(\omega_0\right) I_{in} \tag{S22}$$

$$\delta I_{out} = \Gamma\left(\omega_0\right)\delta I_{in} + \delta\Gamma\left(\omega_0\right) I_{in} \tag{S23}$$

$$\frac{\delta I_{out}}{I_{out}} = \frac{\delta I_{in}}{I_{in}} = \frac{\delta \Gamma_0}{\Gamma_0} \tag{S24}$$

The first and second terms on the right side of Eq. S24 represent the fluctuation of the transmitted light intensity due to the laser power fluctuation and the WGM microcavity spontaneous thermal noise, which is the noise floor of the sensor. Therefore, its noise power spectral density (PSD) is expressed as:

$$S_{\delta I/I}\left(f\right) = \mathrm{RIN}\left(f\right) + S_{\delta I/I}^{T}\left(f\right) \tag{S25}$$

where RIN represents the relative noise of the laser. Figure 1 in the main text shows the laser RIN measured in our experiment, which also includes the background noise of the detection and data acquisition system. $S_{\delta I/I}^{T}\left(f\right)$ is the PSD of the sensor noise introduced by the spontaneous temperature fluctuation, which is given by:

$$S_{\delta I/I}^{T}\left(f\right) = \left(\frac{4\kappa_{int}}{\kappa_{ext}^{2} - \kappa_{int}^{2}}\right)^{2}\left[\left(\frac{\partial \kappa_{ext}}{\partial l}\frac{\partial l}{\partial T}\right)^{2} + n^{2}\alpha_{n}^{2}\left(\frac{\partial \kappa_{ext}}{\partial n}\right)^{2}\right] S_{\delta T}\left(f\right) \tag{S26}$$

The noise in Eq. S26 is also due to thermal expansion and refractive index fluctuation caused by temperature change. According to the analysis in the previous section, the refractive index fluctuation of the sensor in the experiment is much smaller than thermal expansion, so it can be ignored. $S_{\delta T}\left(f\right)$ is the PSD of the fluctuations of temperature

in the microcavity and can be expressed as:

$$S_{\delta T}\left(f\right)=\frac{k_B T^2}{\rho C V_r}\frac{2R^2/\pi^2 D}{1+\left(2fR^2/D\pi\right)^2} \tag{S27}$$

where ρ, C, D, V_r, R and T represent the density, heat capacity, thermal diffusion coefficient, microcavity volume, microsphere radius and temperature of the microcavity, respectively. k_B is the Boltzmann constant. At room temperature, and using the parameters in Supplementary Note 2, we estimate that $S_{\delta T}\left(f\right)$ is less than 10^{-18} K^2/Hz and decreases rapidly after f >10kHz. Then, according to Eq. S26, $S_{\delta I/I}^{T}\left(f\right)$ is estimated to be less than -180 dBc/Hz, which is much smaller than the RIN of the laser, so this part of the noise can be ignored in our sensor.

Supplementary Note 2

2.1 Fabrication Processes of Tapered Fiber and Microspheres

2.1.1 Tapered Fiber Fabrication Procedure

Fiber Preparation: A standard single-mode fiber is stripped of its coating (approximately 2–3 cm in length) to expose the bare fiber, followed by surface cleaning with alcohol.

Heating and Tapering: The fiber is uniformly heated using a hydrogen-oxygen flame while being stretched at a constant speed via a precision translation stage or stepper motor. Real-time Monitoring: The transmitted optical power is continuously monitored using a photodetector to minimize taper-induced losses. Taper Formation: The fiber diameter is reduced from 125 μm to <1 μm, forming a tapered waist. The length and diameter of the taper region are determined by the stretching speed, heating temperature, and duration.

2.1.2 Microsphere Fabrication Procedure

Pre-tapering: A quartz fiber is first tapered to approximately 20 μm using the aforementioned method and then cleaved. Arc Discharge Melting: The fiber tip is fixed between electrodes and melted using a high-voltage arc discharge (temperature > 2000°C). Microsphere Formation: Due to surface tension, the molten quartz forms a

spherical structure. Size and Shape Control: The diameter and morphology of the microsphere can be adjusted by regulating the discharge current, duration, and number of discharges.

2.2 Experimental Methods

2.2.1 Optical Setup

The tapered fiber passes through the absorption cell's optical window and is aligned with the microsphere under a long-working-distance microscope (NAVITAR, 67X ADAPTER). Laser light from a tunable diode laser (NEL, NLK1L5GAAA) was coupled into the taper; the output was directed to a low-noise InGaAs photodetector (Thorlabs, PDA20CS2). Fine adjustment of the laser temperature controller (Stanford Research Systems, LDC501) tuned the emission wavelength to the target absorption line. A 100 Hz triangular waveform was injected into the scan current to produce a wavelength-scanning output, and during modulation a 16.19 kHz sine wave was concurrently superimposed to generate the second-harmonic ($2f$) signal, thereby improving the system's signal-to-noise ratio and enhancing detection sensitivity.

2.2.2 Sealed Chamber Configuration

A three-axis micro-positioning stage (DEYU, DY-XY13) was mounted inside the sealed chamber to align the tapered optical fiber. The microsphere resonator was secured adjacent to the taper using a custom-fabricated stainless-steel clamp, ensuring sub-micrometer stability. Two 0.5 mm through-holes were drilled in the cell wall to accommodate the fiber feedthroughs; after insertion, each port was sealed with optical-grade epoxy (NANDA, 703) to maintain gas tightness. The internal fiber leads were fusion-spliced (Fujikura, 62S+) to external single-mode fibers, completing the light-path interface. To enable measurements of different gas samples, the sealed chamber was equipped with one inlet connected to the integrated gas handling system and one outlet connected to a dry scroll pump (Aoxin Precision Instrument, 2XZ-4 rotary vane vacuum pump), allowing controlled gas flow through the chamber.

2.2.3 Gas-mixing system

Gas samples were delivered from high-purity cylinders containing 1 % CO_2 and

99.9999 % N_2 (Xuzhou Special Gas) through stainless-steel tubing to a multicomponent gas blender (Environics S4000) integrated with a stainless-steel mixing tank. The blender precisely regulated gas flows to achieve complete homogenization within the tank, producing accurate and reproducible concentrations in the 0–500 ppm range. Prior to each measurement, the sealed chamber was evacuated to ~100 Pa using the dry rotary-vane pump and then filled with the prepared standard gas at 1 atm. The chamber outlet was connected to a catalytic converter and activated-carbon scrubber before venting, ensuring safe handling of exhaust gases. This configuration enables controlled introduction of different gas mixtures into the chamber while preserving the integrity of the sealed optical setup, ensuring high reproducibility and reliability of CO_2 sensing experiments.

2.2.4 Sub-ppb CO_2 sample generation and calibration

(1) Preparation of Dilution Gas

Before generating ultra-low-concentration mixtures, the mixing tank and gas lines were repeatedly purged with high-purity N_2 and evacuated to remove residual gases. This procedure minimized background contamination within the system. The background CO_2 concentration of the purified N_2 was verified to be below 0.2 ppb using a mid-infrared gas analyzer prior to the dilution process, ensuring negligible contribution from the diluent gas during sub-ppb mixture preparation.

(2) Iterative Dilution Procedure

Sub-ppb CO_2 mixtures were generated using a controlled multi-step dilution process in the mixing tank.

First, the multicomponent gas blender introduced proportional flows of 1 % CO_2 standard gas and purified N_2 into the tank to produce an intermediate mixture at the ppb level (typically ~150 ppb). The gas mixture was allowed to equilibrate inside the tank to ensure complete homogenization.

Subsequently, an automated evacuate-and-backfill cycle was applied. During each cycle, approximately 90 % of the tank volume was evacuated and immediately replaced with purified N_2. According to the gas dilution law, the CO_2 concentration decreases to

10 % of its previous value after each cycle. The number of dilution cycles required to reach the target concentration was calculated from the initial intermediate concentration.

Through this iterative dilution procedure, stable and reproducible sub-ppb CO_2 mixtures were obtained.

(3) Concentration Verification and Gas Delivery

The final CO_2 concentration in the mixing tank was verified using a high-precision mid-infrared Off-Axis Integrated Cavity Output Spectroscopy (OA-ICOS) analyzer (GMM-1000), which provides a measurement precision better than 0.1 ppb.

After verification, the prepared gas mixture was delivered from the mixing tank to the sealed sensing chamber through stainless-steel tubing. The gas flow was regulated by the multicomponent gas blender to ensure stable exposure conditions during sensing measurements. The accuracy of the prepared concentrations is traceable to the certified standard gas concentration, the calibrated flow-control system of the gas blender, and the precisely controlled volume exchange during the dilution cycles. The combined expanded uncertainty (k=2) of the prepared concentrations in the ppb range is estimated to be better than ±0.3 ppb.

The lowest concentration tested in this work (~0.2 ppb) approaches the detection limit of the sensing system and is therefore used primarily to evaluate the sensor response near the limit of detection.

2.2.5 Sample Measurement and Data Acquisition

The sample gas was introduced into the cell and allowed to equilibrate for 3 min before data acquisition. The photodetector output was split: one channel fed a 16-bit data-acquisition card (National Instruments, USB-6356) to record the raw whispering-gallery-mode transmission, while the other was fed into a lock-in amplifier (Zurich Instruments, MFLI) for $2f$ demodulation. For each concentration step, ten scans were averaged to minimize stochastic variations. All data were processed in LabVIEW with custom scripts for baseline correction, profile fitting, and peak-height extraction.

Supplementary Note 3

3.1 Dynamic Range Analysis

As shown in Eq. S9, the second term in the brackets, the thermo-optic effect, has a negligible effect on the sensor signal change compared to the first term, the thermal expansion effect. Therefore, within a limited variation range of κ_{ext}, the sensor signal can be approximated as proportional to the temperature change δT in the coupling region. For small variations in gas concentration, the temperature rise induced by optical absorption can also be treated as linearly dependent on concentration. Therefore, within this limited range, the amplitude of the resonant mode signal can be considered to have a linear relationship with gas concentration. Similarly, the amplitude of the $2f$ signal can also be approximated as linearly dependent on concentration. The linear fitting results are shown in Figs. S7 and S8, with correlation coefficients exceeding 0.965, indicating good linear response characteristics of the system within the specified concentration range.

This linear response range not only facilitates system calibration at a single concentration point but also supports reliable quantitative measurements in scenarios where concentration fluctuations are relatively small.

3.2 Local Linearity Analysis

A linear response is observed within a limited concentration range. The following three plots illustrate the linear response of the raw optical mode signal over different concentration intervals.

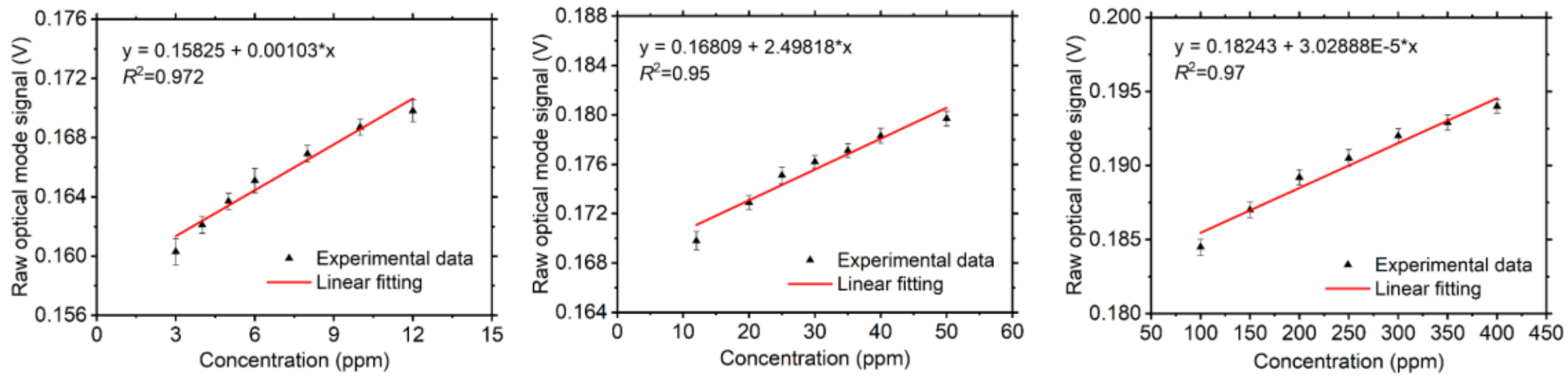

Fig. S7 | Linearity of the raw optical mode signal in various concentration ranges.

Similarly, the linear response of the $2f$ signal over different concentration intervals is presented.

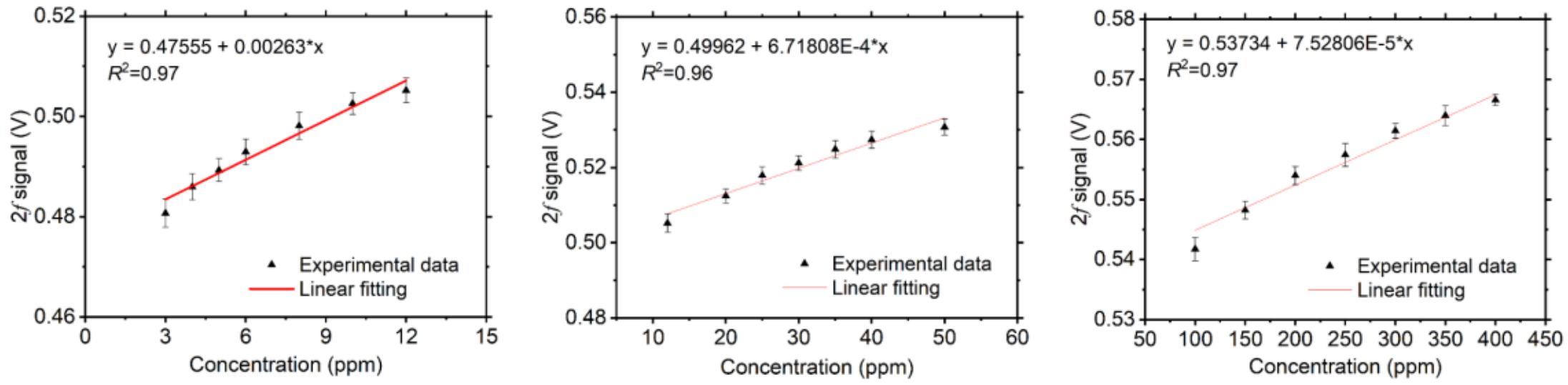

Fig. S8 | Linearity of $2f$ signal in various concentration ranges.

3.3 A portion of the raw optical mode signals and $2f$ signals obtained

A portion of the raw optical mode and $2f$ signals obtained during a continuous 4000 s measurement of a CO_2-N_2 mixture with a concentration of 1.5 ppm is shown below.

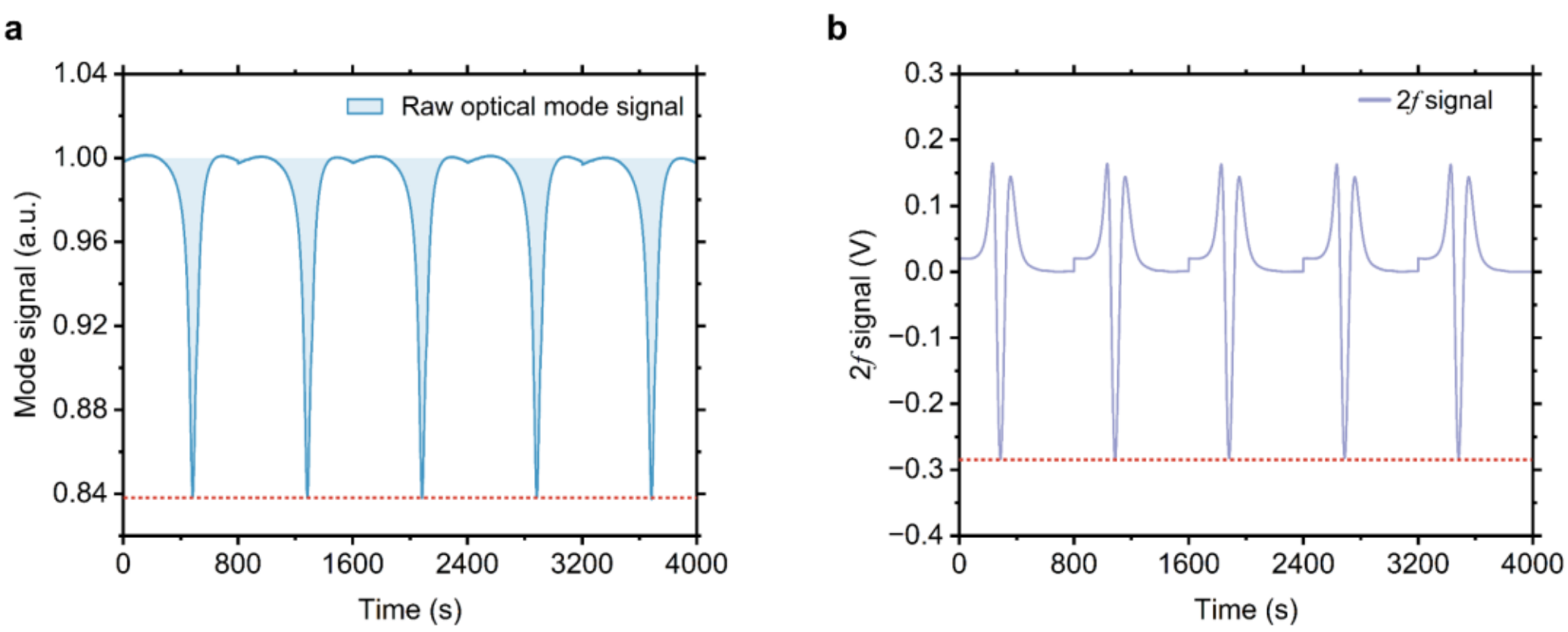

Fig. S9 | a, Representative segment of the raw optical mode signa measured in a CO_2-N_2 mixture with a concentration of 1.5 ppm. **b**, Representative segment of the corresponding 2f signal.

Supplementary Note 4

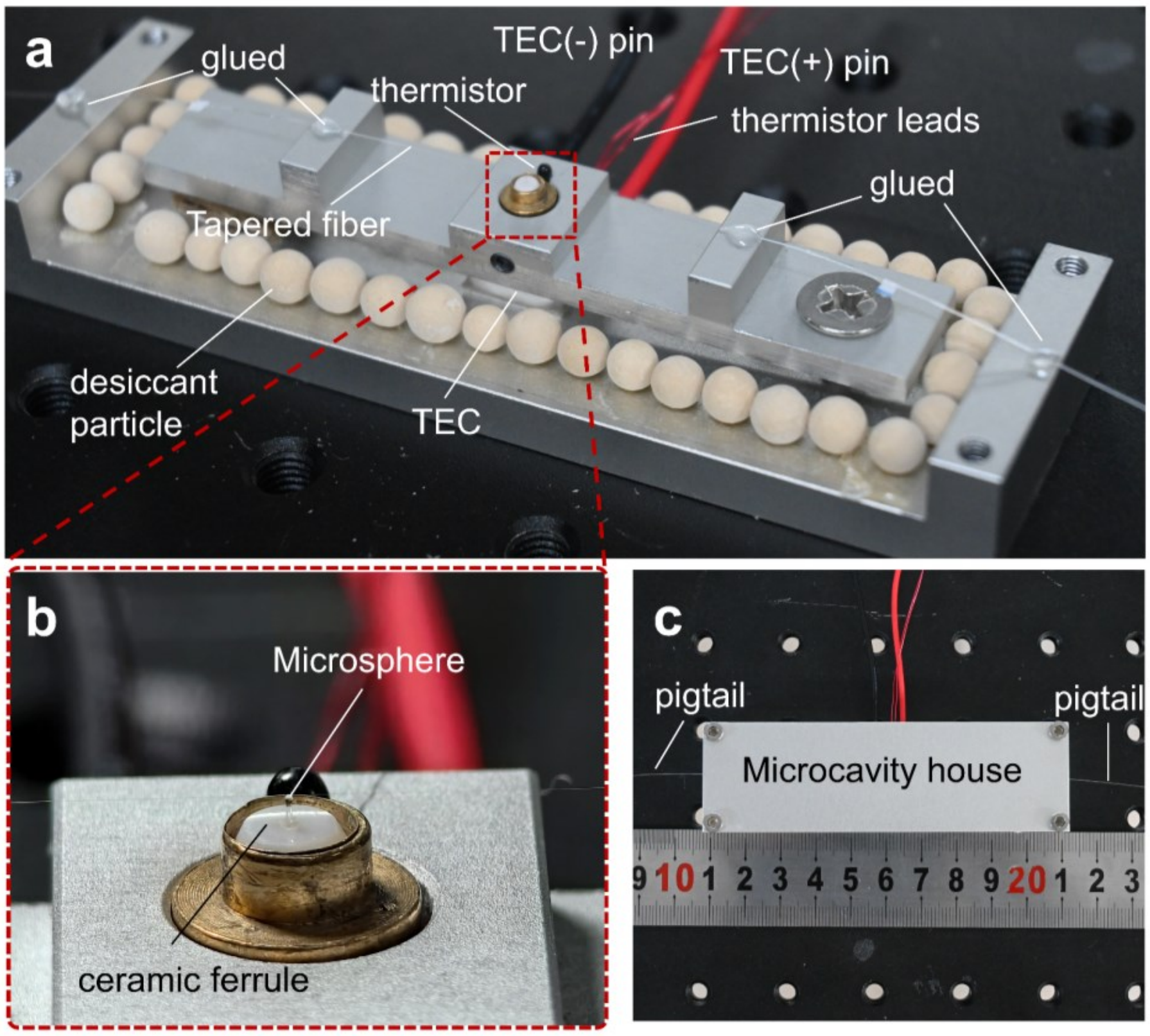

Fig. S10 | a, Packaging structure of tapered fiber coupled with microsphere WGM microcavity. **b**, Zoom-in view of the red dashed square in Fig. a. **c**, Photograph of the fully packaged sensor device.

To enhance the robustness of the WGM microcavity sensor, we fabricated a device package for the microsphere cavity with the tapered fiber, as shown in Fig. S10. First, the microsphere stem was inserted into a standard single-mode fiber ceramic ferrule, with only a small portion of the stem and microsphere exposed. This effectively suppressed forced vibrations of the microsphere caused by external forces, improving the mechanical stability of the microsphere microcavity.

The entire ceramic ferrule was tightly encased in a copper sleeve, with thermal grease filling the gaps between them. The copper sleeve was then mounted onto an aluminum bracket. A thermistor was attached to one side of the copper sleeve for first-stage of temperature control. The first stage of temperature control primarily acts on the small volume near the microsphere, resulting in better local temperature stability. After optimizing the coupling position of the tapered fiber to the microcavity, the

tapered fiber was fixed to the aluminum base with low-thermal-expansion UV glue. Multiple glue points were used along the fiber axis to dampen the effects of external vibrations on the coupling region. At this point, the microcavity and tapered fiber form a single integrated structure, greatly improving the mechanical robustness of the coupling. Finally, the aluminum bracket was placed in an aluminum box (microcavity enclosure), with the pigtails at both ends of the tapered fiber extending out from the sides. The entire aluminum box undergoes independent second-stage temperature control. A precision PID servo controller ensured that the internal air temperature of the aluminum box remained stable with an extremely low fluctuation range.

This microcavity enclosure has not undergone airtight encapsulation, allowing ambient CO_2 to diffuse into the enclosure through the fiber feedthrough openings. To improve the long-term operating time of the sensor, desiccant particles with a nanoporous surface structure are placed inside the microcavity enclosure to absorb water vapor from the external environment, while exhibiting negligible adsorption toward CO_2 under the operating conditions. Therefore, it can maintain long-term dryness inside the microcavity enclosure, reducing the rate of decrease in the Q factor of the microsphere WGM microcavity.

To evaluate the influence of slow thermal fluctuations in the external environment on signal amplitude, measurements were performed on 400 ppm CO_2-N_2 mixtures in a sealed chamber under different temperature conditions. The chamber temperature was controlled using an external heating tape coupled with a PID temperature controller for precise regulation. In the experiment, the temperature was stepped in 1°C increments, and the system was allowed to stabilize for 5 min each setpoint before acquiring the raw optical mode signals. Each measurement was averaged over 200 acquisitions to improve the signal-to-noise ratio. Supplementary Fig. S11 shows the raw mode signals obtained over the 27-42°C range. Because the packaged sensor device contains a dual-stage temperature control system, it can effectively resist temperature changes in the external environment. As shown in Fig. S11, it indicates that the signals remain highly consistent across all temperatures, with amplitude deviations within 1%, demonstrating

that slow environmental temperature changes have minimal impact on the mode signal amplitude and confirming the system's stability under external thermal perturbations.

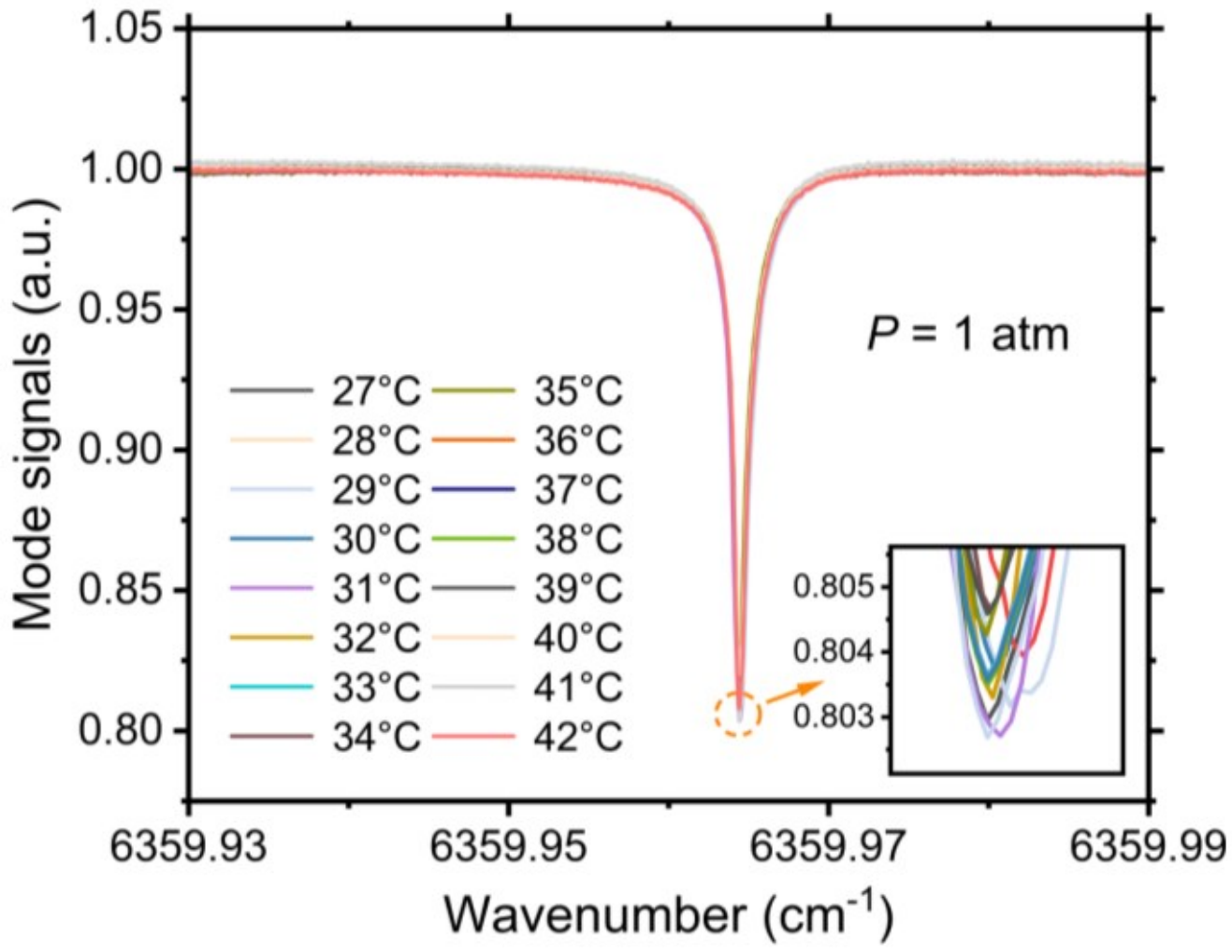

Fig. S11 | Raw optical mode signals for 400 ppm CO_2-N_2 measured over 27-42°C.

Supplementary Note 5

5.1 Detection and Analysis of CH_4 Gas Concentration

To further demonstrate the generality of the developed system, we also performed CH_4 sensing experiments using a DFB laser at 1.651 μm (TR-1651-DFB-B, AA240927410001), achieving similarly high sensitivity at the ppt level and exhibiting superior detection sensitivity compared with CO_2, as shown in Fig. S12. The line intensity of the absorption spectrum of CO_2 we used in the 1572.3 nm band is 1.761×10^{-23} cm^{-1}/molecule.cm^{-2}, while the line intensity of the 1651 nm band used to measure CH_4 is 1.520×10^{-21} cm^{-1}/molecule.cm^{-2}. The line strength of CH_4 is approximately 86 times greater than that of CO_2. The experiment shows that our limit of detection for CH_4 was determined to be 7.2 ppt, while the previous limit of detection for CO_2 was about 168 ppt. Through analysis, it can be seen that as the detection band line strength increases, the sensor has a better limit of detection.

The following is the detailed process of CH_4 detection and analysis:

To assess the system calibration of the dissipative WGM gas sensor for CH_4 detection, we tested various CH_4/N_2 mixtures with concentrations ranging from 1 ppb

to 20 ppm. The mixtures were dynamically generated by diluting a certified 1% CH_4 gas standard (in N_2) using a commercial gas mixing system. All measurements were performed at 1 atm and 296 K to ensure consistent experimental conditions. For each concentration, 20 independent signal sets were acquired within 7 minutes. Each set was the average of 200 repeated scans, improving the signal-to-noise ratio (SNR) and minimizing random errors to ensure reproducibility.

Figs. S12a and S12b show the raw optical mode signals and 2*f* signals measured at different CH_4 concentrations. After system calibration of the dissipative WGM gas sensor for detecting CH_4, 8000 s time series measurements were taken for the raw optical mode and 2*f* signal. Allan deviation analysis is similar to the analysis of CO_2. As shown in Figs. S12c and S12d, we employed the Allan deviation plot to analyze the system's noise and drift behavior over extended measurement times. At short integration time within 600 s, the Allan deviation decreases as the integration time increases with a fitted slope of approximately -0.5, indicating that white noise dominates, which mainly emanates from relative intensity noise of the probe laser and electronic noise of the electrical circuits. At longer time over 600 s, the Allan deviation decreases with fluctuations, which is a signature of system drift. Major drift sources include minor external temperature variation and light power fluctuations that vary slowly over time. In addition, to accurately estimate the minimum detection limit (MDL, i.e. LOD) of the sensor, we recalculated the Allan deviation plot by averaging the data through a 100-point window smoothing. As shown in Fig. S12, an Allan deviation analysis of the raw optical mode signal in Fig. S12c indicates a $Hz^{-1/2}$ performance with a bandwidth-normalized sensitivity of 221.18 ppt*$Hz^{-1/2}$, which remains until reaching an averaging time of 600 s. At this time, the highest CH_4 sensitivity of ~21.8 ppt was achieved. Based on the characteristic of white noise, the theoretically predicted limit of detection under pure white noise at 600 s is calculated as $221.18/600^{1/2} \approx 9.03$ ppt. This theoretical value closely matches the experimentally measured and smoothed optimal detection limit of 21.8 ppt, thereby confirming the validity of the Allan deviation analysis. Similarly, an Allan deviation analysis of 2*f* signal in Fig. S12d indicates a Hz^{-}

$^{1/2}$ performance with a bandwidth-normalized sensitivity of 57.18 ppt*Hz$^{-1/2}$, which remains until reaching an averaging time of 600 s. At this time, the highest CH_4 sensitivity of ~7.2 ppt was achieved. The theoretically predicted limit of detection under pure white noise at 600 s is calculated as $57.18/600^{1/2} \approx 2.33$ ppt. This theoretical value closely matches the experimentally measured and smoothed optimal detection limit of 7.2 ppt, also confirming the validity of the Allan deviation analysis.

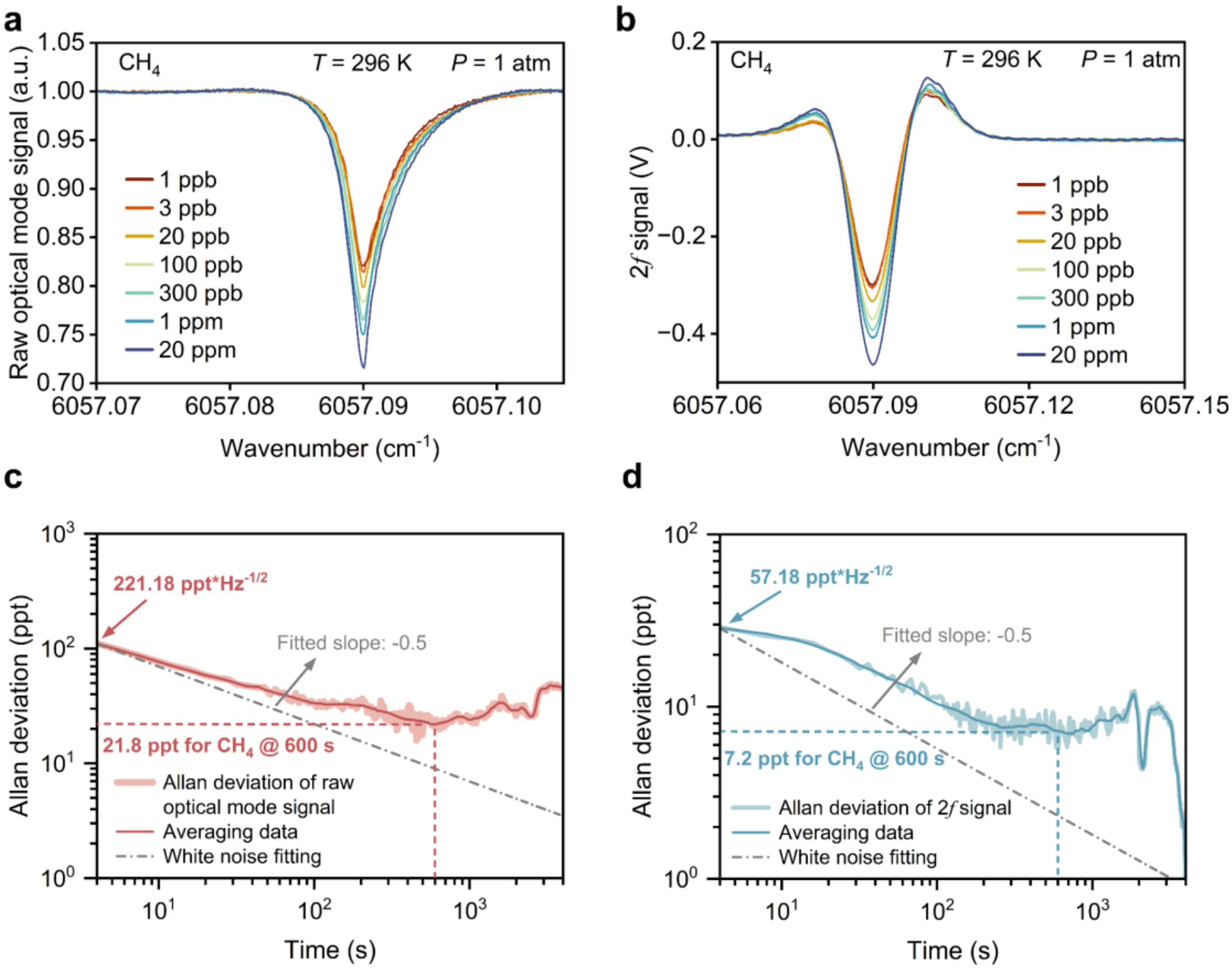

Fig. S12 | **a and b**, The raw optical mode and 2*f* signals measured at different CH_4 concentrations. **c** and **d**, Allan deviation of the dissipative WGM gas sensor.

5.2 Detection and Analysis of C_2H_2 Gas Concentration

In addition, we also conducted similar measurements and analysis on C_2H_2. We performed C_2H_2 sensing experiments using a DFB laser at 1.542 μm (DFB-1542-I-N-1-SM, 19081001), achieving high sensitivity at the ppt level and also exhibiting superior detection sensitivity compared with CO_2, as shown in Fig. S13. This is because the line intensity of the absorption spectrum of CO_2 we used in the 1572.3

nm band is 1.761×10^{-23} cm/mol, while the line intensity of the 1542 nm band used to measure C_2H_2 is 2.602×10^{-22} cm/mol. The line strength of C_2H_2 is approximately 14 times greater than that of CO_2. The experiment shows that our limit of detection for C_2H_2 is about 35 ppt, while the previous limit of detection for CO_2 was about 168 ppt. Together with the CO_2 and CH_4 results, this further confirms the consistent with the stronger absorption line strength.

The following is the detailed process of Allan deviation analysis for C_2H_2 measurement concentration:

To assess the system calibration of the dissipative WGM gas sensor for C_2H_2 detection, we tested various C_2H_2/N_2 mixtures with concentrations ranging from 1 ppb to 2 ppm. The mixtures were dynamically generated by diluting a certified 1% C_2H_2 gas standard (in N_2) using a commercial gas mixing system. All measurements were performed at 1 atm and 296 K to ensure consistent experimental conditions. For each concentration, 20 independent signal sets were acquired within 7 minutes. Each set was the average of 200 repeated scans, improving the signal-to-noise ratio (SNR) and minimizing random errors to ensure reproducibility.

Figs. S13a and S13b show the raw optical mode signals and $2f$ signals measured at different C_2H_2 concentrations. After system calibration of the dissipative WGM gas sensor for detecting C_2H_2, 8000 s time series measurements were taken for the raw optical mode and $2f$ signal. As shown in Figs. S13c and S13d, we employed the Allan deviation plot to analyze the system's noise and drift behavior over extended measurement times. At short integration time within 600 s, the Allan deviation decreases as the integration time increases with a fitted slope of approximately -0.5, indicating that white noise dominates, which mainly emanates from relative intensity noise of the probe laser and electronic noise of the electrical circuits. At longer time over 600 s, the Allan deviation decreases with fluctuations, which is a signature of system drift. Major drift sources include minor external temperature variation and light power fluctuations that vary slowly over time. In addition, to accurately estimate the minimum detection limit (MDL, i.e. LOD) of the sensor, we recalculated the Allan

deviation plot by averaging the data through a 150-point window smoothing. As shown in Fig. S13, an Allan deviation analysis of the raw optical mode signal in Fig. S13c indicates a $Hz^{-1/2}$ performance with a bandwidth-normalized sensitivity of 1.875 ppb*$Hz^{-1/2}$, which remains until reaching an averaging time of 600 s. At this time, the highest C_2H_2 sensitivity of ~109 ppt was achieved. Based on the characteristic of white noise, the theoretically predicted limit of detection under pure white noise at 600 s is calculated as $1875/600^{1/2} \approx 76.55$ ppt. This theoretical value closely matches the experimentally measured and smoothed optimal detection limit of 109 ppt, thereby confirming the validity of the Allan deviation analysis. Similarly, an Allan deviation analysis of 2*f* signal in Fig. S13d indicates a $Hz^{-1/2}$ performance with a bandwidth-normalized sensitivity of 554.38 ppt*$Hz^{-1/2}$, which remains until reaching an averaging time of 600 s. At this time, the highest C_2H_2 sensitivity of ~35 ppt was achieved. The theoretically predicted limit of detection under pure white noise at 600 s is calculated as $554.38/600^{1/2} \approx 22.63$ ppt. This theoretical value closely matches the experimentally measured and smoothed optimal detection limit of 35 ppt, also confirming the validity of the Allan deviation analysis.

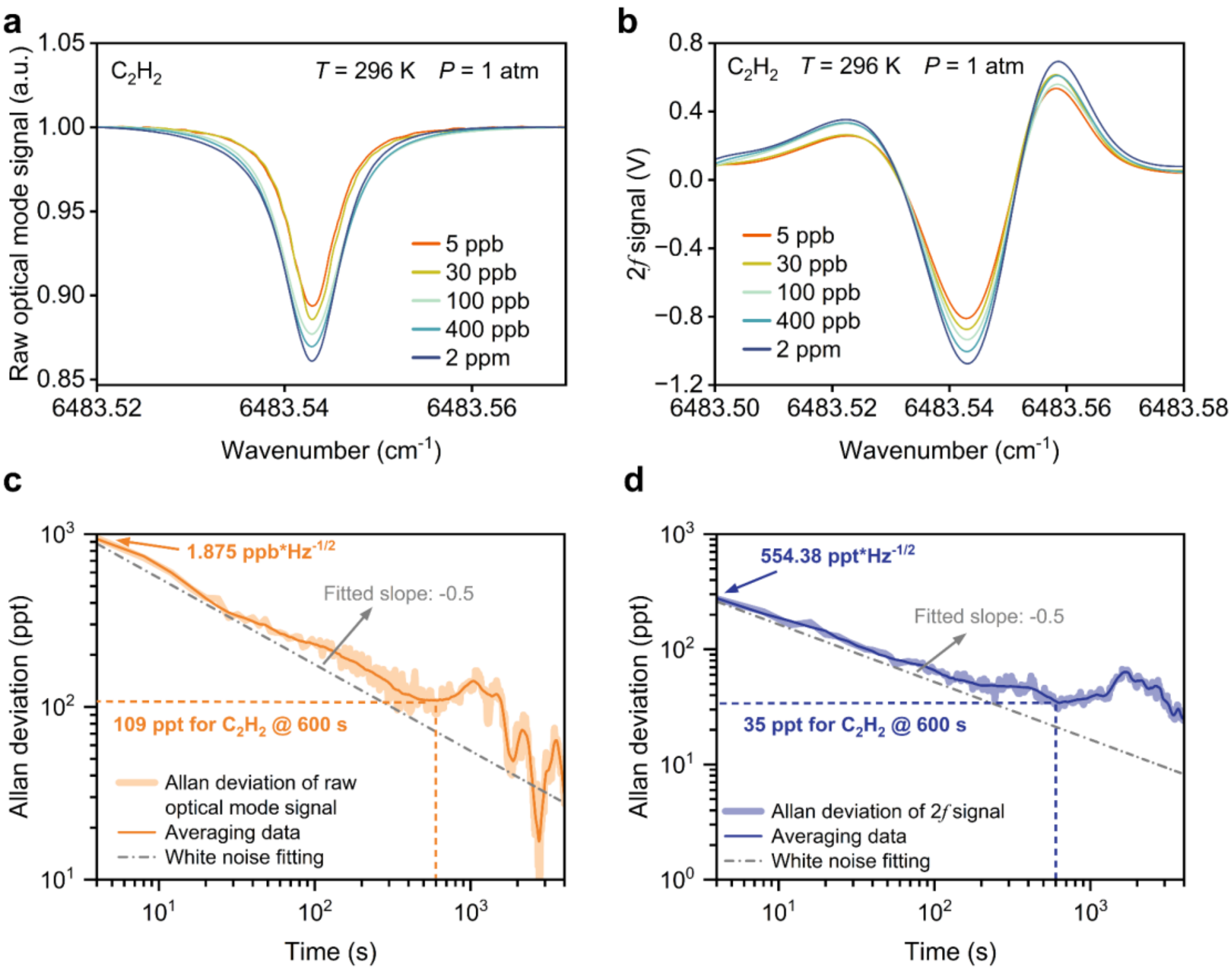

Fig. S13 | a and b, The raw optical mode and 2*f* signals measured at different C_2H_2 concentrations. **c** and **d**, Allan deviation of the dissipative WGM gas sensor.

Supplementary Note 6

Quantitative Analysis of Frequency Fluctuation Effects

The influence of laser-cavity frequency drift on the measurement uncertainty in the presented dissipative sensing scheme is analyzed quantitatively based on the system's characteristic parameters. The ultra-narrow linewidth of the optical mode of the WGM microcavity, typically ~0.001 cm^{-1}, is contrasted with the pressure-broadened linewidth of the target CO_2 absorption line, which is approximately 0.1 cm^{-1} (FWHM) under ambient conditions, as depicted in Fig.1d in the main text and Fig. S14. This two-orders-of-magnitude difference implies that the absorption cross-section varies only minimally across the scale of the linewidth of the optical mode.

Frequency stability characterization indicates that the locked cavity system exhibits a resonance position drift within a range of approximately ±0.003 cm^{-1} within

10 minutes. The drift magnitude (±0.003 cm^{-1}) remains much smaller than the absorption linewidth (~0.1 cm^{-1}), ensuring that the mode remains within the high-slope region of the absorption profile. The *2f* signal is extracted via synchronous demodulation at the modulation frequency. This method renders the measurement inherently insensitive to absolute frequency offsets, as it detects the periodic depth modulation synchronized with the scan, not the static resonance wavelength.

The key quantitative consideration is that the frequency drift (±0.003 cm^{-1}) is still near the absorption peak. Therefore, during each scanning cycle, drift does not shift the resonance outside the effective absorption linewidth, thus maintaining the line shape and amplitude of the signal. The dominant noise floor is thus determined by the intensity noise and the efficiency of the demodulation process, not by laser-cavity detuning variations. This analysis confirms that the designed sensing approach effectively reduces the sensitivity to low-frequency technical noise, including long-term cavity frequency fluctuations.

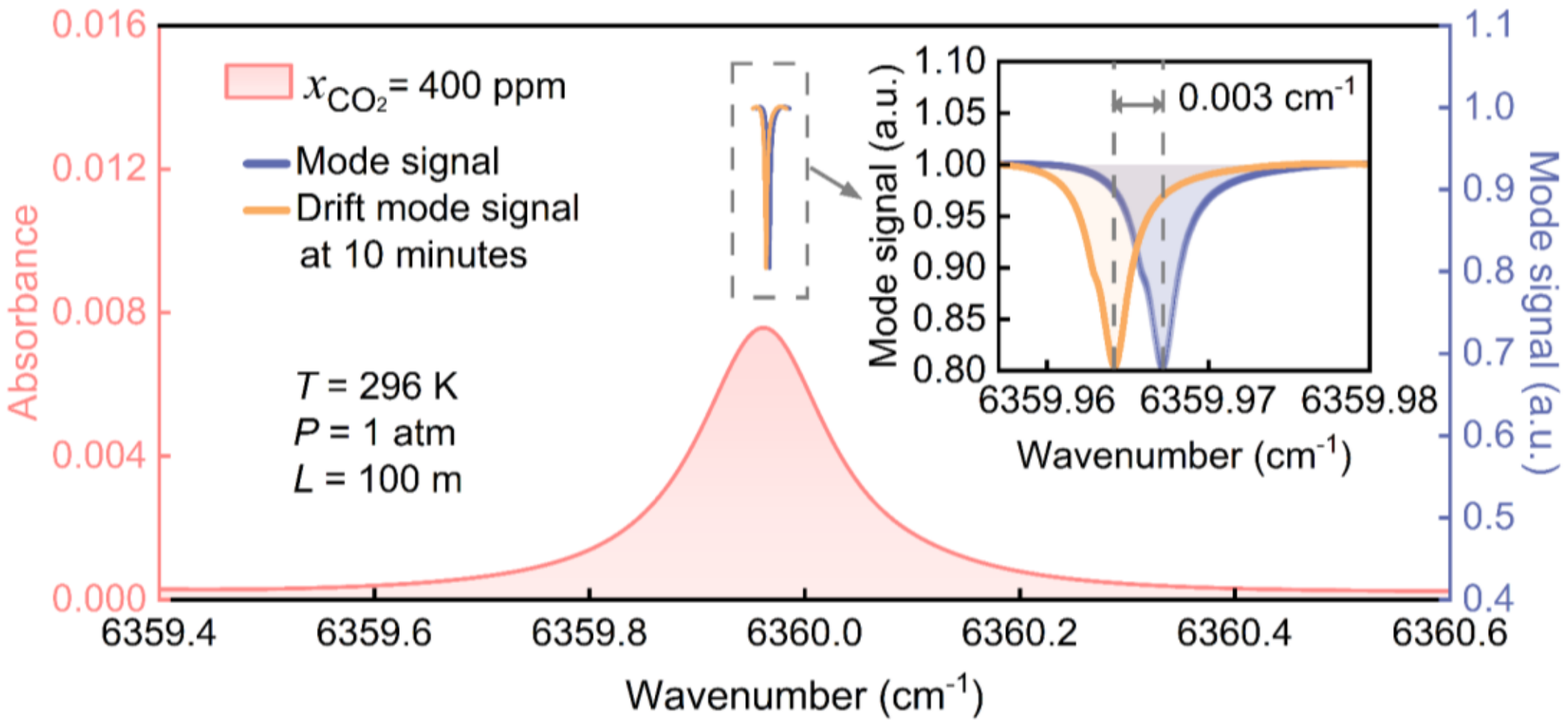

Fig. S14 | CO_2 absorption line and one resonance position of the microsphere cavity. Inserted graph: Frequency drift of resonance mode.

Supplementary Note 7

Gas Selectivity of the Dissipative WGM Sensor

The selective detection of a target gas within complex mixtures is a fundamental requirement for practical sensing. This supplementary section provides a detailed experimental demonstration and theoretical rationale for the intrinsic gas selectivity of

our spectroscopic dissipative sensing platform, which operates on a principle distinct from functionalized coating-based sensors.

7.1 Principle of Spectroscopic Selectivity

The core mechanism enabling selectivity in this work is wavelength-specific optical absorption by gas molecules, a fundamental molecular property. The sensor employs a high-Q WGM microcavity as a sensitive probe for optical absorption by gas. A tunable diode laser is precisely tuned to a wavelength corresponding to a characteristic, narrow-linewidth electronic or vibrational absorption line of a specific target gas (e.g., CO_2 at 1.572 μm, CH_4 at 1.651 μm, C_2H_2 at 1.54 μm). When the laser wavelength spectrally overlaps with the target gas absorption feature, molecules present in the evanescent field of the optical mode absorb photons. Our approach leverages enhanced local optical fields and thermal effects at the coupling region to convert weak absorption into measurable variations in resonance depth. Crucially, gases lacking absorption features at the specific laser wavelength do not contribute to this resonant absorption change, thereby conferring inherent selectivity directly derived from molecular spectroscopy.

7.2 Experimental Validation via Controlled Cross-Sensitivity Tests

To rigorously validate this principle and rule out significant cross-interference, a series of controlled experiments was conducted using gas mixtures. An uncoated microsphere resonator was housed in a sealed gas cell with precise temperature and pressure control. Three distributed feedback (DFB) diode lasers were employed, each centered at the absorption line of a different target gas: CO_2, CH_4, and C_2H_2. Certified standard gases and a computerized multi-channel gas mixing system (Environics Series 4000) were used to generate mixtures with accurately defined concentrations, with high-purity N_2 serving as the balance gas.

7.2.1 Selective Response to Target Gas Variation

For each target gas, the corresponding laser was activated and locked to its

absorption line. The concentration of the target gas was systematically varied (e.g., CO_2: 25, 50, 200, 400 ppm), while the concentrations of the other two potential interferent gases were maintained at constant, non-zero levels. As shown in Supplementary Fig. S15a-c, a strong, monotonic dependence of the resonance depth change of the raw optical mode signal on the target gas concentration was observed for each laser-gas pair. This demonstrates the sensor's primary sensitivity to the intended analyte.

7.2.2 Insensitivity to Non-Target Gas Variation (Cross-Interference Test)

The converse experiment was critical for proving selectivity. For a given laser (e.g., the CO_2-absorbing laser at 1.572 μm), the concentration of its target gas (CO_2) was held constant. Subsequently, the concentrations of the other gases (CH_4 and C_2H_2) were independently varied over a comparable concentration range. The results, summarized in Supplementary Fig. S15d-f, show no significant change in the measured resonance depth of the raw optical mode signal. The observed fluctuations remained within the baseline noise level of the sensor system. This confirms that the presence and varying concentration of gases not absorbing at the specific wavelength do not elicit a measurable response.

7.3 Discussion and Conclusion

The combined dataset from these controlled mixture experiments provides strong experimental evidence for the spectroscopic selectivity of the dissipative sensing mechanism. The sensor's response function is primarily governed by the Beer-Lambert law interaction between the spectrally narrow probe laser and the specific gas species possessing a resonant absorption at that wavelength. This approach fundamentally differs from polymer-coated WGM sensors, which rely on the partitioning of analytes into a functionalized film based on chemical affinity.

The real-world field demonstration of CO_2 monitoring, showing plausible diurnal variations, gains robust contextual support from these laboratory selectivity tests. While atmospheric diurnal patterns are not uniquely specific, the conjunction of field data with known anthropogenic drivers, and more definitively, the laboratory-proven absence of

response to common potential interferents like CH_4 under the CO_2-measurement configuration, strongly supports the interpretation that the recorded signal mainly originates from CO_2 concentration changes.

In summary, the selectivity of this sensor platform is intrinsically rooted in the discrete absorption spectra of gas molecules. This method offers a powerful and complementary alternative to material-based selective coatings, particularly advantageous for applications requiring the detection of small molecules with well-defined, narrow absorption features in the near-infrared region.

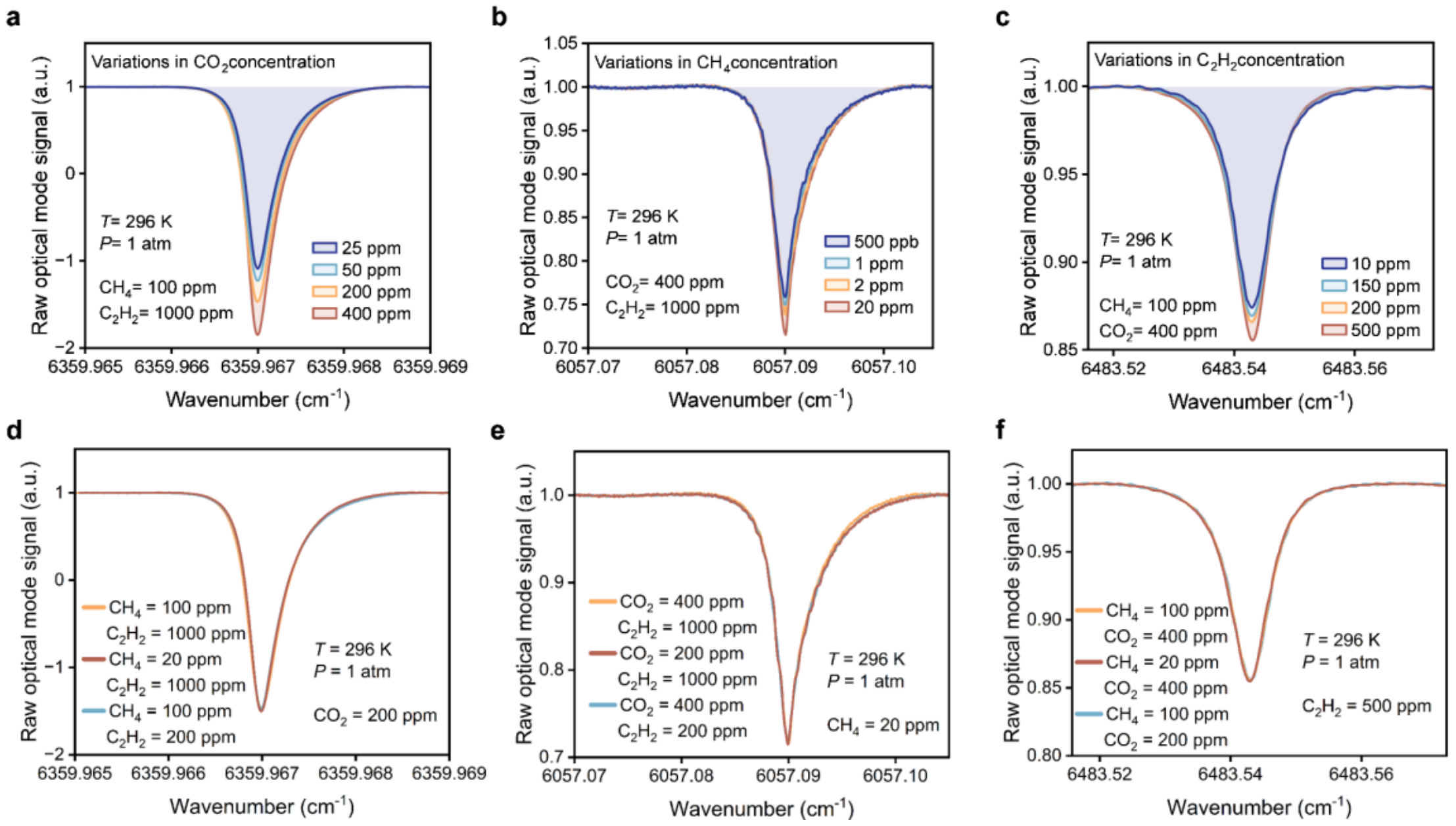

Fig. S15 | Verification experiment of gas selectivity. a, Variation of the raw optical mode signal of CO_2 as a function of CO_2 concentration measured in CO_2–C_2H_2–CH_4 gas mixtures with fixed C_2H_2 and CH_4 concentrations. **b**, Variation of the raw optical mode signal of CH_4 as a function of CH_4 concentration measured in CO_2–C_2H_2–CH_4 gas mixtures with fixed C_2H_2 and CO_2 concentrations. **c**, Variation of the raw optical mode signal of C_2H_2 as a function of C_2H_2 concentration measured in CO_2–C_2H_2–CH_4 gas mixtures with fixed CH_4 and CO_2 concentrations. **d**, Response of the raw optical mode signals of CO_2 to varying concentrations of CH_4 and C_2H_2, respectively, under a constant CO_2 concentration. **e**, Response of the raw optical mode signals of CH_4 to varying concentrations of CO_2 and C_2H_2, respectively, under a constant CH_4 concentration. **f**, Response of the raw optical mode signals of C_2H_2 to varying

concentrations of CH_4 and CO_2, respectively, under a constant C_2H_2 concentration.

Supplementary Note 8

Path Towards System Miniaturization and Integration

The experimental system described in the main text was assembled using discrete commercial bench-top instruments mounted on a mobile laboratory cart. This configuration was intentionally adopted to provide a flexible and well-controlled platform for proof-of-principle validation and rigorous performance benchmarking of the dissipative sensing mechanism. The use of high-performance commercial instruments ensures reliable operation and enables accurate evaluation of the intrinsic performance limits of the proposed approach. Therefore, the cart-based configuration should be regarded as a verification platform rather than the final integrated implementation of the sensor.

Importantly, the sensing principle demonstrated in this work does not rely on bulky instrumentation. The essential functional modules can be consolidated through circuit-level integration and optical packaging. Based on this consideration, we have carried out system integration at the levels of microcavity packaging, electronic consolidation, and overall system assembly, establishing a practical pathway toward miniaturization and portability.

At the optical level, the standalone WGM microcavity has been packaged into a mechanically robust and miniaturized module. The packaging provides stable structural support and reduces susceptibility to external mechanical perturbations, thereby maintaining a stable coupling condition. Temperature regulation and humidity control are incorporated to ensure long-term resonance stability and measurement repeatability. Compared with the optical platform used in the initial experiments, the packaged microcavity module exhibits a significantly reduced footprint. Detailed structural information is provided in **Supplementary Note 4**.

At the electronic level, the functionalities previously provided by separate commercial instruments-including the laser current and temperature controller, function

generator, lock-in amplifier, photodetector, and data acquisition card-have been consolidated onto a custom-designed integrated circuit board. This board-level system combines analog and digital circuitry to implement laser driving and frequency modulation, signal amplification and conditioning, synchronous demodulation, and data acquisition. The integrated electronic module substantially reduces system size and complexity while preserving the core measurement capability. A photograph of the integrated electronic system is shown in Supplementary **Fig. S16**.

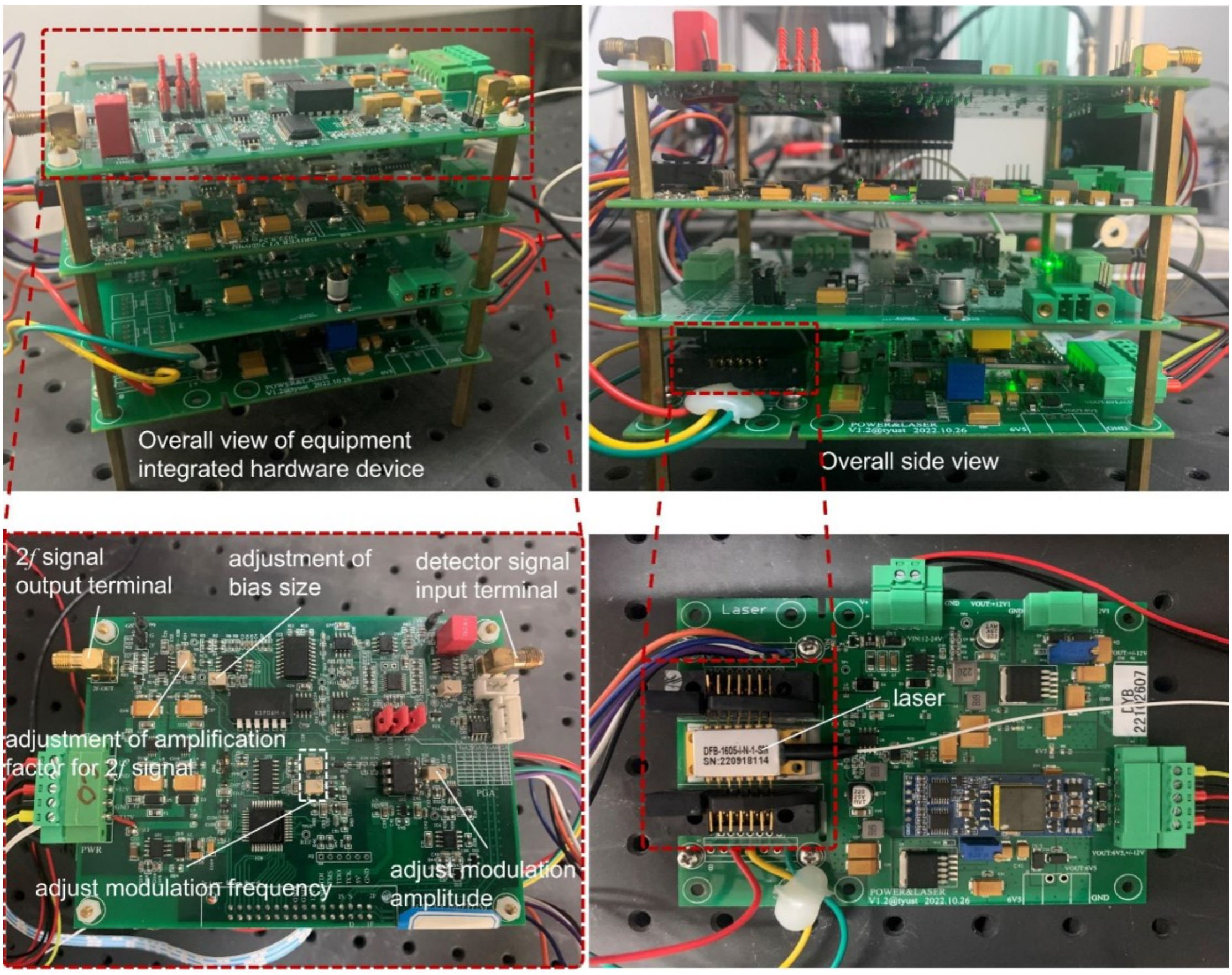

Fig. S16 | Integrated electronic system component.

At the system level, the packaged microcavity module and the integrated electronic module have been assembled into a complete prototype housed within a compact enclosure measuring 26 cm×16 cm, as illustrated in Supplementary Fig. S17. The integrated system performs frequency modulation of the probe laser and synchronous demodulation to extract the 2*f* signal, enabling real-time data acquisition

and processing. Using this miniaturized configuration, we successfully acquired the $2f$ signal corresponding to atmospheric CO_2 absorption modes, demonstrating stable sensing performance under the integrated architecture.

These results demonstrate that the cart-based configuration presented in the main text was primarily intended for methodological validation and performance benchmarking. The successful realization of the integrated prototype confirms that the proposed sensing approach is fully compatible with circuit-level consolidation and compact system packaging. No fundamental technical barrier limits system miniaturization, and the demonstrated prototype establishes a practical pathway toward portable and field-deployable implementations.

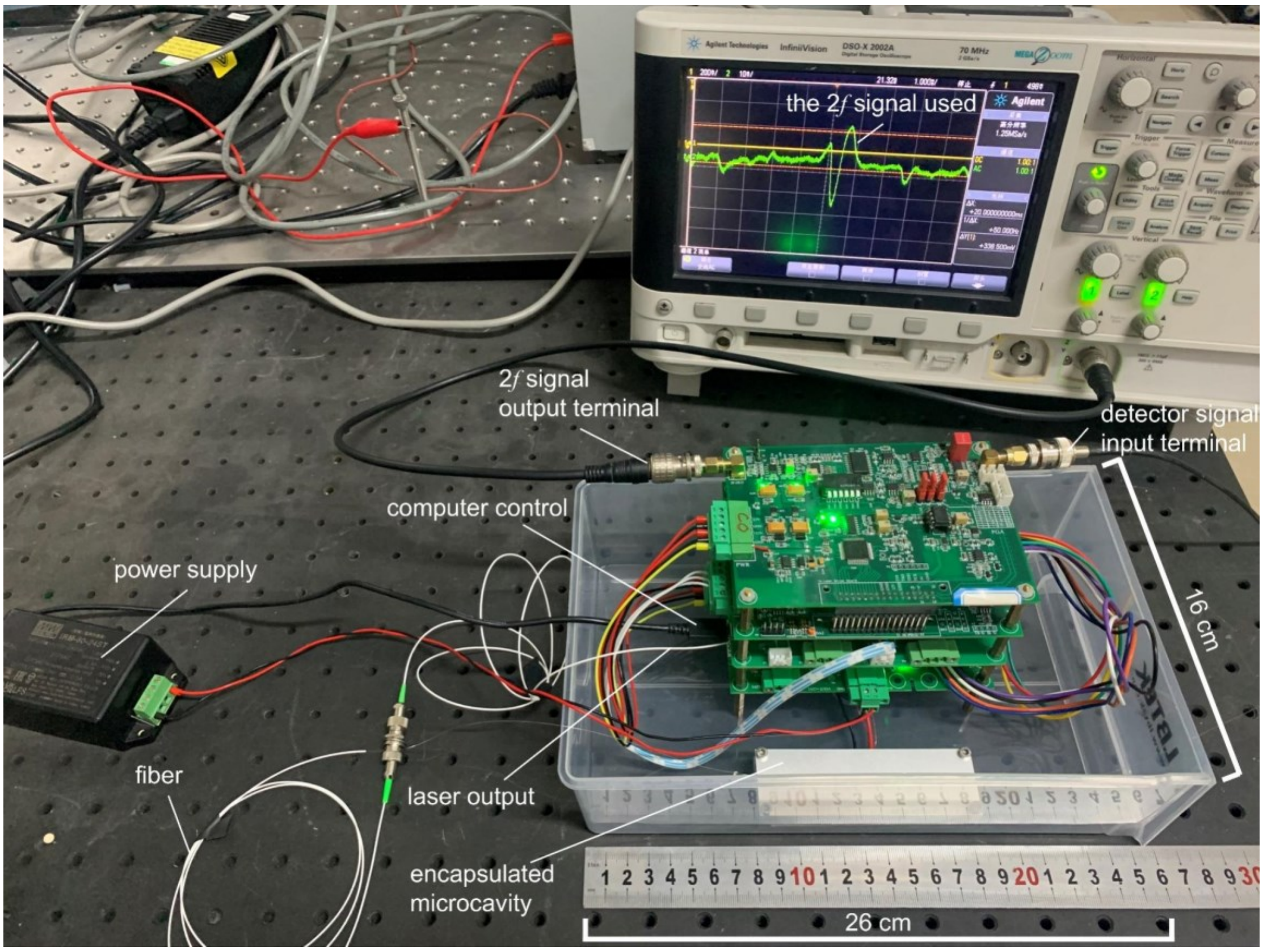

Fig. S17 | Integrated sensor system.

Supplementary Note 9

Considerations on Experimental Matrix and Environmental Robustness

This section provides a detailed rationale for the experimental methodologies

employed in the sensor characterization and discusses critical investigations into environmental interferents, specifically atmospheric humidity. The aim is to clarify fundamental operating principles and to validate the sensor's potential for operation under realistic conditions.

9.1 Reasons for using nitrogen matrix

In this study, high-purity N_2 was employed as the background gas during the initial calibration and fundamental selectivity evaluation stages. This methodology is consistent with established practices in high-precision absorption spectroscopy and microcavity sensing. In related research, inert, dry, and spectroscopically “quiet” N_2 is routinely used to establish system baselines under interference-free conditions. This approach excludes non-target influences and enables quantitative characterization of the intrinsic sensor response to target analytes. This strategy facilitates the effective isolation of molecule-specific absorption contributions, allowing accurate determination of key performance indicators such as sensitivity and detection limit, which are central objectives of the preliminary phase of this investigation.

It should be noted that for conventional absorption spectroscopy techniques employing direct absorption spectral signals as the sensing metric, variations in background gas composition can indeed affect absorption line profiles owing to differences in collisional broadening coefficients among molecular species. This, to a certain extent, constrains the direct transferability of calibration results obtained with N_2 dilution to ambient atmospheric conditions. However, the sensing mechanism adopted in the present work does not rely on precise fitting of absorption line shape parameters; instead, it leverages changes in microcavity resonance modes induced by heat deposition resulting from gas absorption as the core transduction principle. The magnitude of this thermal effect is governed primarily by the absolute number density of target molecules and their absorption cross-section, and is largely independent of non-absorbing background species present in the system.

Consequently, within this dissipative whispering-gallery-mode (WGM) sensing framework, the calibration relationship established using high-purity N_2 as the diluent and calibration gas exhibits good transferability and can therefore be applied to CO_2 concentration measurements under realistic atmospheric conditions.

9.2 Experimental Investigation of Humidity Effects and Surface Phenomena

In contrast to dry gas composition, water vapor presents a significant practical challenge due to its ubiquitous presence and potential for both measurement accuracy and physical perturbation of the microcavity. We conducted a dedicated study to analyze the physical effect of humidity on the WGM microcavity itself.

A silica microsphere was placed in a climate-controlled chamber. As the relative humidity (RH) was varied from 30% to 50% at constant temperature, the WGM resonance exhibited a consistent redshift on the order of several picometers (corresponding to a wavenumber shift of ~±0.16 cm^{-1} for a ±10% RH change), accompanied by minor fluctuations in resonance depth (Supplementary Fig. S18). This shift is attributed to water molecule adsorption altering the effective refractive index in the evanescent field region.

The observed humidity-induced wavenumber shift (±0.16 cm^{-1}) is notably lower than theoretical predictions (often on the order of ±1.6 cm^{-1} or tens of pm/%RH) for a standard hydrophilic silica surface. This discrepancy indicates a fundamental modification of the microsphere surface properties. The key factor is identified as the high-temperature electrofusion process used in microsphere fabrication, which induces two synergistic effects:

Surface Chemical Modification: At extreme temperatures, adjacent surface silanol (Si-OH) groups undergo irreversible dehydration condensation, forming hydrophobic siloxane (Si-O-Si) bridges. This drastically reduces the density of hydrophilic sites available for hydrogen-bonded water adsorption.

Surface Structural Densification: In the molten state, surface atom migration driven by surface tension leads to smoothing and densification of the silica network, minimizing physical defects and pores that could trap water.

Furthermore, during optical characterization, resonant power buildup within the cavity can generate a localized photothermal effect. This “thermal drying” of the immediate surface region can dynamically counteract adsorption, potentially leading to an observed sensitivity lower than the intrinsic material property under equilibrium

conditions.

9.3 Mitigation Strategy: Sensor Packaging

Although the inherent surface hydrophobicity reduces the impact on humidity changes, humidity-induced resonance drift remains a non-negligible noise source for high-precision gas sensing, affecting baseline stability and long-term reliability. To address this, the coupling structure consisting of the tapered fiber and the microsphere WGM microcavity is packaged (detailed in **Supplementary Note 4**). This packaging achieves two critical functions:

This device maintains a constant, dry internal environment around the micro resonator, effectively decoupling it from external humidity fluctuations and particulate contamination, thereby ensuring stability. It allows controlled introduction of analyte gases through a dedicated sampling port. The incoming gas stream passes through desiccant particles before entering the sensing chamber, ensuring a stable and dry local environment for accurate measurements.

Under this packaging configuration, the mode drift was measured while the external relative humidity changed from 30% to 50%. As shown in Fig. S18, under the condition of relative humidity fluctuation of ±10% RH, the resonance wave number shift of the WGM was only about ±0.01 cm^{-1}, and the mode remained within the effective absorption linewidth, which did not affect the measurement of CO_2.

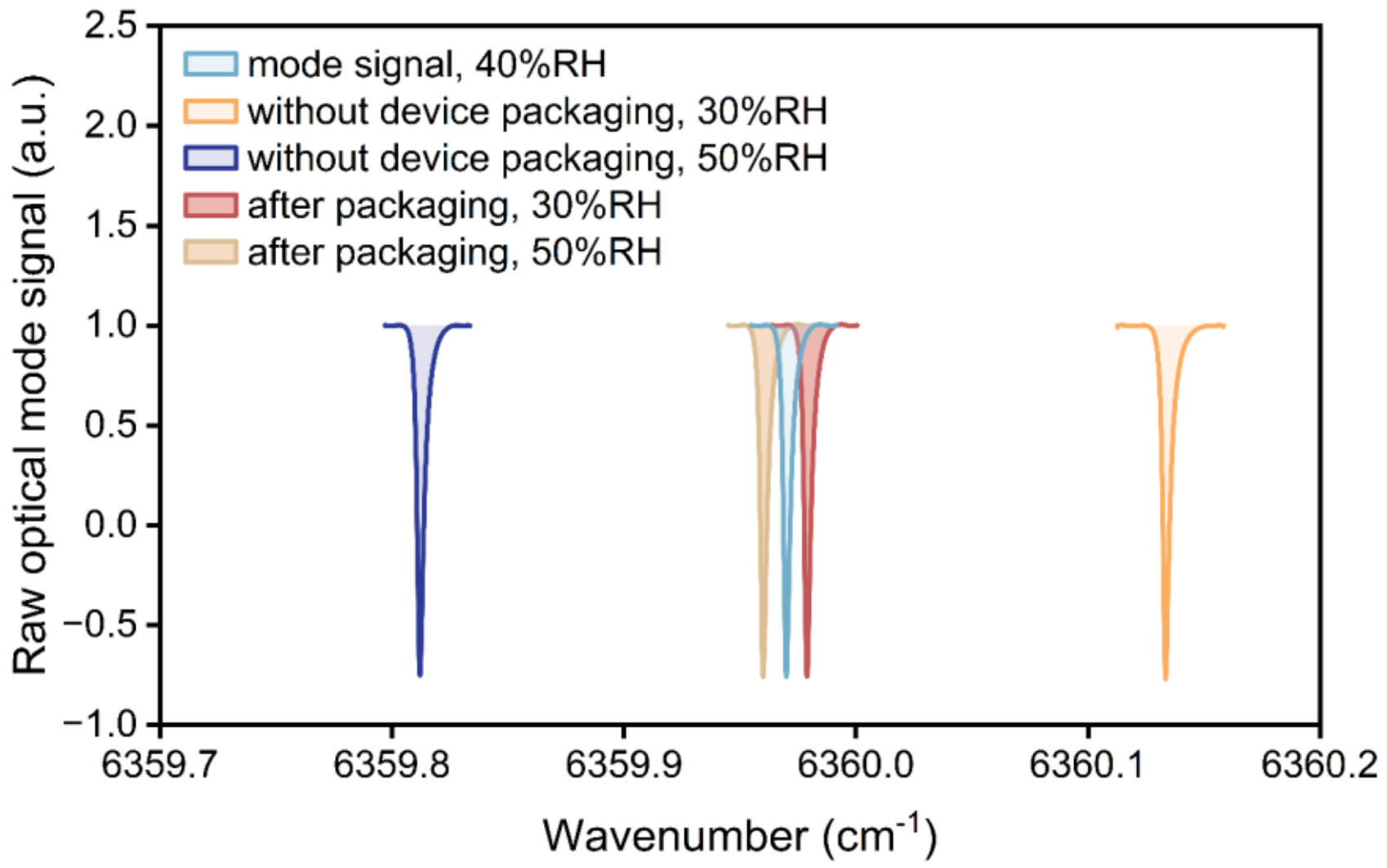

Fig. S18 | Variations of raw optical mode signal under different relative humidity.

Supplementary Note 10

Extended 50-Day Long-Term Stability Assessment

To rigorously assess the operational longevity and intrinsic drift characteristics of the WGM sensor, a dedicated 50-day continuous stability experiment for ambient air enclosed in a sealed chamber was conducted. As shown in Fig. S19, the sensor exhibited exceptional stability during the 50 days. The resonance linewidth ($\Delta\lambda$) fluctuated within ±1.5% of its initial value, indicating a stable Q factor of the microcavity. The resonance depth showed fluctuation of less than 1%, confirming a stable coupling condition between the fiber taper and the microsphere.

However, the coupling systems for the tapered fiber and the microsphere have inherent mechanical stress relaxation. Therefore, in the future, the depth and linewidth of the mode may change on a longer-term scale, and if such changes occur, recalibration will be necessary.

The mounting scheme, which relies on a UV-adhesive-fixed tapered fiber and a silica stem supporting the microsphere, is subject to long-term viscoelastic effects. Over periods of months, nano-scale creep and stress relaxation within the adhesive and the silica structure can occur. This will slowly change the relative position of the tapered fiber and the microsphere, thus altering the coupling strength. As a result, may alter the coupling strength, affecting the resonance depth and linewidth.

Based on the fluctuations in the mode signal amplitude measured over the aforementioned 50-day period, the results indicate that the microspheres have successfully completed the aging test, and it can be predicted that their performance will not exhibit significant changes over an extended period of future use. During prolonged operation, if notable variations in mode width or significant deviations in concentration measurements are observed, the system may be recalibrated or the microspheres replaced.

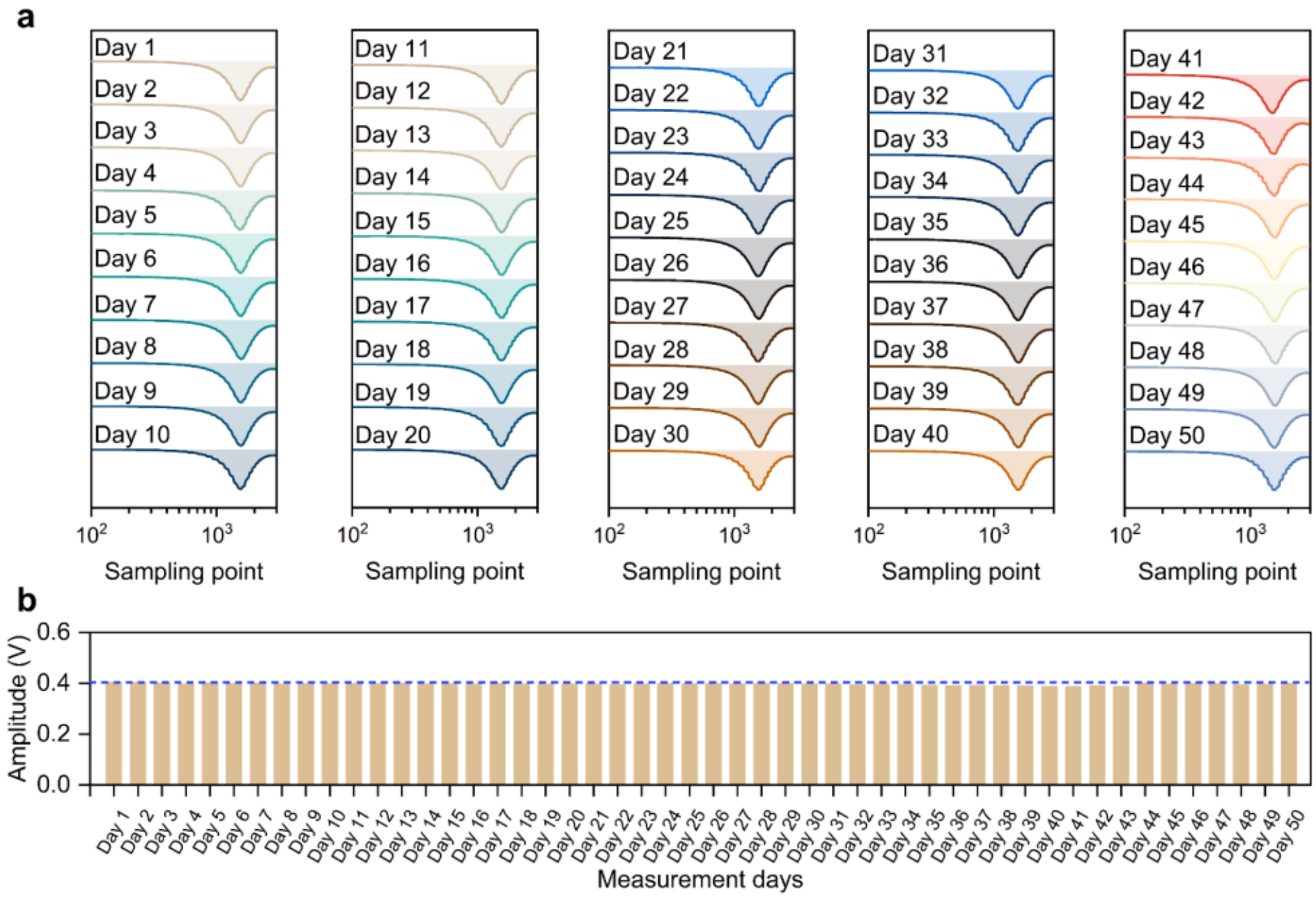

Fig. S19 | **a**, Mode signals measured within 50 days. **b**, Analysis of amplitude of mode signal.
