# Supplementary material for: Sub-parts-per-billion CO2 Detection based on Dissipative Whispering Gallery Mode Microcavity Sensor
Source: Nat Commun. 2026 Jul 4;17:8341. doi: 10.1038/s41467-026-75218-y (PMC13473595; doi:10.1038/s41467-026-75218-y)
Supplement: Supplementary file 2 [file 41467_2026_75218_MOESM2_ESM.pdf]

nature portfolio

Peer Review File

Sub-parts-per-billion CO_2 Detection based on Dissipative Whispering Gallery Mode Microcavity Sensor

Corresponding Author: Professor Tingdong Cai

This file contains all reviewer reports in order by version, followed by all author rebuttals in order by version.

Version 0:

Reviewer comments:

Reviewer #1

(Remarks to the Author)
The manuscript reports an impressive and potentially field-shaping result: a microcavity-based gas sensor achieving sub-ppb detection limits with excellent stability. The work is original, and the demonstrated performance could indeed be relevant for practical applications. However, the theoretical analysis of the sensing mechanism is insufficient, lacks appropriate references, and contains conceptual gaps that undermine the credibility of the interpretation. On the experimental side, while the data are compelling, several performance claims require moderation or further justification. In its current form, the manuscript requires substantial revision before it can be considered for publication in a high-profile journal such as Nature Communications.

Scientific Assessment
1. Theoretical description
Although the experimental data clearly show that CO_2 concentration variations lead to measurable signals at sub-ppm levels, the manuscript does not provide a satisfactory physical explanation of this sensitivity. Several key elements of the analysis are missing:
- A schematic illustration of the resonator–taper configuration is essential. It should clearly indicate the mode structure, the length of the coupling region l, and the spatial extent of the heated region.
- The authors should model absorption-induced heating and estimate the local temperature rise around the coupling region. For example: Can the temperature increase plausibly reach ~0.1 K, and what concentration change is needed for such an increase? Over what spatial region is this heating distributed?
- Using this temperature information, the authors should apply the equations provided in the Supplementary Information to show whether changes in the coupling length can realistically account for the measured signal amplitude.
- If this mechanism is valid, the authors need to show how thermal expansion or refractive-index modifications lead to the observed increase in effective coupling length. The schematic mentioned above is important for conveying this clearly.
- Finally, the theoretical framework should be used to explain why the sensor response follows a logarithmic (not exponential!) dependence on concentration. This is currently unaddressed.
Without these clarifications, the theoretical interpretation remains unconvincing.

2. Experimental Considerations and Data Interpretation
Several issues in the data presentation and performance evaluation should be addressed:
- While the Allan deviation demonstrates the detection limit, the precision varies strongly across the concentration range. What is the precision around 400 ppm? Figures 4 and 5a should be enlarged or supplemented with zoomed-in plots to show the actual scatter.
- Figures 4c and 5c are not meaningful, as the same data used for calibration is plotted again, making a linear response inevitable.
- The direct-absorption and 2f-WMS methods differ by ~10 ppm near 400 ppm. What is the resulting accuracy after calibration? An independent reference measurement would be very helpful.
- Figure 6c contains an incorrect legend and should be corrected.
- The presented traces appear overly smooth. The authors should show raw, unprocessed resonance data, including the noise (e.g., Figs. 3 and 6d,e).
- The laser linewidth used for interrogation should be specified.
Outdoor measurements:

- The field measurement data lack significance without parallel measurements using a calibrated high-end instrument.
- The authors should comment on the influence of humidity. High-Q microcavities are known to be sensitive to adsorbed water, which can change the resonance.
- What is the basis for reporting LoDs at 424 seconds? Moreover, you cannot take a minimum of a noisy Allan plot and claim it is your LoD (0.12 ppb at 424 s of averaging. Will it become 0.5 ppb at 500 seconds??).

3. Practical and Engineering Considerations
To support the claims of robustness and long-term stability, the following aspects should be clarified:
- A clear depiction of the mechanical assembly—how the cavity and tapered fibre are mounted—is necessary. The current illustrations do not convey the stability mechanism.
- How challenging is it to identify or fabricate a cavity whose resonance aligns with the chosen absorption line? Can this be tuned, and if so, by how much?
- More detail on the gas-mixing system is needed. What system was used, and how accurately can concentrations at the 1.5 ppb level be generated and maintained?

4. Moderation of Claims
- Line 54: The manuscript states that frequency locking is "typical" in TDLAS. This is incorrect; frequency locking is not commonly used in standard direct or WMS TDLAS configurations and is typically employed only in advanced cavity-enhanced systems.
- Frequency fluctuations - even in this setup - will still contribute to measurement uncertainty, as the molecular absorption line is not infinitely broad compared with the microcavity resonance.
- The comparison in Table 1 is not well balanced. Techniques such as multi-pass cells or cavity-enhanced spectroscopies can readily achieve sub-ppb detection limits in the mid-IR; on-chip waveguide sensors recently reported 20 ppb. The comparison spans disparate spectral ranges, integration times, and instruments optimized for different applications, making the table's implications unclear.

Reviewer #2

(Remarks to the Author)
Ruan et al, measure gas absorption signals through variations in resonance depth of a WGM microsphere sensor. They achieve detection over a ppm range and a minimum detection limit of 0.12 ppb. Although the manuscript appears technically correct, I cannot recommend acceptance at the level of Nature Communications due to the following:

1. The concept of gas absorption causing change in resonance depth is the basis for microcavity absorption spectroscopy, which is well known (and done better by WGM based frequency combs as they have multiple spectral lines). Those systems are also more truly compact than this one as this one relies on a tapered optical fiber which will hinder true portability). See: https://opg.optica.org/oe/fulltext.cfm?uri=oe-15-20-12959 and all other WGM frequency comb gas sensing papers.
2. The concept of optimizing the dither frequency amplitude for improved signal has already been published e.g., Journal of Lightwave Technology 38, 6393–6401 (2020).
3. The authors claim that their detection limit is five orders of magnitude better than other WGM sensors which rely on resonance frequency shift tracking, however, this is exaggerated. Toroidal sensors have demonstrated low ppt sensing: (e.g., ACS Appl. Mater. Interfaces 16, 5120–5128 (2024) and ACS Appl. Mater. Interfaces 14, 42430–42440).
4. There is also no demonstration of selectivity, which is what the polymer coated WGM sensors show. I would have expected this to be tested with other gases as well. It's not clear to me how the real-world demonstration proves selective detection of CO_2. Other gases can also show diurnal fluctuations.
5. The system mounted on a laboratory cart is not, in my view, consistent with describing the platform as compact or portable.

Reviewer #3

(Remarks to the Author)
The manuscript titled "Sub-ppb CO_2 Detection based on Dissipative Whispering Gallery Mode Microcavity Sensor" presents a non-functionalized dissipative gas sensor utilizing a silica microsphere Whispering Gallery Mode (WGM) cavity, with CO_2 detection employed as a representative case to demonstrate its sensing capability. The device employs a tapered fiber to excite the resonant modes of the microcavity; by leveraging intracavity field enhancement, localized heating in the coupling region is amplified, leading to significant modulation of the coupling strength—enabling high sensitivity to variations in CO_2 concentration. Furthermore, the high Q-factor of the WGM microcavity enhances the thermal effects arising from weak gas absorption. The dissipative sensing mechanism inherently suppresses interference from laser frequency fluctuations, and when combined with modulation-demodulation techniques, effectively mitigates low-frequency intensity noise—overcoming the challenge posed by the inherently weak near-infrared absorption of CO_2. The study demonstrates that, operating in the near-infrared regime, the sensor achieves performance comparable to mid-infrared counterparts, attaining a detection limit at the parts-per-trillion (ppt) level, thereby showing strong potential for trace gas sensing applications. Despite these promising results, several critical aspects require major revisions to improve the clarity, completeness, and scientific rigor of the work:

1. The manuscript indicates that CO_2 absorption induces localized temperature changes near the microcavity, affecting both the resonance wavelength and the coupling efficiency. Given the claimed high sensitivity, it is imperative to clarify how ambient temperature stability was maintained during experiments. Specifically, was a thermostatically controlled environment used? Detailed information on temperature stabilization protocols must be provided to substantiate the

reliability of the reported measurements.

2. While the authors state that spontaneous thermal noise is negligible, the influence of real-world environmental variables remains inadequately addressed. Clarification is needed regarding: (i) the susceptibility of signal detection to external factors such as ambient light intensity and weather conditions; and (ii) the impact of ambient temperature fluctuations on key sensor metrics—including sensitivity, response/recovery dynamics, and baseline stability. A thorough discussion of these effects is essential to assess the sensor's practical deployment feasibility.

3. Although the proposed WGM-based platform is suggested to be applicable to various trace gases with near-infrared absorption features (e.g., CO_2, CH_4, NH_3, H_2S, H_2), the issue of gas selectivity—a fundamental requirement for reliable operation in complex environments—is not discussed. This omission undermines the sensor's credibility and applicability. The authors are strongly encouraged to include an evaluation of selectivity through experimental comparisons with interfering gases, computational modeling (e.g., density functional theory), or other appropriate methods.

4. The CO_2 sensing tests were conducted in a nitrogen matrix, which deviates significantly from standard characterization practices. A clear justification for this choice is required, including the scientific rationale for using pure N_2 as the carrier gas. Moreover, the sensor's compatibility with more realistic atmospheric conditions—such as ambient air containing oxygen, humidity, and other background constituents—should be explicitly evaluated and discussed to support claims of practical utility.

5. Stability assessment—encompassing both long-term performance consistency and material durability—is insufficiently addressed. First, the current testing duration is too short to reflect operational longevity required in real-world monitoring (where sensors may operate continuously for months or years). Short-term data cannot reveal degradation phenomena such as baseline drift or sensitivity loss. Second, no characterization of the sensing material's structural or chemical stability (e.g., via XRD, XPS, SEM before and after exposure) is presented, despite its direct impact on sustained performance. It is recommended that: (a) long-term stability tests be extended over a period of 1–3 months with periodic monitoring of key parameters; and (b) post-exposure material analyses be included to evaluate structural integrity and surface stability.

6. Notably absent are basic morphological characterizations of the sensing structure—such as surface topography, geometric uniformity, or dimensional precision—despite their critical role in determining optical coupling efficiency and thermal response. In microcavity-based sensors, structural features directly influence mode confinement, heat distribution, and gas interaction dynamics. Additionally, given the focus on CO_2 detection, inclusion of CO_2 adsorption-desorption isotherms would further support the interpretation of sensitivity and response mechanisms.

7. The mechanistic interpretation of CO_2 sensing lacks integrated support between textual analysis and experimental evidence. To enhance academic depth and readability, the authors should adopt the standard framework of "experimental observation → mechanistic explanation → graphical validation." Explicit correlation between measured responses and proposed physical processes—supported by schematic illustrations or comparative plots—is necessary to strengthen the logical coherence and scientific robustness of the study.

Version 1:

Reviewer comments:

Reviewer #1

(Remarks to the Author)
I appreciate the very thorough revision by the authors. They have carefully addressed my comments and reworked parts of the manuscript to improve the scientific clarity. The revision has significantly increased the quality of the paper.

However, one major inconsistency remains that should be clarified before I can recommend publication:

Figures 5e and 5f appear inconsistent with the rest of the manuscript. These figures are presented as measurements at the detection limit, yet when comparing Figures 3a,b with Figures 5e,f, the amplitudes and noise levels of both the direct and the 2f signals do not match. If Figures 5e and 5f indeed correspond to measurements at the detection limit, the signal characteristics should nearly overlap within the noise level. As currently presented, the comparison does not appear physically consistent, and the authors should clarify / correct these figures.

Another issue concerns the comparison with the reference instrument. This comparison significantly strengthens the manuscript, particularly by lending credibility to the field measurements. However, regarding the quantitative comparison, I would like the authors to elaborate on the statement: "As shown, the signals remain highly consistent across different temperatures, with deviations within 1%." Is this claim supported by all data points? In particular, toward the end of the measurement sequence the discrepancy between the measurements appears larger than 1%. The authors should either correct or moderate this statement and provide a more detailed data analysis e.g. with pointing out the best and worst data match through insets. In particular, it would be useful to quantify the discrepancy (in ppm), attempt to explain it, and compare it with the laboratory measurements ~400 ppm.

A few additional minor issues remain, and I would appreciate it if the authors could address them before publication.

Page 13, line 257: The authors refer to the signal–concentration relation as exponential, whereas it should rather be described as logarithmic. Please correct this. However, I still find it somewhat difficult to describe the dependence as strictly logarithmic. In the theoretical analysis, the relationship is not strictly logarithmic, and the fit shown in Figure R3 is not particularly strong. This is visible not only in Figure R3 itself but also in the residuals, which exhibit a distinct structure rather than purely random noise. I wonder whether a higher-order polynomial fit might lead to smaller residuals than the logarithmic fit. In any case, I would recommend emphasizing that the logarithmic dependence is not directly derived from theory. For the same reason, the statement "The excellent fitting performance confirms the system's robust quantitative capability across a wide concentration range" should be moderated.

I am also concerned about the sensor performance at higher concentration levels, as the sensitivity will decrease rapidly with increasing concentration. It should therefore be clearly stated (in the conclusion?) that the sensor performs great only for low concentration levels, and the relevant operating concentration range should be defined. I believe that even with this limitation, the device would still be suitable for many practical applications. However, could you list some of them, i.e. where sub ppl CO_2 measurement is beneficial?

Finally, regarding the state of the art for on-chip laser absorption spectroscopy sensors, the following paper reports a detection limit of 20 ppm for CO_2 (I referred to this in my previous review) and should be mentioned for completeness: https://doi.org/10.1364/OPTICA.533710

Reviewer #2

(Remarks to the Author)
The authors have largely addressed my comments, and I support publication of the manuscript in its revised form. I appreciate the authors' substantial revision and the extensive additional work performed in response to the reviewer comments. The manuscript has improved significantly compared with the original submission. In particular, the added cross-sensitivity experiments, additional gas measurements, expanded supplementary analysis, and more balanced comparison with prior WGM sensing literature strengthen the technical support for the reported sensing platform.

Judith Su

Reviewer #3

(Remarks to the Author)
The revision has improved the quality of the manuscript greatly. I think the present paper is suitable for publication.

Version 2:

Reviewer comments:

Reviewer #1

(Remarks to the Author)
The authors have satisfactorily addressed all of my comments, and the manuscript quality has improved accordingly.

Open Access This Peer Review File is licensed under a Creative Commons Attribution 4.0 International License, which permits use, sharing, adaptation, distribution and reproduction in any medium or format, as long as you give appropriate credit to the original author(s) and the source, provide a link to the Creative Commons license, and indicate if changes were made.
In cases where reviewers are anonymous, credit should be given to 'Anonymous Referee' and the source.
The images or other third party material in this Peer Review File are included in the article's Creative Commons license, unless indicated otherwise in a credit line to the material. If material is not included in the article's Creative Commons license and your intended use is not permitted by statutory regulation or exceeds the permitted use, you will need to obtain permission directly from the copyright holder.
To view a copy of this license, visit https://creativecommons.org/licenses/by/4.0/

MANUSCRIPT REVISIONS DETAILS

Journal: Nature Communications

Ms. No.: NCOMMS-25-83903

Ms. Title: Sub-ppb CO_2 Detection based on Dissipative Whispering Gallery Mode Microcavity Sensor

Authors: Shujing Ruan, Guangzhen Gao, Jianing Zhang, Haotian Wang, Dongxing Cheng, Jun Guo, Chuanyong Ren, Weidong Chen, Deyuan Shen, and Tingdong Cai

We sincerely thank the Editor and the three reviewers for their careful evaluation and constructive comments on our manuscript. We have revised the manuscript and the Supplementary Information accordingly.

In the revised manuscript and Supplementary Information, all modifications are highlighted in **green** for clarity. In this response document, reviewer comments are shown in ***italic***, our responses are provided in **blue**, and the corresponding revised or newly added text in the manuscript is indicated in **green**.

Reply to Reviewer #1

The manuscript reports an impressive and potentially field-shaping result: a microcavity-based gas sensor achieving sub-ppb detection limits with excellent stability. The work is original, and the demonstrated performance could indeed be relevant for practical applications. However, the theoretical analysis of the sensing mechanism is insufficient, lacks appropriate references, and contains conceptual gaps that undermine the credibility of the interpretation. On the experimental side, while the data are compelling, several performance claims require moderation or further justification. In its current form, the manuscript requires substantial revision before it can be considered for publication in a high-profile journal such as Nature Communications.

We would like to thank the reviewer for the constructive comments and are delighted that the reviewer is interested in our results. We are happy to address all the comments below. We thank the reviewer for motivating those improvements.

Comment 1: S*cientific Assessment*

1. Theoretical description

Although the experimental data clearly show that CO_2 concentration variations lead to measurable signals at sub-ppm levels, the manuscript does not provide a satisfactory physical explanation of this sensitivity. Several key elements of the analysis are missing:

A schematic illustration of the resonator–taper configuration is essential. It should clearly indicate the mode structure, the length of the coupling region l, and the spatial extent of the heated region.

Answer 1: We thank the reviewer for pointing out this important issue. We have added schematic diagrams illustrating (i) the coupling configuration between the tapered fiber and the microsphere WGM cavity and (ii) the increase in the coupling length of the tapered fiber induced by temperature rise. These additions are included in the revised manuscript and the revised Supplementary Materials, respectively, as described below.

We have added Fig. R1 as an inset to Fig.1a in the revised manuscript to describe the coupling configuration between the tapered fiber and the WGM microcavity, as well as to indicate the heated region due to gas absorption:

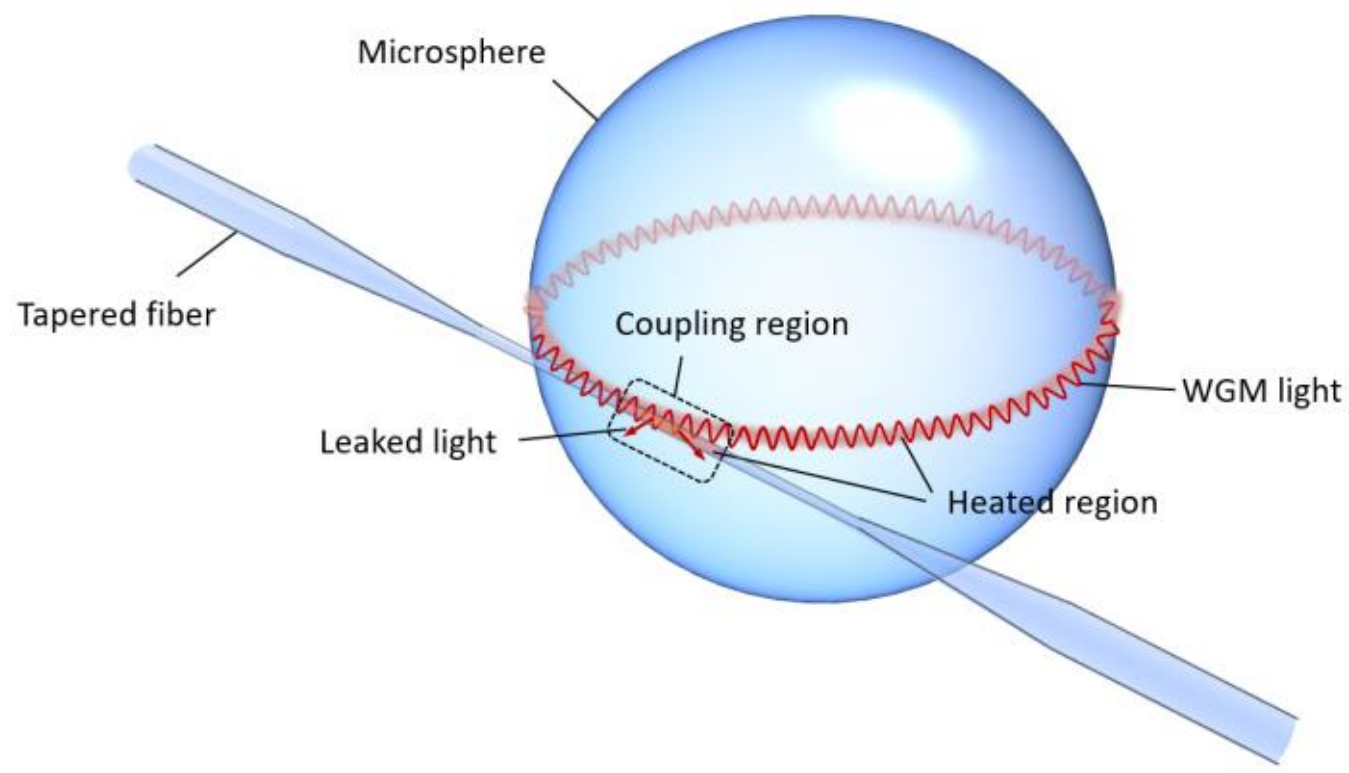

Fig. R1. Schematic diagram of coupling between tapered fiber and microsphere microcavity.

We have added relevant description in the revised manuscript as: "The optical mode in the microsphere microcavity is confined near the equatorial region by total internal reflection, where the field intensity is significantly higher than that in the tapered fiber, as illustrated by the red wavy line in Fig. 1a. The evanescent field component of the optical mode, as well as the light fields that are scattered from the tapered fiber in the coupling region, will be absorbed by the surrounding gas. The absorbed energy gives rise to localized heating, and the generated heat subsequently diffuses along the fiber, the microsphere, and the surrounding air, as indicated by the red shading in the inset of Fig. 1a." on Page 7.

We have added Fig. S4 in the revised supplementary materials to describe the changes in the coupling region before and after heating and expansion. Based on this schematic diagram, we established the relationship between the microsphere radius change δR and the coupling length change δl, which is explained in detail in **Comment 5**.

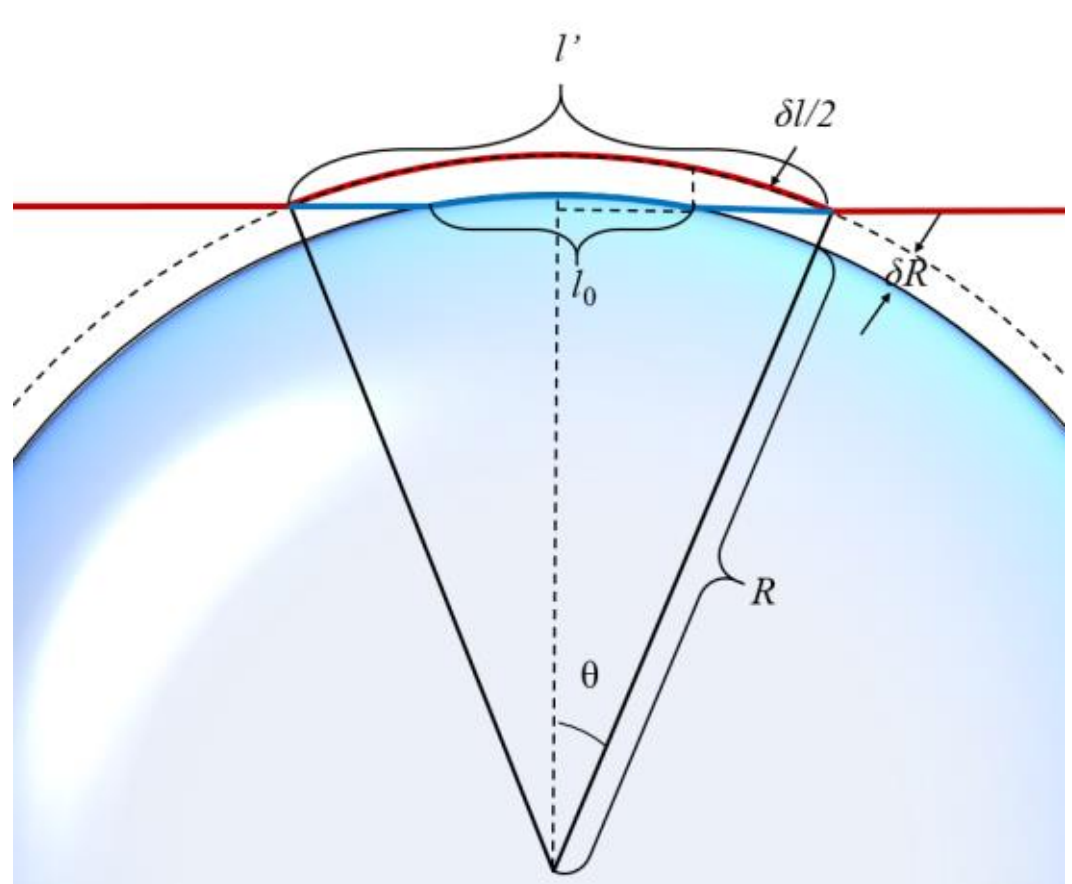

Fig. S4 | Changes in coupling length caused by microsphere expansion before (blue curve) and after (red curve) temperature increase. The black dashed line represents the edge of the

microsphere after expansion.

Comment 2: *The authors should model absorption-induced heating and estimate the local temperature rise around the coupling region. For example: Can the temperature increase plausibly reach ~0.1 K, and what concentration change is needed for such an increase? Over what spatial region is this heating distributed?*

Answer 2: We modeled and analyzed in detail the gas absorption-induced heating, and the additional description has been included in the revised supplementary materials (**Supplementary Note 1.4**).

First, starting from the Beer-Lambert law, we derived the relationship between heating power, gas concentration, and light intensity under the low-concentration approximation [Eqs. (S13)-(S18) in Supplementary Note 1.4]. Then, under the adiabatic approximation, we estimated that the temperature increase induced by gas absorption at a concentration of 1 ppm is 0.04 K/s. Considering heat conduction, we used the finite element numerical method to model heat transfer in the tapered fiber and microsphere microcavity coupling system based on the standard heat conduction equation. The calculation results show that, due to heat diffusion into the microsphere, tapered fiber, and surrounding air, the temperature increase of the microsphere induced by a gas concentration of 3 ppm is 0.0012 K. Furthermore, we found that the heat effectively heats the entire microsphere, and most of the heat dissipates into the surrounding air rather than traveling along the tapered fiber. This is due to the extremely small cross-sectional area of the tapered fiber, which limits the efficiency of heat transfer. This point is crucial, as it guided our analysis of the contribution of changes in coupling length. The analysis in Supplementary Note 1.3 indicates that the change in coupling length primarily depends on the thermal expansion of the microsphere rather than the tapered fiber itself.

We have added **Supplementary Note 1.4** to the supplementary materials to explain this issue:

1.4 Gas absorption induces temperature rise

According to the Beer-Lambert law, the intensity of light absorbed by a gas is:

$$\Delta I = I_0\left(1 - e^{-\alpha(\nu)L}\right) \tag{S15}$$

where I_0 is the initial light intensity, L is the interaction distance in the gas, and α is the absorption coefficient, which is related to the laser frequency v:

$$\alpha(\nu) = Ng(\nu - \nu_0)S \qquad (S16)$$

where N represents the gas molecular number density, S is the absorption line strength, and $g(v\text{-}v_0)$ is the gas absorption line shape function. In the experiment, the linewidth of the laser used is much smaller than the absorption linewidth, and the laser frequency is set at the absorption peak; therefore, the line shape function only needs to consider g_0 at v_0. For a laser wavenumber of 6359.967 cm^{-1}, g_0 and S are 4 cm and 1.76×10^{-23} $cm^{-1}/mol \cdot cm^{-2}$, respectively. The relationship between the absorption coefficient and gas concentration is $\alpha = k \cdot N$, where $k = 7.04 \times 10^{-23}$ cm^2/mol. Therefore, for weak absorption, i.e., $\alpha L << 1$, Eq. S15 can be written as:

$$\Delta I = I_0 \alpha(\nu) L \qquad (S17)$$

Therefore, the heating power is:

$$P_Q = I_0 \alpha L A_Q = I_0 \alpha V \qquad (S18)$$

A_Q and V represent the heating area and volume, respectively. Under the adiabatic approximation (i.e., neglecting heat conduction), the temperature increase per unit time in the heated region is:

$$\Delta T = \frac{P_Q}{\rho c_v V} = \frac{I_0 \alpha}{\rho c_v} \qquad (S19)$$

where ρ and c_v are the density and specific heat capacity of quartz, respectively. In our experiment, I_0 is the light intensity density inside the microsphere WGM cavity, which is significantly enhanced when the laser frequency approaches the microcavity resonance.

$$I_0 = B\Gamma \frac{P_{in}}{A_{eff}} \qquad (S20)$$

P_{in} is the incident power in the tapered fiber, A_{eff} is the mode field area of the WGM in the microcavity, and B is intracavity intensity enhancement factor for the WGM microcavity. The inclusion of the Γ factor accounts for the evanescent field portion of the optical mode interacting with the gas, which leaks outside the microcavity. Furthermore, since the tapered fiber is in close contact with the microcavity surface, strong scattering occurs at the coupling point, significantly increasing the leakage of the light from the microcavity into the surrounding air. Combining Eqs. S19 and S20, the heating rate of the microcavity due to gas absorption at a concentration of 1 ppm can be estimated to be approximately 0.04 K/s, with the parameters used shown in Table S1.

Table S1 The parameter values used for numerical estimation

Parameter	Symbol	Value
Incident power	P_{in}	10 mW
Mode field area	A_{eff}	3 μm^2
Absorption peak	g_0	4 cm
Absorption line strength	S	$1.76 \times 10^{-23} cm^{-1}/mol \cdot cm^{-2}$
Gas molecular number density	N (1 ppm)	1.83×10^{13} mol/cm^3
Thermal expansion coefficient	α_l	0.55×10^{-6} K^{-1}
Thermo-optic coefficient	α_n	10×10^{-6} K^{-1}
Silica density	ρ	2.2 g/cm^3
Temperature	T	300 K
Specific heat capacity	c_v	0.67 J/(g • K)
Constant pressure heat capacity	C_p	0.703 J/(g • K)
Thermal conductivity	k	1.38 W/(m*K)
Light intensity enhancement factor	$B \cdot \Gamma$	~100

Considering that the generated heat diffuses into the microsphere microcavity, the tapered fiber, and the surrounding air, the heat conduction equation is needed:

$$\rho C_p \frac{\partial T}{\partial t} - \nabla \cdot (k \nabla) = Q \qquad \text{(S21)}$$

where T represents temperature, and C_p represents the constant-pressure heat capacity, which for silica is approximately equal to the specific heat capacity c_v. k and Q represent thermal conductivity and generated heat, respectively. Since the composite structure of the tapered fiber and microsphere microcavity is an irregular geometry, a numerical method is required to solve Eq. S21. As described in Fig. 1b in the manuscript, we used a heating ring surrounding the equator of the microsphere to simulate gas absorption-induced heating. The microsphere and tapered fiber were enclosed within a large, air-filled cube. The boundaries of the cube are set to a constant ambient temperature to simulate the large heat capacity of the surrounding environment. The steady-state

temperature distribution is shown in Fig. S6. Fig. S6a shows the temperature distribution at a heat generation per unit volume of $Q = 0.6 \times 10^{6}$ W/m^3 (corresponding to 3 ppm gas absorption), and Fig. S6b shows the temperature variation along the white curve in Fig. S6a. It can be seen that due to heat conduction, the temperature rise is only 0.0012 K compared to the adiabatic condition described in Eq. S19, and the temperature distribution is relatively uniform within the microsphere cavity. The heat does not propagate specifically along the tapered fiber, but rather diffuses into the surrounding air over a range of tens of micrometers. This is because the thermal conductivity cross-sectional area of the tapered fiber is much smaller than that of the surrounding air, even though its thermal conductivity is much greater than that of air.

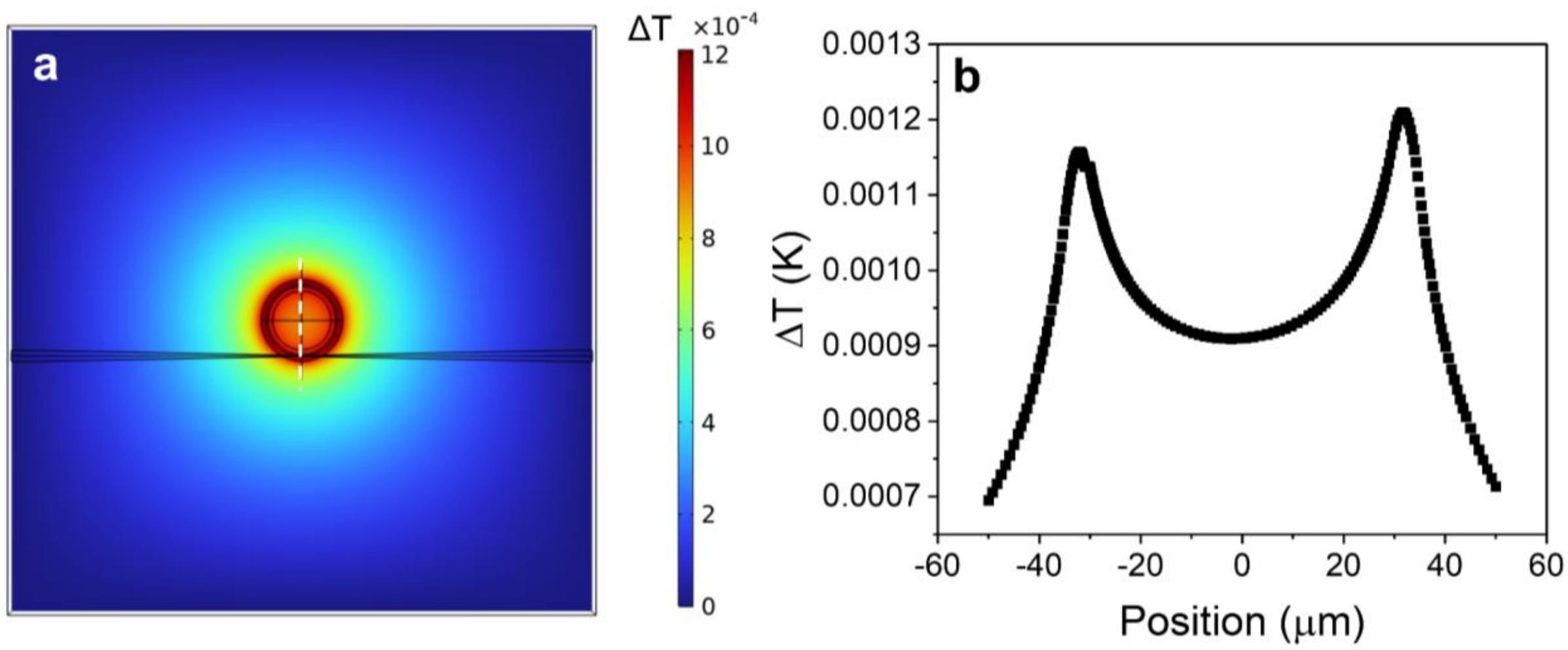

Fig. S6 | a, Temperature variation distribution on the equatorial plane of the microsphere. **b**, Temperature variation along the white dashed line in **a**.

On page 12.

Comment 3: *Using this temperature information, the authors should apply the equations provided in the Supplementary Information to show whether changes in the coupling length can realistically account for the measured signal amplitude.*

Answer 3: The previous answer showed that the temperature increase induced by gas concentrations in the ppm range is on the order of mK. In this answer, we will use this as a starting point and combine it with the proposed theoretical model to estimate the change in sensor signal amplitude, and then compare it with experimental results to verify the correctness of our theoretical

model.

First, we will present the functional relationships between the variables involved in the experiment, as shown in Fig. R2. Except for the logarithmic relationship between the coupling length variation δl and the microcavity radius variation δR (this issue will be discussed in detail in **Comment 5**), all other relationships are linear.

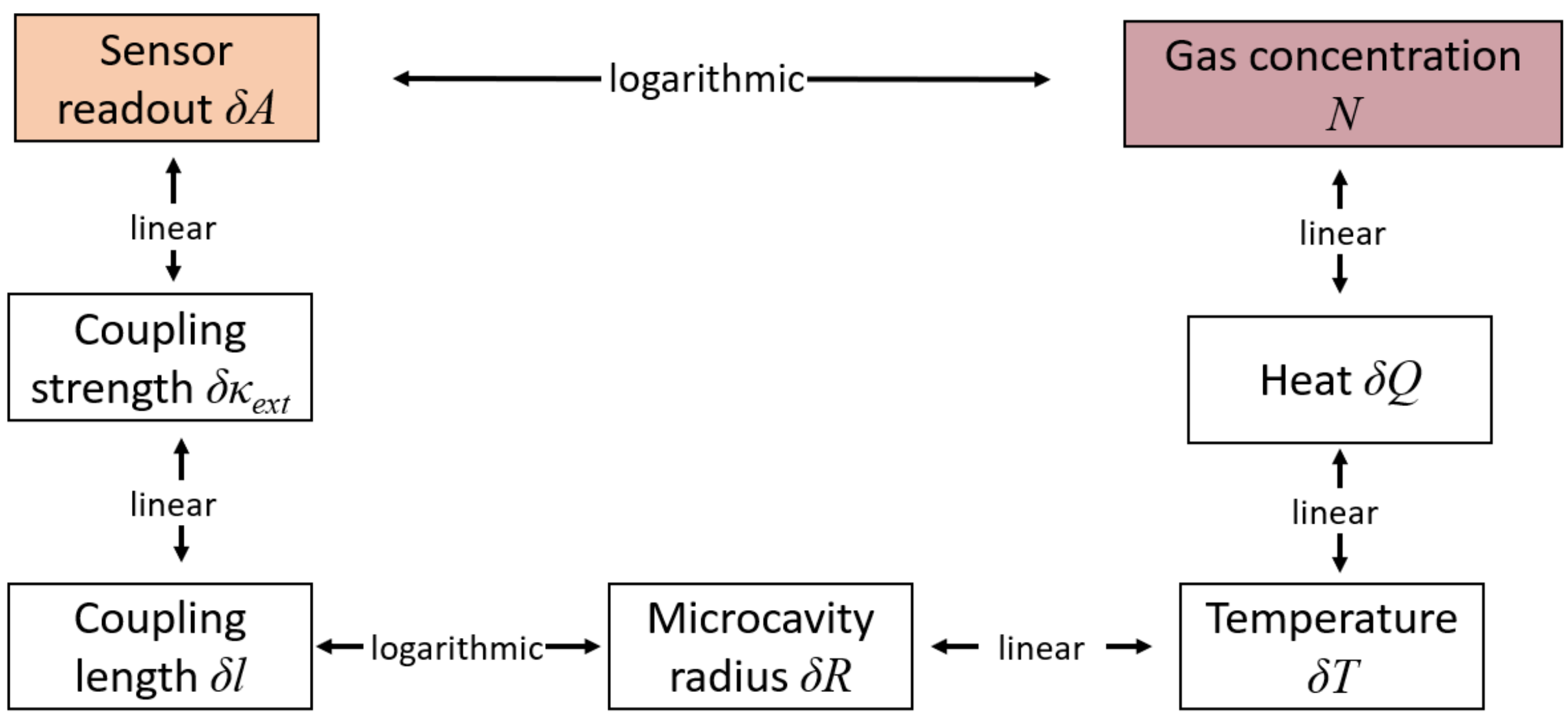

Fig. R2. Block diagram of the relationship between physical variables

Secondly, we will present the magnitude of the slope relationships between different variables. The source of these values will be also explained in detail in Supplementary Note in the revised supplementary materials.

(a) $\delta\kappa_{ext}$ v.s. δA (details in **Supplementary Note 1.1**)

As shown in Eq. 1 in the Methods section of the manuscript and Eq. S6 in Supplementary Note 1.1, starting from the coupled-mode equations, we derived that the readout of our dissipative WGM microcavity sensor is the transmittance at the microcavity resonance. To be consistent with the experimental results (Fig. 3 in the manuscript), we defined a new sensor readout $A = 1 - \Gamma_0$ in the revised Supplementary Note 1.1. Then, based on Eq. S5, we calculated the relationship between the sensor readout A and the coupling strength κ_{ext} for both under-coupled and over-coupled states, as shown in the newly added Fig. S1 in the revised supplementary materials. Based on the calculation results, we found that the sensor exhibits higher sensitivity in the under-coupled state, i.e., the sensitivity of A with respect to κ_{ext} (Fig. S1c):

$$\kappa_{int}\frac{\delta A}{\delta\kappa_{ext}}=3.3 \tag{R1}$$

(b) δl v.s. $\delta\kappa_{ext}$ (details in **Supplementary Note 1.2**)

The influence of refractive index changes on the coupling strength κ_{ext} is mainly reflected through the overlap rate between the WGM microcavity mode field and the tapered fiber mode field, as shown in Eq. S7 in Supplementary Note 1.2. The mode field overlap rate is also affected by the tapered fiber diameter and the coupling position. The influence of coupling length on κ_{ext} is mainly reflected through Eq. S8. When calculating Eq. S7, we first used an axisymmetric model to calculate the mode field in the WGM microcavity and then calculated the mode field of the tapered fiber separately, finally placing them in the same coordinate system, as shown in Fig. S2 in Supplementary Note 1.2 of the revised supplementary materials. For better illustration, we added two-dimensional contour plots in the revised supplementary materials to describe the influence of refractive index, tapered fiber diameter, and coupling length on the coupling strength, as shown in Figs. S2a and S2b. Furthermore, the calculation results show that, within a small range, the coupling strength is linearly related to both the coupling length and the refractive index, as shown in Figs. S2c and S2d. Considering realistic experimental conditions, the influence of refractive index on the coupling strength κ_{ext} under certain temperature changes can be neglected. Therefore, the change in coupling strength κ_{ext} is mainly contributed by the coupling length l, and the sensitivity is (Fig. S2c):

$$\frac{\delta\kappa_{ext}}{\delta l}=1.24\ \text{MHz/nm} \tag{R2}$$

(c) δR v.s. δl (details in **Supplementary Note 1.3**)

As mentioned in the answer to Comment 2, the change in coupling length is primarily due to the expansion of the microsphere, not the tapered fiber itself. A detailed analysis of the relationship between the change in microsphere radius δR and the change in coupling length δl will be presented in the next answer; only the results are given here:

$$\frac{\delta l}{\delta R}=5\times10^{3} \tag{R3}$$

Finally, we can obtain the relationship between the temperature change and the sensor readout:

$$(\kappa_{int}R)\cdot\delta A=\kappa_{int}\frac{\delta A}{\delta\kappa_{ext}}\frac{\delta\kappa_{ext}}{\delta l}\frac{\delta l}{\delta R}R\left(\frac{1}{R}\frac{\partial R}{\partial T}\right)\cdot\delta T \tag{R4}$$

where κ_{int} and R represent the intrinsic decay rate and radius of the microsphere microcavity, respectively, and $\alpha_l = (1/l)\cdot(\partial l/\partial T)$ is the thermal expansion coefficient of silica microsphere. According to the experimental results, as shown in Fig. 4a of the manuscript, when the gas concentration changes from 1.5ppm to 3ppm, the sensor readout changes by approximately 0.005. Substituting R = 30 μm and κ_{int} = 300 MHz, corresponding to an intrinsic $Q_{int} \sim 4 \times 10^6$, and $\alpha_l = 0.55 \times 10^{-6}$ into the above equation, we obtain a corresponding change in sensor readout of 0.005 (after normalization) for a temperature change of 0.004 K. The magnitude of this temperature change is very close to the heat conduction calculation results in Supplementary Note 1.4. Therefore, it can be proven that our theoretical model is reasonable.

We have added the following description to **Supplementary Note 1.1** to explain the relationship between sensor reading A and coupling strength κ_{ext}:

For our dissipative WGM sensors, the change in light intensity transmittance at the resonance can be used as the sensor readout $A=1-\Gamma_0$, as shown in Fig. 3a of the manuscript. Since the change in κ_{int} caused by light absorption by low-concentration gases is negligible, its main contribution comes from the change in κ_{ext}.

According to Eqs. S5 and S6, assuming κ_{int} is a constant, we calculated the transmission spectrum of the WGM microcavity at different coupling strengths κ_{ext}, and the relationship between the sensor readout A and κ_{ext}, as shown in Fig. S1. In the under-coupled ($\kappa_{ext} < \kappa_{int}$) and over-coupled ($\kappa_{ext} > \kappa_{int}$) states, the sensor reading A increases and decreases, respectively, with increasing κ_{ext}. Furthermore, the rate of change of A with respect to $\kappa_{ext}/\kappa_{int}$ is much higher in the under-coupled state than in the over-coupled state, at 3.3 and -0.136, respectively. Therefore, in the experiment, setting the dissipative WGM microcavity sensor in the under-coupled state can significantly improve sensitivity.

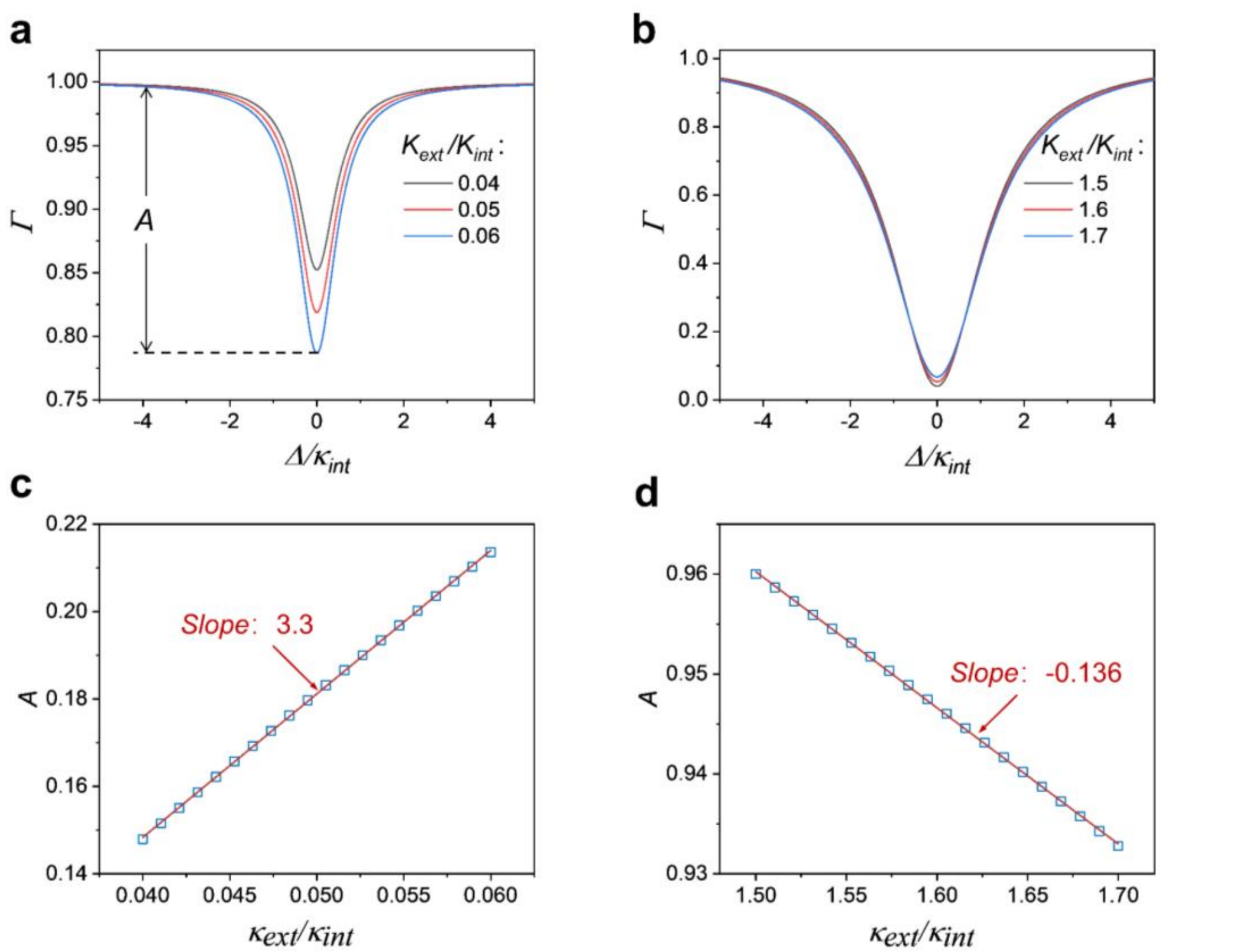

Fig. S1 | The WGM transmission lineshapes of the microsphere cavity under different κ_{ext} in under-coupling **a** and over-coupling **b** states. **c** and **d**, represent the linear relationship between sensor readout A and κ_{ext} under these two coupling states, respectively.

On page 3.

We have added the relevant discussion to the "**Theory and simulations**" section of the manuscript:

According to Eq. (1), we can relate $\delta\kappa_{ext}$ to temperature change δT through thermal expansion and the thermo-optic coefficient:

$$\frac{\delta\Gamma_0}{\Gamma_0} = \frac{4\kappa_{int}\kappa_{\mathrm{ext}}}{|\kappa_{ext}^2 - \kappa_{int}^2|}\left[\frac{\partial\kappa_{\mathrm{ext}}}{\partial n} n\alpha_n \delta T + \frac{\partial\kappa_{\mathrm{ext}}}{\partial l}(\delta l_1 + \delta l_2)\right] \quad (3)$$

The first term in the parenthesis on the right side corresponds to the thermo-optic effect, where n and $\alpha_n = 1/n \cdot \partial n/\partial T$ denote the refractive index and the thermo-optic coefficient of silica, respectively. δl_1 and δl_2 represent the variations in coupling length induced by the thermal expansion of the tapered fiber and the microsphere, respectively.

On page 24.

We have added the following description to **Supplementary Note 1.2** to further explain the relationship between coupling length l and coupling strength κ_{ext}:

The mode field distributions of the WGM microcavity and the tapered fiber at the cross-section of the coupling region were calculated using the finite element numerical method. To more clearly illustrate the overlap ratio of the mode field in Eq. S7, we calculated the modes of the microcavity and the tapered fiber separately and plotted them in the same coordinate system. Figs. S2a and S2b show the superimposed mode fields: that is, the distribution of $|\boldsymbol{E}_f|^2/N_f+|\boldsymbol{E}_r|^2/N_r$ in the coupling region. The diameters of the microcavity and the tapered fiber are 60 μm and 0.6 μm, respectively. Fig. S2c shows the distribution of the mode field intensities of the microcavity and the tapered fiber along the white curve in Fig. S2b. When the diameter of the tapered fiber is <1 μm, a large amount of evanescent field will leak from the fiber, thus enhancing its coupling strength with the microcavity.

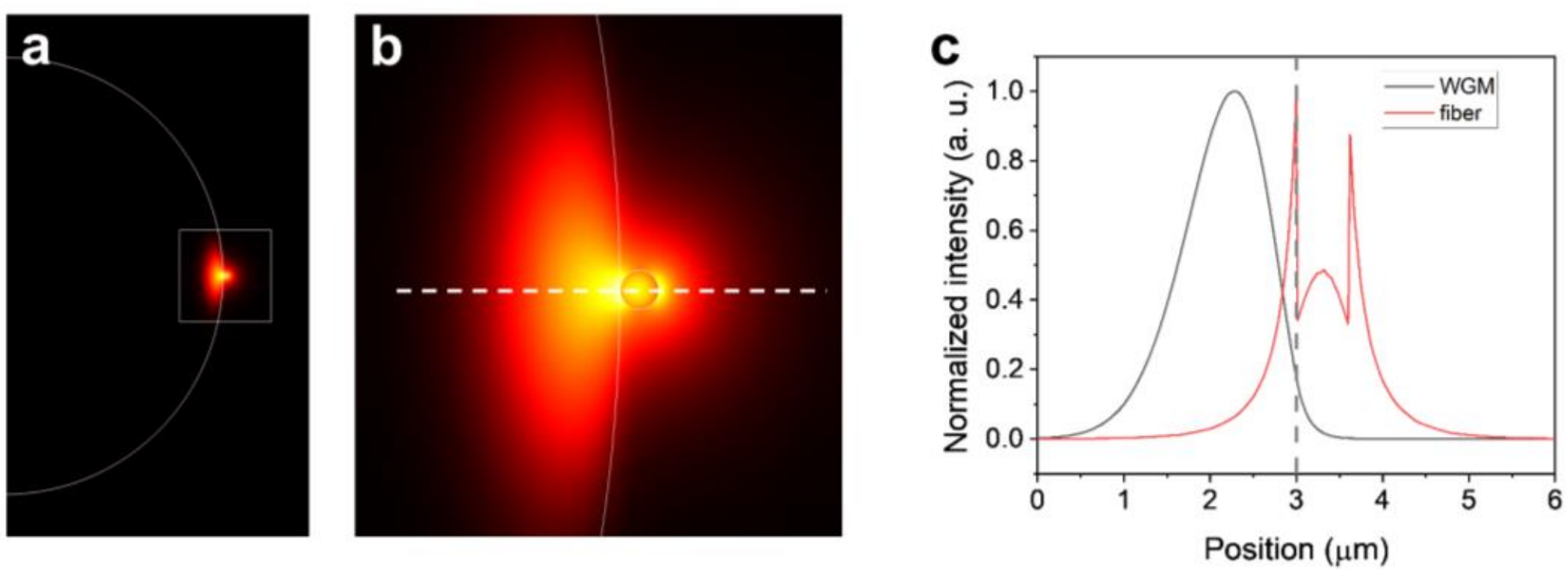

Fig. S2 | **a**, Mode field distribution ($|\boldsymbol{E}_f|^2/N_f+|\boldsymbol{E}_r|^2/N_r$) of microsphere cavity and tapered fiber in the coupling region. **b**, Zoom within the white square in **a**. **c**, The black and red curves represent the mode field distributions of the WGM mode ($|\boldsymbol{E}_r|^2/N_r$) and the tapered fiber mode ($|\boldsymbol{E}_f|^2/N_f$) along the white curve in **b**, respectively. The black vertical curve marks the boundary of the microsphere.

According to Eqs. S7-S9, the relationship between the coupling strength κ_{ext} and the coupling length, fiber diameter, and refractive index was calculated, as shown in Fig. S3. Fig. S3a shows a 2D contour plot of the coupling strength as a function of coupling length and fiber diameter. Initially, the coupling strength increases rapidly with increasing coupling length, but with decreasing fiber diameter, it shows a trend of increasing first and then decreasing. This is because the initial decrease in fiber diameter increases the proportion of the evanescent field, thus increasing the overlap between the mode field of the fiber and the microcavity mode. However, further reducing the diameter causes the fiber mode to deviate from paraxial propagation, i.e., the axial electric field component increases, which significantly reduces its overlap with the microcavity mode, specifically the numerator of the integrand in Eq. S7. Fig. S3b shows a 2D contour plot of the coupling strength as a function of coupling length and refractive index. At a

fixed coupling length, the change in coupling strength is only on the order of kHz within a refractive index change of δn = 0.0003. Fig. S3d shows the sensitivity of the coupling strength to the refractive index: -2.1 MHz/RIU. Since the refractive index n changes only by an order of 10^{-5} for a 1 K temperature variation, the influence of the thermo-optic effect can be ignored in our sensor.

Because the coupling strength κ_{ext} varies only by a few MHz in the experiment, it exhibits a linear relationship with the coupling length within this range, as shown in Fig. S3c. At a tapered fiber diameter of 0.6 μm, the sensitivity of the coupling strength to the coupling length is 1.24 MHz/nm.

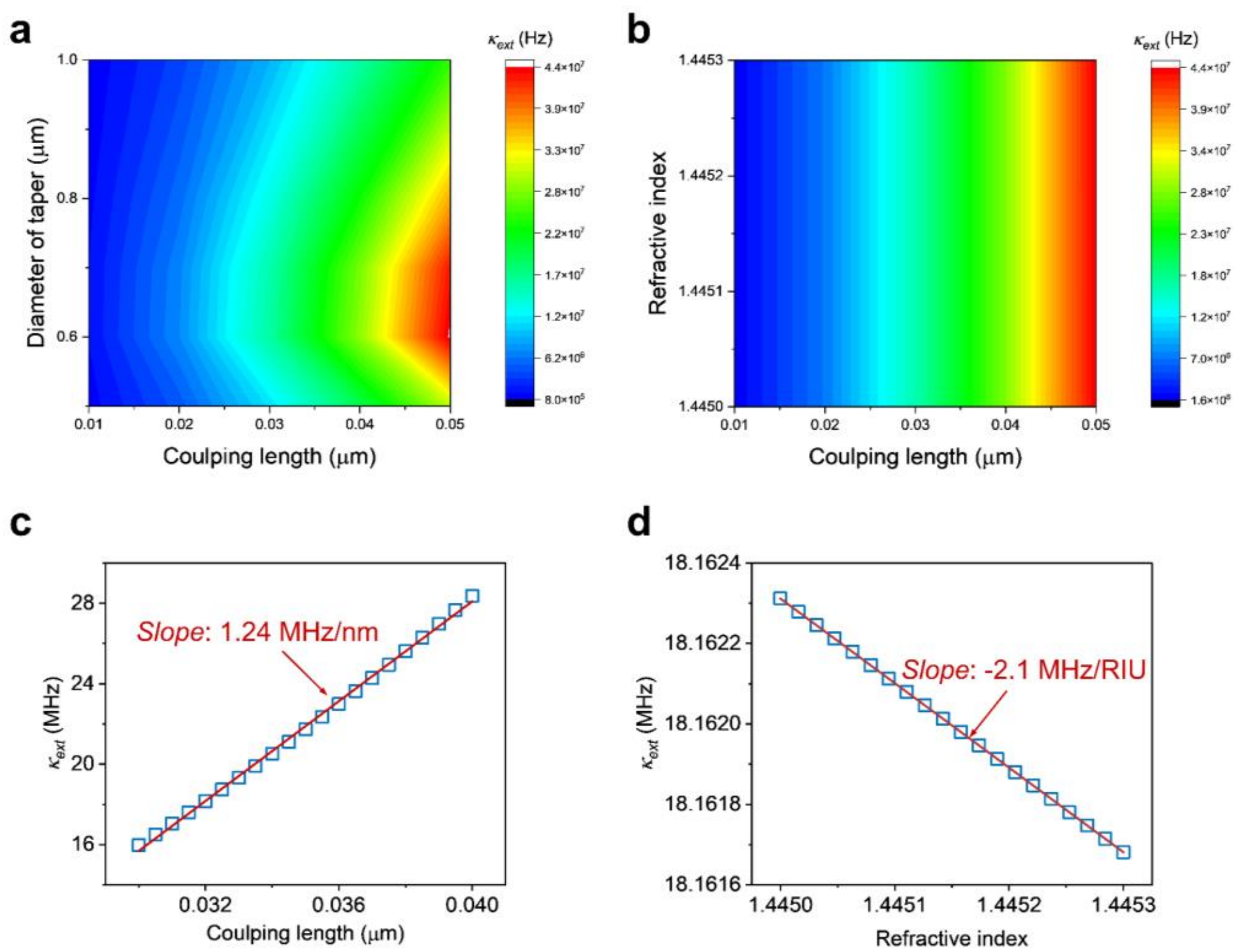

Fig. S3 | a, Two-dimensional contour plot of coupling strength varying with coupling length and tapered fiber diameter. **b**, Two-dimensional contour plot of coupling strength varying with coupling length and refractive index. **c**, The linear relationship between coupling strength and coupling length when the diameter of the tapered fiber is 0.6μm. **d**, The linear relationship between coupling strength and refractive index when the tapered fiber diameter is 0.6μm and the coupling length is 30 nm.

On page 6.

We have added **Supplementary Note 1.5** to further explain the connection between the

theoretical model and the experimental results, thereby demonstrating the validity of the model:

1.5 Comparison between theoretical estimation and experimental results

In this section, we use the theoretical model described above to perform numerical estimations and validate the model against experimental results. According to Fig. 4a in the manuscript, the experimentally measured signal change is 0.005 (normalized) for a gas concentration change of 1.5 ppm (from 1.5 ppm to 3 ppm). Based on the sensitivity of the coupling strength to the sensor readout (~3.3) shown in Fig. S2c of Supplementary Note 1.1, this signal change requires a change in coupling strength κ_{ext} of approximately 0.45 MHz. Here, we assume the intrinsic loss of the microsphere is 300 MHz, corresponding to an intrinsic Q factor $Q_{int} \sim 4 \times 10^6$. Then, using the sensitivity of κ_{ext} to the coupling length l obtained from Fig. S4 in Supplementary Note 1.2: ~1.24 MHz/nm, we can deduce that the coupling length change δl is 0.36 nm. From Eq. S12 and the logarithmic fitting curve in Fig. S5 of Supplementary Note 1.3, we obtain the slope near the zero point, $\delta l/\delta R \sim 5 \times 10^3$. Therefore, a δl change of 0.36 nm corresponds to a δR change of 0.07 pm. Using the thermal expansion formula: $\delta R = R \cdot \alpha_l \cdot \delta T$, and substituting the microsphere radius of 30 μm and the thermal expansion coefficient $\alpha_l = 0.55 \times 10^{-6}$, we obtain a required temperature change of 0.004 K. This result agrees well with the calculated temperature increase due to gas absorption at 3 ppm shown in Fig. S6 (0.0012 K). Therefore, the validity of our theoretical model can be proven.

On page 13.

Comment 4: *If this mechanism is valid, the authors need to show how thermal expansion or refractive-index modifications lead to the observed increase in effective coupling length. The schematic mentioned above is important for conveying this clearly.*

Answer 4: As discussed in Supplementary Note 1.4 and Comment 2, the heat induced by gas absorption does not propagate over long distances along the tapered fiber but is confined to a region of only tens of micrometers. The increase in coupling length caused by such a short heating length is negligible compared to the experimental results. Therefore, we established a model for the increase in coupling length caused by microsphere expansion, as shown **in Fig. S4 in Supplementary Note 1.3** in the revised supplementary materials.

The blue and red curves in Fig. S4 represent the lengths of the tapered optical fiber in contact with the surface of the microsphere before and after its expansion, respectively. Based on

geometric relationships, we can derive a functional relationship between the change in microsphere radius δR and the coupling length δl (the length of the fiber attached to the microsphere surface):

$$\delta l = l' - l_0 = 2(R + \delta R) \cdot \arccos \frac{R - \Delta}{R + \delta R} - l_0 \tag{R5}$$

where l_0 is the initial coupling length, and Δ is a small correction term introduced to satisfy the condition that $\delta l = 0$ when $\delta R = 0$. The detailed derivation process has been added to Supplementary Note 1.3 of the revised supplementary materials. Eq. R5 shows that the smaller l_0 is, the more sensitive the change in δl is relative to the change in δR. Therefore, in the experiment, we minimized the initial coupling length by optimizing the diameter of the tapered fiber in the coupling region and its coupling position. According to the calculation results, when l_0 = 20 nm, the sensitivity of the change in δl relative to the change in δR is 5×10^3. Based on this sensitivity value, we estimated that the theoretical conclusions are very close to the experimentally measured results.

We have added **Supplementary Note 1.3** to further explain the changes in coupling length caused by temperature increase:

1.3 Temperature-induced change in coupling strength

In this section, we will analyze the effect of temperature changes on κ_{ext}. The change of κ_{ext} is mainly caused by the heat generated by the gas absorbing light, which will be written as:

$$\delta \kappa_{\text{ext}} = \frac{\partial \kappa_{\text{ext}}}{\partial n} n \alpha_n \delta T + \frac{\partial \kappa_{\text{ext}}}{\partial l} \delta l \tag{S10}$$

The first term on the right-hand side of the above equation represents the contribution due to the thermo-optic effect, where $\alpha_n = (1/n) \cdot (\partial n / \partial T)$ is the thermo-optic coefficient of silica.

The change in coupling length δl due to temperature has two main contributions. Firstly, the heat generated by the absorption of light by the gas is conducted along the tapered fiber, causing the tapered fiber to heat up and expand within a certain range of the coupling region, resulting in a change of δl_1:

$$\delta l_1 = l_T \alpha_l \delta T \tag{S11}$$

where $\alpha_l = 1/l \cdot \partial l / \partial T$ is the thermal expansion coefficient of quartz, and l_T is the heated tapered fiber region.

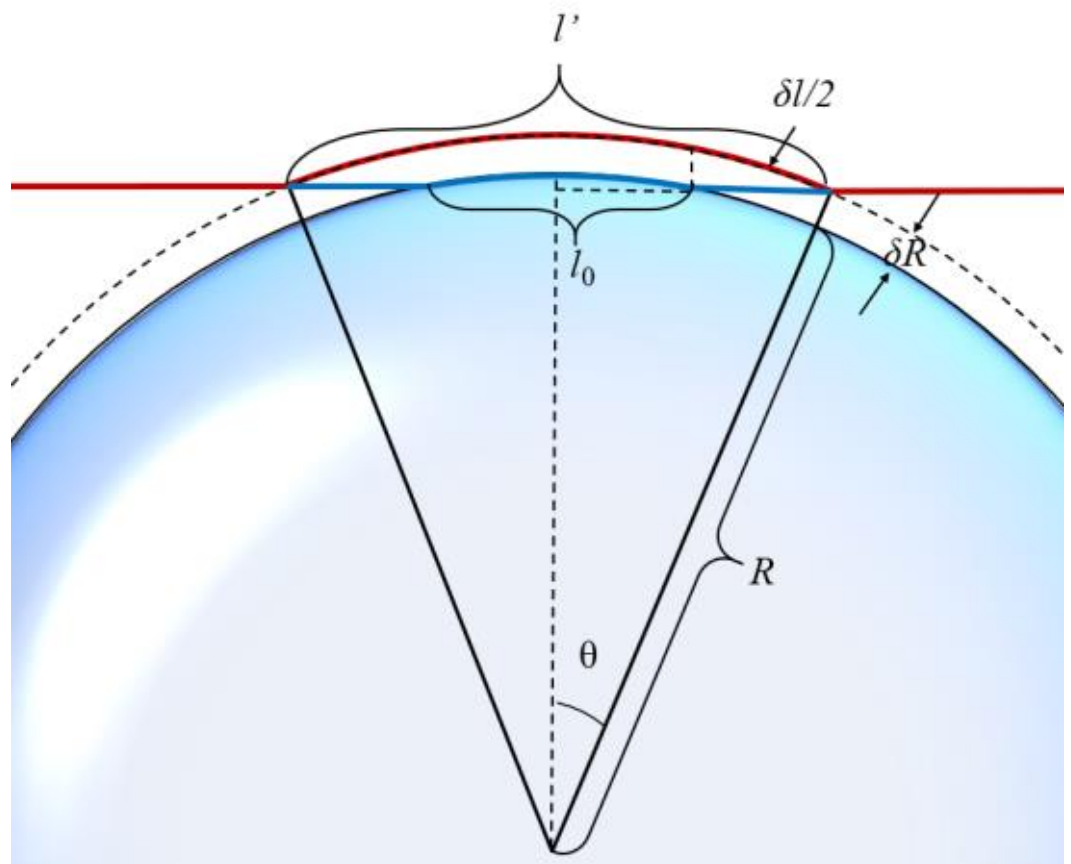

Fig. S4 | Changes in coupling length caused by microsphere expansion before (blue curve) and after (red curve) temperature increase. The black dashed line represents the edge of the microsphere after expansion.

On the other hand, light absorption by the gas causes thermal expansion of the microsphere microcavity, which in turn extends the length of the tapered optical fiber attached to the surface of the microcavity. As shown in Fig. S4, when the tapered fiber is coupled to the microsphere cavity, a section of the fiber is in contact with the microsphere surface, and the coupling region appears as an arc. The blue and red arcs represent the coupling length before and after expansion, respectively, and the solid and dashed black lines represent the edge contours of the microsphere before and after expansion. Based on the geometric relationship in the figure, the change in coupling length δl_2 caused by the change in microcavity radius can be approximately expressed as:

$$\delta l_2 = l' - l_0 = 2(R + \delta R) \cdot \arccos \frac{R - \varDelta}{R + \delta R} - l_0 \tag{S12}$$

where R is the microcavity radius, δR is the expansion of the microcavity radius, and l_0 is the initial coupling length. In the above derivation, we assumed that the angle θ is very small, which is reasonable because the microsphere radius is on the order of tens of micrometers, while the coupling length l_0 is only on the order of tens of nanometers. The value of the correction term $\varDelta$ can be determined by ensuring that when $\delta R = 0$, δl_2 must be zero in the numerical calculation of Eq. S12.

According to Eqs. S10-S12, the change in coupling strength due to temperature variations can be derived:

$$\delta\kappa_{\text{ext}} = \frac{\partial\kappa_{\text{ext}}}{\partial n} n\alpha_n \delta T + \frac{\partial\kappa_{\text{ext}}}{\partial l}(\delta l_1 + \delta l_2)$$
$$= \frac{\partial\kappa_{\text{ext}}}{\partial n} n\alpha_n \delta T + \frac{\partial\kappa_{\text{ext}}}{\partial l}\left[l_T \alpha_l \delta T + 2(R+\delta R)\cdot \arccos\left(\frac{R-\Delta}{R+\delta R}\right) - l_0\right] \tag{S13}$$

where $\delta R = R\alpha_l\delta T$.

According to Eqs. S6, S10, and S13, the relationship between the sensor readout A and the temperature change δT can be derived. The first, second, and third terms on the right side of Eq. S13 represent the changes in coupling strength due to the thermo-optic effect, the expansion of the tapered fiber, and the expansion of the microsphere, respectively. Based on the calculation results in Fig. S3d, the change in coupling strength caused by the thermo-optic effect in the experiment is negligible. Furthermore, according to the heat conduction model (see the analysis in Supplementary Note 1.4), the heated region of the tapered fiber is only tens of micrometers, and its contribution to the change in coupling strength is also negligible. Therefore, the change in coupling strength mainly depends on the expansion of the microsphere WGM cavity, i.e., $\delta l \approx \delta l_2$.
On page 6.

Comment 5: *Finally, the theoretical framework should be used to explain why the sensor response follows a logarithmic (not exponential!) dependence on concentration. This is currently unaddressed.*

Answer 5: According to Eq. S12, we calculated the relationship between δR and δl, as shown by the blue squares in Fig. R3. It is evident that the logarithmic function provides a good fit (red curve). As a result, δR and δl exhibit a logarithmic relationship:

$$\delta l = 268 + 64\ln(\delta R + 0.015)$$

As shown in Fig. R1 in the answer to Comment 3, and Figs. S1c and S3c in the Supplementary Materials, all variables exhibit linear relationships except for the logarithmic relationship between δR and δl. Therefore, we can theoretically deduce that there is also a logarithmic relationship between the sensor reading A and the gas concentration N, which is consistent with experimental observations.

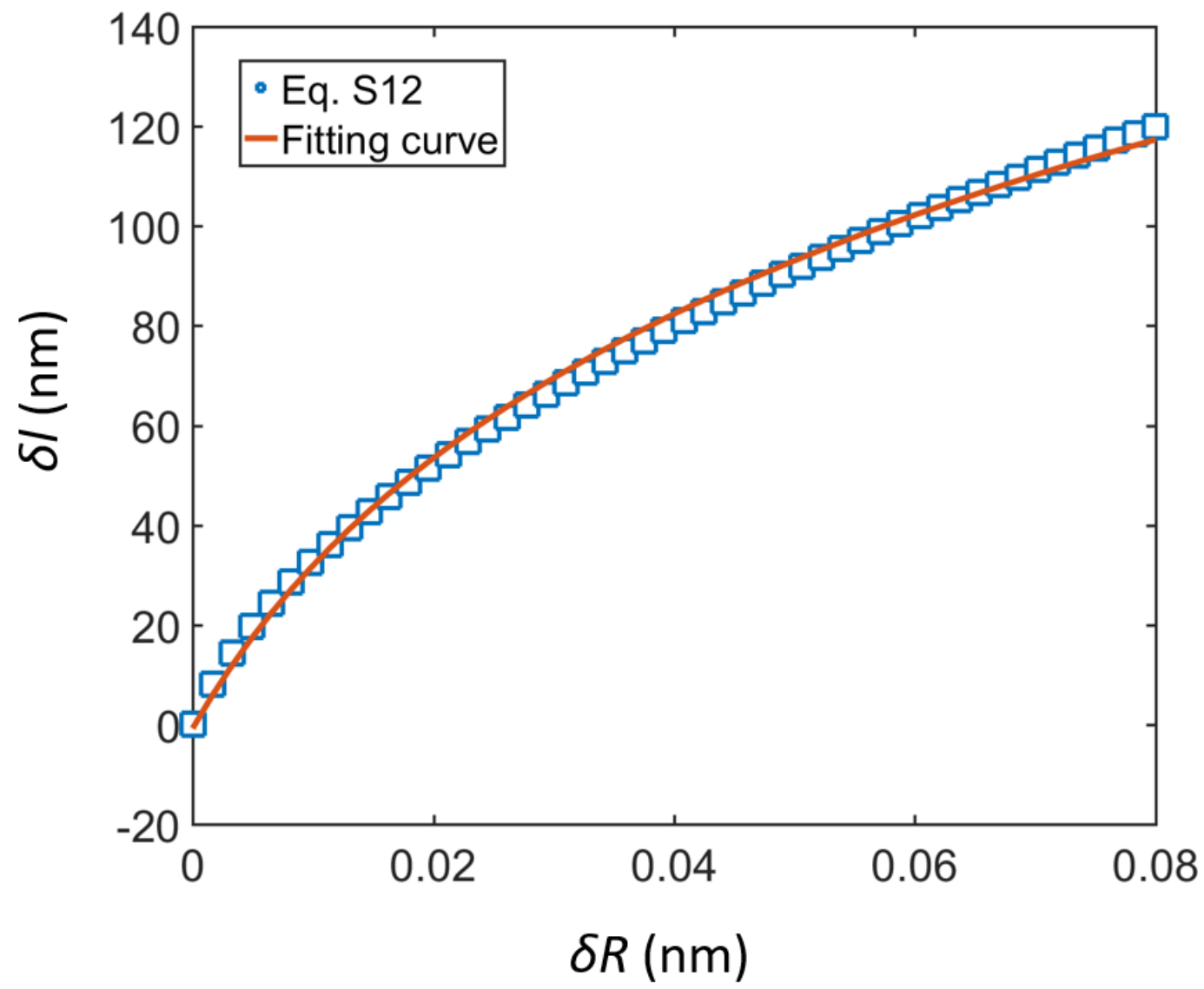

Fig. R3. The logarithmic relationship between the change in coupling length δl and the change in microsphere radius δR.

We have added Fig. R3 as Fig. S5 to Supplementary Note 1.3 in the supplementary materials, along with the relevant discussion:

As shown in Fig. S5, the blue squares represent the numerical results using Eq. S12. In the calculation, l_0, R, and Δ were set to 20 nm, 30 μm, and 1.65 pm, respectively. Clearly, there is a nonlinear relationship between δl_2 and δR. We used a logarithmic function for fitting and found that it fits very well. The fitted function is:

$$\delta l = 268 + 64 \ln(\delta R + 0.015) \tag{S14}$$

According to Figs. S1c and S3c, we find that the sensor readout A is proportional to the change in coupling length δl. On the other hand, due to the expansion of the microcavity caused by gas absorption, at low concentrations, the gas concentration N is proportional to the temperature increase of the microcavity (see Supplementary Note 1.4), and consequently proportional to the change in microsphere radius δR. According to Fig. S5, δl has a logarithmic relationship with δR; therefore, it can be deduced that the sensor readout A has a logarithmic relationship with the gas concentration N, which is consistent with the experimental results shown in Fig. 4 of the manuscript. On page 8.

Comment 6: *2. Experimental Considerations and Data Interpretation*
Several issues in the data presentation and performance evaluation should be addressed:
While the Allan deviation demonstrates the detection limit, the precision varies strongly across the concentration range. What is the precision around 400 ppm? Fig.s 4 and 5a should be enlarged or supplemented with zoomed-in plots to show the actual scatter.

Answer 6: We thank you for raising this critical point regarding the variation in measurement precision across the concentration range and for the suggestion to better visualize the data scatter. These comments are very helpful for improving the clarity of our data presentation.

In response to your suggestion, we have performed a detailed re-analysis of the raw data for each concentration (from 1.5 ppm to 400 ppm).

For each of the ten concentration levels, we have plotted the distribution of the 20 individual data points using box-and-scatter plots, which are now included as an enlarged supplementary panel in the revised manuscript (see the updated Figs. 4g-4p). Each box plot displays the median, mean, and the full distribution of the 20 data points, while the overlaid scatter points allow for direct visual assessment of the dispersion of individual measurements. Each panel compares the mean value, precision, and dispersion at a given concentration for both the raw optical mode signal and the 2*f* signal detection methods. These measurement points support the curve fitting and Allan deviation mentioned later.

The analysis quantitatively confirms the precision (expressed as the standard deviation, 1σ) does vary with concentration. In particular, the precision at high concentrations is now explicitly reported, while the precision at other concentrations can be directly observed from the plots. Specifically, at **400 ppm**, the calculated precision is:
Raw optical mode signal: 5.69 ppm; **2*f* signal:** 4.01 ppm.

This demonstrates that even at high concentrations, the 2*f*-WMS method maintains superior precision, consistent with its noise-suppression advantages. The observed increase in the absolute precision (expressed in ppm) with concentration is an expected consequence of high-relative-accuracy measurement. Crucially, the relative precision remains consistently high across the entire range. Analysis of the 20 data points at each concentration shows that the relative standard deviation (RSD) is approximately 1% for 2*f* signal detection methods at all tested levels (from 1.5 to 400 ppm). This indicates excellent measurement repeatability. The increase in the absolute uncertainty (e.g., from ~0.016 ppm at 1.5 ppm to ~4.01 ppm at 400 ppm) simply reflects the scaling of this constant relative error with the concentration magnitude itself. Therefore, the system

demonstrates stable relative performance, which is the key metric for assessing sensor consistency across its dynamic range.

We hope the addition of these detailed distribution plots and the explicit reporting of concentration-specific precision address your concerns. They provide a transparent and granular view of our data's reliability.

We have revised Fig. 4 and Fig. 5 and merged them into a new Fig. 4 in the revised manuscript as:

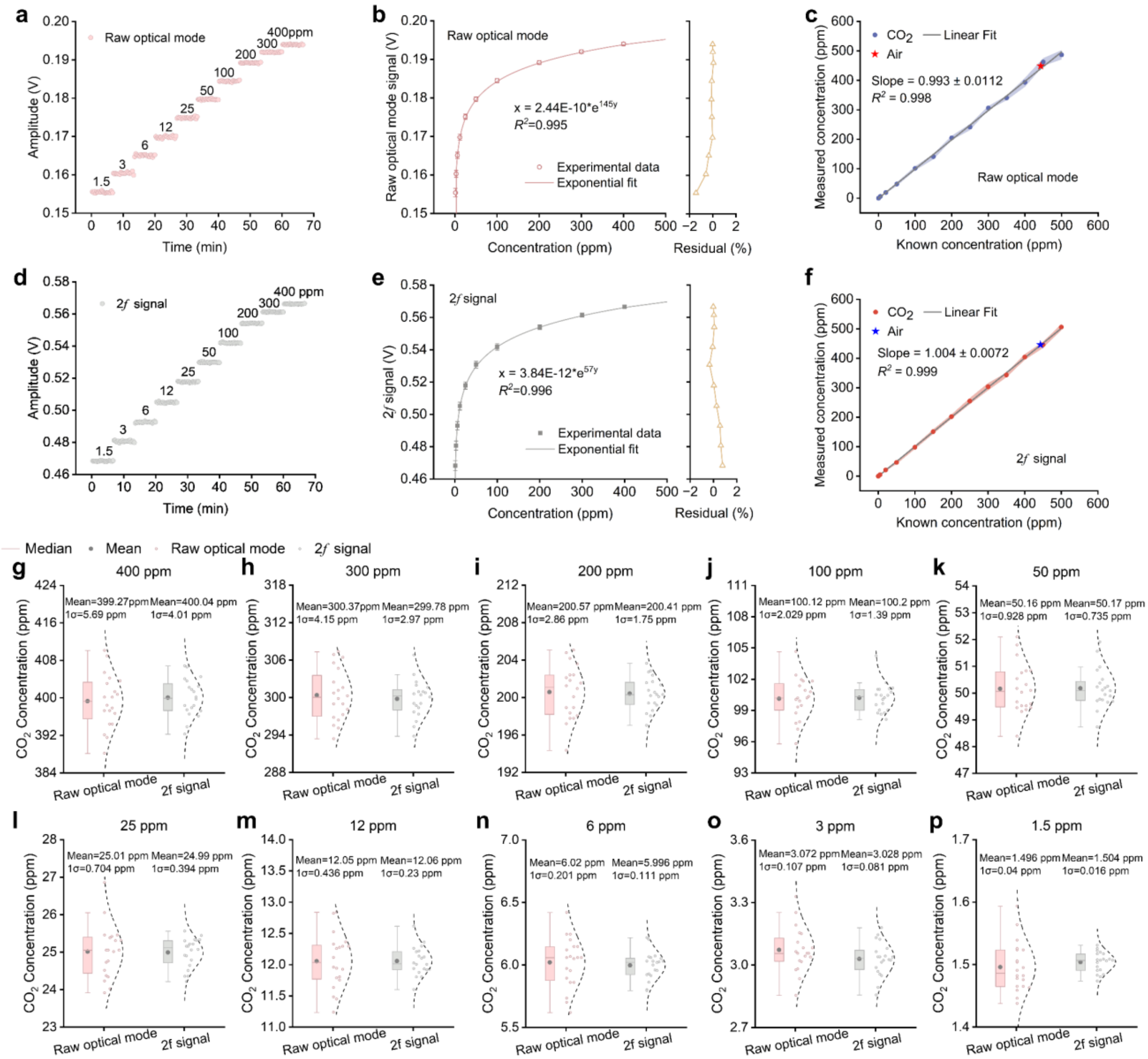

Fig.4 | Dynamic response and calibration of CO_2 sensing. a, The measured optical mode amplitude versus calibration time for different CO_2 concentration levels ranging from 1.5 to 400 ppm. **b**, Experimental data points and the corresponding fitted curve of optical mode amplitude as

a function of CO_2 concentration, showing residuals within 2%. **c**, Comparison between the CO_2 concentration measured by the raw optical mode and the nominal concentration recorded during gas preparation, the shaded area is the error bar. **d**, The measured 2*f* amplitude versus calibration time for different CO_2 concentration levels ranging from 1.5 to 400 ppm. **e**, Experimental data points and corresponding fitted curve of 2*f* amplitude as a function of CO_2 concentration, showing residuals within 1%. **f**, Comparison between the CO_2 concentration measured by 2*f* signal and the nominal concentration recorded during gas preparation, the shaded area is the error bar. **g** to **p**, Analysis of data discrepancy at various concentrations measured in Figs. 4a and 4d. on Page 11.

We have added relevant discussion in the revised manuscript as:
"To provide a detailed assessment of measurement precision and repeatability across the entire dynamic range, statistical analysis was performed on the measured concentration data. As shown in Figs. 4g to 4p, box-and-scatter plots are presented for each of the ten tested concentration levels (from 1.5 ppm to 400 ppm). Each plot displays the distribution of 20 consecutive measurements obtained under stable conditions. The box indicates the interquartile range (IQR), median and mean, while the overlaid individual points illustrate the actual scatter of the data. Both detection methods exhibit consistently high relative repeatability across all concentrations, with a relative standard deviation (RSD) of approximately ±3% in raw optical mode signal and an RSD of approximately ±1% in 2*f* signal. This confirms the stable relative performance of the sensor throughout its operational range." on Page 11.

Comment 7: *Fig.s 4c and 5c are not meaningful, as the same data used for calibration is plotted again, making a linear response inevitable.*

Answer 7: Thank you for raising this critical methodological point. You are entirely correct that evaluating linearity using the same dataset employed for calibration is circular and does not constitute an independent validation of sensor performance. We acknowledge this oversight in our original analysis and have performed a comprehensive new set of experiments specifically designed to address this issue. Below, we detail our corrective actions and the supporting results.

New experimental procedure:

A series of 14 CO_2 concentrations (0.001, 0.1, 5, 20, 50, 100, 150, 200, 250, 300, 350, 400, 450, and 500 ppm) were freshly prepared using a precision gas mixer. Each concentration was

measured independently using both the raw optical mode and the 2*f*-WMS method. Each measured concentration value is obtained by averaging 6 measurements. The asterisks in Figs. 4c and 4f represent the comparison of the results measured by the two methods with the analyzer.

Results:

The sensor responses from these independent test points are plotted against the reference concentrations, as shown in Fig. R4. The data continue to show strong linearity across the tested range:

Raw optical mode signal: R^2 = 0.998, slope = 0.993 ± 0.0112 corresponding to a measurement accuracy of ~0.7%.

2*f* signal: R^2 = 0.999, slope = 1.004 ± 0.0072, corresponding to a measurement accuracy of ~0.4%.

The close-to-unity slopes and high R^2 values obtained from this independent dataset confirm that the sensor exhibits excellent linear response, which is intrinsic to the detection principle and not an artifact of self-fitting. The slightly higher accuracy of the 2*f*-WMS method is consistent with its better noise immunity, as also reflected in the Allan deviation and reference analyzer comparisons.

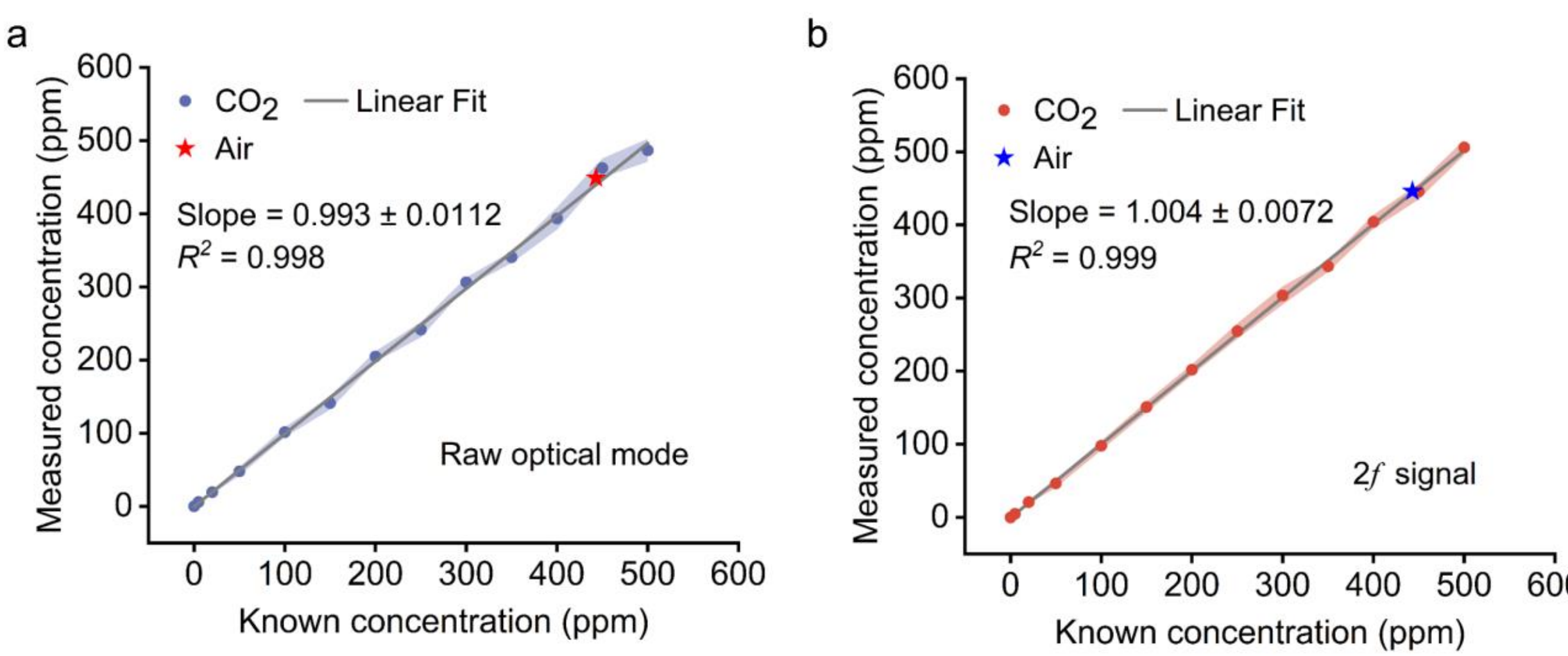

Fig. R4. a, Comparison between the CO_2 concentration measured by the raw optical mode and the nominal concentration recorded during gas preparation, the shaded area is the error bar. **b,** Comparison between the CO_2 concentration measured by 2*f* signal and the nominal concentration recorded during gas preparation, the shaded area is the error bar.

Fig. R4 is represented as Figs. 4c and 4f in the revised manuscript. We have added relevant discussion in the revised manuscript as:

"To independently validate the accuracy of the sensor and avoid potential bias from using the same dataset for calibration and validation, a separate verification experiment was conducted. A series of CO_2 concentrations (0.001, 0.1, 5, 20, 50, 100, 150, 200, 250, 300, 350, 400, 450, and 500 ppm), distinct from the concentrations used for the initial calibration, were prepared using a precision gas mixer. Each concentration was measured independently using both the raw optical mode and 2*f* signal, and the reported values represent the average of six consecutive measurements. Sensor performance was evaluated by comparing the measured concentrations with the corresponding reference values, as shown in Figs. 4c and 4f. Excellent linearity was obtained over the entire tested range. For the raw optical mode, a linear fit yields a slope of 0.993 ± 0.0112 with $R^2 = 0.998$, corresponding to a measurement accuracy of approximately 0.7%. The 2*f* signal exhibits better performance, with a fitted slope of 1.004 ± 0.0072, $R^2 = 0.999$, and an accuracy of approximately 0.4%." on Page 13.

"The asterisks in Figs. 4c and 4f represent the comparison of the results measured by the two methods with the analyzer. When measuring in outdoor atmospheric environments, the absolute deviations from the reference value were approximately 6-8 ppm for the raw optical mode and 3-4 ppm for the 2*f* signal, corresponding to relative accuracies of about ±2% and ±1% of the reading, respectively." on Page 14.

Comment 8: *The direct-absorption and 2f-WMS methods differ by ~10 ppm near 400 ppm. What is the resulting accuracy after calibration? An independent reference measurement would be very helpful.*

Answer 8: Thank you for this insightful comment regarding the discrepancy between the two methods and the need for an independent accuracy assessment. We agree that an independent reference measurement is crucial for validating the absolute accuracy.

In direct response to your suggestion, we have conducted a new controlled experiment. A commercial-grade, calibrated CO_2 analyzer (GMM-1000, specified accuracy ±1% of the reading) was used as the independent reference, as shown in Fig. R5. The practical applicability of this system was evaluated through re-measuring the ambient CO_2 concentration. A comparison was performed using three measurement methods: the raw optical mode measurement, the 2*f* signal measurement method, and a standard commercial analyzer. The concentrations were determined

to be 450 ppm and 446 ppm using the raw optical mode signal and 2*f* signal, respectively. The concentration was determined to be 443 ppm using a standard commercial analyzer. The results demonstrate the higher accuracy of the 2*f*-WMS method. Across the measurement range, the 2*f*-WMS data showed excellent agreement with the commercial reference, with an absolute deviation of approximately 3-4 ppm. In contrast, the raw optical mode data exhibited an absolute deviation of approximately 6-8 ppm relative to the reference, directly explaining the ~10 ppm difference observed between the two methods in our original ambient measurement.

Therefore, the resulting accuracy after calibration against the independent standard is: For the 2*f* method: ~3-4 ppm, the accuracy is approximately ±1% of the reading. For the direct-absorption method: ~6-8 ppm, the accuracy is approximately ±2% of the reading. The primary sources of error include uncertainties in gas mixing, imperfect peak extraction, laser frequency instability, and limitations in environmental pressure and temperature control accuracy.

We have incorporated a summary of this comparative analysis and its results into the revised manuscript to provide a validated benchmark for the system's accuracy, as shown in Fig. R6. The primary error sources for the raw optical mode signal method, as previously noted, are indeed more effectively mitigated by the 2*f*-WMS technique, which is now quantitatively confirmed against an independent standard.

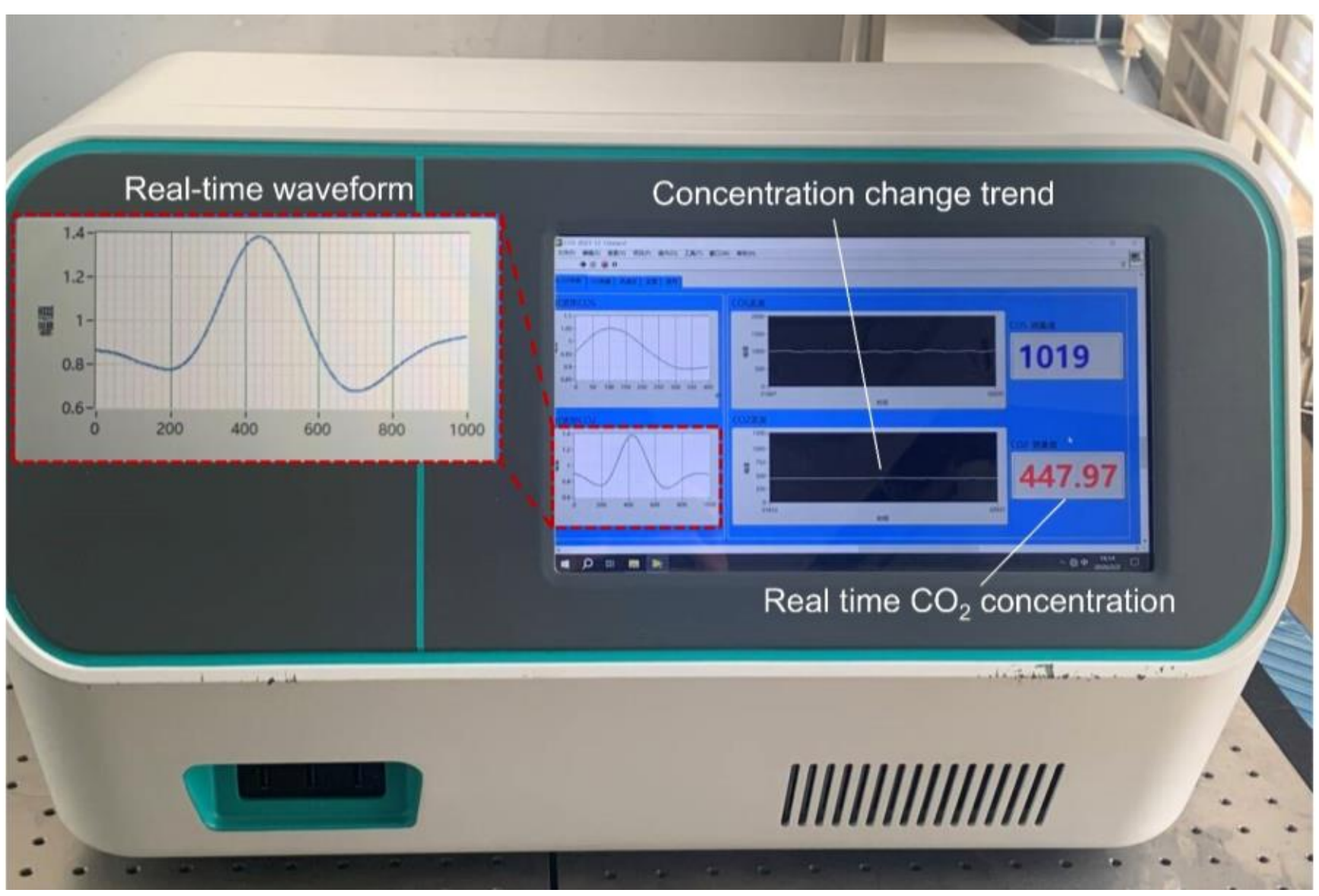

Fig. R5. Commercial grade calibrated CO_2 analyzer.

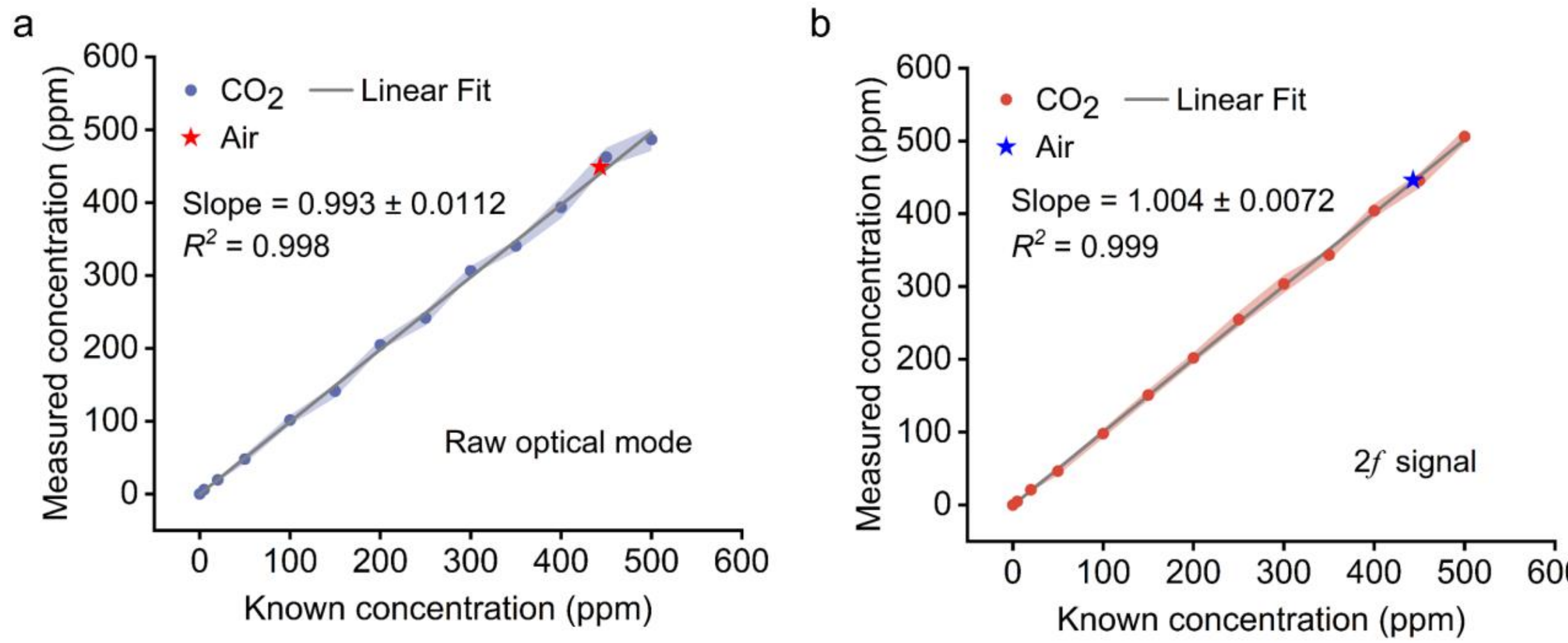

Fig. R6. **a,** Comparison between the CO_2 concentration measured by the raw optical mode and the nominal concentration recorded during gas preparation, the shaded area is the error bar. **b,** Comparison between the CO_2 concentration measured by 2*f* signal and the nominal concentration recorded during gas preparation, the shaded area is the error bar.

We have added relevant discussion in the revised manuscript as:

"The absolute accuracy of the concentration measurements and the practical applicability of the system were further assessed through an independent comparison experiment under ambient CO_2 conditions. Measurements were carried out simultaneously using the raw optical mode, the demodulated 2*f* signal, and a commercial calibrated CO_2 analyzer (GMM-1000, specified accuracy ±1% of reading), which served as the reference instrument. The analyzer reported a concentration of 443 ppm, while the raw optical mode and 2*f* method yielded 450 ppm and 446 ppm, respectively. The asterisks in Figs. 4c and 4f represent the comparison of the results measured by the two methods with the analyzer. When measuring in outdoor atmospheric environments, the absolute deviations from the reference value were approximately 6-8 ppm for the raw optical mode and 3-4 ppm for the 2*f* signal, corresponding to relative accuracies of about ±2% and ±1% of the reading, respectively. The main sources of uncertainty include gas mixing errors, peak extraction inaccuracies, laser frequency fluctuations, and limited precision in pressure and temperature control. Overall, these results confirm the superior accuracy of the 2*f*-WMS approach, which benefits from enhanced noise suppression and reduced sensitivity to baseline drift." on Page 14.

Comment 9: *Fig. 6c contains an incorrect legend and should be corrected.*

Answer 9: We appreciate your careful review and for pointing out the error in the legend of Fig. 6c. We have corrected it by swapping the labels for "Raw optical mode signal" and "2*f* signal" accordingly. As shown in Fig. R7, in the modified Fig., we separate the two Allan deviations and plot them separately. The revised figure has been updated in the manuscript, and the curves now correctly match their respective labels. This change does not affect the data or conclusions, and is solely a clarification.

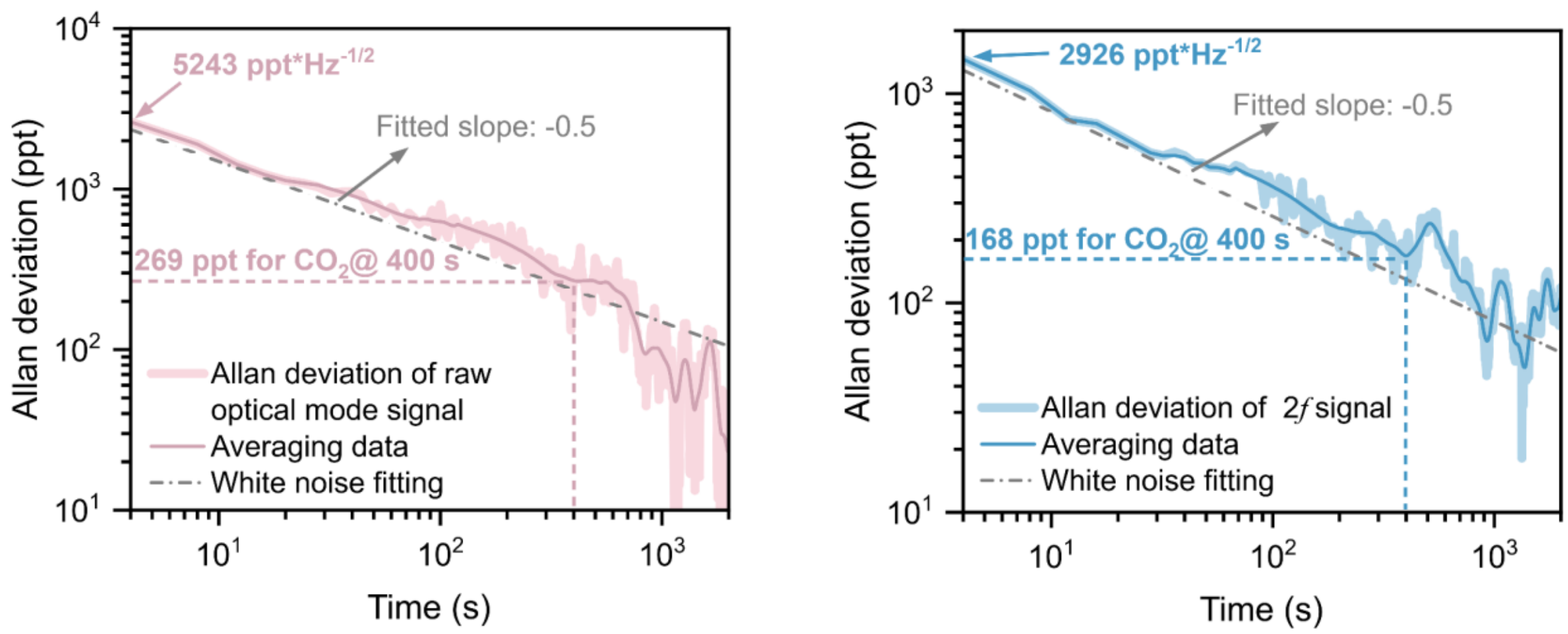

Fig. R7. Allan deviation plot of the dissipative WGM gas sensor.

Comment 10: *The presented traces appear overly smooth. The authors should show raw, unprocessed resonance data, including the noise (e.g., Figs. 3 and 6d,e).*

Answer 10: We appreciate your careful observation regarding the apparent smoothness of the resonance traces. We would like to clarify that the data shown in the Fig. 3 are raw and have not been subjected to any smoothing or filtering post-processing. To further clarify this point, we have added enlarged views of the signals in the revised figures to explicitly show the noise level of the raw data.The high signal-to-noise ratio achieved under the current experimental conditions results in traces that appear smooth at the scale of the main plots. We have added enlarged views in the revised Figs. 3 and 5e, 5f, the noise characteristics at both the resonance peak and the baseline. After analysis, it can be seen that the original optical mode signal is modulated and demodulated

to generate a $2f$ signal, which greatly improves the signal-to-noise ratio and effectively suppresses noise.

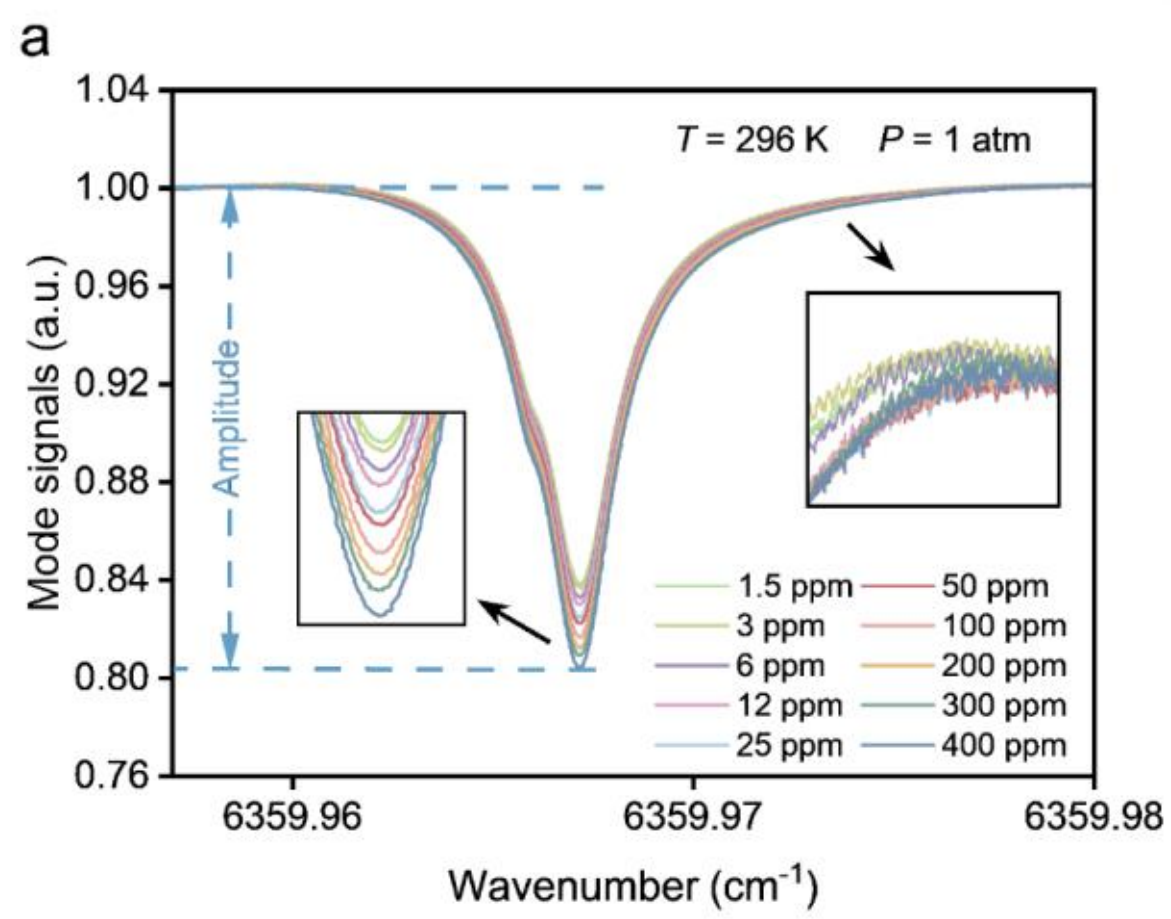

Fig.3 | Sensor response to varying CO_2 concentrations. a, The raw optical mode signals measured at different CO_2 concentrations. **b**, The $2f$ signals measured at different CO_2 concentrations.

We have added relevant discussion in the revised manuscript as:

"The improvement in detection performance provided by the modulation-demodulation scheme was examined by recording both the direct optical resonance signal (without modulation) and the demodulated second-harmonic ($2f$) signal at each concentration level. The optical resonance traces shown in Fig. 3 are raw data, without any smoothing or filtering in post-processing. For clarity, each main spectrum is accompanied by a magnified inset that highlights the detailed spectral structure and intrinsic noise characteristics." on Page 10.

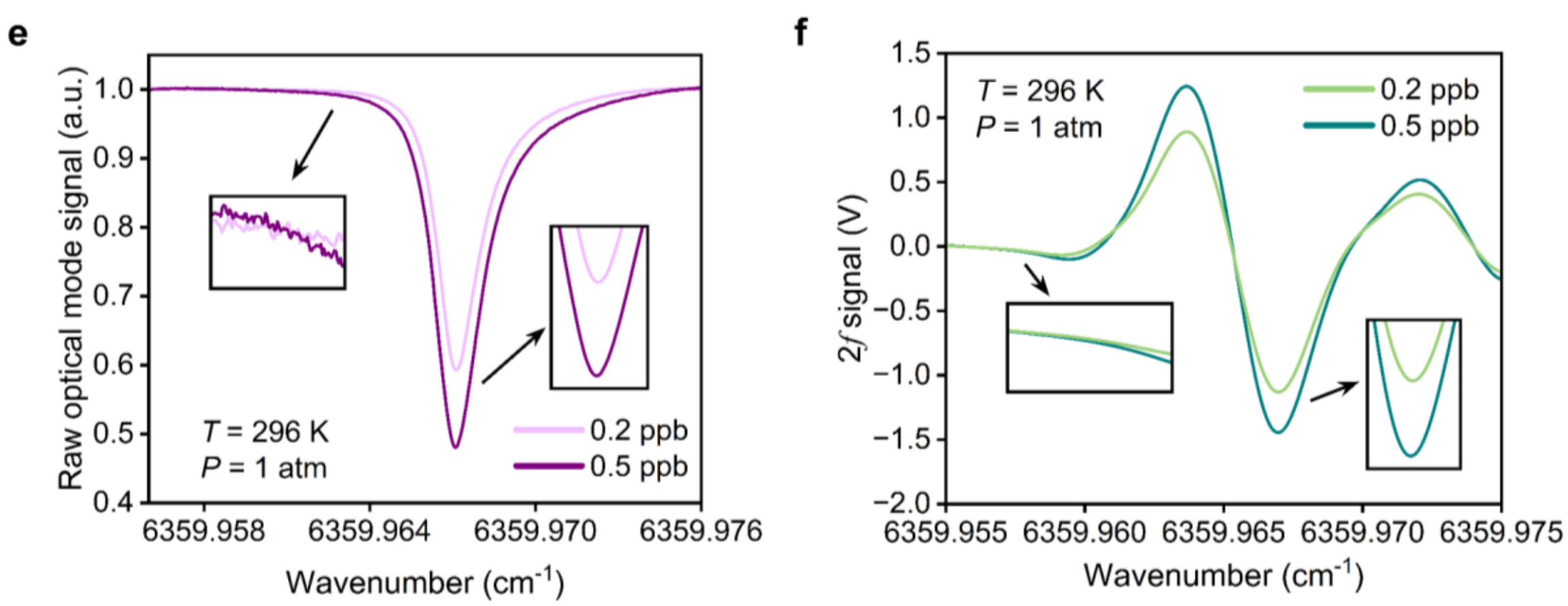

Fig.5 | e and **f,** Raw optical mode signals and $2f$ signals for CO_2-N_2 mixtures at 0.2 ppb and 0.5

ppb concentrations with approximately 400 s integration time.

We have added relevant discussion in the revised manuscript as:
"To verify the reliability of the Allan-derived detection limits, additional measurements were performed on CO_2/N_2 mixtures with concentrations of 0.2 ppb and 0.5 ppb, prepared through multiple dilution steps, using an integration time of 400 s. The corresponding raw optical mode and 2*f* responses are shown in Figs. 5e and 5f, respectively. The magnified insets reveal clear signal separation between the two concentration levels, with well-resolved features above the intrinsic noise floor. These results demonstrate that the sensor maintains high sensitivity and stable performance even near its detection limit." on Page 15.

Comment 11: *The laser linewidth used for interrogation should be specified.*

Answer 11: Thank you for this important comment.
We used an unbalanced Michelson interferometer to measure the laser noise. The advantage of this method is that it can measure the amplitude of laser frequency fluctuations at different Fourier frequency offsets and derive the laser linewidth. As shown in Fig. R8a, the laser signal under test is split into two paths by a 2×2 3dB coupler. The first path passes through a 100m fiber delay line and is then reflected by a Faraday reflector (FRM). The second path is simply reflected by the FRM. The FRMs are used to eliminate the influence of polarization fluctuations in the interferometer on the interference signal. The two reflected beams interfere within the coupler, and the resulting optical signal is converted into an electrical signal by a photodetector (PD).

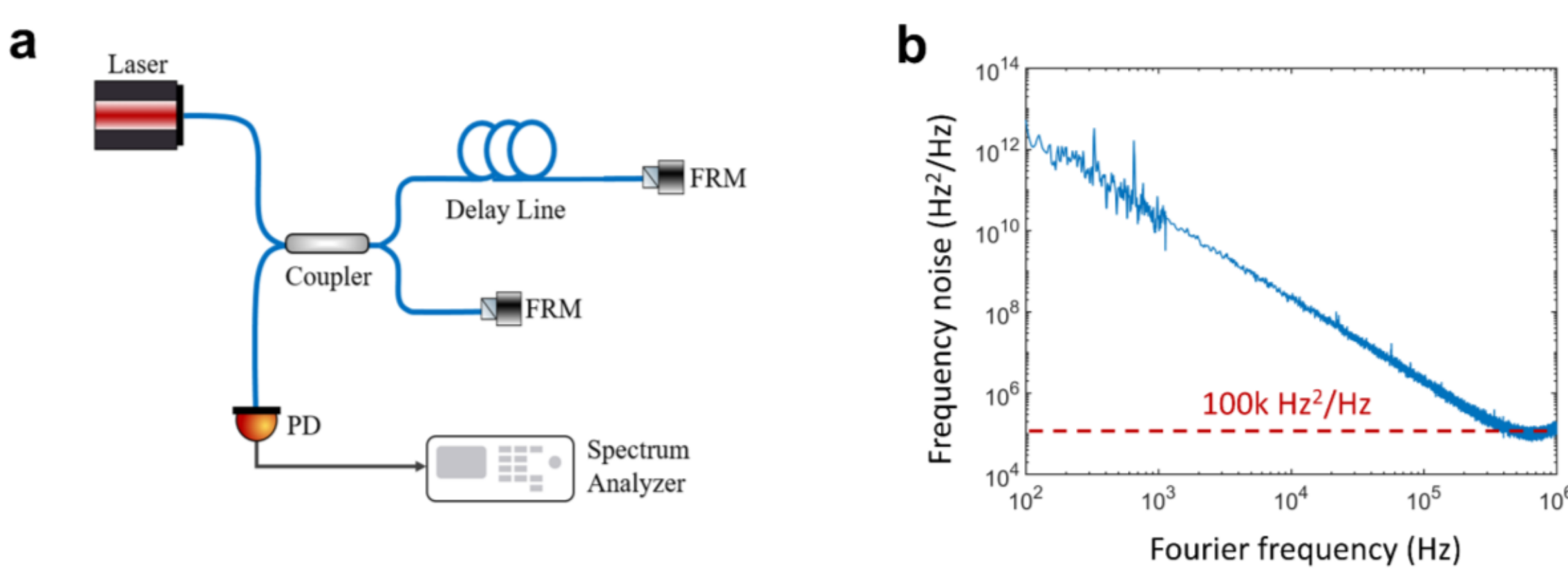

Fig. R8. a, Experimental setup for laser frequency noise measurement. **b**, Measured power spectral density of the laser frequency noise; the red dashed line indicates white noise.

The data is then acquired by a data acquisition card and processed using a computer program to obtain the laser frequency noise power spectral density (PSD). The data processing flow is as follows: photocurrent $I(t)$ → Hilbert transform → differential phase fluctuation $\Delta\varphi(t)$ → Fourier transform → differential phase PSD $S_{\Delta\varphi}$ → multiplied by frequency discrimination accuracy → laser phase noise PSD S_φ → laser frequency noise PSD S_v. The measured laser frequency noise PSD is shown in Fig. R8b.

The experimentally measured laser frequency noise PSD is shown in Fig. R8b. Near the Fourier frequency offset of 1 MHz, the laser frequency noise exhibits white noise characteristics, and the laser linewidth $\delta v = \pi \cdot S_v$ can be obtained. Since the white noise is caused by quantum fluctuations of the laser, it can also be called the Schawlow-Townes (ST) linewidth. Based on the measurement results, we calculated the laser's ST linewidth to be approximately 0.3 MHz. This linewidth is smaller than the 2 MHz claimed in the laser's factory test report. Because, in addition to quantum noise, some technical noise, such as environmental fluctuations causing changes in the laser cavity length, can increase the laser linewidth. For the sake of accuracy, we still use the linewidth from the factory report in the manuscript. Therefore, compared to the mode linewidth of the microcavity, the laser linewidth is sufficiently narrow to accurately characterize the microcavity modes.

We have added the laser linewidth information to the manuscript: "A distributed feedback (DFB) tunable diode laser (NEL, NLK1L5GAAA) with the linewidth of ~2MHz centered at ~1572.3 nm (Fig. 1d) was used" on Page 7.

Comment 12: *Outdoor measurements:*

The field measurement data lack significance without parallel measurements using a calibrated high-end instrument.

Answer 12: Thank you for your valuable comment regarding the significance of our outdoor measurement data. We fully agree that parallel measurements with a calibrated reference instrument are essential for validating sensor performance under real-world conditions. In direct response to your suggestion, we have conducted a new two-day outdoor monitoring campaign in which our dissipative WGM-based CO_2 sensor was operated side-by-side with a calibrated, high-end commercial CO_2 analyzer (model GMM-1000, calibration traceable to Chinese metrological

standards).

The results of this comparative measurement are now presented in the revised manuscript (new Fig. 6a). As shown, the concentration trends recorded by our sensor exhibit excellent consistency with those from the commercial instrument over the entire monitoring period. A detailed correlation analysis yields a high coefficient of determination ($R^2 > 0.95$), and the absolute deviation between the two datasets falls within an acceptable range for environmental monitoring applications (± 5 ppm). These results confirm that our sensor reliably captures the characteristic diurnal variations of atmospheric CO_2, including the nighttime peak and daytime drawdown, as influenced by local biogenic and anthropogenic activity.

The successful parallel validation strengthens the practical relevance of our work. It demonstrates that our system, while achieving a competitive limit of detection (168 ppt) and favorable practical attributes (ease of alignment, small sample volume, low cost), also performs robustly in unshielded, fluctuating ambient conditions. We have updated the “Real-Time Monitoring of Ambient CO_2” section accordingly and explicitly discuss the comparative results and their implications. We believe this addition substantially enhances the validity and impact of our field-testing claims.

We have revised Fig. 6 in the revised manuscript as:

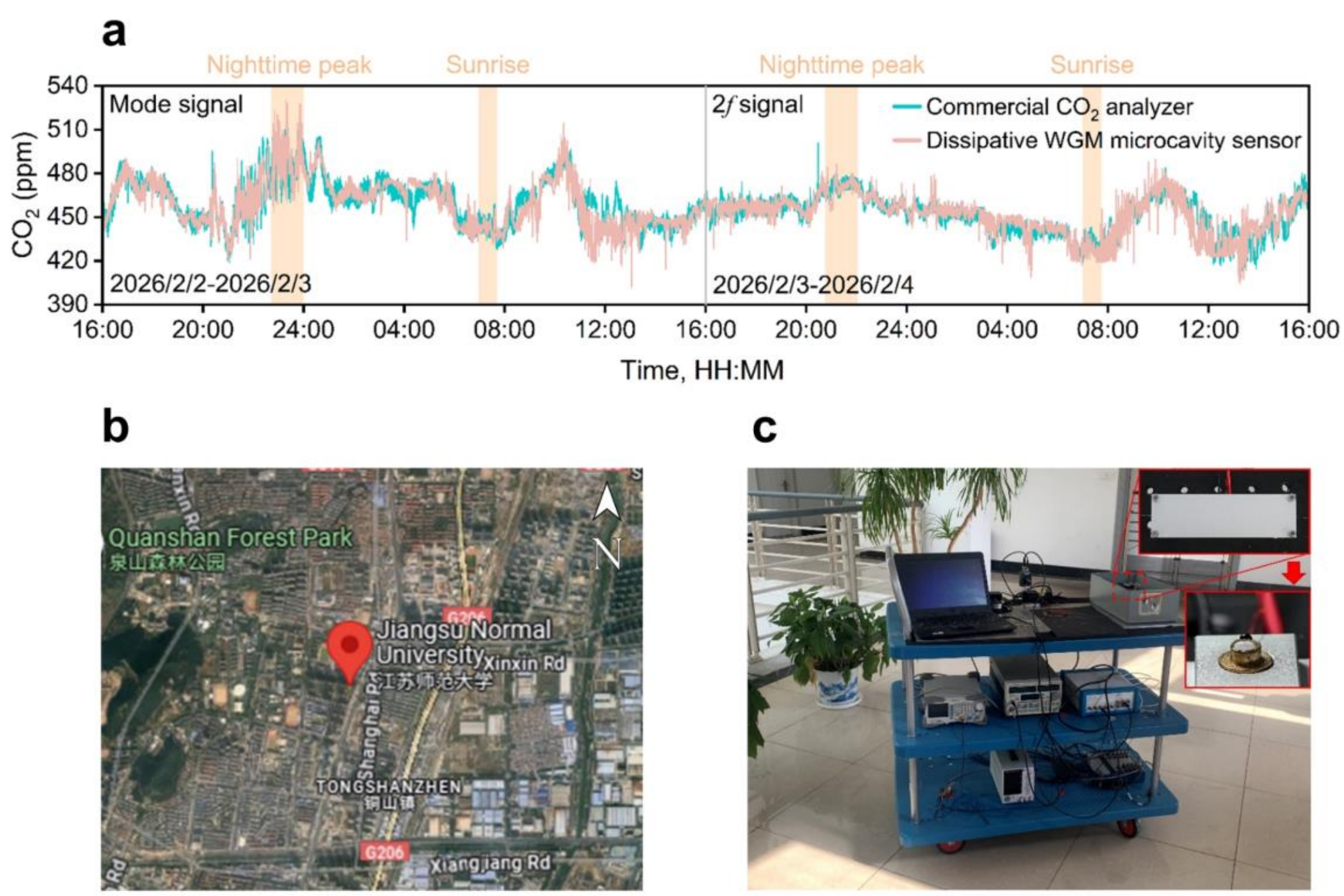

Fig.6 | Continuous monitoring of CO_2 in the actual atmosphere. a, Comparative time series of

ambient CO_2 concentration over ~48 h, measured simultaneously by the dissipative WGM-based sensor (raw optical mode and $2f$ signal) and a calibrated commercial reference analyzer. **b**, Detailed locations of dissipative WGM-based CO_2 sensor measurement site and its surroundings in Xuzhou. Map provided by Google Earth. **c**, The whole CO_2 gas sensing system on a mobile cart. The inset shows the packaged coupling system of the microsphere microcavity and the tapered optical fiber.
on Page 18

We have added relevant discussion in the revised manuscript as:

"To evaluate the practical applicability of the system, long-term ambient CO_2 monitoring was conducted on the main campus of Jiangsu Normal University (JSNU, 34.19°N, 117.18°E, 10 m above ground level (a.g.l.)), as shown in Fig. 6b. The dissipative WGM-based sensor was deployed alongside the calibrated commercial CO_2 analyzer (GMM-1000) to perform simultaneous and parallel measurements. Both instruments sampled ambient air at the same location. The dissipative WGM-based CO_2 sensor was implemented on a compact mobile cart, as shown in Fig. 6c. In this stage of the study, commercial instruments-including the laser controller, lock-in amplifier, function generator and photodetector-were employed to provide a stable platform for validating the sensing mechanism and benchmarking system performance under well-controlled laboratory conditions. The use of discrete instruments was intended for performance evaluation rather than representing the prototype architecture of the system. The core functionalities can be consolidated through custom-designed electronics and packaged optical components, enabling substantial miniaturization and improved portability. A prototype implementation of the integrated system is described in Supplementary Note 8. For real-time ambient monitoring, the encapsulated devices of microspheres and tapered fibers are directly placed in the natural environment. This microcavity enclosure has not undergone airtight encapsulation, allowing ambient CO_2 to diffuse into the enclosure through the fiber feedthrough openings.

Fig. 6a displays 24 h of CO_2 measurements obtained from the raw optical mode, the $2f$ signals and the calibrated commercial analyzer. All three datasets exhibit a pronounced diurnal variation characteristic of a campus environment. From 00:00 to 06:00, CO_2 concentrations increase gradually due to continuous plant respiration, minimal human activity, and relatively weak atmospheric mixing. A more rapid rise is observed between 06:00 and 08:00, corresponding to the onset of morning activities. Between 08:00 and 15:00, enhanced solar radiation strengthens photosynthetic uptake, leading to a progressive decline in CO_2 concentration and a daytime minimum around noon. After 15:00, as solar intensity decreases, photosynthetic activity weakens

and CO_2 levels begin to rise again. From 18:00 to 24:00, the combined effects of ceased photosynthesis, ongoing plant respiration, human presence, and traffic emissions produce a distinct nighttime peak. Two independent monitoring campaigns were conducted. In both cases, the raw optical mode and 2*f* signals from the dissipative WGM-based sensor were benchmarked against the calibrated commercial analyzer. The measured time series exhibit highly consistent temporal trends and concentration levels across all instruments, demonstrating the reliability, repeatability, and field applicability of the proposed dissipative WGM-based CO_2 sensing system." on Page 17.

Comment 13: *The authors should comment on the influence of humidity. High-Q microcavities are known to be sensitive to adsorbed water, which can change the resonance.*

Answer 13: We thank the reviewer for raising this crucial question. We strongly agree that environmental humidity is one of the important factors affecting the practical performance of ultra-high Q-factor WGM microcavities. Under long-term use, the adsorption of water molecules on the microcavity surface does introduce additional optical absorption loss, leading to additional optical absorption and a reduction in the Q-factor.

To address this issue, we implemented targeted packaging measures during the experimental phase (see **Supplementary Note 4 and the answer to Comment 15**). We used a packaging structure based on an aluminum microcavity housing, which incorporates silica gel particles near the ventilation holes at both ends to absorb water vapor entering the microcavity housing from the external environment. This effectively isolates the microcavity from external humidity while ensuring effective circulation of the gas being tested.

On the other hand, the Q-factor of WGM microcavities is influenced by multiple factors. The Q-factor of the microsphere WGM cavity used in our experiment is approximately 10^6. The limiting factors of the Q-factor mainly come from coupling losses of the tapered fiber, scattering losses on the microcavity surface, etc., which far outweigh the absorption of water molecules. Therefore, the attenuating effect of water molecule absorption on the Q-factor only appears during long-term use, and the decay period of the microcavity Q-factor is much shorter than that of WGM microcavities with extremely high Q-factors ($>10^8$).

We conducted a dedicated, **50-day continuous stability assessment** of a non-functionalized WGM microsphere under the controlled environment (constant temperature, dry actual atmospheric composition). The resonance depth and linewidth (proportional to Q^{-1}) were

monitored continuously.

As shown in Fig. S19, for the 50 days, the sensor demonstrated exceptional stability. The resonance linewidth remained stable within ±1.5%, and the resonance depth showed minimal variation (<1%). The Allan deviation of the frequency signal reached a minimum, indicating that the system noise was dominated by fundamental thermorefractive noise, not by drift. This period represents the sensor's optimal performance window.

The 50-day high-stability window validates the effectiveness of our environmental control, while the possible long-term degradation highlights the long-term challenges posed by these intrinsic material and mechanical processes. We have updated the manuscript to include a summary of this 50-day stability experiment.

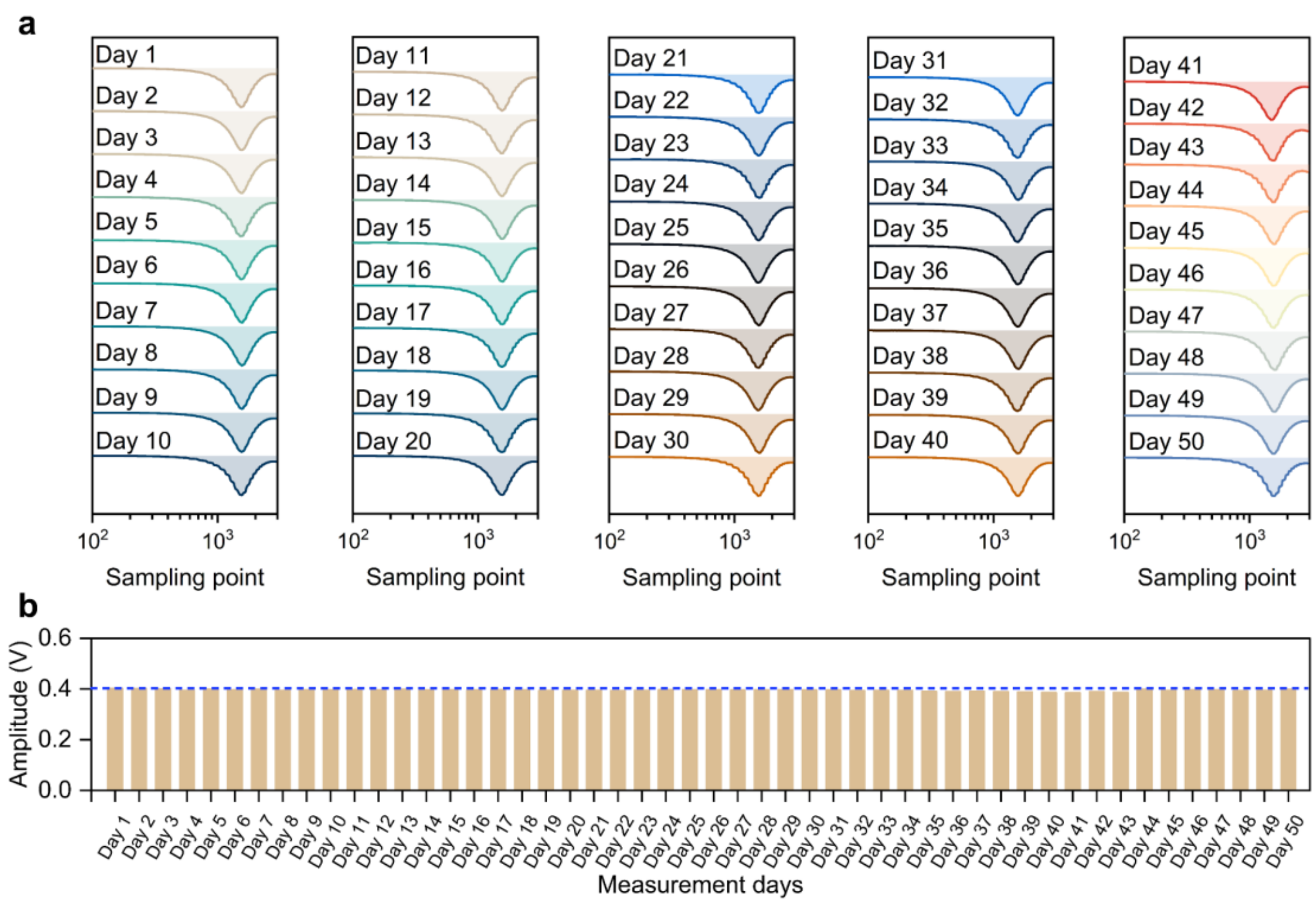

Fig. S19 | a, Mode signals measured within 50 days. b, Analysis of amplitude of mode signal.

We have added relevant discussion in Supplementary Note 4 the revised Supplementary materials as:

"This microcavity enclosure has not undergone airtight encapsulation, allowing ambient CO_2 to diffuse into the enclosure through the fiber feedthrough openings. To improve the long-term operating time of the sensor, desiccant particles with a nanoporous surface structure are placed inside the microcavity enclosure to absorb water vapor from the external environment, while

exhibiting negligible adsorption toward CO_2 under the operating conditions. Therefore, it can maintain long-term dryness inside the microcavity enclosure, reducing the rate of decrease in the Q factor of the microsphere WGM microcavity." on Page 21.

Comment 14: *What is the basis for reporting LoDs at 424 seconds? Moreover, you cannot take a minimum of a noisy Allan plot and claim it is your LoD (0.12 ppb at 424 s of averaging. Will it become 0.5 ppb at 500 seconds?).*

Answer 14: Thank you for this important comment regarding the interpretation of the Allan deviation analysis.

For the sake of accuracy and rigor in the Allan deviation analysis, the previously used Allan deviation data are no longer employed here. Instead, a newly collected Allan deviation signal has been acquired, and a more comprehensive detection limit analysis has been conducted. As shown in Fig.5c and 5d, we employed the Allan deviation plot to analyze the system's noise and drift behavior over extended measurement times. At short integration time (below 400 s), the Allan deviation decreases as the integration time increases with a fitted slope of approximately -0.5, indicating that white noise domintets, which mainly emanates from relative intensity noise of the probe laser and electronic noise of the electrical circuits. At longer time over 400 s, the Allan deviation decreases with fluctuations, which is a signature of system drift. Major drift sources include external temperature minor variation and light power fluctuations that vary slowly over time. In addition, to accurately estimate the minimum detection limit (MDL, i.e. LOD) of sensor, we recalculated the Allan deviation plot by averaging the data through a 50-point window smoothing. As shown in Fig. 5, an Allan deviation analysis of the raw optical mode signal in Fig. 5c indicates a $Hz^{-1/2}$ performance with a bandwidth-normalized sensitivity of 5243 ppt*$Hz^{-1/2}$, which remains until reaching an averaging time of 400 s. At this time, the highest CO_2 sensitivity of ~269 ppt was achieved. Based on the characteristic of white noise, the theoretically predicted detection limit under pure white noise at 400 s is calculated as $5243 / 400^{1/2} \approx 262.15$ ppt. This theoretical value closely matches the experimentally measured and smoothed optimal detection limit of 269 ppt, thereby confirming the validity of the Allan deviation analysis. Similarly, an Allan deviation analysis of 2*f* signal in Fig. 5d indicates a $Hz^{-1/2}$ performance with a bandwidth-normalized sensitivity of 2926 ppt*$Hz^{-1/2}$, which remains until reaching an averaging time of 400 s. At this time, the highest CO_2 sensitivity of ~168 ppt was achieved. The theoretically predicted

detection limit under pure white noise at 400 s is calculated as 2926 /$400^{1/2}$ ≈ 146.3 ppt. This theoretical value closely matches the experimentally measured and smoothed optimal detection limit of 168 ppt, also confirming the validity of the Allan deviation analysis. [Ref: *Nat. Commun.* **16**, 10549 (2025)]

We have added relevant discussion in the revised manuscript as:

"To evaluate the stability of the WGM microcavity–based sensor, continuous measurements a 1.5 ppm CO_2/N_2 mixture were performed over a period of approximately 66 min with a temporal resolution of 4 s, as shown in Figs. 5a and 5b. The corresponding raw optical mode and 2*f* signals are partially presented in Fig. S9 of the supplementary material. The measurement precision was analyzed separately for the raw optical mode and the 2*f* signal. For the raw optical mode (Fig. 5a), the histogram of the retrieved concentrations follows a normal distribution centered around the mean value. Gaussian fitting yields an R^2 value of 0.983. The precision, defined as the standard deviation (1σ) of the distribution, is 7.51 ppb, corresponding to 8.8 ppb in terms of the half width at half maximum (HWHM). For the 2*f* signal (Fig. 5b), the histogram also exhibits a normal distribution, with a Gaussian fit R^2 of 0.987. The corresponding precision is significantly improved to 2.28 ppb (1σ) and 2.7 ppb (HWHM). Compared with the raw optical mode, the modulation-demodulation technique effectively suppresses low-frequency noise, resulting in a ~3.3-fold improvement in measurement precision compared to the raw optical mode.

The minimum detection limit (MDL, *i.e.* LOD) was further quantified through Allan deviation analysis. To ensure a reliable estimation, the time-series data were preprocessed with a 50-point moving-average window prior to calculating the Allan deviation. For the raw optical mode (Fig. 5c), the Allan deviation exhibits a $\tau^{-1/2}$ dependence, indicating white-noise-limited performance, with a bandwidth-normalized sensitivity of 5243 ppt $Hz^{-1/2}$. This behavior persists up to an averaging time of 400 s, at which a MDL of approximately 269 ppt is achieved. For the 2*f* signal (Fig. 5d), a similar $\tau^{-1/2}$ trend is observed, with an improved bandwidth-normalized sensitivity of 2926 ppt $Hz^{-1/2}$. At an averaging time of 400 s, the MDL reaches approximately 168 ppt, confirming the superior noise performance of the 2*f*-based measurement. To verify the reliability of the Allan-derived detection limits, additional measurements were performed on CO_2/N_2 mixtures with concentrations of 0.2 ppb and 0.5 ppb, prepared through multiple dilution steps, using an integration time of 400 s. The corresponding raw optical mode and 2*f* responses are shown in Figs. 5e and 5f, respectively. The magnified insets reveal clear signal separation between the two concentration levels, with well-resolved features above the intrinsic noise floor. These results demonstrate that the sensor maintains high sensitivity and stable performance even near its

detection limit." on Page 14.

We have revised Fig. 5 in the revised manuscript as:

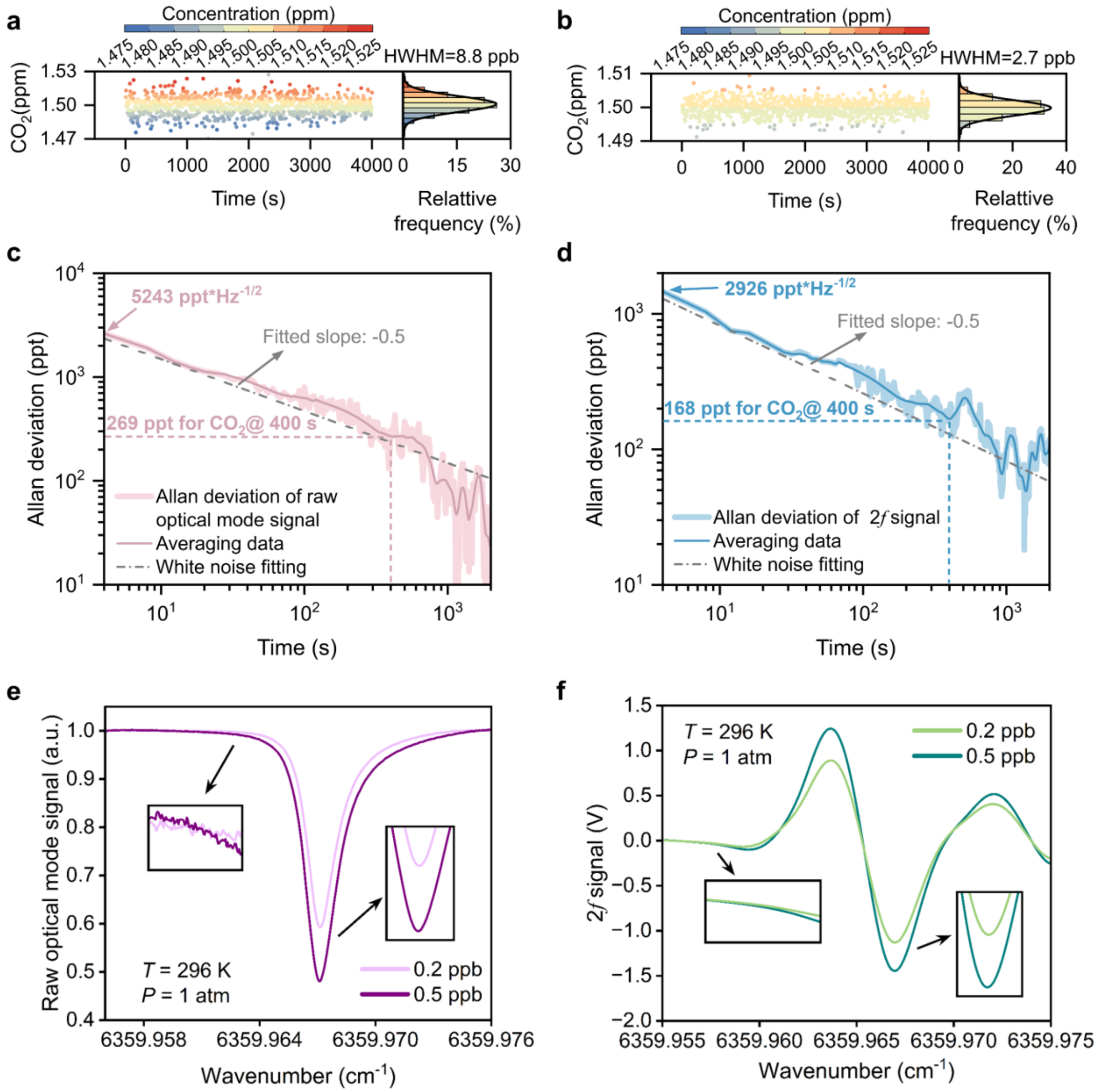

Fig.5 | Sensor stability and sub-ppb detection capability. a and b, Distribution of CO_2 concentration measured continuously for 4000 s for 1.5 ppm CO_2-N_2 sample using raw optical mode and 2*f* signal (*T*=296 K, *P*=1 atm), as well as the frequency distribution and Gaussian profile fitting of measured concentration. **c** and **d**, Allan deviation plot of the dissipative WGM gas sensor based on the data shown in Figs. 5a and 5b. **e** and **f,** Raw optical mode signals and 2*f* signals for CO_2-N_2 mixtures at 0.2 ppb and 0.5 ppb concentrations with 400 s integration time. on Page 16.

Comment 15: *3. Practical and Engineering Considerations*

To support the claims of robustness and long-term stability, the following aspects should be clarified:

- A clear depiction of the mechanical assembly—how the cavity and tapered fibre are mounted—is necessary. The current illustrations do not convey the stability mechanism.

Answer 15: We fully agree with the reviewer's point that the robustness and long-term stability of the device are crucial for its practical application. To address this challenge, we integrated the sensor into a specially designed packaging module (as shown in Fig. **R9**). This packaging design ensures excellent device performance in complex environments through system engineering in the following three aspects:

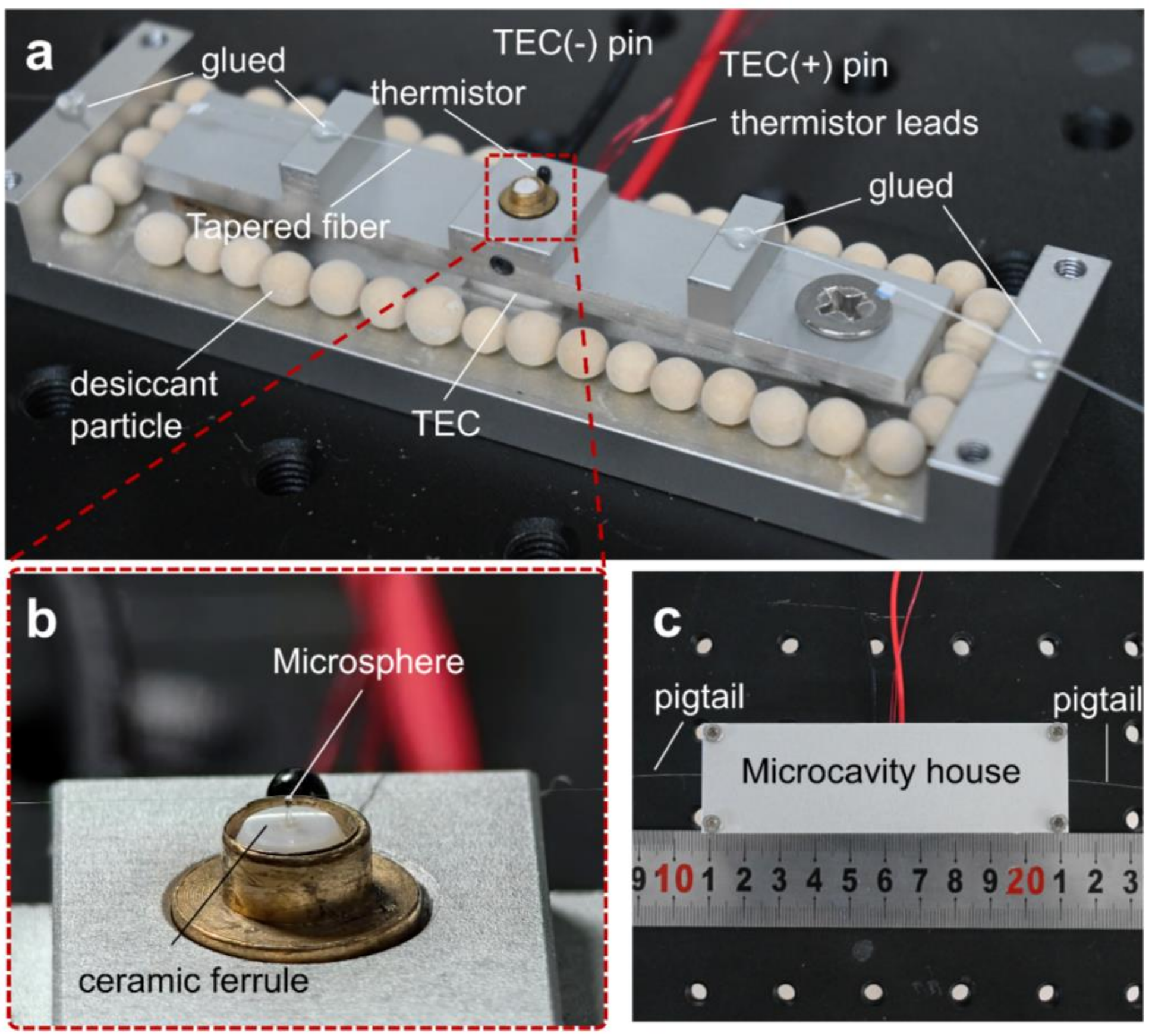

Fig. R9. a, Packaging structure of tapered fiber coupled with microsphere WGM microcavity. **b**, Zoom of the black dashed square in **a**. **c**, Photograph of the fully packaged sensor device.

(a) Mechanical Stability: Our WGM microcavity is fabricated directly on the end face of a standard single-mode optical fiber, with the fiber itself serving as the natural supporting cantilever for the microsphere. Considering that the slender structure of the single-mode fiber is susceptible to airflow disturbances and acoustic waves in the air, leading to mechanical vibrations, we adopted multi-layer reinforcement measures. First, the fiber is encapsulated in a standard ceramic ferrule, exposing only the microsphere and a very short section of the fiber, significantly reducing the cantilever length to improve mechanical rigidity. Second, the ceramic ferrule is tightly embedded in a brass sleeve and finally mounted on a sturdy aluminum bracket. For coupling, we used a precision translation stage to pre-align the tapered fiber with the microsphere. After establishing the optimal coupling point, we fixed it to the aluminum bracket using UV-curing adhesive. We set multiple stress release points along the fiber axis to attenuate externally transmitted vibrations. Finally, the entire assembly is sealed in an aluminum alloy shielding box (Microcavity house), effectively isolating external acoustic noise and airflow interference.

We have added the relevant discussion has also been added to revised manuscript.

"Our device-packaged sensor incorporates a dual-stage temperature control system. This system effectively mitigates the impact of temperature fluctuations in the ambient environment on the sensor's readouts (see Supplementary Note 4). Within the thermally stabilized microcavity house, during sensor operation, the heat generated as the laser frequency scans across the WGM resonance is mainly localized to the region surrounding the microcavity and the tapered fiber. Consequently, the WGM coupling strength primarily responds to this localized temperature changing, demonstrating low susceptibility to temperature variations occurring in the broader surroundings. Additionally, some desiccants are placed inside the microcavity house to filter out water vapor from the environment, thereby ensuring the long-term stability and working life of the sensor." On page 18.

We have added Fig. **R9** as Fig. **S10** to the revised supplementary materials, and the relevant discussion has also been added to Supplementary Note 4.

"To enhance the robustness of the WGM microcavity sensor, we fabricated a device package for the microsphere cavity with the tapered fiber, as shown in Fig. S10. First, the microsphere stem was inserted into a standard single-mode fiber ceramic ferrule, with only a small portion of the stem and microsphere exposed. This effectively suppressed forced vibrations of the microsphere caused by external forces, improving the mechanical stability of the microsphere microcavity.

The entire ceramic ferrule was tightly encased in a copper sleeve, with thermal grease filling the gaps between them. The copper sleeve was then mounted onto an aluminum bracket. A

thermistor was attached to one side of the copper sleeve for first-stage of temperature control. The first stage of temperature control primarily acts on the small volume near the microsphere, resulting in better local temperature stability. After optimizing the coupling position of the tapered fiber to the microcavity, the tapered fiber was fixed to the aluminum base with low-thermal-expansion UV glue. Multiple glue points were used along the fiber axis to dampen the effects of external vibrations on the coupling region. At this point, the microcavity and tapered fiber form a single integrated structure, greatly improving the mechanical robustness of the coupling. Finally, the aluminum bracket was placed in an aluminum box (microcavity enclosure), with the pigtails at both ends of the tapered fiber extending out from the sides. The entire aluminum box undergoes independent second-stage temperature control. A precision PID servo controller ensured that the internal air temperature of the aluminum box remained stable with an extremely low fluctuation range.

This microcavity enclosure has not undergone airtight encapsulation, allowing ambient CO_2 to diffuse into the enclosure through the fiber feedthrough openings. To improve the long-term operating time of the sensor, desiccant particles with a nanoporous surface structure are placed inside the microcavity enclosure to absorb water vapor from the external environment, while exhibiting negligible adsorption toward CO_2 under the operating conditions. Therefore, it can maintain long-term dryness inside the microcavity enclosure, reducing the rate of decrease in the Q factor of the microsphere WGM microcavity.” On page 21.

(b) Temperature Control: To minimize the impact of ambient temperature fluctuations on sensor readings, we employed a two-stage active temperature control strategy. The first stage of temperature control acts on the brass sleeve, using a thermistor to monitor and feedback control the local temperature in real time; a TEC cooling plate is attached to the bottom of the aluminum bracket with thermal grease, and the aluminum alloy shielding box acts as a heat sink in contact with the hot side of the TEC. Since the rest of the aluminum bracket is not in direct contact with the aluminum alloy shielding box, this temperature control strategy can target a smaller volume, thus achieving better local temperature stability.

The second stage of temperature control targets the overall aluminum casing, ensuring that the air temperature inside the microcavity chamber is in thermal equilibrium by controlling the ambient temperature. Through closed-loop control of a high-precision PID servo control system, we successfully controlled the temperature around the microcavity within a very small fluctuation range, thus ensuring extremely high spectral stability.

(c) Humidity Control: To mitigate the influence of ambient humidity variations, we maintain a controlled dry atmosphere inside the sealed aluminum enclosure. The fiber feedthrough ports, necessary for optical access, are equipped with desiccant cartridges filled with silica gel. These cartridges act as selective filters, passively absorbing incoming water vapor from the external environment while allowing CO_2 to pass through with minimal interaction. This design ensures a stable, low-humidity internal environment. The effectiveness of this humidity control is experimentally validated, as shown in Fig. S18. The measured spectral drift of the cavity mode remains negligible when the external relative humidity is deliberately varied over a significant range, confirming that the internal conditions are effectively decoupled from external hygroscopic fluctuations. We have added Fig. S18 to the revised supplementary materials, and the relevant discussion has also been added to Supplementary Note 9.

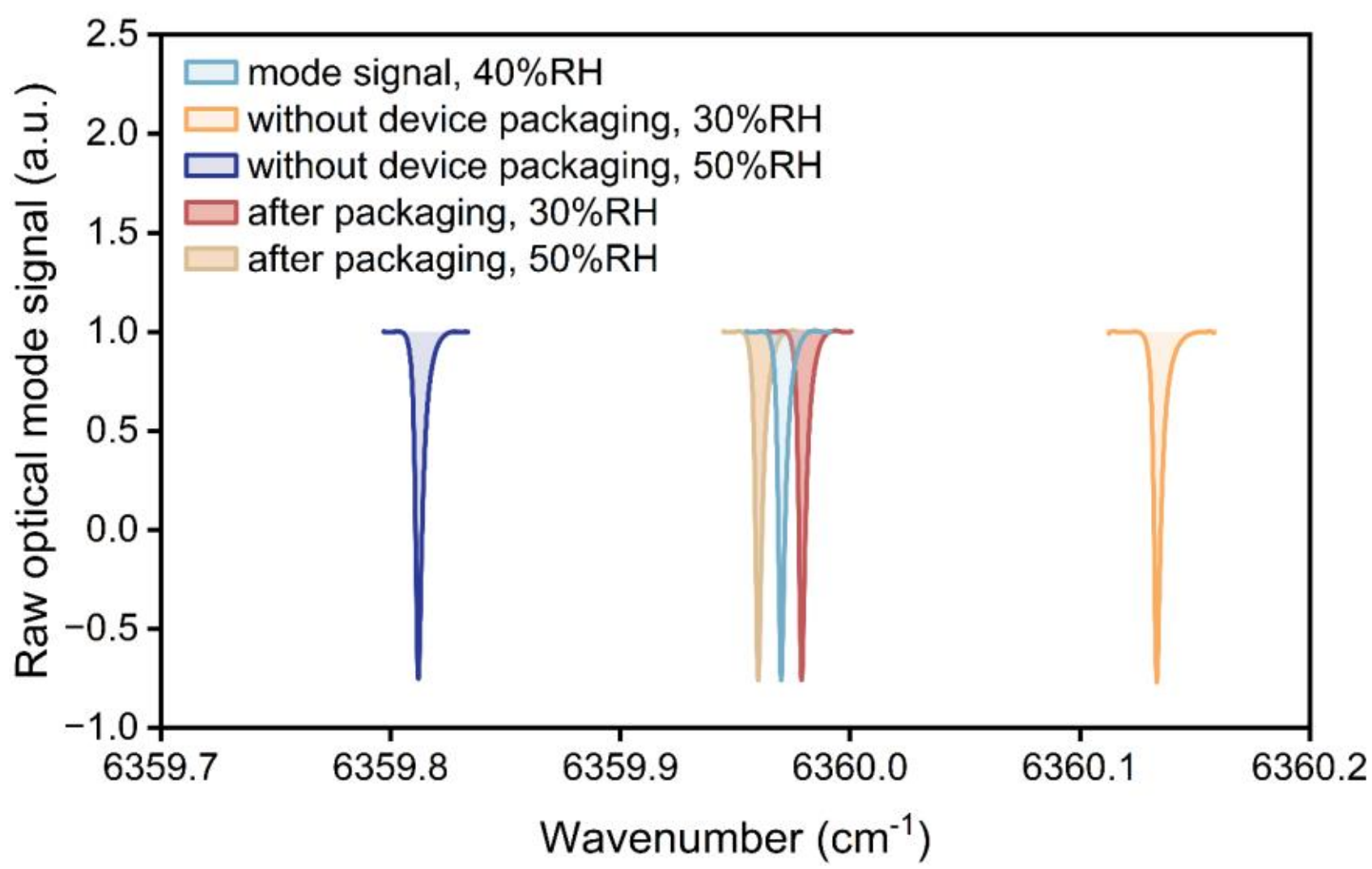

Fig. S18 | Variations of raw optical mode signal under different relative humidity.

On page 38.

"Although the inherent surface hydrophobicity reduces the impact on humidity changes, humidity-induced resonance drift remains a non-negligible noise source for high-precision gas sensing, affecting baseline stability and long-term reliability. To address this, the coupling structure consisting of the tapered fiber and the microsphere WGM microcavity is packaged (detailed in **Supplementary Note 4**). This packaging achieves two critical functions:

This device maintains a constant, dry internal environment around the micro resonator, effectively decoupling it from external humidity fluctuations and particulate contamination, thereby ensuring stability. It allows controlled introduction of analyte gases through a dedicated

sampling port. The incoming gas stream passes through desiccant particles before entering the sensing chamber, ensuring a stable and dry local environment for accurate measurements.

Under this device packaging, the mode drift was measured when the external relative humidity changed from 30% to 50%. As shown in Fig. S18, under the condition of relative humidity fluctuation of $\pm$10% RH, the resonance wave number shift of the WGM was only about $\pm$0.01 cm^{-1}, and the mode remained within the effective absorption linewidth, which did not affect the measurement of CO_2." On page 37.

Comment 16: *How challenging is it to identify or fabricate a cavity whose resonance aligns with the chosen absorption line? Can this be tuned, and if so, by how much?*

Answer 16: The silica microsphere WGM cavity used in our experiments inherently supports a high density of optical modes. This is due to its ability to sustain multiple families of transverse modes (e.g., fundamental and higher-order radial modes), each characterized by a different effective refractive index and resonant wavelength. Consequently, within the spectral bandwidth of a typical gas absorption line, there exists a dense distribution of cavity resonance modes. The individual resonant linewidths are significantly finer than the spacing between adjacent modes, ensuring multiple discrete resonances are available across the broader absorption profile.

A key feature of our platform is the ability to selectively excite specific WGMs through precise control of the coupling between the tapered fiber and the microsphere. **As detailed in Supplementary Note 1.2**, the excitation of microcavity modes depends on the mode field overlap ratio. Different transverse modes exhibit unique field distributions. By precisely adjusting the lateral and axial position of the tapered fiber relative to the microsphere, we can dramatically alter this overlap, thereby exciting the mode we need. This tunability is demonstrated experimentally in Fig. R10, which shows that changing the coupling position readily accesses different resonator modes within the same spectral window.

The practical simplicity of achieving resonance alignment is fundamentally enabled by the significant scale difference between the key spectral features, as illustrated in Fig. 1d of the manuscript. The full width at half-maximum (FWHM) of the target CO_2 absorption line at 1573.2 nm is substantially broader than the individual WGM linewidth. This relationship guarantees that the broad absorption envelope inherently includes several cavity resonances. Therefore, the experimental task is selecting one from the readily available dense mode spectrum, a resonance

that falls suitably within the absorption peak. This selection is efficiently accomplished in real-time by monitoring the transmission spectrum while finely adjusting the fiber-cavity coupling position.

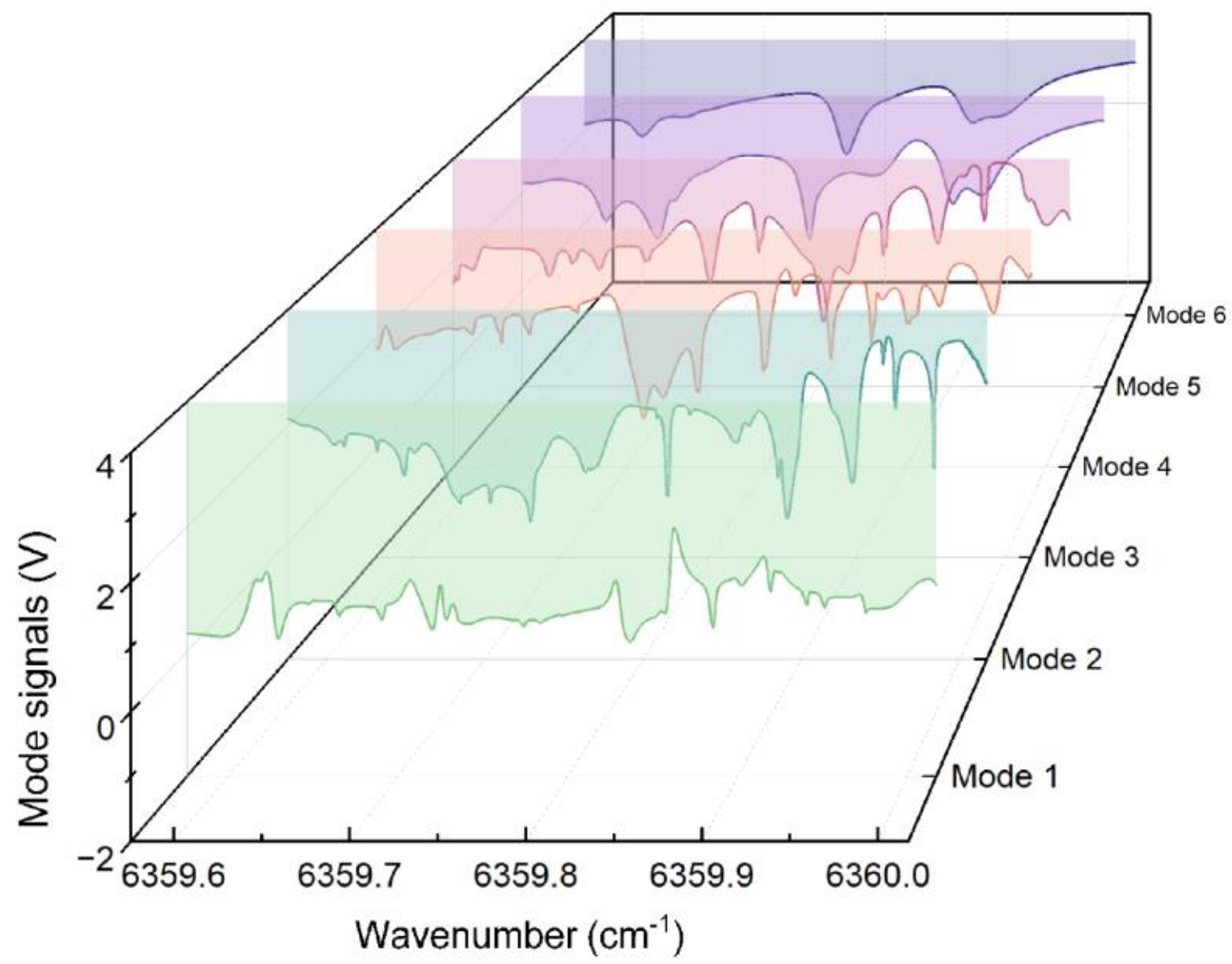

Fig. R10. WGM microcavity modes excited at different coupling positions

We have added relevant discussion in the revised manuscript as:

"Given that the absorption spectrum linewidth is much larger than the mode linewidth, and the microsphere cavity can support numerous modes within a certain spectral width, the coupling can be optimized in the experiment to excite the best mode for sensing applications." on Page 8.

Comment 17: *More detail on the gas-mixing system is needed. What system was used, and how accurately can concentrations at the 1.5 ppb level be generated and maintained?*

Answer 17: Thank you for raising this important comment. We fully agree that a clear description of the gas preparation procedure is essential for evaluating the sensor performance at ultra-low concentrations such as 1.5 ppb. The gas-generation methodology used in this work combines precise dynamic blending with a controlled iterative dilution process to reliably produce trace-level CO_2 mixtures.

17.1 Gas-Mixing System and Limits of Direct Blending

The gas-mixing system used in this study is an Environics Series 4000 multicomponent gas blender equipped with three calibrated mass-flow controllers (MFCs), as illustrated in Fig. R11. The source

gases were certified 1% CO_2 and 99.9999% N_2 (Xuzhou Special Gas). Prior to use, the N_2 diluent was passed through a purification stage and its background CO_2 concentration was verified to be below 0.2 ppb.

Although the Environics system provides highly accurate dynamic gas blending, direct generation of extremely low concentrations such as 1.5 ppb using continuous flow mixing alone is challenging. This limitation arises mainly from two factors:

Dilution ratio limitation of the MFCs: the minimum controllable flow rate of a typical MFC (~1% of full scale) restricts the maximum achievable dilution ratio, making direct generation of sub-10 ppb mixtures difficult in a stable manner.

Background contamination of the balance gas: even ultra-high-purity nitrogen may contain trace CO_2 impurities that become significant when attempting to generate concentrations at the ppb level.

For these reasons, direct continuous blending was used only to generate an intermediate concentration within the optimal operating range of the system.

17.2 Stepwise Method for Generating 1.5 ppb CO_2

To achieve the target concentration of 1.5 ppb, a controlled two-stage dilution strategy was implemented using the gas blender and a stainless-steel mixing tank.

Step 1: Generation of an intermediate concentration:

First, the Environics system dynamically blended the 1% CO_2 standard gas with purified N_2 to produce a stable intermediate mixture at the ppb level (typically around 150 ppb). This mixture was introduced into a stainless-steel mixing tank where sufficient residence time ensured complete gas homogenization.

Step 2: Iterative dilution in the mixing tank:

The concentration was then reduced using an automated evacuate-and-backfill dilution cycle. During each cycle, approximately 90% of the gas volume in the mixing tank was evacuated and immediately replaced with purified N_2. According to the gas dilution law, the CO_2 concentration decreases to 10% of its previous value after each cycle. By controlling the number of dilution cycles, the concentration could be precisely reduced to the target value of 1.5 ppb.

The advantage of this method is that the final concentration depends primarily on the accurately controlled volume exchange ratio, rather than on extremely small dynamic flow rates from the MFCs.

17.3 Concentration Accuracy, Stability, and Independent Verification

The accuracy of the generated gas concentration is traceable to three factors:

(1) the certified concentration of the primary standard gas,

(2) the calibrated mass-flow controllers of the Environics system used to generate the intermediate mixture, and

(3) the precisely controlled volume exchange during the dilution cycles.

Based on these factors, the combined expanded uncertainty (k=2) of the prepared concentrations in the ppb range is estimated to be better than ±0.3 ppb.

To independently verify the gas concentration, the final CO_2 mixture in the mixing tank was measured using a high-precision mid-infrared Off-Axis Integrated Cavity Output Spectroscopy (OA-ICOS) analyzer (GMM-1000), which provides a measurement precision better than 0.1 ppb. After verification, the prepared gas was delivered from the mixing tank to the sealed sensing chamber for sensor measurements.

In summary, by combining the precise dynamic blending capability of the Environics Series 4000 system with a controlled iterative dilution technique in a mixing tank, we were able to reliably generate and verify a stable 1.5 ppb CO_2 test environment. This approach effectively overcomes the limitations of direct dynamic dilution and provides a well-controlled gas environment for evaluating the sensor performance at ultra-low concentrations.

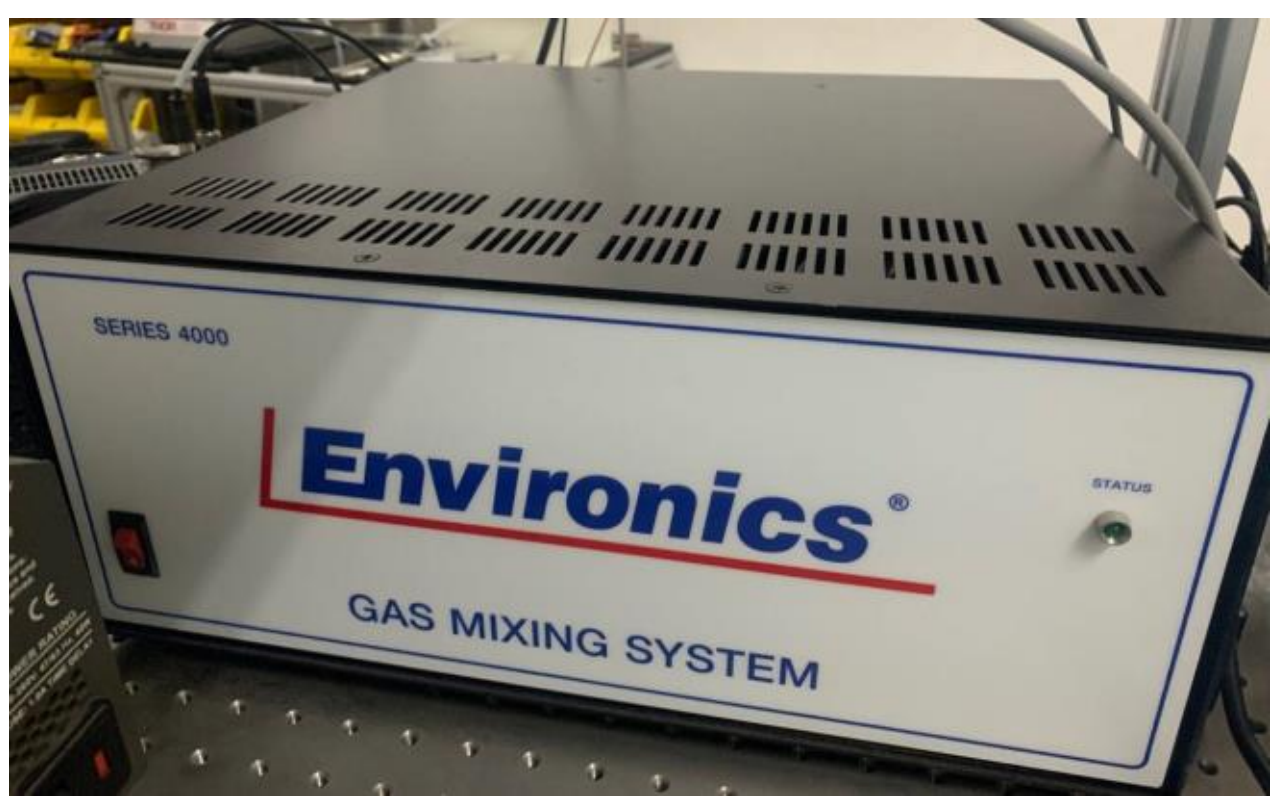

Fig. R11. Precision three-port mass-flow controller (Environics, Series 4000).

We have added relevant discussion in the revised supplementary information (**Supplementary Note 2)** as:

"**2.2.3 Gas-mixing system**

Gas samples were delivered from high-purity cylinders containing 1 % CO_2 and 99.9999 % N_2 (Xuzhou Special Gas) through stainless-steel tubing to a multicomponent gas blender (Environics S4000) integrated with a stainless-steel mixing tank. The blender precisely regulated

gas flows to achieve complete homogenization within the tank, producing accurate and reproducible concentrations in the 0–500 ppm range. Prior to each measurement, the sealed chamber was evacuated to ~100 Pa using the dry rotary-vane pump and then filled with the prepared standard gas at 1 atm. The chamber outlet was connected to a catalytic converter and activated-carbon scrubber before venting, ensuring safe handling of exhaust gases. This configuration enables controlled introduction of different gas mixtures into the chamber while preserving the integrity of the sealed optical setup, ensuring high reproducibility and reliability of CO_2 sensing experiments.

2.2.4 Sub-ppb CO_2 sample generation and calibration

(1) Preparation of Dilution Gas

Before generating ultra-low-concentration mixtures, the mixing tank and gas lines were repeatedly purged with high-purity N_2 and evacuated to remove residual gases. This procedure minimized background contamination within the system. The background CO_2 concentration of the purified N_2 was verified to be below 0.2 ppb using a mid-infrared gas analyzer prior to the dilution process, ensuring negligible contribution from the diluent gas during sub-ppb mixture preparation.

(2) Iterative Dilution Procedure

Sub-ppb CO_2 mixtures were generated using a controlled multi-step dilution process in the mixing tank.

First, the multicomponent gas blender introduced proportional flows of 1 % CO_2 standard gas and purified N_2 into the tank to produce an intermediate mixture at the ppb level (typically ~150 ppb). The gas mixture was allowed to equilibrate inside the tank to ensure complete homogenization.

Subsequently, an automated evacuate-and-backfill cycle was applied. During each cycle, approximately 90 % of the tank volume was evacuated and immediately replaced with purified N_2. According to the gas dilution law, the CO_2 concentration decreases to 10 % of its previous value after each cycle. The number of dilution cycles required to reach the target concentration was calculated from the initial intermediate concentration.

Through this iterative dilution procedure, stable and reproducible sub-ppb CO_2 mixtures were obtained.

(3) Concentration Verification and Gas Delivery

The final CO_2 concentration in the mixing tank was verified using a high-precision mid-infrared Off-Axis Integrated Cavity Output Spectroscopy (OA-ICOS) analyzer (GMM-1000),

which provides a measurement precision better than 0.1 ppb.

After verification, the prepared gas mixture was delivered from the mixing tank to the sealed sensing chamber through stainless-steel tubing. The gas flow was regulated by the multicomponent gas blender to ensure stable exposure conditions during sensing measurements. The accuracy of the prepared concentrations is traceable to the certified standard gas concentration, the calibrated flow-control system of the gas blender, and the precisely controlled volume exchange during the dilution cycles. The combined expanded uncertainty (k=2) of the prepared concentrations in the ppb range is estimated to be better than ±0.3 ppb.

The lowest concentration tested in this work (~0.2 ppb) approaches the detection limit of the sensing system and is therefore used primarily to evaluate the sensor response near the limit of detection." on Page 16.

Comment 18: *4. Moderation of Claims*

Line 54: The manuscript states that frequency locking is "typical" in TDLAS. This is incorrect; frequency locking is not commonly used in standard direct or WMS TDLAS configurations and is typically employed only in advanced cavity-enhanced systems.

Answer 18: As you rightly pointed out, TDLAS generally does not employ frequency locking, since scanning the wavelength is essential to capture the absorption lines of the target molecules. Frequency locking is typically employed in applications requiring long-term stability to mitigate laser wavelength drift. This technique requires sophisticated electronic circuits, along with a frequency reference setup or advanced digital frequency-locking algorithms, and is therefore mostly utilized in advanced cavity-enhanced systems. Accordingly, we have revised the description in the original manuscript.

We have removed the previous statement and added the sentence:

"Moreover, advanced gas detection systems based on absorption spectroscopy require frequency locking loops to enhance detection performance, often relying on standard molecular absorption lines or high-finesse Fabry-Pérot (FP) cavities as frequency references." on Page 3.

Thanks to the reviewer's suggestions, the current statement is more precise.

Comment 19: *Frequency fluctuations - even in this setup - will still contribute to measurement uncertainty, as the molecular absorption line is not infinitely broad compared with the microcavity resonance.*

Answer 19: Thank you for raising the important point regarding frequency fluctuations and their potential contribution to measurement uncertainty. We want to clarify the relationship between our cavity resonance linewidth, the molecular absorption linewidth, and the observed frequency stability, which together ensure the robustness of our dissipative sensing approach.

As noted in your comment, the finite linewidth of the absorption feature relative to the cavity resonance is indeed a key consideration. In our system, the WGM resonance has an extremely narrow linewidth (~0.001 cm^{-1}), while the CO_2 absorption linewidth under ambient conditions is pressure-broadened to approximately 0.1 cm^{-1}, as shown in supplementary Fig. R12. This means the molecular absorption line is about 100 times broader than the cavity resonance. Consequently, small frequency drifts between the laser and cavity have a substantially reduced impact on the recorded absorption signal, because the absorption cross-section seen by the cavity field changes very slowly over such a small fractional shift in wavelength.

Moreover, we have carefully characterized the long-term frequency stability of our sensor. The fluctuation of the resonance position over several minutes is on the order of $\pm$0.001 cm^{-1}, which is well within the broad envelope of the absorption line and near the peak. During this time period, we have completed the measurement. Crucially, these slow variations do not affect our dissipative readout, because the signal is derived from the resonance depth (transmitted intensity at resonance), not the resonance frequency. The modulation-demodulation scheme further isolates the absorption-induced depth change from low-frequency technical noise, including residual cavity frequency drift.

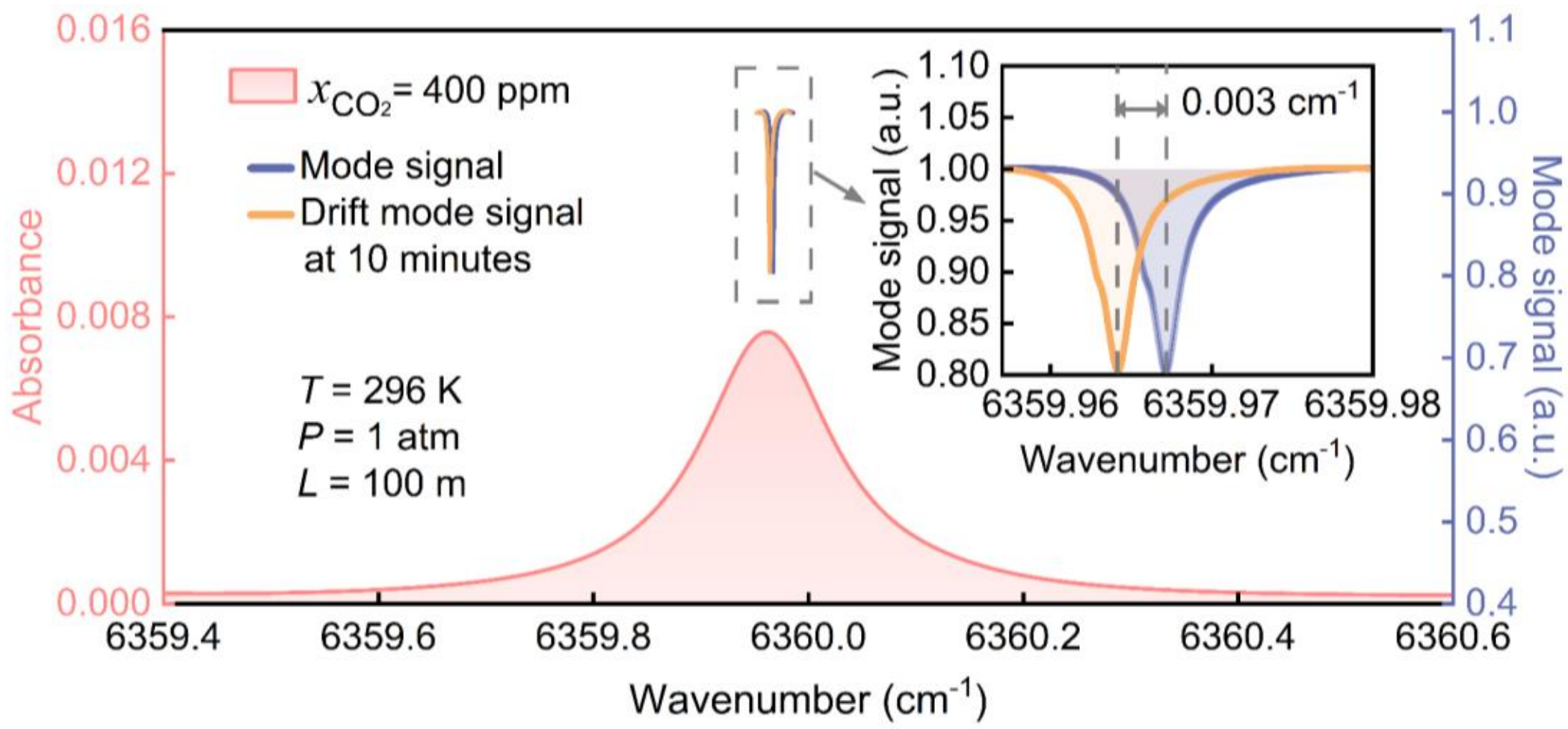

Fig. R12. CO_2 absorption line and one resonance position of the microsphere cavity. Inserted graph: Frequency drift of resonance mode.

Quantitative analysis

In our dissipative sensing scheme, we apply a triangular-wave frequency scan with a span of approximately 1 cm^{-1}, which fully covers the entire CO_2 absorption line (with a linewidth of ~0.1 cm^{-1}). During each scan cycle, we record the complete cavity transmission spectrum, including its depth variation induced by molecular absorption. The short-term cavity frequency drift (the measured value at 10 minutes is approximately ±0.003 cm^{-1}) does not alter the shape or amplitude of the recorded absorption feature within each scan. As long as the resonance mode remains near the peak, the measured absorption-induced depth modulation remains quantitatively consistent, as shown in Fig. R12. Fig. R12 has been added as supplementary Fig. S14 in the supplementary information. For long-term measurements, we can use an electronic frequency locking loop to lock the laser frequency to the microcavity resonance. This technique not only eliminates long-term drift of the laser frequency relative to the microcavity resonance but also allows for real-time acquisition of sensor readings, which is advantageous in analyzing dynamic changes in gas concentration.

We will incorporate this explanation into the revised manuscript to more clearly articulate why frequency drift does not materially affect the uncertainty in our dissipative detection scheme.

We have added relevant discussion in the revised supplementary information (**Supplementary Note 6**) as:

Quantitative Analysis of Frequency Fluctuation Effects

The influence of laser-cavity frequency drift on the measurement uncertainty in the presented dissipative sensing scheme is analyzed quantitatively based on the system's characteristic parameters. The ultra-narrow linewidth of the optical mode of the WGM microcavity, typically ~0.001 cm^{-1}, is contrasted with the pressure-broadened linewidth of the target CO_2 absorption line, which is approximately 0.1 cm^{-1} (FWHM) under ambient conditions, as depicted in Fig.1d in the main text and Fig. S14. This two-orders-of-magnitude difference implies that the absorption cross-section varies only minimally across the scale of the linewidth of the optical mode.

Frequency stability characterization indicates that the locked cavity system exhibits a resonance position drift within a range of approximately ±0.003 cm^{-1} within 10 minutes. The drift magnitude (±0.003 cm^{-1}) remains much smaller than the absorption linewidth (~0.1 cm^{-1}), ensuring that the mode remains within the high-slope region of the absorption profile. The $2f$ signal is extracted via synchronous demodulation at the modulation frequency. This method renders the measurement inherently insensitive to absolute frequency offsets, as it detects the periodic depth modulation synchronized with the scan, not the static resonance wavelength.

The key quantitative consideration is that the frequency drift (±0.003 cm^{-1}) is still near the absorption peak. Therefore, during each scanning cycle, drift does not shift the resonance outside the effective absorption linewidth, thus maintaining the line shape and amplitude of the signal. The dominant noise floor is thus determined by the intensity noise and the efficiency of the demodulation process, not by laser-cavity detuning variations. This analysis confirms that the designed sensing approach effectively reduces the sensitivity to low-frequency technical noise, including long-term cavity frequency fluctuations. On Page 28.

Comment 20: *The comparison in Table 1 is not well balanced. Techniques such as multi-pass cells or cavity-enhanced spectroscopies can readily achieve sub-ppb detection limits in the mid-IR; on-chip waveguide sensors recently reported 20 ppb. The comparison spans disparate spectral ranges, integration times, and instruments optimized for different applications, making the table's implications unclear.*

Answer 20: Thank you for your insightful comment regarding the balance and clarity of Table 1. We appreciate you highlighting the challenge of comparing technologies that operate in disparate spectral ranges, with different integration times, and which are inherently optimized for distinct primary applications.

The primary objective of Table 1 is not to declare superiority in all parameters, but to visually map the inherent trade-offs and design philosophies across a representative spectrum of state-of-the-art CO_2 sensing approaches. We intentionally included this breadth to contextualize our work's specific niche. By presenting parameters like sample cell volume, optical alignment difficulty, estimated system cost, and integration time alongside the LOD, the table aims to illustrate that ultra-low LODs (e.g., from sophisticated multi-pass or cavity-enhanced mid-IR systems) are often achieved at the expense of system complexity, size, cost, and ease of deployment.

In this landscape, our work demonstrates that a near-infrared, dissipative WGM-based sensor can achieve a fundamentally competitive LOD (168 ppt), which is comparable to or even surpasses many systems, including several mid-IR configurations listed. Crucially, it attains this while simultaneously scoring favorably on the practicality metrics most relevant for field-deployable, cost-sensitive, and user-friendly applications: a small sample volume, easy optical alignment, and low estimated system cost. This combination is rare, as highlighted in the table where other techniques typically sacrifice one or more of these attributes for supreme sensitivity.

Regarding the recent progress in on-chip sensors mentioned in your comment, we have taken note of relevant advances such as the tapered sub-wavelength grating slot waveguide sensor reported by Zhang et al. (2023), which achieves a detection limit of 21.73 ppb for N_2O at 7.70 µm. This indeed represents a significant step forward in on-chip mid-infrared gas sensing. Many optical sensing platforms can target different gases by selecting appropriate absorption wavelengths. For instance, in Tunable Diode Laser Absorption Spectroscopy (TDLAS), the target analyte can be altered by switching the laser source to a wavelength corresponding to a different absorption line. Similarly, in dispersive whispering-gallery-mode (WGM) sensors, the selective detection of various gases can be achieved through tailored functionalization of the cavity surface. In line with this general flexibility, our sensor architecture also supports multi-gas sensing by utilizing laser sources at distinct wavelengths matched to the absorption features of different target molecules. A direct, quantitative comparison of sensing performance across different gas species is inherently challenging and often not methodologically sound. This is primarily because the intrinsic absorption strength, linewidth, and spectroscopic profile vary significantly from one gas to another. These fundamental differences make it difficult to establish a unified metric that fairly evaluates sensor performance independently of the molecule-specific photophysical properties. Therefore, to enable a meaningful and equitable benchmark against existing technologies, we have focused our comparative analysis in this work exclusively on CO_2 sensing. This approach allows for a direct evaluation of sensitivity, limit of detection, and stability across different sensor platforms

when addressing the same well-defined molecular transition. Our Table 1 already includes leading on-chip CO_2 sensor demonstrations, and our work illustrates that comparable or better detection limits can be attained in the near-infrared while maintaining favorable practical attributes. In addition, the line intensity of N_2O at 7.70 μm is 1.681×10^{-19} cm/mol. The line strength of the band we measured for CO_2 is 1.761×10^{-23} cm/mol. The line strength of N_2O is about 9546 times greater than that of CO_2. Through evaluation, theoretically, our technology could theoretically reach a detection level on the order of tens of ppq for N_2O under similar conditions.

In direct response to your suggestion to ensure a meaningful and fair comparison among different CO_2 sensing technologies, we have carefully re-examined and updated several entries in the table based on the most recent and reliable data available in the literature.

The key revisions include:

Multi-pass cell: LOD adjusted to 130 ppb (from 450 ppb), Optimal integration time adjusted to 18 s, Wavelength adjusted to 2.004 μm.

On-chip waveguide sensor: LOD adjusted to 627 ppb (from 7.56 ppm), Optimal integration time adjusted to 36 s, Wavelength adjusted to 4.23 μm.

SEIRA sensor: LOD adjusted to 1.0 ppm (from 20.0 ppm), Wavelength adjusted to 6-8 μm.

MEMS-based sensor: LOD adjusted to 580 ppt (from 50.0 ppm), Optimal integration time adjusted to 49 s, Wavelength adjusted to 4.323 μm.

LITES: LOD adjusted to 3.59 ppm (from 47.7 ppm), Optimal integration time adjusted to 100 s, Wavelength adjusted to 2.00401 μm.

Add non-functionalized dispersed WGM sensor: LOD is 184 ppb, Wavelength is 1.55 μm.

These updates ensure that the table more faithfully captures the current landscape of CO_2 sensing technologies, providing readers with a balanced and informative comparison across different approaches. The revised table now better underscores the trade-offs between limit of detection, system complexity, size, and cost-contextualizing the competitive position of our dissipative WGM sensor, which achieves 168 ppt LOD while maintaining ease of alignment, small sample volume, and low cost.

To ensure the accuracy and currency of the data presented in the revised Table 1, we have also updated the corresponding references to reflect the sources of the newly cited performance metrics. These updates ensure full traceability and align the reference list precisely with the revised data in Table 1.

The key reference updates are as follows:

47. Gu, M. *et al.* Portable TDLAS Sensor for Online Monitoring of CO_2 and H_2O Using a Miniaturized Multi-Pass Cell. *Sensors* **23**, 2072 (2023).

50. Lim, J. *et al.* Ultrasensitive Mid-Infrared Optical Gas Sensor Based on Germanium-on-Insulator Photonic Circuits with Limit-of-Detection at Sub-ppm Level. *ACS Photonics* **11**, 4268-4278 (2024).

51. Zhou, H., Ren, Z., Xu, C., Xu, L. & Lee, C. MOF/Polymer-Integrated Multi-Hotspot Mid-Infrared Nanoantennas for Sensitive Detection of CO_2 Gas. *Nano-Micro Lett.* **14**, 207 (2022).

54. Yin, Y., Ren, D., Wang, Y., Gao, D. & Shi, J. Design and Test of a High-Sensitivity MEMS Capacitive Resonator for Photoacoustic Gas Detection. *IEEE Sens. J.* **24**, 40171-40178 (2024).

57. Mu, J. *et al.* LITES-Based Sensitive CO_2 Detection Using 2 μm Diode Laser and Self-Designed 9.5 kHz Quartz Tuning Fork. *Sensors* **25**, 2099 (2025).

67. Qin, P., Kang, X. & Gan, X. Machine learning-enhanced on-chip micro-ring resonator platform for detection and recognition of low-concentration gas mixtures. *Opt. Express* **33**, 24844-24854 (2025).

We have revised Fig. 7 in the revised manuscript as:

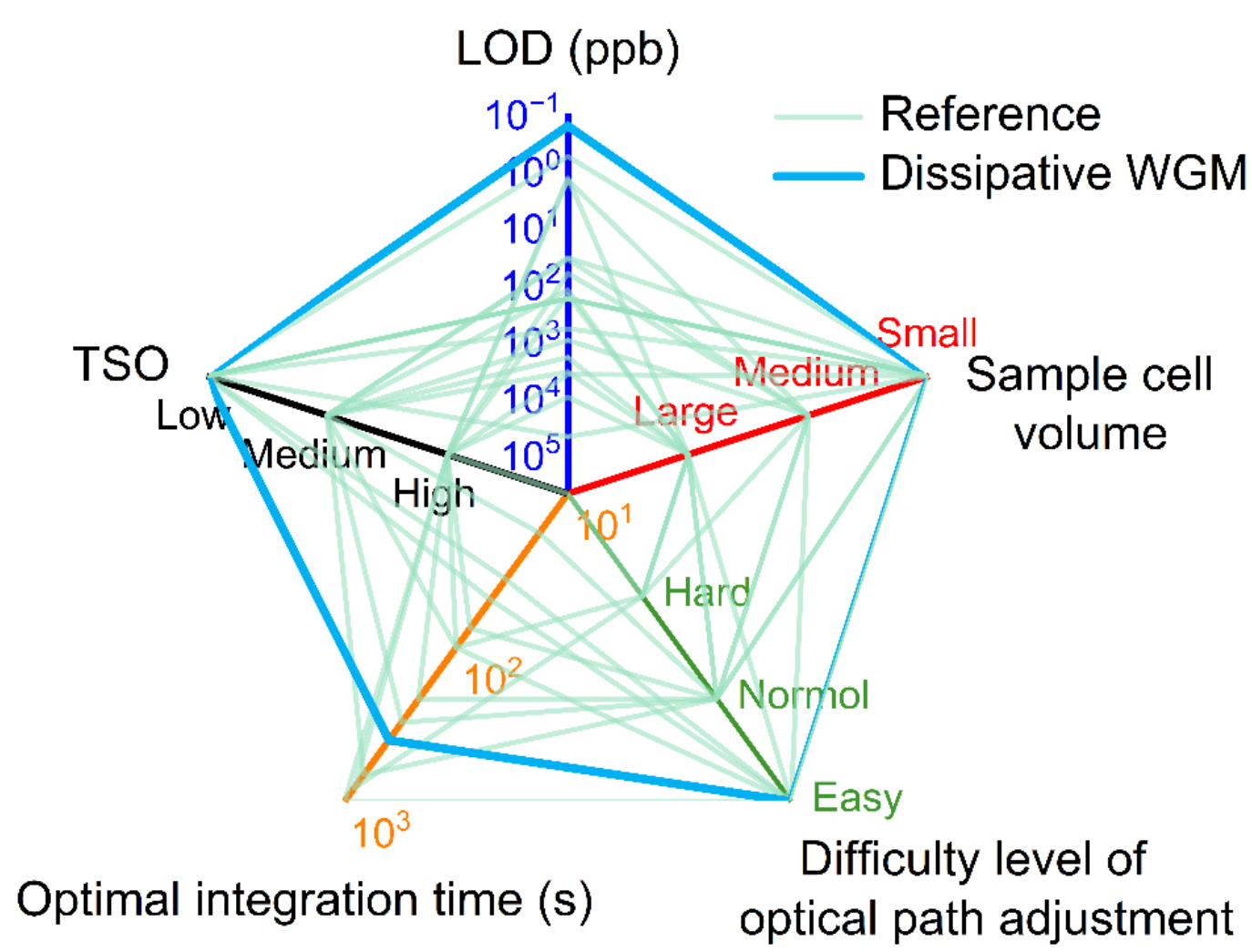

Fig.7 | Comparison of Sensor Performance. Performance comparison of the dissipative WGM gas sensor with state-of-the-art CO_2 sensors from the literature (Table 1 with Supporting References).

We have corrected Table 1 in the revised manuscript as:

Table 1. Comparison of gas sensing performance of state-of-the-art CO_2 sensors

Spectral technology	LOD[a]	Sample cell volume	Difficulty level of optical path adjustment	TSO[b]	Optimal integration time	Wavelength
Multi-pass Cell[47]	130 ppb	Large	Normal	Medium	18 s	2.004 μm
OA-CEAS[48]	1.40 ppb	Large	Hard	High	100 s	1.6 μm
CRDS[49]	160 ppb	Large	Hard	High	816 s	1.6 μm
On-chip[50]	627 ppb	Small	Easy	Low	36 s	4.23 μm
SEIRA[51]	1.0 ppm	Medium	Hard	Medium	N/A	6-8 μm
PAS[52]	37.0 ppb	Medium	Normal	Medium	307 s	4.3 μm
QEPAS[53]	35.0 ppb	Small	Normal	Medium	670 s	2.0 μm
MEMS[54]	580 ppt	Small	Easy	Low	49 s	4.323 μm
CEPAS[55]	4.00 ppm	Small	Normal	High	75 s	1.57 μm
Photothermal Spectroscopy[56]	1.50 ppb	Medium	Easy	High	1000 s	2.003 μm
LITES[57]	3.59 ppm	Large	Easy	Medium	100 s	2.00401 μm
Dispersion Spectroscopy[58]	65.78 ppb	Medium	Normal	High	218 s	4.329 μm
NDIR[59]	2.00 ppm	Large	Normal	Medium	N/A	4.3 μm
FTIR[60]	10.0 ppm	Large	Hard	High	5 s	4.25 μm
Fiber FP Interferometer[61]	480 ppb	Small	Easy	Medium	N/A	4.266 μm
Fiber Grating[62]	401 ppm	Medium	Easy	Medium	20 s	0.7 μm
Silicon Microring Resonator[63]	370 ppm	Small	Easy	High	N/A	1.56 μm
Antiresonant Fiber[64]	144 ppm	Small	Normal	Medium	1.5 s	1.574 μm
Photonic Crystals[65]	2000 ppm	Small	Easy	Medium	N/A	0.463-0.668 μm
Functionalized Dispersed WGM[66]	50.0 ppm	Small	Easy	Low	N/A	1.525-1.57 μm
Dispersed WGM[67]	184 ppb	Small	Easy	Low	N/A	1.55 μm
Dissipative WGM (This work)	***168 ppt***	***Small***	***Easy***	***Low***	***400 s***	***1.572 μm***

[a]LOD represents the limit of detection.

[b]TSO represents total system cost.

Reply to Reviewer #2

Ruan et al, measure gas absorption signals through variations in resonance depth of a WGM microsphere sensor. They achieve detection over a ppm range and a minimum detection limit of 0.12 ppb. Although the manuscript appears technically correct, I cannot recommend acceptance at the level of Nature Communications due to the following:

We thank the reviewer for the careful reading of our manuscript and for providing constructive comments. We understand the reviewer's concern regarding whether the manuscript meets the publication criteria of *Nature Communications*. In response to the reviewer's comments and the editorial guidance, we have carefully revised the manuscript and carried out substantial additional analyses and clarifications to strengthen the presentation and technical rigor of the work.

Specifically, the manuscript has been thoroughly revised, and extensive supplementary information has been added to provide detailed experimental validation, methodological clarification, and additional discussions requested by the reviewers. These revisions significantly improve the clarity and completeness of the study.

Importantly, this work demonstrates a gas sensing approach based on dissipative whispering-gallery-mode (WGM) microcavity sensing, in which molecular absorption is transduced through variations in the resonance depth of the cavity. We believe this mechanism represents a distinct sensing paradigm compared with conventional dispersive or absorption-based approaches. We hope that the revised manuscript and the detailed responses below will help clarify the novelty and significance of the work.

Comment 1: *The concept of gas absorption causing change in resonance depth is the basis for microcavity absorption spectroscopy, which is well known (and done better by WGM based frequency combs as they have multiple spectral lines). Those systems are also more truly compact than this one as this one relies on a tapered optical fiber which will hinder true portability). See: https://opg.optica.org/oe/fulltext.cfm?uri=oe-15-20-12959 and all other WGM frequency comb gas sensing papers.*

Answer 1: Thank you for your comments. The concept that gas absorption modifies the resonance

depth in microcavities has indeed been discussed in previous studies. The article you provided does outline the principle of basic dissipative sensing, but it does not include any experimental validation for detection. While dissipative whispering gallery mode (WGM) sensors have been reported, and we have cited experimental examples in the introduction (such as acoustic sensing). To the best of our knowledge, experimental demonstrations of gas sensing based specifically on the dissipative WGM sensing mechanism remain very limited.. In this work, we not only refine the theory of dissipative WGM sensors for gas detection but also carry out corresponding experimental verification, achieving substantially improved detection sensitivity compared with conventional dispersive-type WGM sensing schemes. Moreover, the proposed theory applies to all mechanisms where temperature rise induces changes in coupling strength, thereby extending the applicability of dissipative WGM microcavity sensors beyond gas detection to any scenario involving such thermally induced coupling variations.

It is true that on-chip microring WGM sensors with waveguide coupling offer greater portability compared to tapered fiber coupling. However, most systems still rely on off-chip laser sources, which require fiber coupling into the chip and demand extremely high alignment precision between the fiber and the chip. In terms of system architecture and portability, these setups are comparable to our proposed system. After packaging, our packaged system can still provide reasonable robustness and portability for laboratory and field demonstrations, as shown in Fig. R13 in the manuscript.

Furthermore, our sensor is fundamentally different from microcavity optical frequency combs. A microcavity frequency comb is essentially an active device that leverages the Kerr nonlinearity of the microcavity material to generate multiple laser lines at different wavelengths. When interacting with gases, the gas affects the comb's complex dynamics by modifying the cavity refractive index and introducing absorption losses, both of which alter the comb signal. As the reviewer noted, the advantage of microcavity combs lies in their multiple spectral lines, enabling detection of gases with absorption peaks at different wavelengths. In particular, dual-comb schemes based on microcavities have emerged as highly promising on-chip sensing platforms for the future. However, due to their operational principle, they require sophisticated experimental setups in practice. For instance, precise locking of the laser-cavity detuning to achieve soliton state combs. Unlike microcavity frequency comb systems that rely on nonlinear comb generation and complex stabilization schemes, our approach directly transduces molecular absorption into resonance depth variation using a passive microcavity. This enables a significantly simpler sensing architecture.

In contrast, the dissipative WGM microcavity sensor proposed in this work remains a passive device. The incident laser serves to induce the photothermal effect and probe the cavity modes, rather than pumping the cavity to generate optical nonlinearities. By employing a widely tunable laser (e.g., an external-cavity diode laser), our sensor could also target gas absorption lines at different wavelengths. Compared with microcavity frequency combs, our sensor offers advantages in terms of structural simplicity of the experimental setup and signal generation.

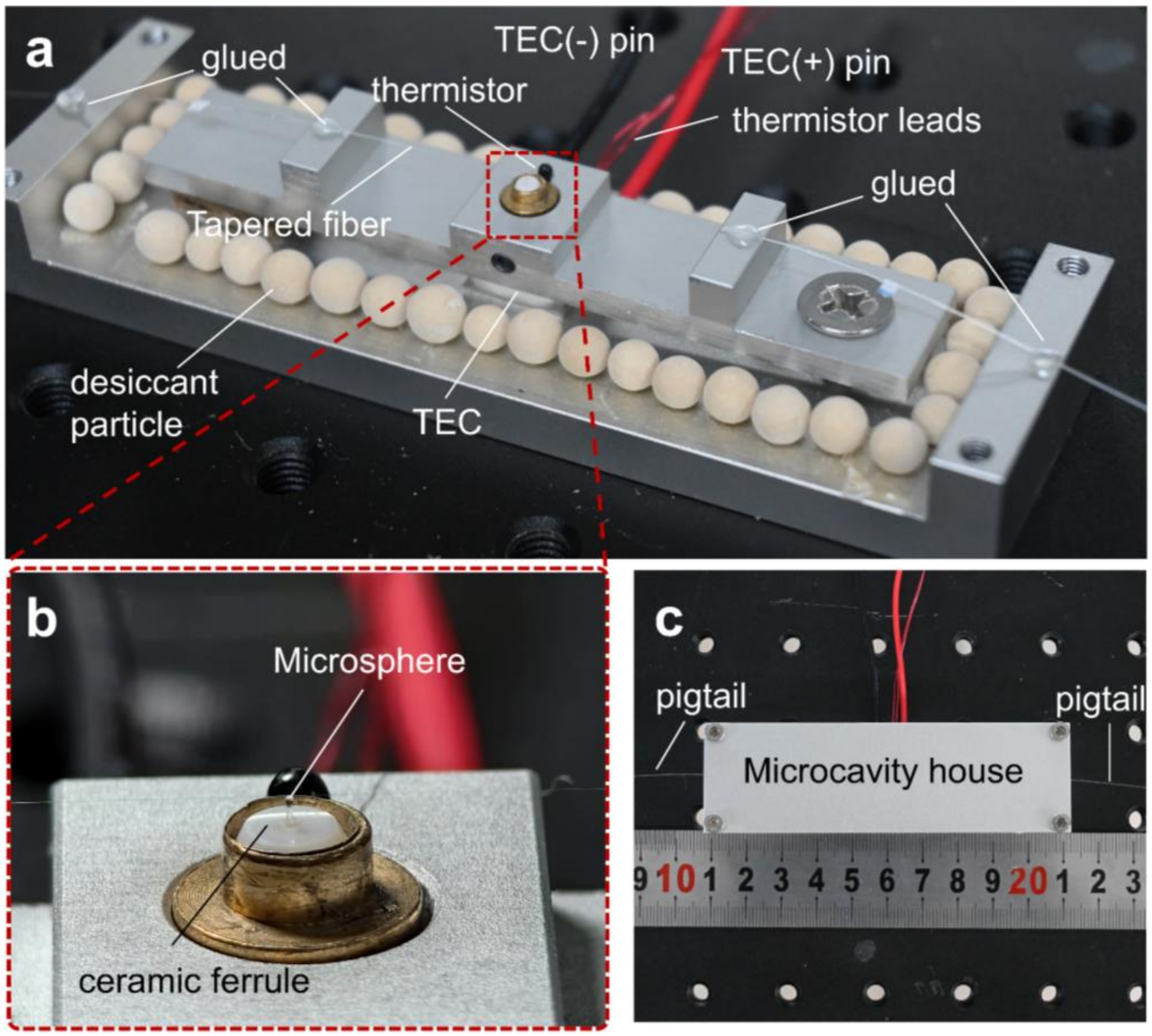

Fig. R13. a, Packaging structure of tapered fiber coupled with microsphere WGM microcavity. **b**, Zoom of the black dashed square in **a**. **c**, Photograph of the fully packaged sensor device.

Comment 2: *The concept of optimizing the dither frequency amplitude for improved signal has already been published e.g., Journal of Lightwave Technology 38, 6393–6401 (2020).*

Answer 2: Thank you for raising this important point regarding the optimization of modulation parameters. We appreciate your attention to the relevant prior work on frequency-locked

microcavity sensing (Journal of Lightwave Technology 38, 6393–6401 (2020)), which we have also cited in our revised manuscript as a key reference in the advancement of WGM-based sensing systems.

In the cited article, the authors used an electronic feedback loop to lock the laser frequency to the microcavity resonance. Their system uses a dither signal to modulate the laser frequency and generate an error signal for active stabilization. By detecting changes in the control signal within the frequency locking loop, they can detect shifts in the microcavity resonance frequency caused by external disturbances. Therefore, this sensor is essentially still a dispersive WGM microcavity sensor. The cited article solves the problem of detuning between the laser frequency and the microcavity resonance in dispersive microcavity sensors, thus significantly improving the detection performance. At the same time, this technique can track the microcavity resonance shift in real time, which is very promising for achieving real-time detection and improving the signal-to-noise ratio of the sensor. However, these sensors still face the problem of being insensitive to low concentrations of gases. Therefore, to achieve high sensitivity and low detection limits, functionalization of the microcavity surface is still necessary. As shown in the two references mentioned in Comment 3, to achieve sub-ppb level gas detection, it is still necessary to modify the microcavity surface with ferrocene-based polymers for gases such as NO.

In contrast, our work employs wavelength modulation technique, which is fundamentally different in both mechanism and purpose. Our modulation is achieved by applying current modulation to the laser wavelength, encoding the raw mode signal into a harmonic signal, and then extracting the signal at the second harmonic frequency using a lock-in amplifier. This is an optimized scheme for applying our dissipative microcavity sensor to trace gas detection. Since the dissipative sensing mechanism of WGM microcavities utilizes the conversion of gas absorption into a measurable change in mode resonance depth, the laser intensity and electronics noise will directly affect the sensitivity of the signal. In dispersive WGM sensors, however, the laser frequency noise is a significant factor affecting signal sensitivity. Therefore, this is the fundamental difference in motivation between our modulation-demodulation scheme and the laser frequency locking scheme mentioned in the cited article.

As shown in Fig 2a in the manuscript, the intensity noise of our laser was measured to be close to the quantum noise limit (shot noise) at frequency offsets >1 kHz. Therefore, modulating the signal to a high-frequency band effectively suppresses the influence of low-frequency noise on the detection sensitivity. The modulation-demodulation scheme serves as an auxiliary tool to improve signal quality and demonstrate the ultimate performance of our sensing platform. As

shown in Fig. 3 of the manuscript, we also present the measurement results obtained from the raw mode signal for comparison. It can be clearly observed that the results measured using the $2f$ signal exhibit significantly better performance than those obtained from the raw signal. This comparison highlights the effectiveness of the modulation–demodulation scheme in improving the signal-to-noise ratio of the sensing platform.

We hope this clarifies that although both studies involve modulation, their physical principles, system architectures, and sensing objectives are different. It is worth noting that laser frequency locking can lock the laser frequency to the microcavity resonance, which is compatible with our dissipative sensing mechanism. Thank you again for your insightful comments; we will summarize the future development path of our dissipative sensor technology in the conclusion section.

We have added relevant discussion in the introduction in the revised manuscript as:

"In recent years, a number of emerging gas sensing techniques have been developed to overcome the limitations of traditional methods. These include photoacoustic[18,19] and photothermal[20,21] detection, fiber interferometry[22], fiber grating[23], and microcavity-based resonant sensing[24]. Most of them exploit interactions between gas molecules and light or sound, and offer key advantages such as compactness, low power consumption, and high sensitivity. Among these, optical microcavities based on whispering gallery modes (WGM) have shown great promise[25-30]. WGM cavities can be scaled down to the micrometer level while maintaining high Q-factors and excellent integrability. Thanks to their ultra-high intracavity field intensity, WGM sensors are exceptionally sensitive to slight changes in refractive index or absorption. Usually, WGM microcavities have dispersive sensing mechanism that detect the wavelength shift of microcavity resonance caused by changes in external parameters[31,32]. By utilizing frequency locking technology, the detuning between the laser frequency and the microcavity resonance can be eliminated, thereby effectively improving the signal-to-noise ratio and enabling real-time detection[25, 27, 28]. However, when a WGM microcavity sensor is employed for gas detection, the gas-induced variation in the refractive index of the microcavity is intrinsically weak, thereby limiting the detection sensitivity of the system." on Page 3.

We have added relevant discussion in the outlook in the revised manuscript as:

"Furthermore, implementation of frequency-locking techniques to stabilize the laser to the cavity resonance would further enhance long-term stability and enable continuous real-time monitoring. Combined with the maturity and availability of telecom-grade light sources and

detectors, these attributes position the dissipative WGM-based sensor as a promising platform for high-sensitivity, compact, and cost-effective trace gas detection in both laboratory and field applications." on Page 22.

We have added relevant references in the revised manuscript as:

25. Hao, S. & Su, J. Noise-Induced Limits of Detection in Frequency Locked Optical Microcavities. *J. Lightwave Technol.* **38**, 6393-6401 (2020).

27. Gin, A. *et al.* Label-free, real-time monitoring of membrane binding events at zeptomolar concentrations using frequency-locked optical microresonators. *Nat. Commun.* **15**, 7445 (2024)

28. Suebka, S., Gin, A. & Su, J. Frequency locked whispering evanescent resonator (FLOWER) for biochemical sensing applications. *Nat. Protoc.* **20**, 1616-1650 (2025)

Comment 3: *The authors claim that their detection limit is five orders of magnitude better than other WGM sensors which rely on resonance frequency shift tracking, however, this is exaggerated. Toroidal sensors have demonstrated low ppt sensing: (e.g., ACS Appl. Mater. Interfaces 16, 5120–5128 (2024) and ACS Appl. Mater. Interfaces 14, 42430–42440).*

Answer 3: We thank the reviewer for pointing out that the original statement could be misleading. In our work, we demonstrated that our sensor exhibits a detection limit five orders of magnitude better than other WGM sensors that rely on resonance frequency shift tracking specifically for CO_2 detection. This comparison was originally based on previously reported dispersive WGM sensors targeting CO_2 [Ref: *Opt. Express* 27, 1991-2000, 2019]. We have discovered recent research through further investigation [Ref: *Opt. Express* 33, 24844-24854, 2025], which achieves an LOD of 184 ppb for CO_2 using a dispersive WGM scheme. Accordingly, we have revised the statement in the Abstract to more accurately reflect that our sensor's limit of detection is superior to that of the reported dispersive WGM sensors targeting CO_2, rather than claiming a five-orders-of-magnitude improvement.

We have made modifications to the relevant discussions in the revised manuscript abstract as:

"The sensor enables quantitative detection over a concentration range of 1.5-400 ppm (R^2 > 0.99), achieving an accuracy of ~0.4% and a limit of detection of 168 ppt at an integration time of

400 s. Its performance surpasses that of previously reported dispersive WGM-based sensors for CO_2 detection." on Page 1.

The two papers you pointed out-ACS Appl. Mater. Interfaces 2024, 16, 5120–5128 (NO detection) and ACS Appl. Mater. Interfaces 2022, 14, 42430-42440 (DIMP, NH_3, HCHO detection)-are indeed significant contributions to the field of functionalized WGM sensing, achieving remarkable ppt-level sensitivity for their respective target gases. In addition, through further investigation of the literature, we found that the authors of the above-mentioned references have also reported other WGM sensors for biomolecular detection [Ref: *Nat. Commun.* **15**, 7445, 2024 and *Nat. Protoc.* **20**, 1616-1650, 2025]. Those studies adopted laser frequency-locking techniques, which, if combined with our system, could achieve even higher signal-to-noise ratio and sensitivity. We therefore plan to integrate this approach into our future work. We have cited these important references in our revised manuscript and discussed their significance in the laser frequency locking technology and the context of selective gas sensing with surface-modified microcavities.

We have added relevant references in the revised manuscript as:

27. Gin, A. *et al.* Label-free, real-time monitoring of membrane binding events at zeptomolar concentrations using frequency-locked optical microresonators. *Nat. Commun.* **15**, 7445 (2024)

28. Suebka, S., Gin, A. & Su, J. Frequency locked whispering evanescent resonator (FLOWER) for biochemical sensing applications. *Nat. Protoc.* **20**, 1616-1650 (2025)

33. Xu, Y. *et al.* Low Part-Per-Trillion, Humidity Resistant Detection of Nitric Oxide Using Microtoroid Optical Resonators. *ACS Appl. Mater. Interfaces* **16**, 5120-5128 (2024).

34. Li, C. *et al.* Part-per-Trillion Trace Selective Gas Detection Using Frequency Locked Whispering-Gallery Mode Microtoroids. *ACS Appl. Mater. Interfaces* **14**, 42430-42440 (2022).

We have added relevant discussion in the revised manuscript as:

"It is observed that sensing schemes based on WGM microcavities functionalized with specific coatings have demonstrated low limits of detection for certain gases. For instance, a WGM sensor coated with a ferrocene-containing polymer has achieved a limit of detection of 2.34 ppt for nitric oxide[33], while a WGM sensor modified with a polyvinyl alcohol coating has reached 117 ppt for formaldehyde[34]. These studies outline multiple promising pathways for optical gas sensing

toward higher sensitivity, improved selectivity, and improved integrability." on Page 4.

However, it is important to note that direct comparison of LOD across different gases can be misleading, as the detection performance depends strongly on gas-specific properties such as polarizability, absorption strength, interaction mechanisms with functional layers, and diffusion dynamics. For example, NO exhibits strong and selective binding with ferrocene-based polymers, enabling exceptional sensitivity. In contrast, CO_2 is chemically less reactive and more challenging to detect with high sensitivity in unmodified or weakly interacting surfaces, which is why conventional dispersive WGM sensors for CO_2 cannot achieve good LODs.

The difference in principle between the dispersive WGM sensor and our dissipative WGM sensor has been elucidated in the previous two comments. WGM microcavities utilize high-Q optical resonances where light circulates along the cavity perimeter, creating an enhanced evanescent field extending from the surface. When the cavity surface is functionalized with a selective polymer coating (e.g., ferrocene-containing polymers for NO), target gas molecules interact specifically with the coating through adsorption, chemical reactions, or swelling, thereby changing the local refractive index or cavity radius. This induces a shift in the resonance wavelength, which is tracked in real time with high resolution (e.g., via frequency-locked systems like FLOWER). The extreme sensitivity arises from the long photon confinement and high Q, enabling detection down to ppt levels with selectivity conferred by the functionalized coating. In contrast, our approach relies on wavelength-selective molecular absorption rather than surface functionalization. The achievable sensitivity is expected to scale with the absorption line strength of the target gas. Functionalized dispersion WGM sensing is also an excellent gas detection method, but the two belong to different technical paths. Compared to functionalized WGM sensors, the unmodified microcavity in our sensor exhibits a longer operational lifetime.

In our study, we introduce a dissipative sensing mechanism that converts molecular absorption into resonance depth variation rather than tracking resonance frequency shifts. Because the signal originates from wavelength-selective molecular absorption, the achievable sensitivity is expected to depend on the absorption line strength of the target gas.

To illustrate this point and demonstrate the generality of our platform, we additionally performed sensing experiments for CH_4 and C_2H_2 using DFB lasers at 1.651 μm and 1.542 μm, respectively (Figs. S12 and S13). The absorption line intensity of CH_4 at 1651 nm (1.520×10^{-21} cm/mol) is approximately 86 times stronger than that of the CO_2 line used at 1572.3 nm (1.761×10^{-23} cm/mol), resulting in a significantly improved limit of detection of ~7.2 ppt compared with ~168 ppt for CO_2. Similarly, the C_2H_2 transition at 1542 nm has a line strength of 2.602×10^{-22}

cm/mol, about 14 times stronger than that of the CO_2 transition used here, leading to a detection limit of ~35 ppt. These results collectively indicate that the detection sensitivity of the dissipative WGM sensing platform scales positively with the absorption line intensity of the target gas.

Additional CH_4 and C_2H_2 sensing experiments have been added in the Supplementary Note 5 to demonstrate the generality of the sensing mechanism. Detailed experimental results are provided in Figs. S12 and S13.

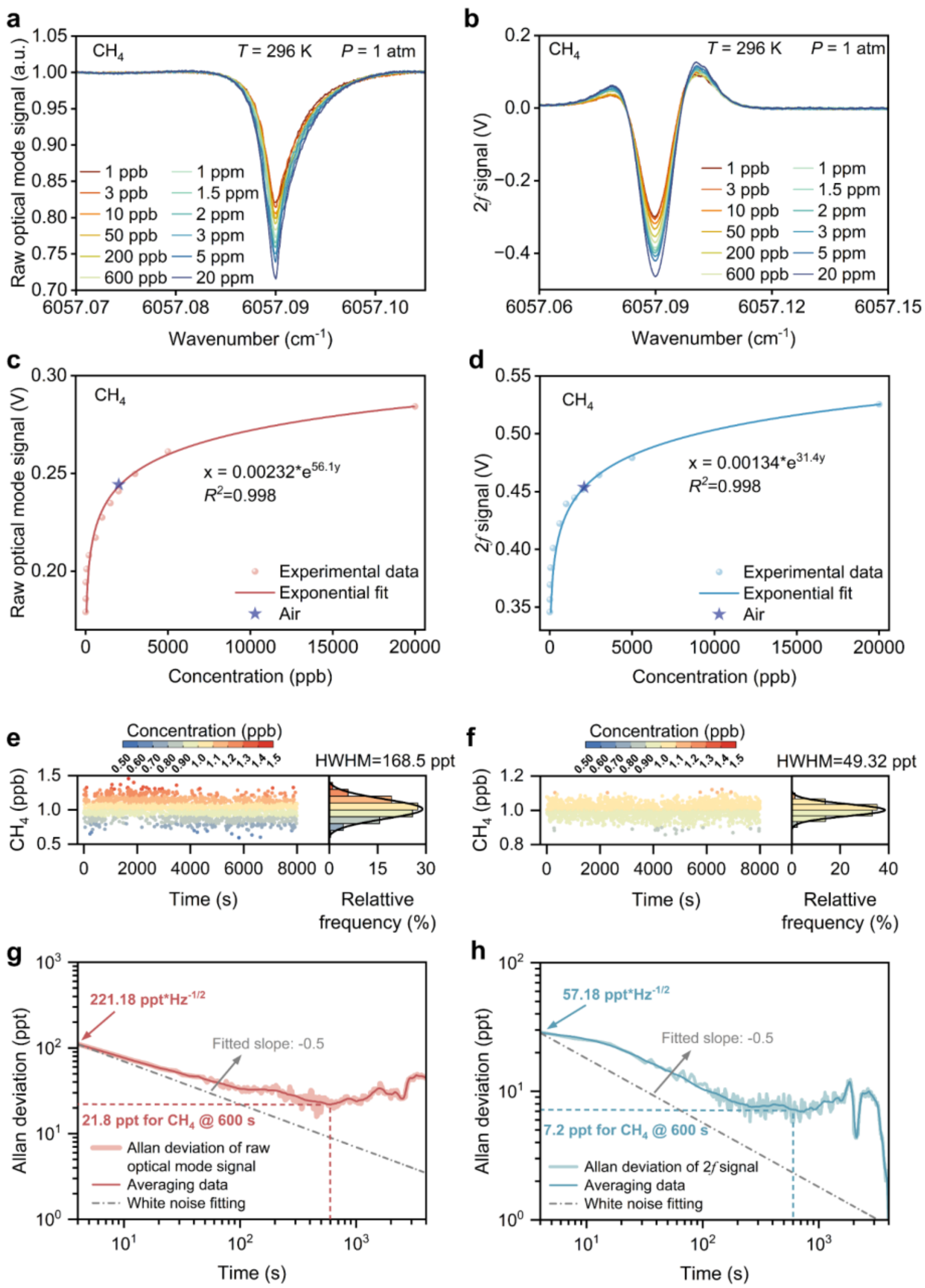

Fig. S12 | a, The raw optical mode signals measured at different CH_4 concentrations. **b**, The 2*f* signals measured at different CH_4 concentrations. **c**, Experimental data points and the corresponding fitted curve of optical mode amplitude as a function of CH_4 concentration. **d**,

Experimental data points and corresponding fitted curve of 2*f* amplitude as a function of CH_4 concentration. **e**, Distribution of CH_4 concentration measured continuously for 8000 s for 1 ppb CH_4-N_2 sample using raw optical mode signal (T=296 K, P=1 atm), as well as the frequency distribution and Gaussian profile fitting of measured concentration. **f**, Distribution of CH_4 concentration measured continuously for 8000 s for 1 ppb CH_4-N_2 sample using 2*f* signal (T=296 K, P=1 atm), as well as the frequency distribution and Gaussian profile fitting of measured concentration. **g** and **h**, Allan deviation of the dissipative WGM gas sensor based on the data shown in Figs. 6e and 6f.

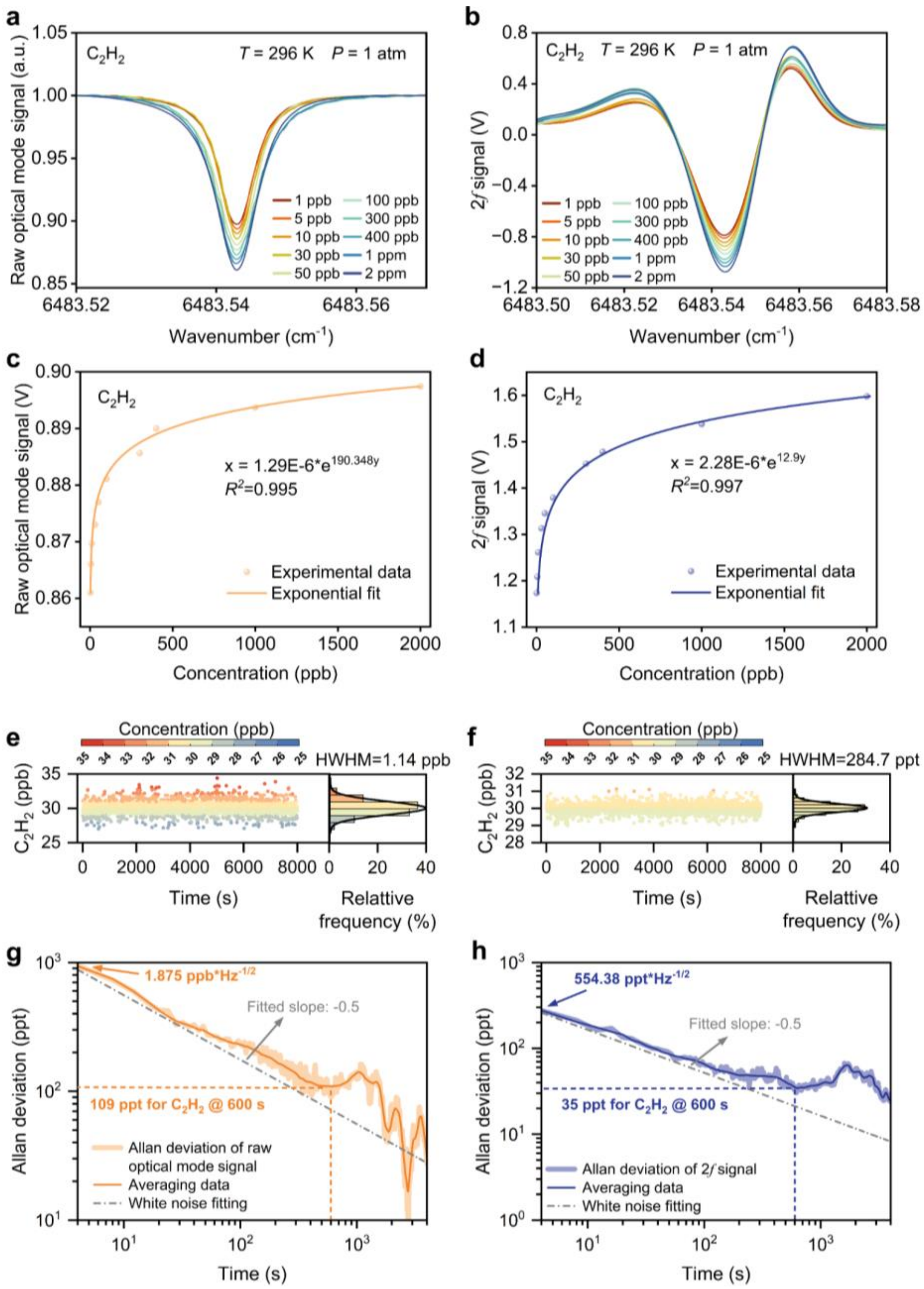

Fig. S13 | a, The raw optical mode signals measured at different C_2H_2 concentrations. **b**, The 2*f*

signals measured at different C_2H_2 concentrations. **c**, Experimental data points and the corresponding fitted curve of optical mode amplitude as a function of C_2H_2 concentration. **d**, Experimental data points and corresponding fitted curve of $2f$ amplitude as a function of C_2H_2 concentration. **e**, Distribution of C_2H_2 concentration measured continuously for 8000 s for 30 ppb C_2H_2-N_2 sample using raw optical mode signal (T=296 K, P=1 atm), as well as the frequency distribution and Gaussian profile fitting of measured concentration. **f**, Distribution of C_2H_2 concentration measured continuously for 8000 s for 30 ppb C_2H_2-N_2 sample using $2f$ signal (T=296 K, P=1 atm), as well as the frequency distribution and Gaussian profile fitting of measured concentration. **g** and **h**, Allan deviation of the dissipative WGM gas sensor based on the data shown in Figs. 6e and 6f.

Perhaps our previous description was not sufficiently clear, which may have led to some misunderstanding by the reviewers. We have revised the relevant section in the manuscript to explicitly state that the claimed improvement refers specifically to dispersion-based CO_2-sensing WGM sensors. Additionally, we have added the two references mentioned by the reviewer, along with two further studies by the same authors on biosensing, to appropriately position our work within the context of prior achievements in functionalized WGM sensing.

Comment 4: *There is also no demonstration of selectivity, which is what the polymer coated WGM sensors show. I would have expected this to be tested with other gases as well. It's not clear to me how the real-world demonstration proves selective detection of CO2. Other gases can also show diurnal fluctuations.*

Answer 4: Thank you for raising the important point regarding sensor selectivity and for highlighting the impressive selectivity achieved by polymer-coated WGM sensors. We appreciate the opportunity to clarify the fundamentally different mechanism by which our sensor achieves selectivity, which is indeed a critical aspect of our work.

The selectivity of our sensor is not derived from a functionalized coating, but rather from spectroscopic selectivity inherent to the gas molecules themselves. Our dissipative sensing scheme operates by locking a tunable laser to a wavelength that coincides with a characteristic electronic or vibrational absorption line of a specific target gas (e.g., CO_2 at 1.572 μm, CH_4 at 1.651 μm). Only when the laser wavelength overlaps with a gas's absorption feature does the absorption-

induced change in the cavity's optical loss (manifested as a resonance depth change) become significant. The narrow linewidth of the laser enables precise locking onto the target absorption line, ensuring strong resistance to interference from other gases. This principle forms the very basis of our method and provides its intrinsic selectivity.

To rigorously demonstrate this, we performed a series of cross-sensitivity experiments with gas mixtures. The sensor platform (a bare, non-functionalized WGM microsphere) was placed in a sealed chamber. We used three near-infrared lasers, which can work in the gas absorption bands of CO_2, CH_4 and C_2H_2 respectively, and measured a series of mixed gases with different concentrations through the precision gas mixing system. In addition to these three gases, other gases in the seal gas chamber are high-purity N_2.

3.1. Selectivity Test with CO_2-Absorption Laser

Using a laser locked at the CO_2 absorption band (~1.572 μm), we varied only the CO_2 concentration (400 ppm, 200 ppm, 50 ppm and 25 ppm) while holding the concentrations of CH_4 and C_2H_2 constant. A clear and corresponding change in the resonance depth was observed, as shown in Fig. S15a. Conversely, when the CO_2 concentration was held constant and the concentrations of CH_4 and C_2H_2 were varied independently, no significant change in the raw optical mode signal was detected by the CO_2-tuned laser, as shown in Fig. S15d.

3.2. Control Experiments with Other Gases

The same experiment was repeated using lasers tuned to the characteristic absorption bands of CH_4 (~1.651 μm) and C_2H_2 (~1.54 μm). In perfect agreement with the principle:

With the CH_4-tuned laser, the raw optical mode signal changed only when the CH_4 concentration was varied, remaining unaffected by changes in CO_2 and C_2H_2 concentrations, as shown in Figs. S15b and S15e.

With the C_2H_2-tuned laser, the raw optical mode signal changed only when the C_2H_2 concentration was varied, remaining unaffected by changes in CO_2 and CH_4 concentrations, as shown in Figs. S15c and S15f.

These controlled experiments provide strong evidence that the sensor's response is exclusively tied to the concentration of the gas whose absorption line matches the laser wavelength. These results collectively confirm that the sensing response originates from wavelength-specific molecular absorption rather than nonspecific environmental perturbations..

Regarding the field demonstration, the observed diurnal fluctuation pattern is strongly correlated with known anthropogenic activity and is consistent in magnitude and trend with typical atmospheric CO_2 dynamics. While we acknowledge that other gases can also exhibit diurnal cycles,

the combination of this real-world correlation and, more decisively, the controlled laboratory mixture experiments described above provides a robust argument for the selective detection of CO_2 in our demonstration. We carried out new CO_2 measurements over two consecutive days, with parallel measurements taken using a commercial-grade, calibrated CO_2 analyzer. The results showed good agreement with the reference analyzer.

In summary, our sensor achieves selectivity through wavelength-specific optical absorption, a complementary approach to the material-specific chemical affinity used in polymer-coated sensors. We have now added a detailed description of these selectivity experiments to the revised manuscript to explicitly address this key point.

We have added Fig. S15 to the revised supplementary materials:

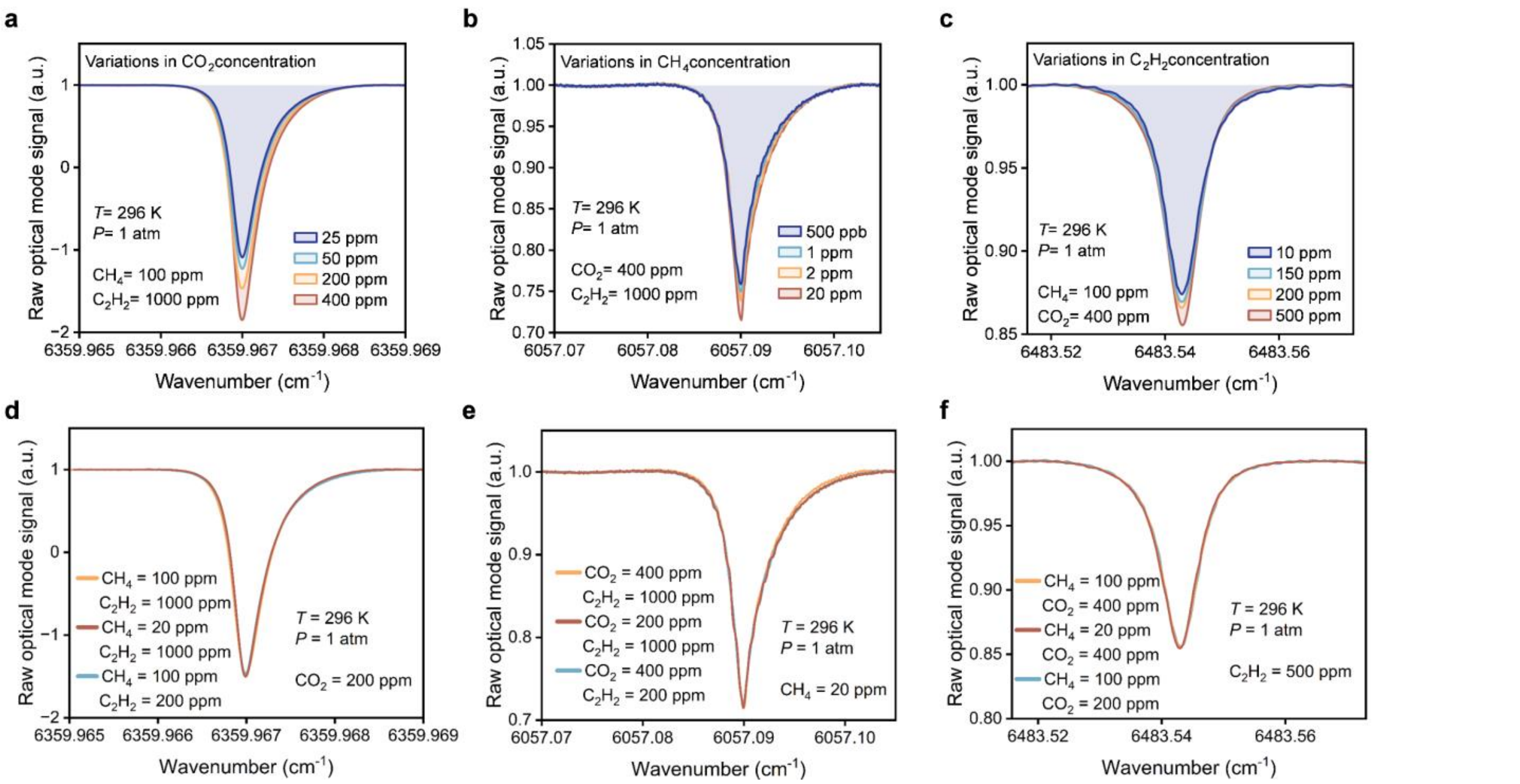

Fig. S15 | Verification experiment of gas selectivity. a, Variation of the raw optical mode signal of CO_2 as a function of CO_2 concentration measured in CO_2-C_2H_2-CH_4 gas mixtures with fixed C_2H_2 and CH_4 concentrations. **b**, Variation of the raw optical mode signal of CH_4 as a function of CH_4 concentration measured in CO_2-C_2H_2-CH_4 gas mixtures with fixed C_2H_2 and CO_2 concentrations. **c**, Variation of the raw optical mode signal of C_2H_2 as a function of C_2H_2 concentration measured in CO_2-C_2H_2-CH_4 gas mixtures with fixed CH_4 and CO_2 concentrations. **d**, Response of the raw optical mode signal of CO_2 to varying concentrations of CH_4 and C_2H_2, respectively, under a constant CO_2 concentration. **e**, Response of the raw optical mode signal of CH_4 to varying concentrations of CO_2 and C_2H_2, respectively, under a constant CH_4 concentration.

f, Response of the raw optical mode signal of C_2H_2 to varying concentrations of CH_4 and CO_2, respectively, under a constant C_2H_2 concentration.

On page 32.

We have added relevant discussions in the revised supplementary information (**Supplementary Note 7**) as:

"Gas Selectivity of the Dissipative WGM Sensor

The selective detection of a target gas within complex mixtures is a fundamental requirement for practical sensing. This supplementary section provides a detailed experimental demonstration and theoretical rationale for the intrinsic gas selectivity of our spectroscopic dissipative sensing platform, which operates on a principle distinct from functionalized coating-based sensors.

7.1 Principle of Spectroscopic Selectivity

The core mechanism enabling selectivity in this work is wavelength-specific optical absorption by gas molecules, a fundamental molecular property. The sensor employs a high-Q WGM microcavity as a sensitive probe for optical absorption by gas. A tunable diode laser is precisely tuned to a wavelength corresponding to a characteristic, narrow-linewidth electronic or vibrational absorption line of a specific target gas (e.g., CO_2 at 1.572 μm, CH_4 at 1.651 μm, C_2H_2 at 1.54 μm). When the laser wavelength spectrally overlaps with the target gas absorption feature, molecules present in the evanescent field of the optical mode absorb photons. Our approach leverages enhanced local optical fields and thermal effects at the coupling region to convert weak absorption into measurable variations in resonance depth. Crucially, gases lacking absorption features at the specific laser wavelength do not contribute to this resonant absorption change, thereby conferring inherent selectivity directly derived from molecular spectroscopy.

7.2 Experimental Validation via Controlled Cross-Sensitivity Tests

To rigorously validate this principle and rule out significant cross-interference, a series of controlled experiments was conducted using gas mixtures. An uncoated microsphere resonator was housed in a sealed gas cell with precise temperature and pressure control. Three distributed feedback (DFB) diode lasers were employed, each centered at the absorption line of a different target gas: CO_2, CH_4, and C_2H_2. Certified standard gases and a computerized multi-channel gas mixing system (Environics Series 4000) were used to generate mixtures with accurately defined concentrations, with high-purity N_2 serving as the balance gas.

7.2.1 Selective Response to Target Gas Variation

For each target gas, the corresponding laser was activated and locked to its absorption line. The concentration of the target gas was systematically varied (e.g., CO_2: 25, 50, 200, 400 ppm), while the concentrations of the other two potential interferent gases were maintained at constant, non-zero levels. As shown in Supplementary Fig. S15a-c, a strong, monotonic dependence of the resonance depth change of the raw optical mode signal on the target gas concentration was observed for each laser-gas pair. This demonstrates the sensor's primary sensitivity to the intended analyte.

7.2.2 Insensitivity to Non-Target Gas Variation (Cross-Interference Test)

The converse experiment was critical for proving selectivity. For a given laser (e.g., the CO_2-absorbing laser at 1.572 μm), the concentration of its target gas (CO_2) was held constant. Subsequently, the concentrations of the other gases (CH_4 and C_2H_2) were independently varied over a comparable concentration range. The results, summarized in Supplementary Fig. S15d-f, show no significant change in the measured resonance depth of the raw optical mode signal. The observed fluctuations remained within the baseline noise level of the sensor system. This confirms that the presence and varying concentration of gases not absorbing at the specific wavelength do not elicit a measurable response.

7.3 Discussion and Conclusion

The combined dataset from these controlled mixture experiments provides strong experimental evidence for the spectroscopic selectivity of the dissipative sensing mechanism. The sensor's response function is primarily governed by the Beer-Lambert law interaction between the spectrally narrow probe laser and the specific gas species possessing a resonant absorption at that wavelength. This approach fundamentally differs from polymer-coated WGM sensors, which rely on the partitioning of analytes into a functionalized film based on chemical affinity.

The real-world field demonstration of CO_2 monitoring, showing plausible diurnal variations, gains robust contextual support from these laboratory selectivity tests. While atmospheric diurnal patterns are not uniquely specific, the conjunction of field data with known anthropogenic drivers, and more definitively, the laboratory-proven absence of response to common potential interferents like CH_4 under the CO_2-measurement configuration, strongly supports the interpretation that the recorded signal mainly originates from CO_2 concentration changes.

In summary, the selectivity of this sensor platform is intrinsically rooted in the discrete absorption spectra of gas molecules. This method offers a powerful and complementary alternative to material-based selective coatings, particularly advantageous for applications requiring the

detection of small molecules with well-defined, narrow absorption features in the near-infrared region." On page 29.

Comment 5: *The system mounted on a laboratory cart is not, in my view, consistent with describing the platform as compact or portable.*

Answer 5: Thank you for your comment regarding the portability of the experimental setup. We appreciate the opportunity to clarify both the focus of this work and the ongoing efforts toward system miniaturization.

5.1 The core purpose of this article

We agree that the laboratory cart-based setup used in this feasibility demonstration does not represent a fully miniaturized or portable system. The primary objective of this manuscript is to introduce and experimentally validate a novel dissipative WGM sensing principle for trace gas detection. The key contribution of this method lies in its ability to convert weak molecular absorption into measurable resonance depth changes. The cart-based system served as a flexible testbed to rigorously evaluate the principle and benchmark its performance; it should be considered a verification platform rather than a final integrated implementation.

5.2 Progress toward miniaturization

Importantly, the sensing principle demonstrated does not rely on bulky instrumentation. The essential functional modules can be consolidated through optical packaging and circuit-level integration. Building on this, we have made substantial progress toward a compact, portable implementation (**Supplementary Note 4, Supplementary Note 8**).

Specifically, to achieve compactness, we have carried out the following tasks:

5.2.1 Microcavity Device Packaging

The core sensor element has been successfully packaged into a compact, stand-alone device. This packaging, detailed in **Supplementary Note 4**, enhances mechanical stability and allows for integrated temperature and humidity control, while significantly reducing the form factor of the cavity module itself.

5.2.2 Electronic Integration

The functions previously provided by separate commercial instruments—including laser control, function generator, lock-in amplifier, photodetector, and data acquisition—have been consolidated into a custom-designed board-level system An image of this integrated system is

provided in Supplementary Fig. R14 (now included in **Supplementary Note 8** as **Fig. S16**). This integrated module preserves all core measurement capabilities while substantially reducing footprint and complexity.

In summary, while the present article focuses on establishing a dissipative WGM sensing approach, we have concurrently pursued and achieved significant miniaturization of key components. The revised manuscript now includes a discussion on this integration path to provide a more complete perspective on the technology's scalability.

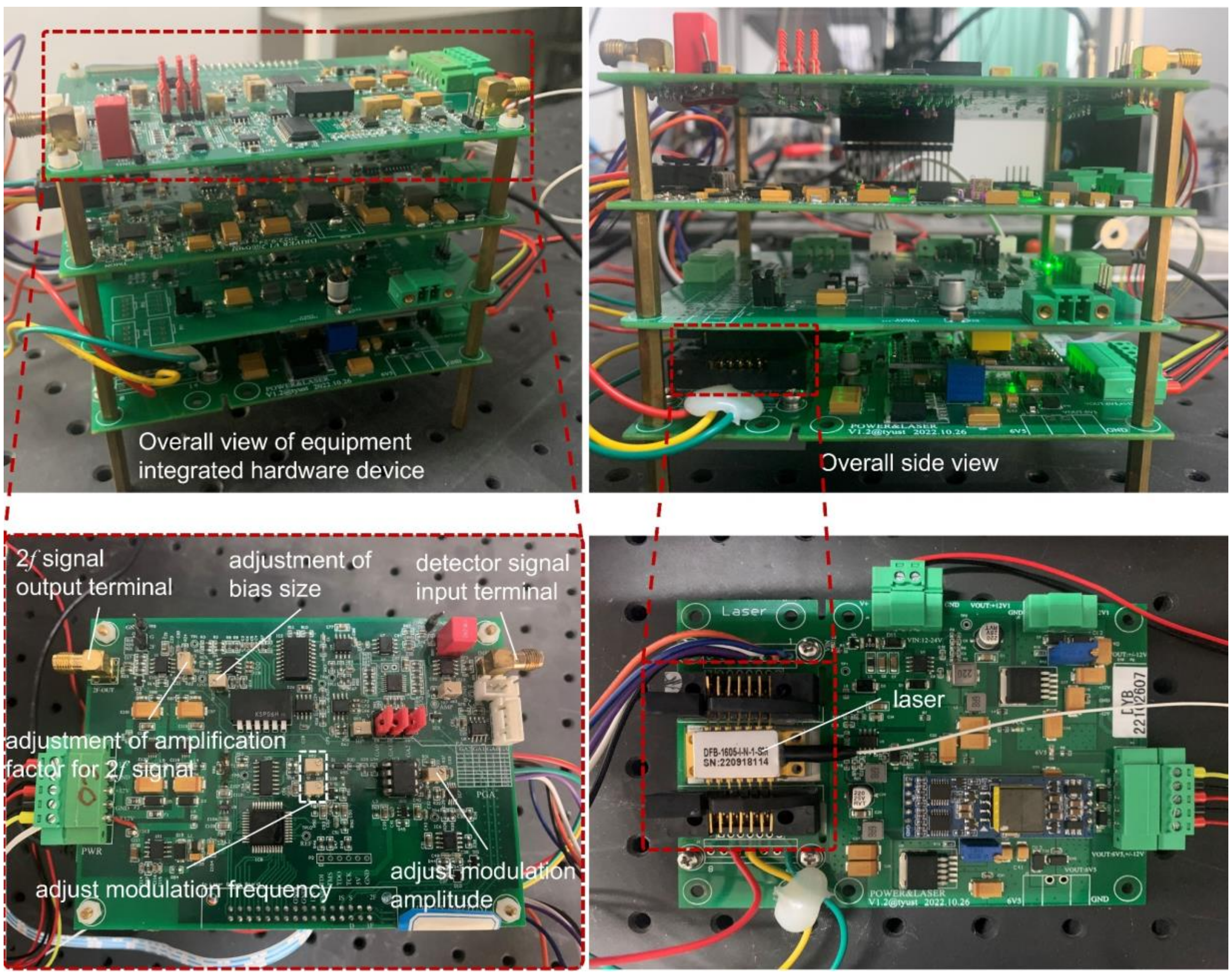

Fig. R14. Integrated electronic system component.

5.2.3 Overall structure of sensing system

As illustrated in Fig. R15 (now included in **Supplementary Note 8** as **Fig. S17**), the miniaturized electronic integration module and the packaged microcavity device have been successfully combined into a fully functional sensor prototype housed within a compact enclosure measuring 26 cm × 16 cm. This integrated system is capable of modulating the WGM resonance

signals and then demodulating them into $2f$ signals for real-time data acquisition.

These results confirm that the cart-based configuration was intended for principle validation. Concurrently, the successful development of the integrated prototype demonstrates that the dissipative WGM sensing approach is fully compatible with compact packaging and portable deployment. No fundamental technical barriers prevent further miniaturization, establishing a clear pathway toward a field-deployable system.

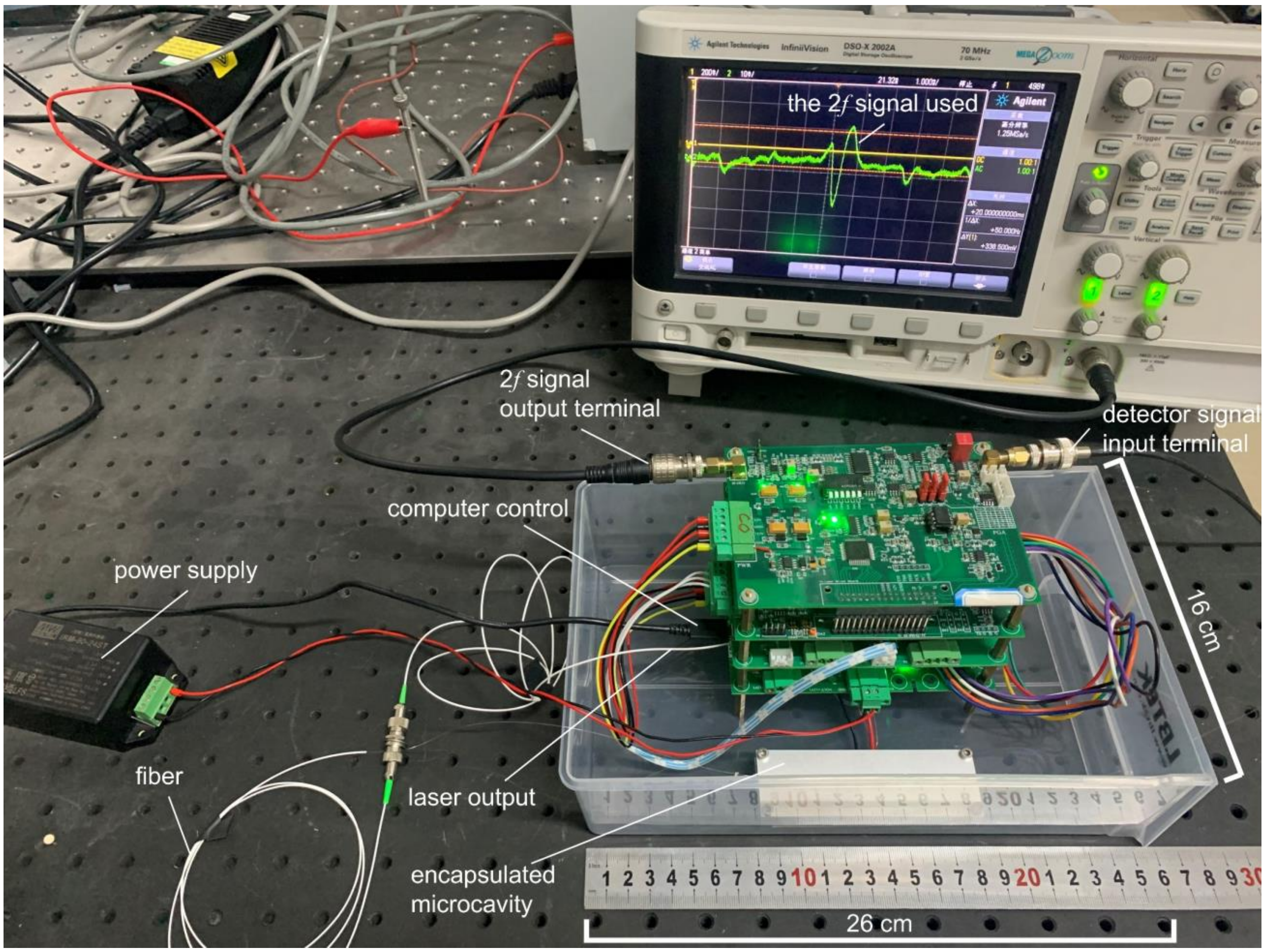

Fig. R15. Integrated sensor system.

We have added relevant discussions in the revised supplementary information (**Supplementary Note 8**) as:

Path Towards System Miniaturization and Integration

The experimental system described in the main text was assembled using discrete commercial bench-top instruments mounted on a mobile laboratory cart. This configuration was intentionally adopted to provide a flexible and well-controlled platform for proof-of-principle validation and

rigorous performance benchmarking of the dissipative sensing mechanism. The use of high-performance commercial instruments ensures reliable operation and enables accurate evaluation of the intrinsic performance limits of the proposed approach. Therefore, the cart-based configuration should be regarded as a verification platform rather than the final integrated implementation of the sensor.

Importantly, the sensing principle demonstrated in this work does not rely on bulky instrumentation. The essential functional modules can be consolidated through circuit-level integration and optical packaging. Based on this consideration, we have carried out system integration at the levels of microcavity packaging, electronic consolidation, and overall system assembly, establishing a practical pathway toward miniaturization and portability.

At the optical level, the standalone WGM microcavity has been packaged into a mechanically robust and miniaturized module. The packaging provides stable structural support and reduces susceptibility to external mechanical perturbations, thereby maintaining a stable coupling condition. Temperature regulation and humidity control are incorporated to ensure long-term resonance stability and measurement repeatability. Compared with the optical platform used in the initial experiments, the packaged microcavity module exhibits a significantly reduced footprint. Detailed structural information is provided in **Supplementary Note 4**.

At the electronic level, the functionalities previously provided by separate commercial instruments-including the laser current and temperature controller, function generator, lock-in amplifier, photodetector, and data acquisition card-have been consolidated onto a custom-designed integrated circuit board. This board-level system combines analog and digital circuitry to implement laser driving and frequency modulation, signal amplification and conditioning, synchronous demodulation, and data acquisition. The integrated electronic module substantially reduces system size and complexity while preserving the core measurement capability. A photograph of the integrated electronic system is shown in Supplementary Fig. S16.

At the system level, the packaged microcavity module and the integrated electronic module have been assembled into a complete prototype housed within a compact enclosure measuring 26 cm×16 cm, as illustrated in Supplementary Fig. S17. The integrated system performs frequency modulation of the probe laser and synchronous demodulation to extract the $2f$ signal, enabling real-time data acquisition and processing. Using this miniaturized configuration, we successfully acquired the $2f$ signal corresponding to atmospheric CO_2 absorption modes, demonstrating stable sensing performance under the integrated architecture.

These results demonstrate that the cart-based configuration presented in the main text was

primarily intended for methodological validation and performance benchmarking. The successful realization of the integrated prototype confirms that the proposed sensing approach is fully compatible with circuit-level consolidation and compact system packaging. No fundamental technical barrier limits system miniaturization, and the demonstrated prototype establishes a practical pathway toward portable and field-deployable implementations.

Reply to Reviewer #3

The manuscript titled "Sub-ppb CO_2 Detection based on Dissipative Whispering Gallery Mode Microcavity Sensor" presents a non-functionalized dissipative gas sensor utilizing a silica microsphere Whispering Gallery Mode (WGM) cavity, with CO_2 detection employed as a representative case to demonstrate its sensing capability. The device employs a tapered fiber to excite the resonant modes of the microcavity; by leveraging intracavity field enhancement, localized heating in the coupling region is amplified, leading to significant modulation of the coupling strength—enabling high sensitivity to variations in CO_2 concentration. Furthermore, the high Q-factor of the WGM microcavity enhances the thermal effects arising from weak gas absorption. The dissipative sensing mechanism inherently suppresses interference from laser frequency fluctuations, and when combined with modulation-demodulation techniques, effectively mitigates low-frequency intensity noise—overcoming the challenge posed by the inherently weak near-infrared absorption of CO_2. The study demonstrates that, operating in the near-infrared regime, the sensor achieves performance comparable to mid-infrared counterparts, attaining a detection limit at the parts-per-trillion (ppt) level, thereby showing strong potential for trace gas sensing applications. Despite these promising results, several critical aspects require major revisions to improve the clarity, completeness, and scientific rigor of the work:

We thank the reviewer for the careful evaluation of our manuscript and for providing constructive comments. We appreciate the reviewer's positive assessment of the potential of our dissipative sensing approach and the achievements in sensitivity. Below, we provide a point-by-point response to the comments. We thank the reviewer for motivating these improvements.

Comment 1: *The manuscript indicates that CO_2 absorption induces localized temperature changes near the microcavity, affecting both the resonance wavelength and the coupling efficiency. Given the claimed high sensitivity, it is imperative to clarify how ambient temperature stability was maintained during experiments. Specifically, was a thermostatically controlled environment used? Detailed information on temperature stabilization protocols must be provided to substantiate the reliability of the reported measurements.*

Answer 1: As illustrated in Fig. R16, we employed a **dual-stage temperature control strategy** to ensure high thermal stability during the experiment. **Stage 1** involves controlling the temperature of the copper sleeve using a thermistor mounted on its side for feedback control. **Stage 2** regulates the aluminum enclosure to stabilize the air temperature within the microcavity chamber. By utilizing a high-precision PID controller, the temperature stability of the air inside the chamber is maintained within an extremely low fluctuation range.

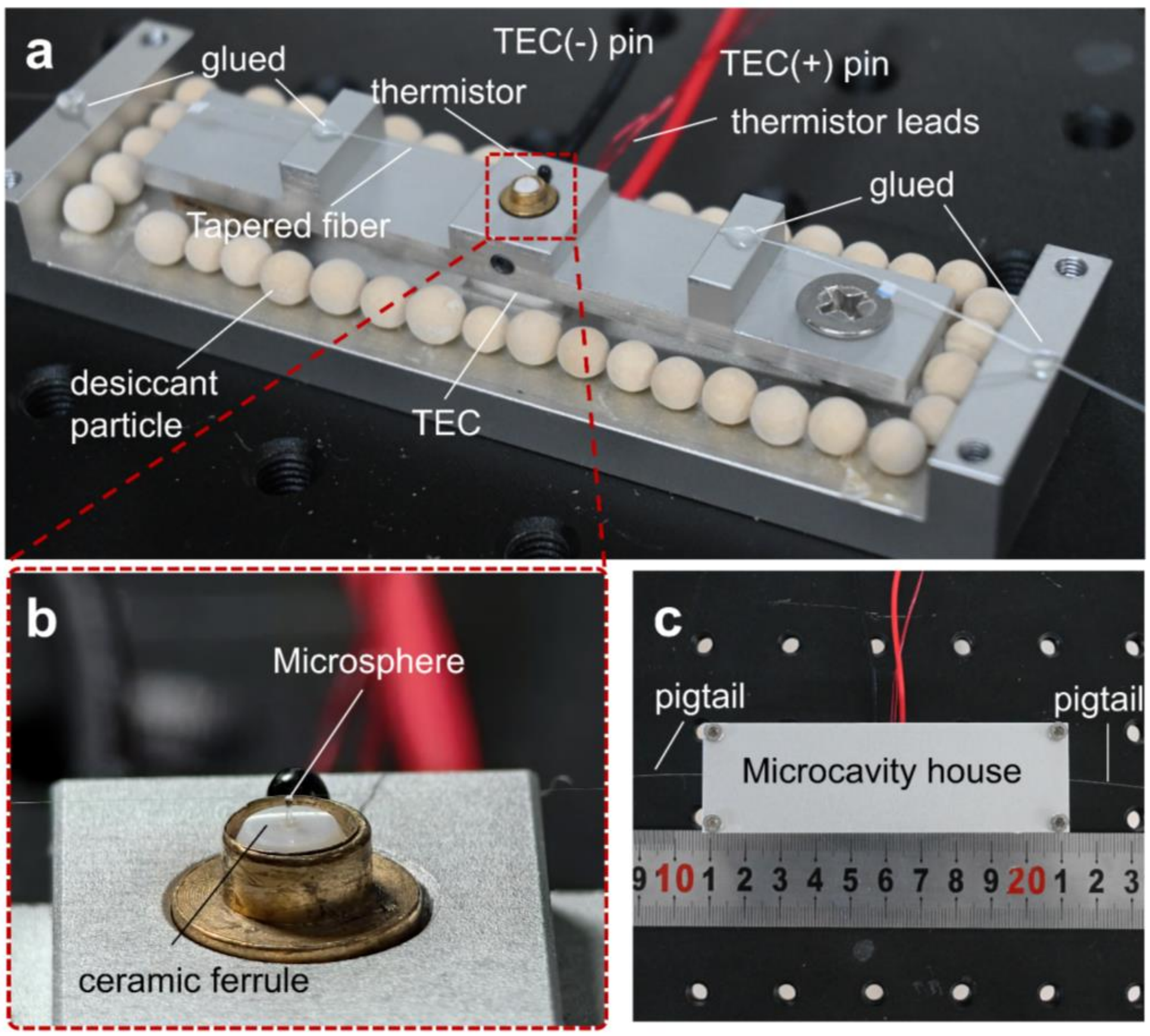

Fig. R16. a, Packaging structure of tapered fiber coupled with microsphere WGM microcavity. **b**, Zoom of the red dashed square in **a**. **c**, Photograph of the fully packaged sensor device.

During operation, the gas absorption-induced heating of the microcavity reaches thermal equilibrium through heat diffusion with the surrounding environment. We modeled and analyzed in detail the gas absorption-induced heating, and the additional description has been included in the revised Supplementary Information (**Supplementary Note 1.4**).

First, starting from the Beer-Lambert law, we derived the relationship between heating power, gas concentration, and light intensity under the low-concentration approximation [Eqs. (S13)-(S18)

in Supplementary Note 1.4]. Then, under the adiabatic approximation, we estimated that the temperature increase induced by gas absorption at a concentration of 1 ppm is ~0.04 K/s. Considering heat conduction, we used the finite element numerical method to model heat transfer in the tapered fiber and microsphere microcavity coupling system based on the standard heat conduction equation. The calculation results show that, due to heat diffusion into the microsphere, tapered fiber, and surrounding air, the temperature increase of the microsphere induced by a gas concentration of 3 ppm is 0.0012 K. Furthermore, we found that the absorbed energy effectively heats the entire microsphere, and most of the heat dissipates into the surrounding air rather than conducting along the tapered fiber. This is due to the extremely small cross-sectional area of the tapered fiber, which limits the efficiency of heat transfer. This point is crucial because it guided our analysis of the contribution of coupling-length variations. The analysis in Supplementary Note 1.3 indicates that the change in coupling length primarily depends on the thermal expansion of the microsphere rather than the tapered fiber itself.

To evaluate the impact of environmental temperature fluctuations on sensor performance, we measured the signal variation as the ambient temperature was ramped from 27°C to 42°C. As shown in Fig. S11 (Supplementary Note 4), the signal fluctuation remained within 1%, demonstrating the robustness of our sensor.

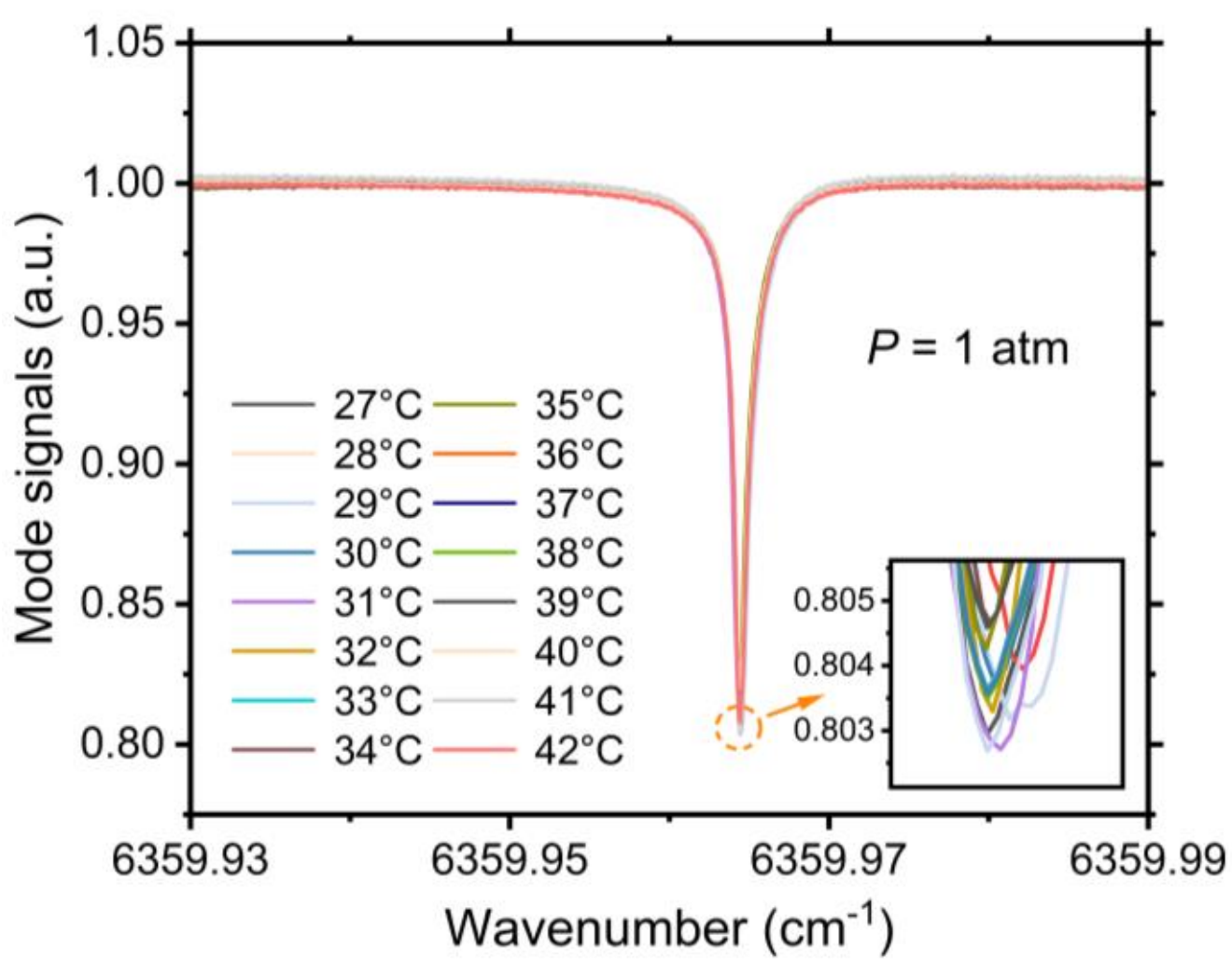

Fig. S11 | Raw optical mode signals for 400 ppm CO_2-N_2 measured over 27-42°C.
On page 23.

Comment 2: *While the authors state that spontaneous thermal noise is negligible, the influence of real-world environmental variables remains inadequately addressed. Clarification is needed regarding: (i) the susceptibility of signal detection to external factors such as ambient light intensity and weather conditions; and (ii) the impact of ambient temperature fluctuations on key sensor metrics—including sensitivity, response/recovery dynamics, and baseline stability. A thorough discussion of these effects is essential to assess the sensor's practical deployment feasibility.*

Answer 2: As discussed in the above answer, real-world environmental variables primarily affect sensor signals through temperature fluctuations. By deploying a dual-stage temperature control strategy, our system effectively mitigates the impact of these external thermal perturbations. Experimental measurements demonstrate that under a wide ambient temperature variation of ~15 K, the fluctuation in sensor readings is suppressed to within 1%. This level of stability satisfies the error tolerance typically required for commercial gas detection systems.

On the other hand, humidity and dust particles in real-world environments can compromise the long-term lifespan of the sensor. To address this, we implemented device-level packaging (as shown in **Fig. R16 in Comment 1**). This packaging maintains a clean and dry micro-environment around the microsphere cavity, effectively preserving its high-Q properties and ensuring sustained long-term performance without degradation (Details can be found in **Comment 4** and **Comment 5**).

We have added the relevant discussion has also been added to revised manuscript.

"Our device-packaged sensor incorporates a dual-stage temperature control system. This system effectively mitigates the impact of temperature fluctuations in the ambient environment on the sensor's readouts (see Supplementary Note 4). Within the thermally stabilized microcavity house, during sensor operation, the heat generated as the laser frequency scans across the WGM resonance is mainly localized to the region surrounding the microcavity and the tapered fiber. Consequently, the WGM coupling strength primarily responds to this localized temperature changing, demonstrating low susceptibility to temperature variations occurring in the broader surroundings. Additionally, some desiccants are placed inside the microcavity house to filter out water vapor from the environment, thereby ensuring the long-term stability and working life of the sensor." On page 18.

We have added the relevant discussion to Supplementary Note 4:

"To enhance the robustness of the WGM microcavity sensor, we fabricated a device package

for the microsphere cavity with the tapered fiber, as shown in Fig. S10. First, the microsphere stem was inserted into a standard single-mode fiber ceramic ferrule, with only a small portion of the stem and microsphere exposed. This effectively suppressed forced vibrations of the microsphere caused by external forces, improving the mechanical stability of the microsphere microcavity.

The entire ceramic ferrule was tightly encased in a copper sleeve, with thermal grease filling the gaps between them. The copper sleeve was then mounted onto an aluminum bracket. A thermistor was attached to one side of the copper sleeve for first-stage of temperature control. The first stage of temperature control primarily acts on the small volume near the microsphere, resulting in better local temperature stability. After optimizing the coupling position of the tapered fiber to the microcavity, the tapered fiber was fixed to the aluminum base with low-thermal-expansion UV glue. Multiple glue points were used along the fiber axis to dampen the effects of external vibrations on the coupling region. At this point, the microcavity and tapered fiber form a single integrated structure, greatly improving the mechanical robustness of the coupling. Finally, the aluminum bracket was placed in an aluminum box (microcavity enclosure), with the pigtails at both ends of the tapered fiber extending out from the sides. The entire aluminum box undergoes independent second-stage temperature control. A precision PID servo controller ensured that the internal air temperature of the aluminum box remained stable with an extremely low fluctuation range.

This microcavity enclosure has not undergone airtight encapsulation, allowing ambient CO_2 to diffuse into the enclosure through the fiber feedthrough openings. To improve the long-term operating time of the sensor, desiccant particles with a nanoporous surface structure are placed inside the microcavity enclosure to absorb water vapor from the external environment, while exhibiting negligible adsorption toward CO_2 under the operating conditions. Therefore, it can maintain long-term dryness inside the microcavity enclosure, reducing the rate of decrease in the Q factor of the microsphere WGM microcavity.

To evaluate the influence of slow thermal fluctuations in the external environment on signal amplitude, measurements were performed on 400 ppm CO_2-N_2 mixtures in a sealed chamber under different temperature conditions. The chamber temperature was controlled using an external heating tape coupled with a PID temperature controller for precise regulation. In the experiment, the temperature was stepped in 1° C increments, and the system was allowed to stabilize for 5 min each setpoint before acquiring the raw optical mode signals. Each measurement was averaged over 200 acquisitions to improve the signal-to-noise ratio. Supplementary Fig. S11 shows the raw mode signals obtained over the 27-42° C range. Because the packaged sensor device contains a

dual-stage temperature control system, it can effectively resist temperature changes in the external environment. As shown in Fig. S11, it indicates that the signals remain highly consistent across all temperatures, with amplitude deviations within 1%, demonstrating that slow environmental temperature changes have minimal impact on the mode signal amplitude and confirming the system's stability under external thermal perturbations." On page 21.

Comment 3: *Although the proposed WGM-based platform is suggested to be applicable to various trace gases with near-infrared absorption features (e.g., CO_2, CH_4, NH_3, H_2S, H_2), the issue of gas selectivity—a fundamental requirement for reliable operation in complex environments—is not discussed. This omission undermines the sensor's credibility and applicability. The authors are strongly encouraged to include an evaluation of selectivity through experimental comparisons with interfering gases, computational modeling (e.g., density functional theory), or other appropriate methods.*

Answer 3: We thank you for emphasizing the critical importance of gas selectivity for reliable sensing in complex environments. You are correct that this is a fundamental requirement. We address this key issue through rigorous experimental validation of the sensor's intrinsic spectroscopic selectivity, which is fundamentally different from the material-based selectivity of functionalized coatings.

The selectivity of our sensor is not derived from a functionalized coating, but rather from spectroscopic selectivity inherent to the gas molecules themselves. Our dissipative sensing scheme operates by locking a tunable laser to a wavelength that coincides with a characteristic vibrational or rovibrational absorption line of a specific target gas (e.g., CO_2 at 1.572 μm, CH_4 at 1.651 μm). Only when the laser wavelength overlaps with a gas's absorption feature does the absorption-induced change in the cavity's optical loss (manifested as a resonance depth change) become significant. The narrow linewidth of the laser enables precise locking onto the target absorption line, ensuring immunity to interference from other gases. This principle forms the very basis of our method and provides its intrinsic selectivity.

To rigorously demonstrate this, we performed a series of cross-sensitivity experiments with gas mixtures. The sensor platform (a bare, non-functionalized WGM microsphere) was placed in a sealed chamber. We used three near-infrared lasers, which can operate at the absorption bands of CO_2, CH_4 and C_2H_2 respectively, and measured a series of mixed gases with different

concentrations through the precision gas mixing system. The balance gas in the chamber was high-purity N_2.

3.1. Selectivity Test with CO_2-Absorption Laser

Using a laser locked at the CO_2 absorption band (~1.572 μm), we varied only the CO_2 concentration (400 ppm, 200 ppm, 50 ppm and 25 ppm) while holding the concentrations of CH_4 and C_2H_2 constant. A clear and corresponding change in the resonance depth was observed, as shown in Fig. S15a. Conversely, when the CO_2 concentration was held constant and the concentrations of CH_4 and C_2H_2 were varied independently, no significant change in the raw optical mode signal was detected by the CO_2-tuned laser, as shown in Fig. S15d.

3.2. Control Experiments with Other Gases

The same experiment was repeated using lasers tuned to the characteristic absorption bands of CH_4 (~1.651 μm) and C_2H_2 (~1.54 μm). The results were in perfect agreement with the sensing principle:

With the CH_4-tuned laser, the raw optical mode signal changed only when the CH_4 concentration was varied, remaining unaffected by changes in CO_2 and C_2H_2 concentrations, as shown in Figs. S15b and S15e.

With the C_2H_2-tuned laser, the raw optical mode signal changed only when the C_2H_2 concentration was varied, remaining unaffected by changes in CO_2 and CH_4 concentrations, as shown in Figs. S15c and S15f.

These controlled experiments provide conclusive evidence that the sensor’s response is exclusively tied to the concentration of the gas whose absorption line matches the laser wavelength. These results collectively confirm that the sensing response originates from wavelength-specific molecular absorption rather than nonspecific environmental perturbations.

Regarding the field demonstration, the observed diurnal fluctuation pattern is strongly correlated with known anthropogenic activity and is fully consistent in magnitude and trend with expected CO_2 dynamics. While we acknowledge that other gases can also exhibit diurnal cycles, the combination of this real-world correlation and, more decisively, the controlled laboratory mixture experiments described above provides a robust argument for the selective detection of CO_2 in our demonstration. We performed additional CO_2 measurements over two consecutive days, with parallel measurements taken using a commercial-grade, calibrated CO_2 analyzer. The results showed good agreement.

In summary, our sensor achieves selectivity through wavelength-specific optical absorption, a complementary approach to the material-specific chemical affinity used in polymer-coated

sensors. We have now added a detailed description of these selectivity experiments to the revised manuscript to explicitly address this key point. These results collectively confirm that the sensing response originates from wavelength-specific molecular absorption rather than nonspecific environmental perturbations, thereby validating the intrinsic spectroscopic selectivity of the proposed dissipative WGM sensing mechanism.

We have added Fig. S15 to the revised supplementary materials:

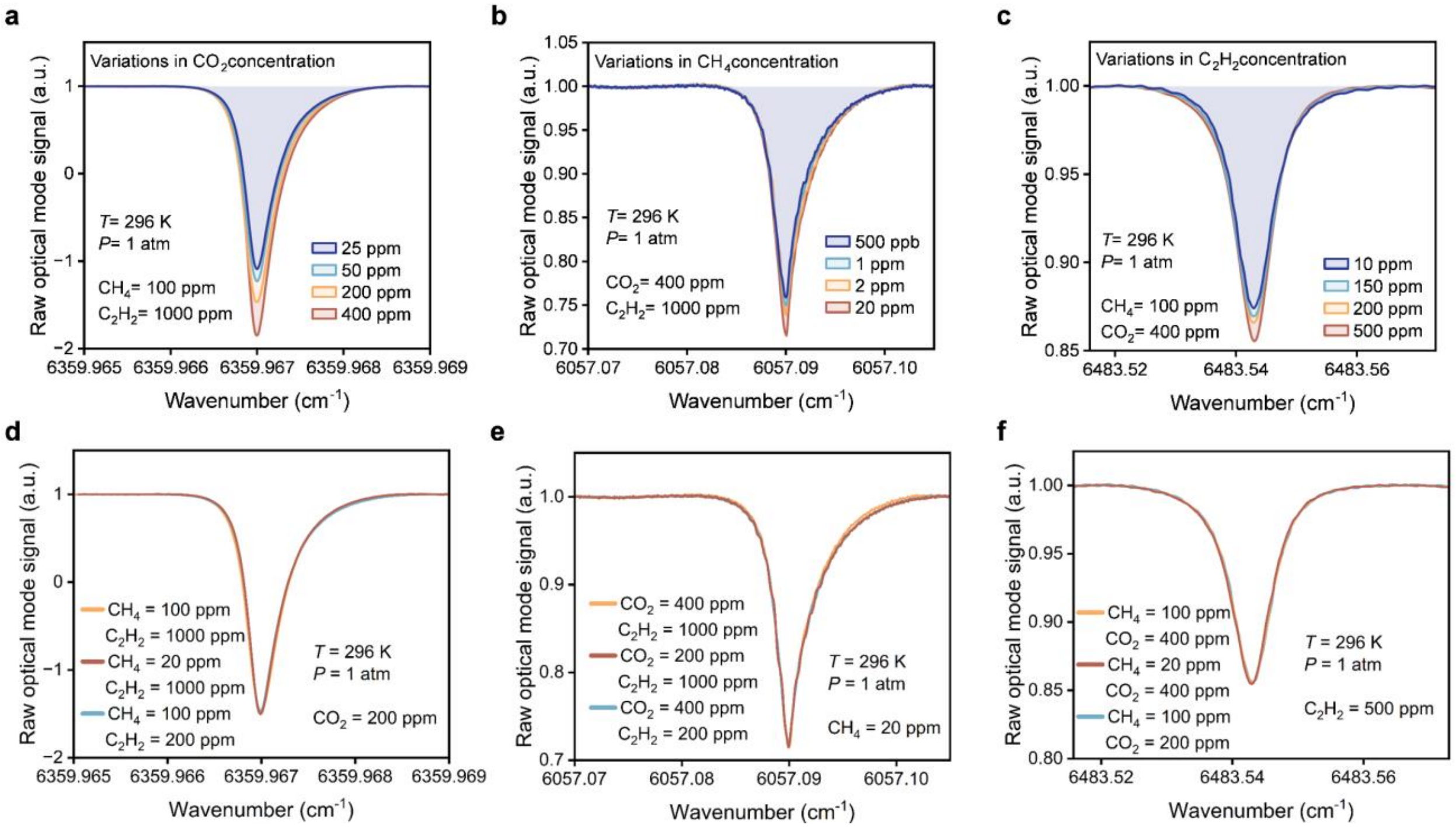

Fig. S15 | Verification experiment of gas selectivity. a, Variation of the raw optical mode signal of CO_2 as a function of CO_2 concentration measured in CO_2-C_2H_2-CH_4 gas mixtures with fixed C_2H_2 and CH_4 concentrations. **b**, Variation of the raw optical mode signal of CH_4 as a function of CH_4 concentration measured in CO_2-C_2H_2-CH_4 gas mixtures with fixed C_2H_2 and CO_2 concentrations. **c**, Variation of the raw optical mode signal of C_2H_2 as a function of C_2H_2 concentration measured in CO_2-C_2H_2-CH_4 gas mixtures with fixed CH_4 and CO_2 concentrations. **d**, Response of the raw optical mode signal of CO_2 to varying concentrations of CH_4 and C_2H_2, respectively, under a constant CO_2 concentration. **e**, Response of the raw optical mode signal of CH_4 to varying concentrations of CO_2 and C_2H_2, respectively, under a constant CH_4 concentration. **f**, Response of the raw optical mode signal of C_2H_2 to varying concentrations of CH_4 and CO_2, respectively, under a constant C_2H_2 concentration. On page 32.

We have added relevant discussions in the revised supplementary information (**Supplementary**

Note 7) as:

"Gas Selectivity of the Dissipative WGM Sensor

The selective detection of a target gas within complex mixtures is a fundamental requirement for practical sensing. This supplementary section provides a detailed experimental demonstration and theoretical rationale for the intrinsic gas selectivity of our spectroscopic dissipative sensing platform, which operates on a principle distinct from functionalized coating-based sensors.

7.1 Principle of Spectroscopic Selectivity

The core mechanism enabling selectivity in this work is wavelength-specific optical absorption by gas molecules, a fundamental molecular property. The sensor employs a high-Q WGM microcavity as a sensitive probe for optical absorption by gas. A tunable diode laser is precisely tuned to a wavelength corresponding to a characteristic, narrow-linewidth electronic or vibrational absorption line of a specific target gas (e.g., CO_2 at 1.572 μm, CH_4 at 1.651 μm, C_2H_2 at 1.54 μm). When the laser wavelength spectrally overlaps with the target gas absorption feature, molecules present in the evanescent field of the optical mode absorb photons. Our approach leverages enhanced local optical fields and thermal effects at the coupling region to convert weak absorption into measurable variations in resonance depth. Crucially, gases lacking absorption features at the specific laser wavelength do not contribute to this resonant absorption change, thereby conferring inherent selectivity directly derived from molecular spectroscopy.

7.2 Experimental Validation via Controlled Cross-Sensitivity Tests

To rigorously validate this principle and rule out significant cross-interference, a series of controlled experiments was conducted using gas mixtures. An uncoated microsphere resonator was housed in a sealed gas cell with precise temperature and pressure control. Three distributed feedback (DFB) diode lasers were employed, each centered at the absorption line of a different target gas: CO_2, CH_4, and C_2H_2. Certified standard gases and a computerized multi-channel gas mixing system (Environics Series 4000) were used to generate mixtures with accurately defined concentrations, with high-purity N_2 serving as the balance gas.

7.2.1 Selective Response to Target Gas Variation

For each target gas, the corresponding laser was activated and locked to its absorption line. The concentration of the target gas was systematically varied (e.g., CO_2: 25, 50, 200, 400 ppm), while the concentrations of the other two potential interferent gases were maintained at constant, non-zero levels. As shown in Supplementary Fig. S15a-c, a strong, monotonic dependence of the resonance depth change of the raw optical mode signal on the target gas concentration was observed for each laser-gas pair. This demonstrates the sensor's primary sensitivity to the intended

analyte.

7.2.2 Insensitivity to Non-Target Gas Variation (Cross-Interference Test)

The converse experiment was critical for proving selectivity. For a given laser (e.g., the CO_2-absorbing laser at 1.572 μm), the concentration of its target gas (CO_2) was held constant. Subsequently, the concentrations of the other gases (CH_4 and C_2H_2) were independently varied over a comparable concentration range. The results, summarized in Supplementary Fig. S15d-f, show no significant change in the measured resonance depth of the raw optical mode signal. The observed fluctuations remained within the baseline noise level of the sensor system. This confirms that the presence and varying concentration of gases not absorbing at the specific wavelength do not elicit a measurable response.

7.3 Discussion and Conclusion

The combined dataset from these controlled mixture experiments provides strong experimental evidence for the spectroscopic selectivity of the dissipative sensing mechanism. The sensor's response function is primarily governed by the Beer-Lambert law interaction between the spectrally narrow probe laser and the specific gas species possessing a resonant absorption at that wavelength. This approach fundamentally differs from polymer-coated WGM sensors, which rely on the partitioning of analytes into a functionalized film based on chemical affinity.

The real-world field demonstration of CO_2 monitoring, showing plausible diurnal variations, gains robust contextual support from these laboratory selectivity tests. While atmospheric diurnal patterns are not uniquely specific, the conjunction of field data with known anthropogenic drivers, and more definitively, the laboratory-proven absence of response to common potential interferents like CH_4 under the CO_2-measurement configuration, strongly supports the interpretation that the recorded signal mainly originates from CO_2 concentration changes.

In summary, the selectivity of this sensor platform is intrinsically rooted in the discrete absorption spectra of gas molecules. This method offers a powerful and complementary alternative to material-based selective coatings, particularly advantageous for applications requiring the detection of small molecules with well-defined, narrow absorption features in the near-infrared region." On page 29.

Comment 4: *The CO_2 sensing tests were conducted in a nitrogen matrix, which deviates significantly from standard characterization practices. A clear justification for this choice is*

required, including the scientific rationale for using pure N_2 as the carrier gas. Moreover, the sensor's compatibility with more realistic atmospheric conditions—such as ambient air containing oxygen, humidity, and other background constituents—should be explicitly evaluated and discussed to support claims of practical utility.

Answer 4: We thank you for these insightful and critical points regarding the experimental matrix and environmental robustness, which are essential for assessing practical applicability. We appreciate the opportunity to clarify our methodological choice and to present additional data addressing performance in realistic atmospheric conditions.

4.1 Reasons for using nitrogen matrix

In this study, high-purity N_2 was employed as the background gas during the initial calibration and fundamental selectivity evaluation stages. This methodology is consistent with established practices in high-precision absorption spectroscopy and microcavity sensing. In related research, inert, dry, and spectroscopically "quiet" N_2 is routinely used to establish system baselines under interference-free conditions. This approach excludes non-target influences and enables quantitative characterization of the intrinsic sensor response to target analytes. This strategy facilitates the effective isolation of molecule-specific absorption contributions, allowing accurate determination of key performance indicators such as sensitivity and detection limit, which are central objectives of the preliminary phase of this investigation [Ref: *Anal. Chem.* **97**, 18152-18158 (2025)] [Ref: *Nat. Commun.* **15**, 8761 (2024)].

It should be noted that for conventional absorption spectroscopy techniques employing direct absorption spectral signals as the sensing metric, variations in background gas composition can indeed affect absorption line profiles owing to differences in collisional broadening coefficients among molecular species. This, to a certain extent, constrains the direct transferability of calibration results obtained with N_2 dilution to ambient atmospheric conditions. However, the sensing mechanism adopted in the present work does not rely on precise fitting of absorption line shape parameters; instead, it leverages changes in microcavity resonance modes induced by heat deposition resulting from gas absorption as the core transduction principle. The magnitude of this thermal effect is governed primarily by the absolute number density of target molecules and their absorption cross-section, and is largely independent of non-absorbing background species present in the system.

Consequently, within this dissipative whispering-gallery-mode (WGM) sensing framework,

the calibration relationship established using high-purity N_2 as the diluent and calibration gas exhibits good transferability and can therefore be applied to CO_2 concentration measurements under realistic atmospheric conditions.

4.2 Explicit Evaluation with Realistic Atmospheric Conditions: Humidity Effects

We fully agree with the reviewer that explicit testing with realistic atmospheric components, especially water vapor, is crucial. To this end, we conducted a dedicated investigation into the influence of humidity on the WGM microresonator itself, independent of gas absorption.

The bare microsphere was placed in a temperature- and humidity-controlled chamber. We monitored the resonance wavelength and depth while varying the relative humidity (RH) from 30% to 50% at constant temperature, as shown in Fig. S18. The result shows a consistent redshift of the resonance wavelength on the order of several picometers (corresponding to ~0.16 cm^{-1} in wavenumber) with increasing RH, accompanied by minor fluctuations in resonance depth. This is attributed to the adsorption of water molecules onto the silica surface, altering the local refractive index and possibly introducing additional scattering loss.

The whispering gallery mode microspheres fabricated from electrically fused silica single-mode fibers exhibit a resonance wavenumber shift of only approximately ± 0.16 cm^{-1} under relative humidity fluctuations of ±10% RH. This value is significantly lower than the theoretical prediction (approximately ±1.62 cm^{-1}) based on a fully hydrophilic silica surface model.

The humidity sensitivity of whispering gallery mode optical microcavities originates from the adsorption of water molecules on the cavity surface, which alters the effective refractive index within the evanescent field region. For a typical hydrophilic silica surface, its abundant silanol groups serve as highly effective adsorption sites for water molecules. Theoretical predictions, usually based on this model, estimate a relatively high humidity sensitivity (on the order of tens of pm/%RH). However, the sensitivity observed in this experiment (approximately 3.95 pm/%RH after conversion) is significantly lower than this expectation, implying a fundamental alteration in the surface properties of the device.

4.3.1. Mechanism analysis: the core role of electrofusion high temperature treatment

The extremely high temperatures experienced during the electrical fusion fabrication process are key to the transformative reshaping of the microsphere's surface properties. This reshaping significantly weakens its interaction with water molecules through the following two synergistic mechanisms:

(1) Surface Chemical Modification: dehydration and condensation of silicon hydroxyl

In a high-temperature environment, irreversible dehydration condensation reaction occurs

between adjacent silanol groups (Si–OH). This reaction converts the high-density, hydrophilic silanol groups on the surface into hydrophobic siloxane bridges. The exponential decrease in the surface concentration of silanol groups directly leads to a drastic reduction in the active sites available for the hydrogen-bond-mediated physisorption of water molecules, thereby fundamentally suppressing the formation of multilayer water films.

(2) Thermally induced rearrangement of surface physical structure

In the molten state, the surface atoms migrate and rearrange rapidly driven by surface tension. This process leads to surface smoothing and densification. As shown in the subsequent **Comment 5** by SEM, a denser and more complete surface network structure is formed, which further reduces the physical defects and pores available for water molecule adsorption.

4.4. Sensor device packaging to solve the influence of humidity

From our experiments, it is known that such direct humidity-induced optical mode perturbation constitutes a source of noise and potential drift for gas sensing. It increases measurement uncertainty and poses challenges to long-term stability and sensor longevity. To address this issue directly, we have developed a sealed, dry nitrogen-purged encapsulation for the sensor module, as detailed in our response to **Comment 1**. This encapsulation serves a dual-purpose approach: (i) it maintains a clean, dry, and stable microenvironment around the microsphere, effectively decoupling it from external fluctuations in humidity and particulate matter, thereby preserving stability; (ii) it allows for the controlled introduction of target gases. This design choice directly mitigates the humidity interference issue and ensures high sensitivity and accuracy in the gas measurements.

Under this packaging configuration, the mode drift was measured while the external relative humidity changed from 30% to 50%. As shown in Fig. S18, under the condition of relative humidity fluctuation of ±10% RH, the resonance wave number shift of the WGM mode was only about ±0.01 cm^{-1}, and the mode remained at the peak of the absorption peak, which did not affect the measurement of CO_2.

These discussions and new data have been incorporated into the revised manuscript.

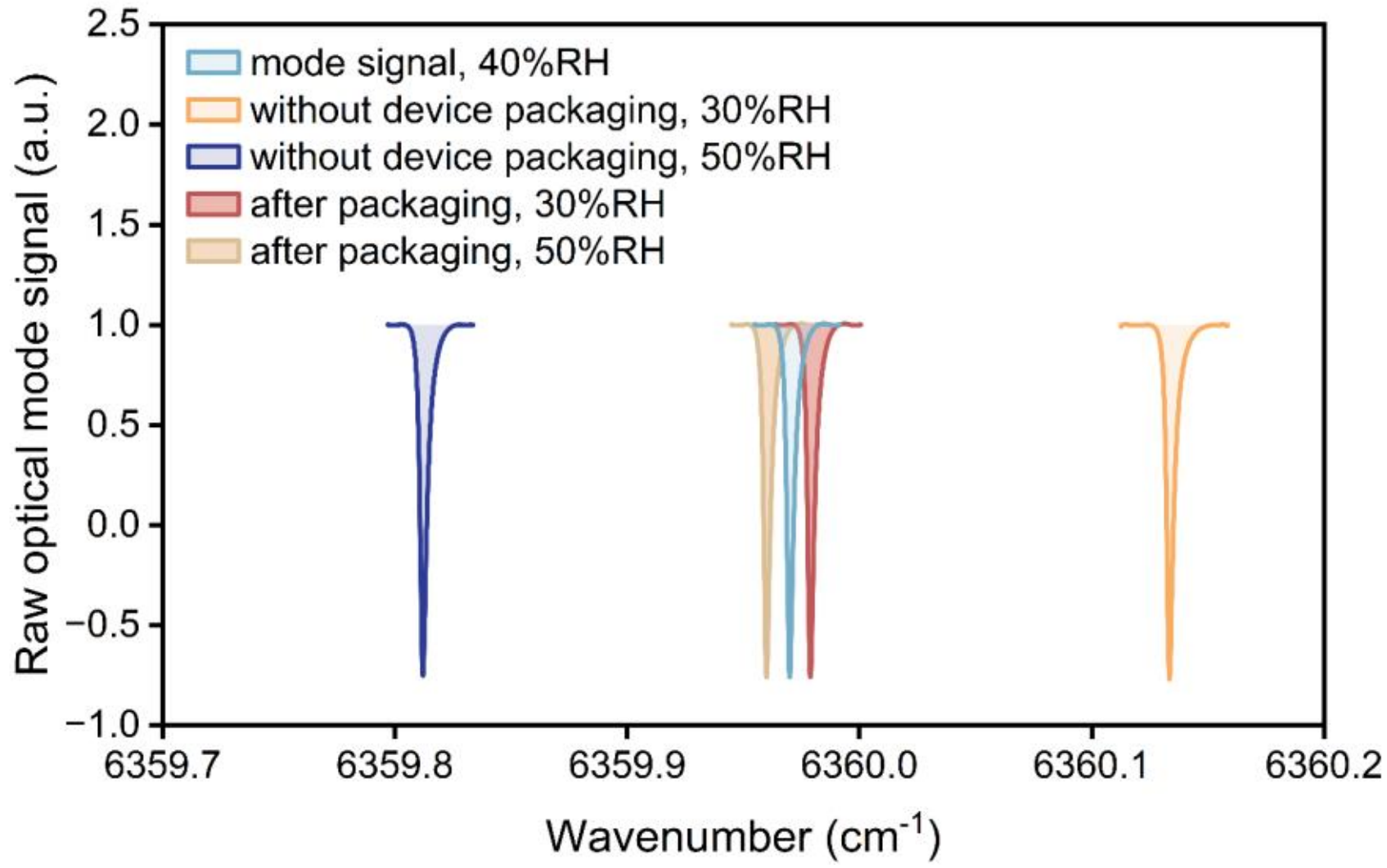

Fig. S18 | Variations of raw optical mode signal under different relative humidity.

We have added the relevant discussion to Supplementary Note 9:

Considerations on Experimental Matrix and Environmental Robustness

This section provides a detailed rationale for the experimental methodologies employed in the sensor characterization and discusses critical investigations into environmental interferents, specifically atmospheric humidity. The aim is to clarify fundamental operating principles and to validate the sensor's potential for operation under realistic conditions.

9.1 Reasons for using nitrogen matrix

In this study, high-purity N_2 was employed as the background gas during the initial calibration and fundamental selectivity evaluation stages. This methodology is consistent with established practices in high-precision absorption spectroscopy and microcavity sensing. In related research, inert, dry, and spectroscopically “quiet” N_2 is routinely used to establish system baselines under interference-free conditions. This approach excludes non-target influences and enables quantitative characterization of the intrinsic sensor response to target analytes. This strategy facilitates the effective isolation of molecule-specific absorption contributions, allowing accurate determination of key performance indicators such as sensitivity and detection limit, which are central objectives of the preliminary phase of this investigation [Ref: *Anal. Chem.* **97**, 18152-18158 (2025)] [Ref: *Nat. Commun.* **15**, 8761 (2024)].

It should be noted that for conventional absorption spectroscopy techniques employing direct absorption spectral signals as the sensing metric, variations in background gas composition can indeed affect absorption line profiles owing to differences in collisional broadening coefficients

among molecular species. This, to a certain extent, constrains the direct transferability of calibration results obtained with N_2 dilution to ambient atmospheric conditions. However, the sensing mechanism adopted in the present work does not rely on precise fitting of absorption line shape parameters; instead, it leverages changes in microcavity resonance modes induced by heat deposition resulting from gas absorption as the core transduction principle. The magnitude of this thermal effect is governed primarily by the absolute number density of target molecules and their absorption cross-section, and is largely independent of non-absorbing background species present in the system.

Consequently, within this dissipative whispering-gallery-mode (WGM) sensing framework, the calibration relationship established using high-purity N_2 as the diluent and calibration gas exhibits good transferability and can therefore be applied to CO_2 concentration measurements under realistic atmospheric conditions.

9.2 Experimental Investigation of Humidity Effects and Surface Phenomena

In contrast to dry gas composition, water vapor presents a significant practical challenge due to its ubiquitous presence and potential for both measurement accuracy and physical perturbation of the microcavity. We conducted a dedicated study to analyze the physical effect of humidity on the WGM microcavity itself.

A silica microsphere was placed in a climate-controlled chamber. As the relative humidity (RH) was varied from 30% to 50% at constant temperature, the WGM resonance exhibited a consistent redshift on the order of several picometers (corresponding to a wavenumber shift of ~±0.16 cm^{-1} for a ±10% RH change), accompanied by minor fluctuations in resonance depth (Supplementary Fig. S18). This shift is attributed to water molecule adsorption altering the effective refractive index in the evanescent field region.

The observed humidity-induced wavenumber shift (±0.16 cm^{-1}) is notably lower than theoretical predictions (often on the order of ±1.6 cm^{-1} or tens of pm/%RH) for a standard hydrophilic silica surface. This discrepancy indicates a fundamental modification of the microsphere surface properties. The key factor is identified as the high-temperature electrofusion process used in microsphere fabrication, which induces two synergistic effects:

Surface Chemical Modification: At extreme temperatures, adjacent surface silanol (Si-OH) groups undergo irreversible dehydration condensation, forming hydrophobic siloxane (Si-O-Si) bridges. This drastically reduces the density of hydrophilic sites available for hydrogen-bonded water adsorption.

Surface Structural Densification: In the molten state, surface atom migration driven by

surface tension leads to smoothing and densification of the silica network, minimizing physical defects and pores that could trap water.

Furthermore, during optical characterization, resonant power buildup within the cavity can generate a localized photothermal effect. This "thermal drying" of the immediate surface region can dynamically counteract adsorption, potentially leading to an observed sensitivity lower than the intrinsic material property under equilibrium conditions.

9.3 Mitigation Strategy: Sensor Packaging

Although the inherent surface hydrophobicity reduces the impact on humidity changes, humidity-induced resonance drift remains a non-negligible noise source for high-precision gas sensing, affecting baseline stability and long-term reliability. To address this, the coupling structure consisting of the tapered fiber and the microsphere WGM microcavity is packaged (detailed in **Supplementary Note 4**). This packaging achieves two critical functions:

This device maintains a constant, dry internal environment around the micro resonator, effectively decoupling it from external humidity fluctuations and particulate contamination, thereby ensuring stability. It allows controlled introduction of analyte gases through a dedicated sampling port. The incoming gas stream passes through desiccant particles before entering the sensing chamber, ensuring a stable and dry local environment for accurate measurements.

Under this packaging configuration, the mode drift was measured while the external relative humidity changed from 30% to 50%. As shown in Fig. S18, under the condition of relative humidity fluctuation of ±10% RH, the resonance wave number shift of the WGM was only about ±0.01 cm^{-1}, and the mode remained within the effective absorption linewidth, which did not affect the measurement of CO_2.

Comment 5: *Stability assessment—encompassing both long-term performance consistency and material durability—is insufficiently addressed. First, the current testing duration is too short to reflect operational longevity required in real-world monitoring (where sensors may operate continuously for months or years). Short-term data cannot reveal degradation phenomena such as baseline drift or sensitivity loss. Second, no characterization of the sensing material's structural or chemical stability (e.g., via XRD, XPS, SEM before and after exposure) is presented, despite its direct impact on sustained performance. It is recommended that: (a) long-term stability tests be extended over a period of 1–3 months with periodic monitoring of key parameters; and (b) post-exposure material analyses be included to evaluate structural integrity and surface stability.*

Answer 5: Thank you for your insightful comments regarding the long-term stability of our sensor.

The gas cell was designed and validated for high hermeticity. The chamber body is stainless steel, and all feedthroughs use ultra-high vacuum (UHV)-compatible, welded or O-ring seals. During all experiments, the chamber was continuously supplied with dry N_2, and the sustained ultra-low baseline drift of the WGM resonance serves as functional proof that external atmospheric perturbations were effectively excluded. This robust sealing created the controlled environment necessary to isolate the sensor's intrinsic drift mechanisms.

In direct response to your recommendation, we conducted a dedicated, **50-day continuous stability assessment** of a non-functionalized WGM microsphere under the aforementioned controlled environment (constant temperature, dry actual atmospheric composition). The resonance depth and linewidth (proportional to Q^{-1}) were monitored continuously.

As shown in Fig. S19, for the 50 days, the sensor demonstrated exceptional stability. The resonance linewidth remained constant within ±1.5%, and the resonance depth showed minimal variation (<1%). The Allan deviation of the frequency signal reached a minimum, indicating that the system noise was dominated by fundamental thermorefractive noise, not by drift. This period represents the sensor's optimal performance window.

Over longer time scales, the Q-factor may exhibit a gradual decline. This is due to mechanical stress relaxation. The mounting structures, including the UV-adhesive-fixed tapered fiber and the silica stem holding the microsphere, undergo nano-scale viscoelastic creep and stress relaxation over extended periods. This slowly alters the critical coupling condition between the fiber and the microsphere, leading to the observed resonance depth shallowing and contributing to low-frequency drift. The 50-day high-stability window validates the effectiveness of our environmental control, while the possible long-term degradation highlights the long-term challenges posed by these intrinsic material and mechanical processes.

Your feedback is invaluable in guiding our development towards practical deployment. Based on these findings, our future work will focus on mitigating these long-term mechanical relaxation effects. We have updated the manuscript to include a summary of this 50-day stability experiment. We thank you again for pushing us to conduct this important investigation, which significantly strengthens the discussion on the sensor's operational limits and future development roadmap.

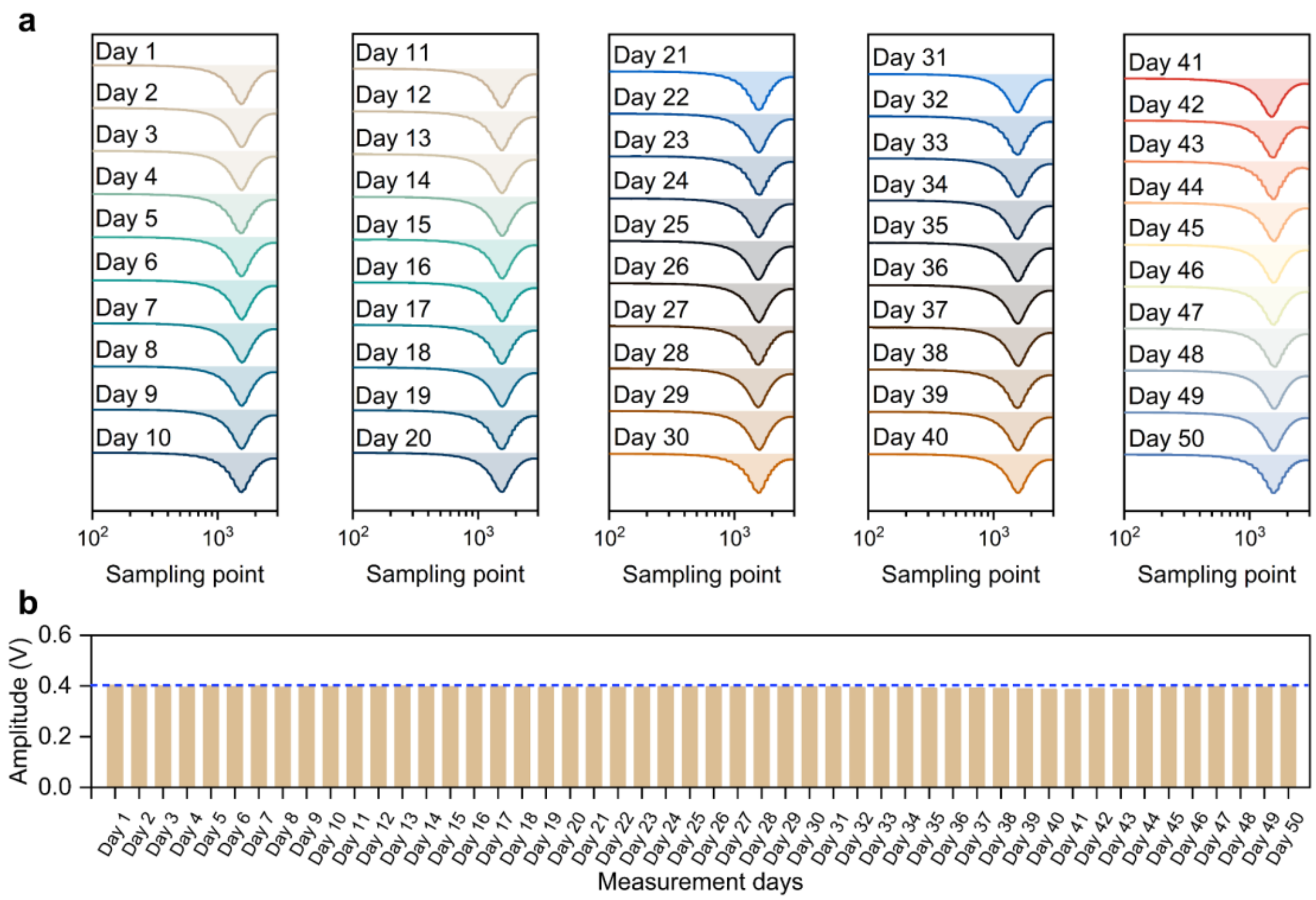

Fig. S19 | **a**, Mode signals measured within 50 days. **b**, Analysis of amplitude of mode signal.

We have added the relevant discussion to Supplementary Note 10:

Extended 50-Day Long-Term Stability Assessment

To rigorously assess the operational longevity and intrinsic drift characteristics of the WGM sensor, a dedicated 50-day continuous stability experiment for ambient air enclosed in a sealed chamber was conducted. As shown in Fig. S19, the sensor exhibited exceptional stability during the 50 days. The resonance linewidth ($\Delta\lambda$) fluctuated within ±1.5% of its initial value, indicating a stable Q factor of the microcavity. The resonance depth showed fluctuation of less than 1%, confirming a stable coupling condition between the fiber taper and the microsphere.

However, the coupling systems for the tapered fiber and the microsphere have inherent mechanical stress relaxation. Therefore, in the future, the depth and linewidth of the mode may change on a longer-term scale, and if such changes occur, recalibration will be necessary.

The mounting scheme, which relies on a UV-adhesive-fixed tapered fiber and a silica stem supporting the microsphere, is subject to long-term viscoelastic effects. Over periods of months, nano-scale creep and stress relaxation within the adhesive and the silica structure can occur. This will slowly change the relative position of the tapered fiber and the microsphere, thus altering the coupling strength. As a result, may alter the coupling strength, affecting the resonance depth and

linewidth.

Based on the fluctuations in the mode signal amplitude measured over the aforementioned 50-day period, the results indicate that the microspheres have successfully completed the aging test, and it can be predicted that their performance will not exhibit significant changes over an extended period of future use. During prolonged operation, if notable variations in mode width or significant deviations in concentration measurements are observed, the system may be recalibrated or the microspheres replaced.

As illustrated in Fig. R17, the fabricated microsphere microcavities possess an extremely smooth and dense surface morphology, which contributes to their structural stability in ambient air. Comparative SEM imaging over a one-month interval revealed negligible surface variations, as shown in Fig. R17b and R17c respectively. Nevertheless, considering that the long-term maintenance of high Q-factors is sensitive to environmental cleanliness and humidity, we have packaged the microspheres, as detailed in our responses to Comments 1 and 2. This packaged device stabilizes the local environment, ensuring that the Q-factor remains preserved without significant degradation during long-term operation.

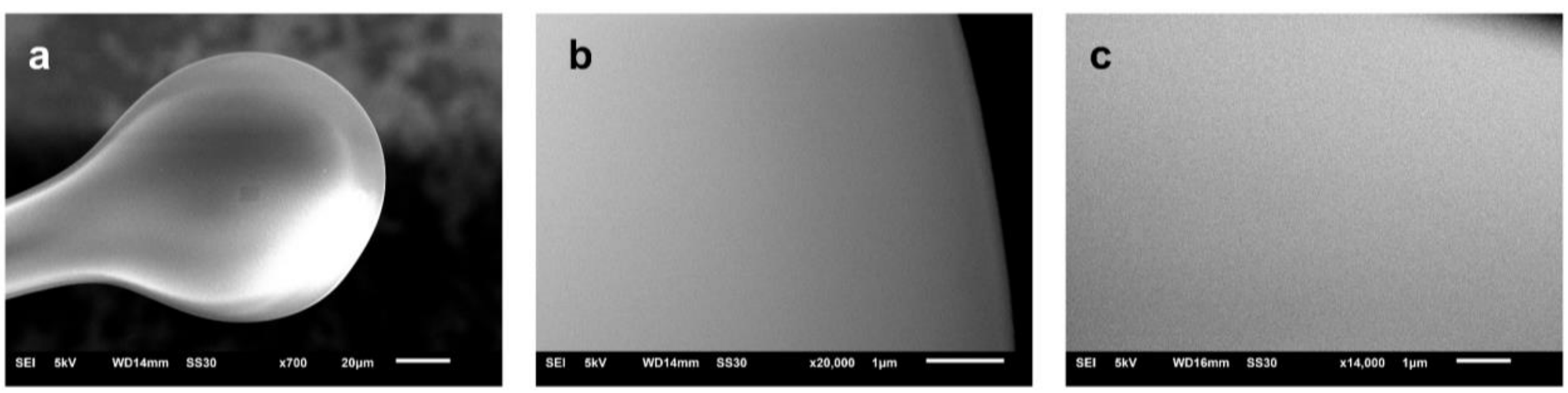

Fig. R17 | **a**, SEM image of a microsphere WGM microcavity. **b**, magnified view of the microcavity surface. **c**, surface of the microsphere one month after fabrication.

Comment 6: *Notably absent are basic morphological characterizations of the sensing structure—such as surface topography, geometric uniformity, or dimensional precision—despite their critical role in determining optical coupling efficiency and thermal response. In microcavity-based sensors, structural features directly influence mode confinement, heat distribution, and gas interaction dynamics. Additionally, given the focus on* CO_2 *detection, inclusion of* CO_2 *adsorption-desorption isotherms would further support the interpretation of sensitivity and response mechanisms.*

Answer 6: As described in Supplementary Note 2 of the supplementary materials, our microsphere microcavities are fabricated by melting the fiber end using arc discharge heating, resulting in a spherical structure due to liquid surface tension. Therefore, this fabrication process ensures that the microcavity has an extremely smooth surface, as shown in Fig. R17. silica itself does not possess specific chemical bonding sites for CO_2 molecules; thus, the microsphere surface has negligible adsorption on CO_2 molecules.

Because a portion of the modes within the microcavity extends into the surrounding air in the form of an evanescent field (Fig. S2 in Supplementary Note 1.2), CO_2 gas molecules near the microcavity surface can interact with this evanescent field, rather than through adsorption.

We have added Figs. R17a and R17b as Fig. 1b to the revised manuscript and included the relevant discussion: "Scanning electron microscope (SEM) images of the microsphere are shown in Fig. 1b. The extremely smooth surface of the microcavity ensures a very high Q-factor." and "The evanescent field component of the optical mode, as well as the light fields that are scattered from the tapered fiber in the coupling region, will be absorbed by the surrounding gas." on Page 7.

Thank you for your reminder. Regarding the CO_2 adsorption-desorption isotherm, while it is indeed important for materials with microporous structures, for fused silica that is fully dense and without special surface modification, the CO_2 adsorption-desorption isotherm is essentially a flat line coinciding with the horizontal axis, indicating negligible adsorption capacity.

The negligible adsorption of CO_2 on fused silica arises from its intrinsic structural and surface chemical properties. Fused silica is an amorphous SiO_2 network composed of densely connected SiO_4 tetrahedra and lacks intrinsic micro- or mesoporosity. Consequently, the accessible surface area originates almost entirely from the external surface, yielding an extremely low specific surface area (typically $<1\ m^2\ g^{-1}$), several orders of magnitude lower than that of porous adsorbents. In addition, the surface is primarily terminated by siloxane (-Si-O-Si-) and silanol (-Si-OH) groups, which interact only weakly with CO_2 through van der Waals forces, while specific chemisorption is generally absent. According to the classification of the International Union of Pure and Applied Chemistry, non-porous solids such as fused silica are expected to exhibit a Type II adsorption isotherm. However, due to the extremely small surface area and weak adsorbate–surface interactions, the adsorption signal is expected to be very small and may approach the detection limit. Adsorption capacity vs. pressure: Across the entire pressure range from low to high, the adsorption capacity ≈ 0. The curve shows almost no increase, appearing as a nearly horizontal line.

Hysteresis loop: None. The adsorption and desorption branches completely overlap, as the minimal adsorption that may occur is fully reversible monolayer physisorption.

Comment 7: *The mechanistic interpretation of CO_2 sensing lacks integrated support between textual analysis and experimental evidence. To enhance academic depth and readability, the authors should adopt the standard framework of "experimental observation → mechanistic explanation → graphical validation." Explicit correlation between measured responses and proposed physical processes—supported by schematic illustrations or comparative plots—is necessary to strengthen the logical coherence and scientific robustness of the study.*

Answer 7:

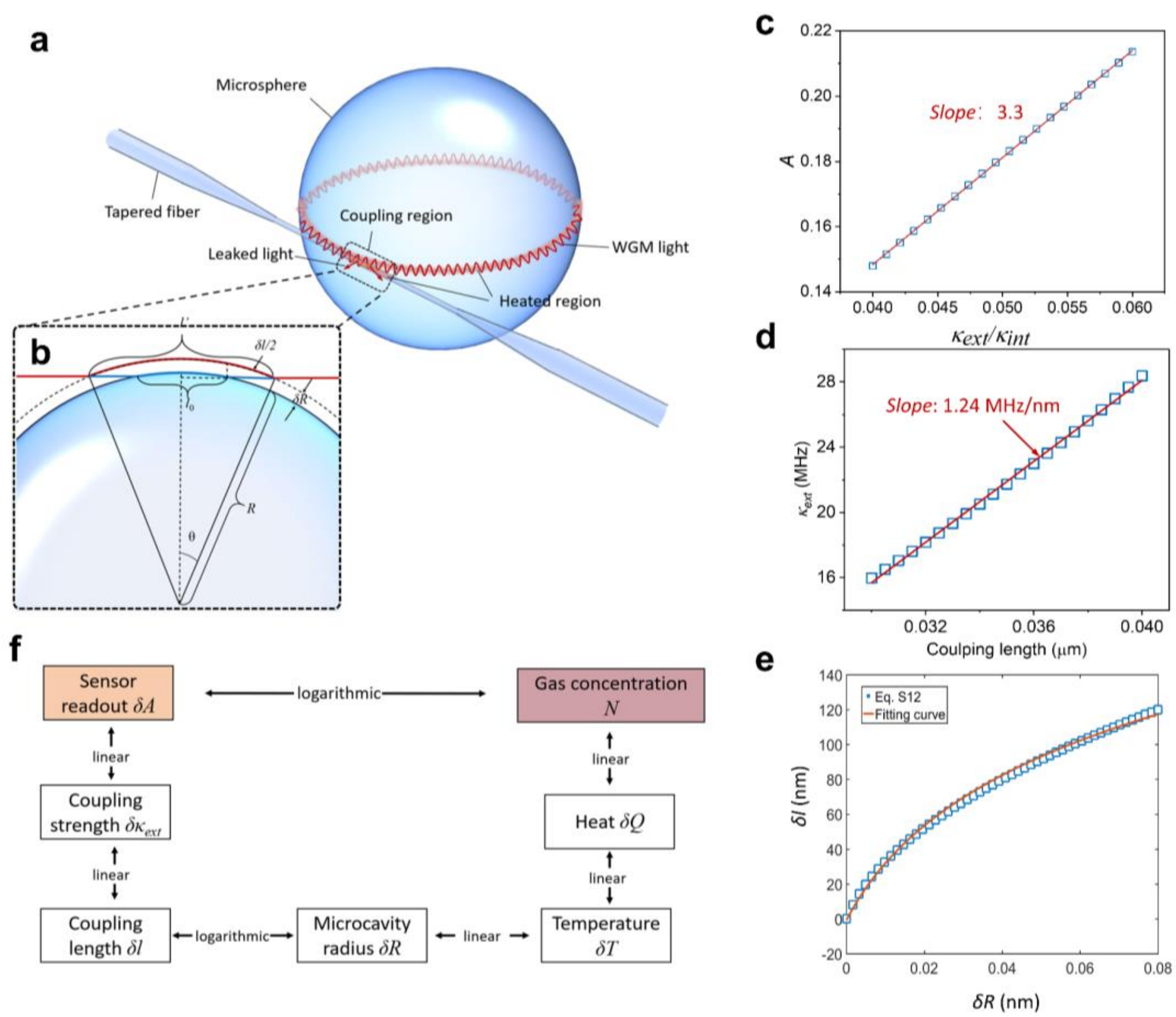

Fig. R18 | a, Schematic diagram of the coupling between a tapered fiber and a microsphere microcavity. The red wavy line and shaded area indicate the optical mode and heating region, respectively. **b**, Schematic diagram of the increased coupling length due to microsphere expansion.

c and **d**, show the linear relationship between coupling strength and sensor reading, and between coupling length and coupling strength, respectively. **e**, Logarithmic relationship between microsphere radius and coupling length. **f**, Block diagram of the relationship between physical variables.

We sincerely thank the reviewer for their constructive suggestions, which have helped improve the clarity and structural coherence of the manuscript. In the revised version, we reorganized the discussion to follow the standard framework of experimental observation → mechanistic modeling → graphical validation. We then verified the logarithmic relationship between the sensor signal and gas concentration across multiple experimental datasets. To validate the system's performance, we validated the sensor response by comparing the measured values with known gas concentrations, evaluated detection sensitivity via Allan deviation analysis, and finally demonstrated the system's capability for real-world atmospheric monitoring through long-term measurements.

To establish the link between the sensing mechanism and the experimental evidence, we have incorporated new schematics, theoretical models, and numerical simulations. The specific updates are as follows:

1. Schematic Illustration: We present the schematic of the dissipative microsphere CO_2 gas sensor (Fig. R18a), clearly depicting the coupling structure between the tapered fiber and the WGM microsphere, the optical mode distribution, and the gas absorption-induced heating region. Additionally, we provide a schematic illustrating the increase in the tapered fiber coupling area due to microsphere expansion caused by heating (Fig. R18b). This is particularly critical for analyzing the relationship between the variation in coupling length (δl) and the change in microsphere radius (δR).

2. Definition of Sensor Readout: To align with experimental observations, we redefined the sensor readout as $A = 1 - \Gamma_0$, where Γ_0 represents the transmittance at the WGM resonance dip. Based on coupled-mode theory, we established the relationship between the dissipative WGM sensor readout A and the external coupling strength κ_{ext}. We found that the sensor exhibits higher sensitivity in the under-coupled regime, and the readout A increases with κ_{ext} (Fig. R18c). This result is consistent with our experimental observations (Figs. 3-5 in the manuscript).

3. Analysis of Coupling Strength Factors: We analyzed the factors influencing the coupling strength κ_{ext}. Gas absorption-induced heating can affect κ_{ext} via the thermo-optic effect (refractive index change), thermal expansion of the tapered fiber, and thermal expansion of the microsphere.

However, numerical calculations reveal that the contributions of the first two factors are negligible compared to the third. Consequently, based on Fig. R18b, we established a mathematical relationship between the expansion of the microsphere radius and the change in coupling length under absorption-induced heating. This relationship can be well described by a logarithmic function, as shown in Fig. R18e.

4. Heat Generation and Transfer Modeling: First, based on the Beer-Lambert law, we derived the relationship between heat generation and gas concentration at low concentrations. Then, using a heat transfer model and the Finite Element Method (FEM), we calculated the temperature distribution of the microsphere, tapered fiber, and surrounding environment at thermal equilibrium. We found that, compared to an adiabatic model, the temperature rise of the sensor is significantly reduced due to heat conduction effects, remaining on the order of millikelvins (mK).

5. Quantitative Evaluation of the Theoretical Model: As shown in Fig. R18f, except for the logarithmic relationship between coupling length δl and microsphere radius δR, all other intermediate variables exhibit linear relationships. Therefore, we derived a final logarithmic relationship between the sensor readout A and gas concentration N, which aligns with experimental observations (Figs. 4b and 4e in the manuscript). Finally, our theoretical model estimates that the sensor readout changes caused by ppm-level gas in experiments require an absorption-induced temperature rise on the order of mK. This result is consistent with the temperature rise predicted by the heat-transfer model, supporting the validity of the proposed theoretical framework.

Detailed discussions regarding these points have been added to Supplementary Notes 1.1–1.5. We believe these additions significantly enhance the scientific depth and rigor of the manuscript.

The specific changes made to the article are as follows:

Figs. R18a and R18b have been added to the manuscript and supplementary materials as Fig. 1a and Fig. S4, respectively.

The relevant discussion "We can relate $\delta\kappa_{ext}$ to temperature change δT through thermal expansion and the thermo-optic coefficient:

$$\frac{\delta\Gamma_0}{\Gamma_0} = \frac{4\kappa_{int}\kappa_{\mathrm{ext}}}{|\kappa_{ext}^2 - \kappa_{int}^2|}\left[\frac{\partial\kappa_{\mathrm{ext}}}{\partial n} n\alpha_n \delta T + \frac{\partial\kappa_{\mathrm{ext}}}{\partial l}(\delta l_1 + \delta l_2)\right] \quad (3)$$

The first term in the parenthesis on the left side corresponds to the thermo-optic effect, where n and $\alpha_n = \frac{1}{n}\frac{\partial n}{\partial T}$ denote the refractive index and the thermo-optic coefficient of silica, respectively. δl_1 and δl_2 represent the variations in coupling length induced by the thermal expansion of the tapered fiber and the microsphere cavity, respectively (a detailed analysis is provided in Supplementary Note 1.3). The optical field distribution of the WGM microcavity and tapered fiber

at coupling cross section is shown in Fig. 1d. The coupling strength depends on the mode field overlap ratio between tapered fiber and microcavity as well as the length of coupling region, and the latter is more sensitive to temperature changes (detailed analysis is provided in Supplementary Note 1.2)." has been added to the "Theory and simulations" section of the manuscript.

The relevant discussion has been added to section "**Supplementary Note 1.1**":

For our dissipative WGM sensor, the change in light intensity transmittance at the resonance can be used as the sensor readout $A=1-\Gamma_0$, as shown in Fig. 3a of the manuscript. Since the change in κ_{int} caused by light absorption by low-concentration gases is negligible, its main contribution comes from the change in κ_{ext}.

According to Eqs. S5 and S6, assuming κ_{int} is a constant, we calculated the transmission spectrum of the WGM microcavity at different coupling strengths κ_{ext}, and the relationship between the sensor readout A and κ_{ext}, as shown in Fig. S2. In the under-coupled ($\kappa_{ext} < \kappa_{int}$) and over-coupled ($\kappa_{ext} > \kappa_{int}$) states, the sensor reading A increases and decreases, respectively, with increasing κ_{ext}. Furthermore, the rate of change of A with respect to $\kappa_{ext}/\kappa_{int}$ is much higher in the under-coupled state than in the over-coupled state, at 3.3 and -0.136, respectively. Therefore, in the experiment, setting the dissipative WGM microcavity sensor in the under-coupled state can significantly improve sensitivity.

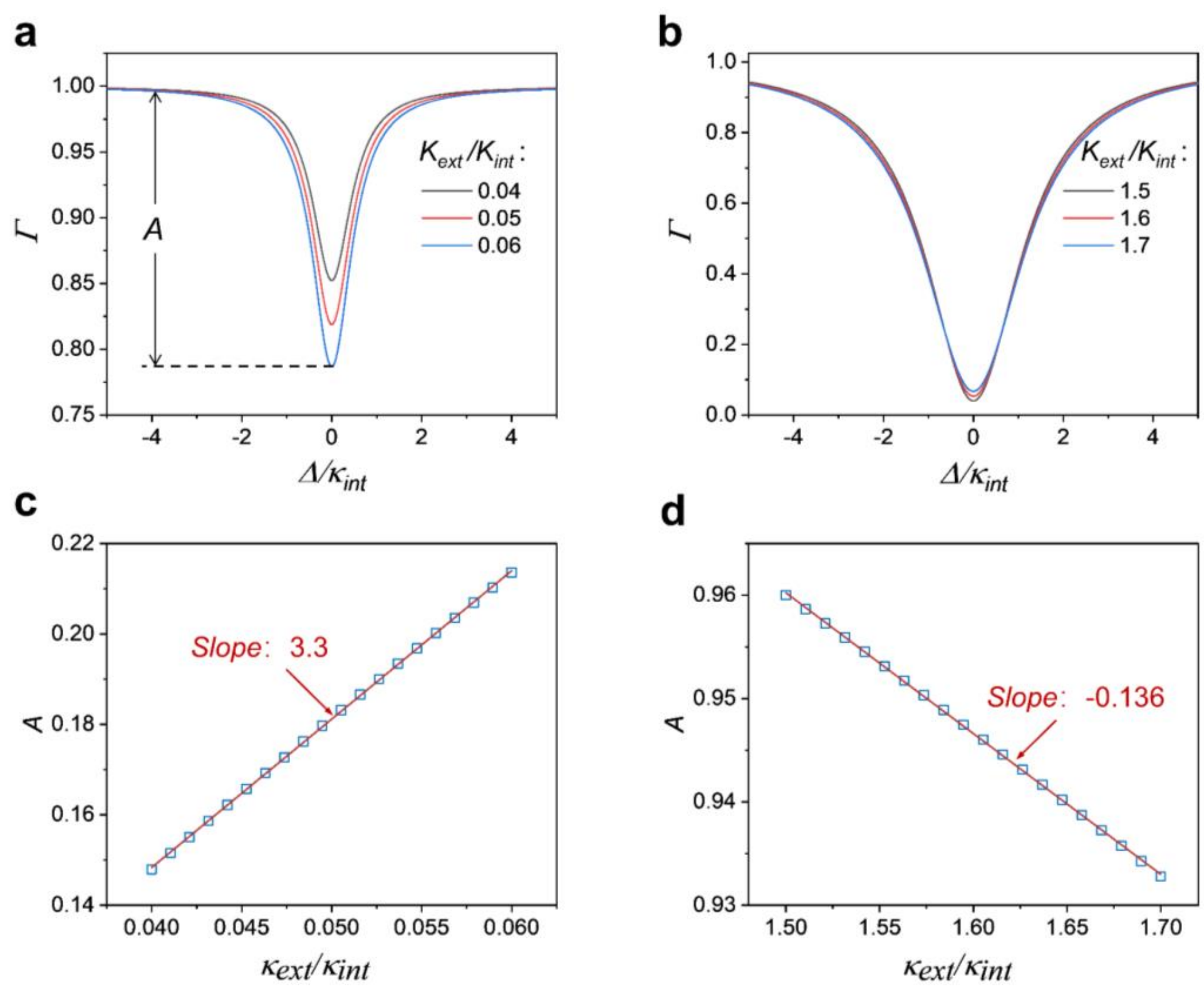

Fig. S1 | The WGM transmission lineshapes of the microsphere cavity under different κ_{ext} in under-

coupling **a** and over-coupling **b** states. **c** and **d**, represent the linear relationship between sensor readout A and κ_{ext} under these two coupling states, respectively.

The relevant discussion has been added to section "**Supplementary Note 1.2**":

The mode field distributions of the WGM microcavity and the tapered fiber at the cross-section of the coupling region were calculated using the finite element numerical method. To more clearly illustrate the overlap ratio of the mode field in Eq. S7, we calculated the modes of the microcavity and the tapered fiber separately and plotted them in the same coordinate system. Figs. S2a and S2b show the superimposed mode fields: that is, the distribution of $|\boldsymbol{E}_f|^2/N_f+|\boldsymbol{E}_r|^2/N_r$ in the coupling region. The diameters of the microcavity and the tapered fiber are 60 μm and 0.6 μm, respectively. Fig. S2c shows the distribution of the mode field intensities of the microcavity and the tapered fiber along the white curve in Fig. S2b. When the diameter of the tapered fiber is <1 μm, a large amount of evanescent field will leak from the fiber, thus enhancing its coupling strength with the microcavity.

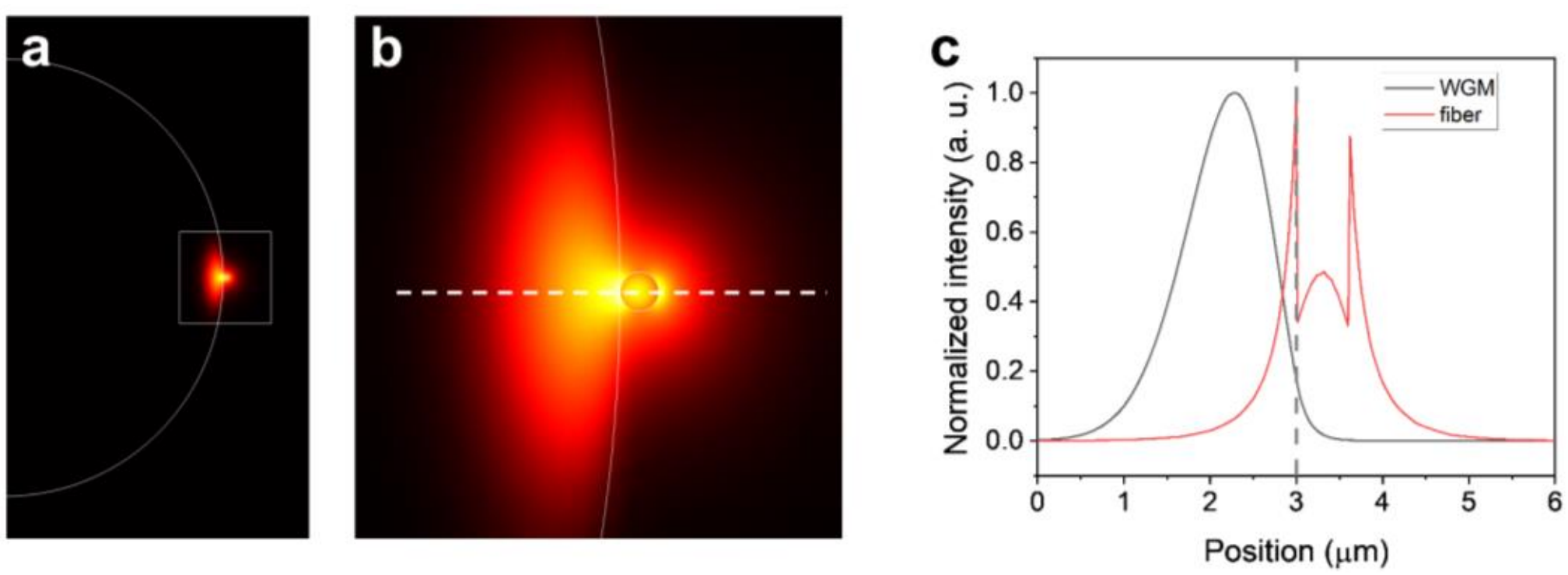

Fig. S2 | **a**, Mode field distribution ($|\boldsymbol{E}_f|^2/N_f+|\boldsymbol{E}_r|^2/N_r$) of microsphere cavity and tapered fiber in the coupling region. **b**, Zoom within the white square in **a**. **c**, The black and red curves represent the mode field distributions of the WGM mode ($|\boldsymbol{E}_r|^2/N_r$) and the tapered fiber mode ($|\boldsymbol{E}_f|^2/N_f$) along the white curve in **b**, respectively. The black vertical curve marks the boundary of the microsphere.

According to Eqs. S7-S9, the relationship between the coupling strength κ_{ext} and the coupling length, fiber diameter, and refractive index was calculated, as shown in Fig. S3. Fig. S3a shows a 2D contour plot of the coupling strength as a function of coupling length and fiber diameter. Initially, the coupling strength increases rapidly with increasing coupling length, but with decreasing fiber diameter, it shows a trend of increasing first and then decreasing. This is because the initial decrease in fiber diameter increases the proportion of the evanescent field, thus increasing the overlap between the mode field of the fiber and the microcavity mode. However,

further reducing the diameter causes the fiber mode to deviate from paraxial propagation, i.e., the axial electric field component increases, which significantly reduces its overlap with the microcavity mode, specifically the numerator of the integrand in Eq. S7. Fig. S3b shows a 2D contour plot of the coupling strength as a function of coupling length and refractive index. At a fixed coupling length, the change in coupling strength is only on the order of kHz within a refractive index change of δn = 0.0003. Fig. S3d shows the sensitivity of the coupling strength to the refractive index: -2.1 MHz/RIU. Since the refractive index n changes only by an order of 10^{-5} for a 1 K temperature variation, the influence of the thermo-optic effect can be ignored in our sensor.

Because the coupling strength κ_{ext} varies only by a few MHz in the experiment, it exhibits a linear relationship with the coupling length within this range, as shown in Fig. S3c. At a tapered fiber diameter of 0.6 μm, the sensitivity of the coupling strength to the coupling length is 1.24 MHz/nm.

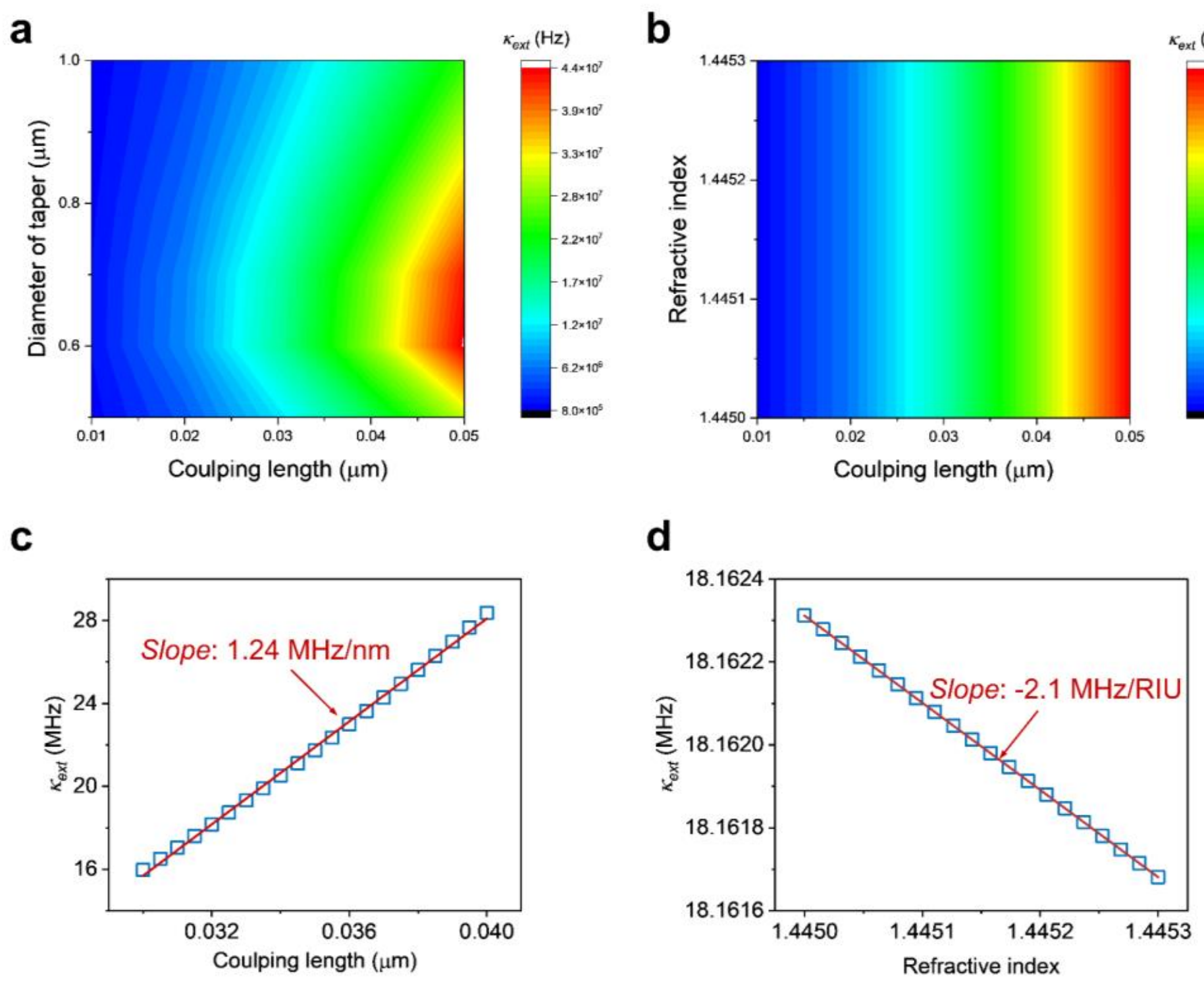

Fig. S3 | **a**, Two-dimensional contour plot of coupling strength varying with coupling length and tapered fiber diameter. **b**, Two-dimensional contour plot of coupling strength varying with coupling length and refractive index. **c**, The linear relationship between coupling strength and coupling length when the diameter of the tapered fiber is 0.6μm. **d**, The linear relationship between coupling strength and refractive index when the tapered fiber diameter is 0.6μm and the coupling

length is 30 nm.

The relevant discussion has been added to section "**Supplementary Note 1.3**":

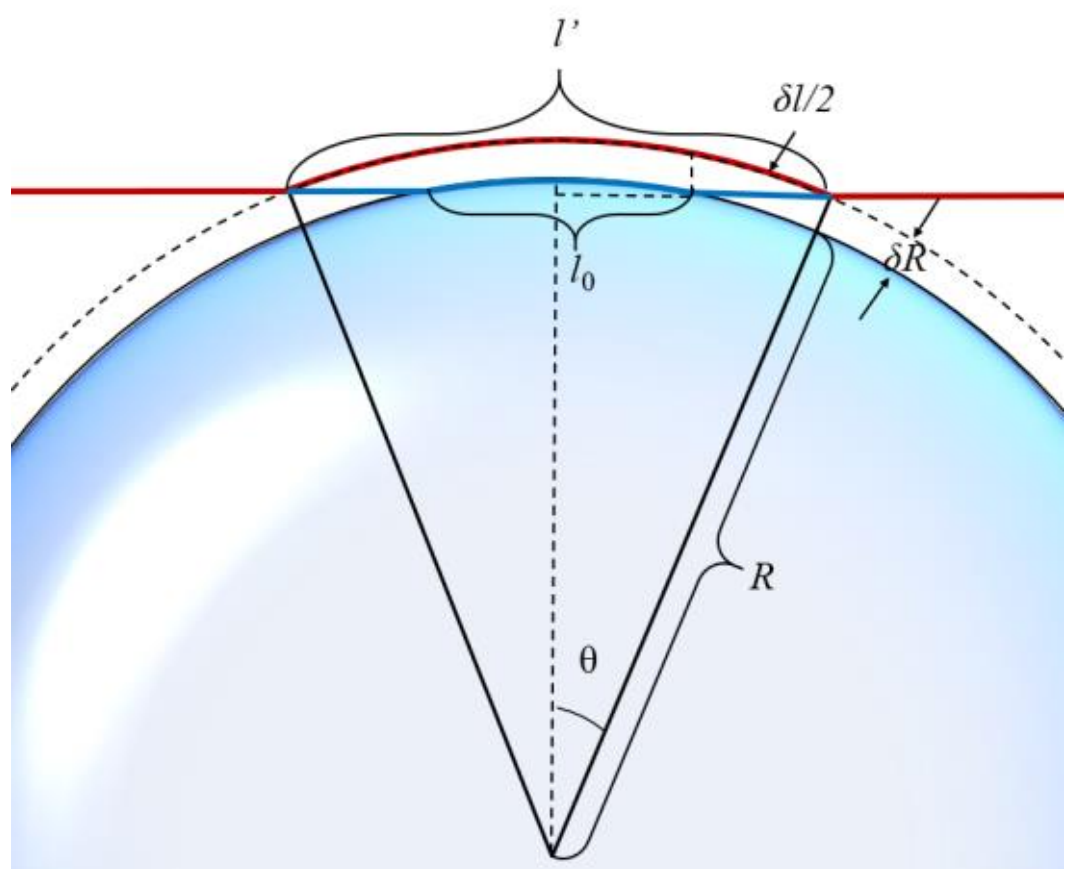

Fig. S4 | Changes in coupling length caused by microsphere expansion before (blue curve) and after (red curve) temperature increase. The black dashed line represents the edge of the microsphere after expansion.

In this section, we will analyze the effect of temperature changes on κ_{ext}. The change of κ_{ext} is mainly caused by the heat generated by the gas absorbing light, which will be written as:

$$\delta\kappa_{\mathrm{ext}} = \frac{\partial\kappa_{\mathrm{ext}}}{\partial n} n\alpha_n \delta T + \frac{\partial\kappa_{\mathrm{ext}}}{\partial l}\delta l \quad \text{(S10)}$$

The first term on the right-hand side of the above equation represents the contribution due to the thermo-optic effect, where $\alpha_n = (1/n) \cdot (\partial n / \partial T)$ is the thermo-optic coefficient of silica.

The change in coupling length δl due to temperature has two main contributions. Firstly, the heat generated by the absorption of light by the gas is conducted along the tapered fiber, causing the tapered fiber to heat up and expand within a certain range of the coupling region, resulting in a change of δl_1:

$$\delta l_1 = l_T \alpha_l \delta T \quad \text{(S11)}$$

where $\alpha_l = 1/l \cdot \partial l / \partial T$ is the thermal expansion coefficient of quartz, and l_T is the heated tapered fiber region.

On the other hand, light absorption by the gas causes thermal expansion of the microsphere microcavity, which in turn extends the length of the tapered optical fiber attached to the surface of the microcavity. As shown in Fig. S4, when the tapered fiber is coupled to the microsphere cavity,

a section of the fiber is in contact with the microsphere surface, and the coupling region appears as an arc. The blue and red arcs represent the coupling length before and after expansion, respectively, and the solid and dashed black lines represent the edge contours of the microsphere before and after expansion. Based on the geometric relationship in the figure, the change in coupling length δl_2 caused by the change in microcavity radius can be approximately expressed as:

$$\delta l_2 = l' - l_0 = 2(R+\delta R)\cdot \arccos\frac{R-\varDelta}{R+\delta R} - l_0 \tag{S12}$$

where R is the microcavity radius, δR is the expansion of the microcavity radius, and l_0 is the initial coupling length. In the above derivation, we assumed that the angle θ is very small, which is reasonable because the microsphere radius is on the order of tens of micrometers, while the coupling length l_0 is only on the order of tens of nanometers. The value of the correction term $\varDelta$ can be determined by ensuring that when $\delta R = 0$, δl_2 must be zero in the numerical calculation of Eq. S12.

According to Eqs. S10-S12, the change in coupling strength due to temperature variations can be derived:

$$\begin{aligned}\delta\kappa_{\text{ext}} &= \frac{\partial \kappa_{\text{ext}}}{\partial n} n\alpha_n \delta T + \frac{\partial \kappa_{\text{ext}}}{\partial l}(\delta l_1 + \delta l_2)\\ &= \frac{\partial \kappa_{\text{ext}}}{\partial n} n\alpha_n \delta T + \frac{\partial \kappa_{\text{ext}}}{\partial l}\left[l_T\alpha_l\delta T + 2(R+\delta R)\cdot\arccos\left(\frac{R-\varDelta}{R+\delta R}\right) - l_0\right]\end{aligned} \tag{S13}$$

where $\delta R = R\alpha_l\delta T$.

According to Eqs. S6, S10, and S13, the relationship between the sensor readout A and the temperature change δT can be derived. The first, second, and third terms on the right side of Eq. S13 represent the changes in coupling strength due to the thermo-optic effect, the expansion of the tapered fiber, and the expansion of the microsphere, respectively. Based on the calculation results in Fig. S3d, the change in coupling strength caused by the thermo-optic effect in the experiment is negligible. Furthermore, according to the heat conduction model (see the analysis in Supplementary Note 1.4), the heated region of the tapered fiber is only tens of micrometers, and its contribution to the change in coupling strength is also negligible. Therefore, the change in coupling strength mainly depends on the expansion of the microsphere WGM cavity, i.e., $\delta l \approx \delta l_2$.

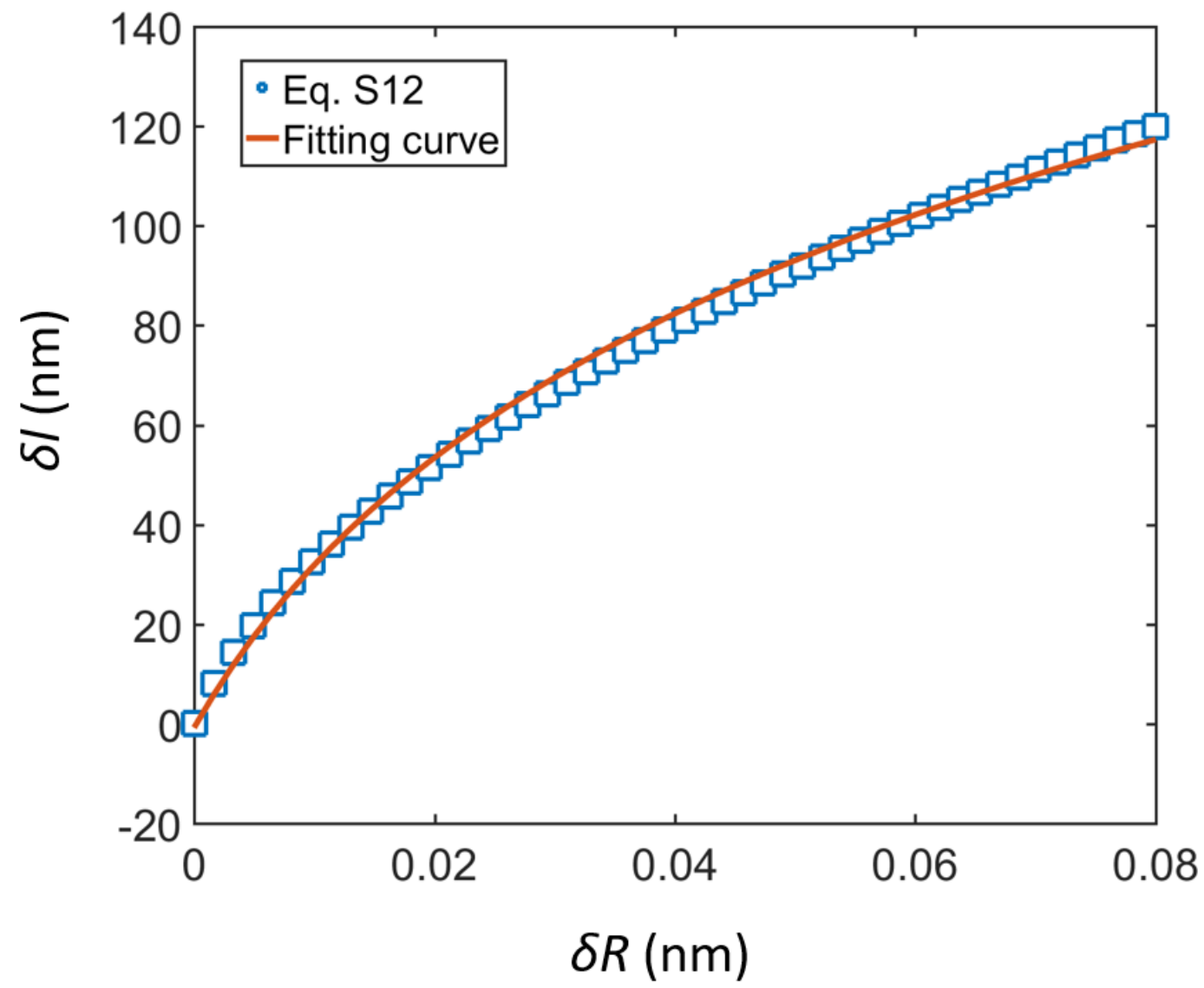

Fig. S5 | The logarithmic relationship between the change in coupling length δl and the change in microsphere radius δR.

As shown in Fig. S5, the blue squares represent the numerical results using Eq. S12. In the calculation, l_0, R, and Δ were set to 20 nm, 30 µm, and 1.65 pm, respectively. Clearly, there is a nonlinear relationship between δl_2 and δR. We used a logarithmic function for fitting and found that it fits very well. The fitted function is:

$$\delta l = 268 + 64\ln(\delta R + 0.015) \tag{S14}$$

According to Figs. S1c and S3c, we find that the sensor readout A is proportional to the change in coupling length δl. On the other hand, due to the expansion of the microcavity caused by gas absorption, at low concentrations, the gas concentration N is proportional to the temperature increase of the microcavity (see Supplementary Note 1.4), and consequently proportional to the change in microsphere radius δR. According to Fig. S5, δl has a logarithmic relationship with δR; therefore, it can be deduced that the sensor readout A has a logarithmic relationship with the gas concentration N, which is consistent with the experimental results shown in Fig. 4 of the manuscript.

The relevant discussio has been added to section "**Supplementary Note 1.4**":

1.4 Gas absorption induces temperature rise

According to the Beer-Lambert law, the intensity of light absorbed by a gas is:

$$\Delta I = I_0(1 - e^{-\alpha(\nu)L}) \quad (S15)$$

where I_0 is the initial light intensity, L is the interaction distance in the gas, and α is the absorption coefficient, which is related to the laser frequency v:

$$\alpha(\nu) = Ng(\nu - \nu_0)S \quad (S16)$$

where N represents the gas molecular number density, S is the absorption line strength, and $g(v\text{-}v_0)$ is the gas absorption line shape function. In the experiment, the linewidth of the laser used is much smaller than the absorption linewidth, and the laser frequency is set at the absorption peak; therefore, the line shape function only needs to consider g_0 at v_0. For a laser wavenumber of 6359.967 cm^{-1}, g_0 and S are 4 cm and 1.76×10^{-23} $cm^{-1}/mol \cdot cm^{-2}$, respectively. The relationship between the absorption coefficient and gas concentration is $\alpha = k \cdot N$, where $k = 7.04 \times 10^{-23}$ cm^2/mol. Therefore, for weak absorption, i.e., $\alpha L << 1$, Eq. S15 can be written as:

$$\Delta I = I_0 \alpha(\nu) L \quad (S17)$$

Therefore, the heating power is:

$$P_Q = I_0 \alpha L A_Q = I_0 \alpha V \quad (S18)$$

A_Q and V represent the heating area and volume, respectively. Under the adiabatic approximation (i.e., neglecting heat conduction), the temperature increase per unit time in the heated region is:

$$\Delta T = \frac{P_Q}{\rho c_v V} = \frac{I_0 \alpha}{\rho c_v} \quad (S19)$$

where ρ and c_v are the density and specific heat capacity of quartz, respectively. In our experiment, I_0 is the light intensity density inside the microsphere WGM cavity, which is significantly enhanced when the laser frequency approaches the microcavity resonance.

$$I_0 = B\Gamma \frac{P_{in}}{A_{eff}} \quad (S20)$$

P_{in} is the incident power in the tapered fiber, A_{eff} is the mode field area of the WGM in the microcavity, and B is intracavity intensity enhancement factor for the WGM microcavity. The inclusion of the Γ factor accounts for the evanescent field portion of the optical mode interacting with the gas, which leaks outside the microcavity. Furthermore, since the tapered fiber is in close contact with the microcavity surface, strong scattering occurs at the coupling point, significantly increasing the leakage of the light from the microcavity into the surrounding air. Combining Eqs. S19 and S20, the heating rate of the microcavity due to gas absorption at a concentration of 1 ppm

can be estimated to be approximately 0.04 K/s, with the parameters used shown in Table S1.

Table S1 The parameter values used for numerical estimation

Parameter	Symbol	Value
Incident power	P_{in}	10 mW
Mode field area	A_{eff}	3 μm^2
Absorption peak	g_0	4 cm
Absorption line strength	S	1.76 × 10^{-23} cm^{-1}/mol • cm^{-2}
Gas molecular number density	N (1 ppm)	1.83×10^{13} mol/cm^3
Thermal expansion coefficient	α_l	0.55×10^{-6} K^{-1}
Thermo-optic coefficient	α_n	10×10^{-6} K^{-1}
Silica density	ρ	2.2 g/cm^3
Temperature	T	300 K
Specific heat capacity	c_v	0.67 J/(g • K)
Constant pressure heat capacity	C_p	0.703 J/(g • K)
Thermal conductivity	k	1.38 W/(m*K)
Light intensity enhancement factor	$B \cdot \Gamma$	~100

Considering that the generated heat diffuses into the microsphere microcavity, the tapered fiber, and the surrounding air, the heat conduction equation is needed:

$$\rho C_p \frac{\partial T}{\partial t} - \nabla \cdot (k \nabla) = Q \quad \text{(S21)}$$

where T represents temperature, and C_p represents the constant-pressure heat capacity, which for silica is approximately equal to the specific heat capacity c_v. k and Q represent thermal conductivity and generated heat, respectively. Since the composite structure of the tapered fiber and microsphere microcavity is an irregular geometry, a numerical method is required to solve Eq. S21. As described in Fig. 1b in the manuscript, we used a heating ring surrounding the equator of the microsphere to simulate gas absorption-induced heating. The microsphere and tapered fiber were enclosed within a large, air-filled cube. The boundaries of the cube are set to a constant ambient

temperature to simulate the large heat capacity of the surrounding environment. The steady-state temperature distribution is shown in Fig. S6. Fig. S6a shows the temperature distribution at a heat generation per unit volume of $Q = 0.6 \times 10^{6}$ W/m^3 (corresponding to 3 ppm gas absorption), and Fig. S6b shows the temperature variation along the white curve in Fig. S6a. It can be seen that due to heat conduction, the temperature rise is only 0.0012 K compared to the adiabatic condition described in Eq. S19, and the temperature distribution is relatively uniform within the microsphere cavity. The heat does not propagate specifically along the tapered fiber, but rather diffuses into the surrounding air over a range of tens of micrometers. This is because the thermal conductivity cross-sectional area of the tapered fiber is much smaller than that of the surrounding air, even though its thermal conductivity is much greater than that of air.

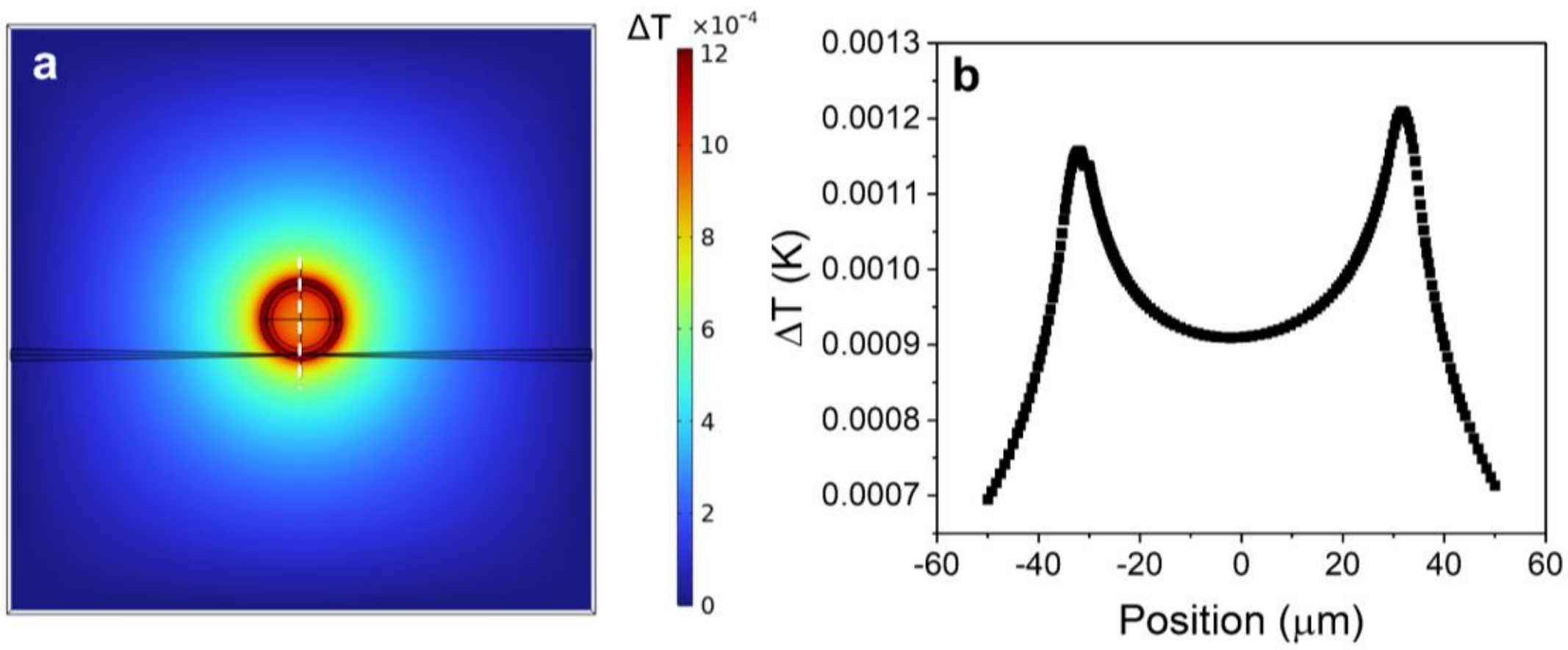

Fig. S6 | a, Temperature variation distribution on the equatorial plane of the microsphere. **b**, Temperature variation along the white dashed line in **a**.

The relevant discussion has been added to section "**Supplementary Note 1.5**":

1.5 Comparison between theoretical estimation and experimental results

In this section, we use the theoretical model described above to perform numerical estimations and validate the model against experimental results. According to Fig. 4a in the manuscript, the experimentally measured signal change is 0.005 (normalized) for a gas concentration change of 1.5 ppm (from 1.5 ppm to 3 ppm). Based on the sensitivity of the coupling strength to the sensor readout (~3.3) shown in Fig. S2c of Supplementary Note 1.1, this signal change requires a change in coupling strength κ_{ext} of approximately 0.45 MHz. Here, we assume the intrinsic loss of the microsphere is 300 MHz, corresponding to an intrinsic Q factor $Q_{int} \sim 4 \times 10^6$. Then, using the sensitivity of κ_{ext} to the coupling length l obtained from Fig. S4 in Supplementary Note 1.2: ~1.24 MHz/nm, we can deduce that the coupling length change δl is 0.36

nm. From Eq. S12 and the logarithmic fitting curve in Fig. S5 of Supplementary Note 1.3, we obtain the slope near the zero point, $\delta l/\delta R \sim 5 \times 10^3$. Therefore, a δl change of 0.36 nm corresponds to a δR change of 0.07 pm. Using the thermal expansion formula: $\delta R = R \cdot \alpha_l \cdot \delta T$, and substituting the microsphere radius of 30 μm and the thermal expansion coefficient $\alpha_l = 0.55 \times 10^{-6}$, we obtain a required temperature change of 0.004 K. This result agrees well with the calculated temperature increase due to gas absorption at 3 ppm shown in Fig. S6 (0.0012 K). Therefore, the validity of our theoretical model can be proven.

MANUSCRIPT REVISIONS DETAILS

Journal: Nature Communications

Ms. No.: NCOMMS-25-83903B

Ms. Title: Sub-ppb CO_2 Detection based on Dissipative Whispering Gallery Mode Microcavity Sensor

Authors: Shujing Ruan, Guangzhen Gao, Jianing Zhang, Haotian Wang, Dongxing Cheng, Jun Guo, Chuanyong Ren, Weidong Chen, Deyuan Shen, and Tingdong Cai

We sincerely thank the Editor and the three reviewers for their careful evaluation and constructive comments on our manuscript. We have revised the manuscript and the Supplementary Information accordingly.

In the revised manuscript and Supplementary Information, all modifications are highlighted in **green** for clarity. In this response document, reviewer comments are shown in ***italic***, our responses are provided in **blue**, and the corresponding revised or newly added text in the manuscript is indicated in **green**.

Reply to Reviewer #1

I appreciate the very thorough revision by the authors. They have carefully addressed my comments and reworked parts of the manuscript to improve the scientific clarity. The revision has significantly increased the quality of the paper.

We sincerely thank the reviewer for the recognition of our work and for providing detailed suggestions. We are happy to respond to all the comments below. We appreciate the reviewer for helping us further improve the quality of our manuscript.

However, one major inconsistency remains that should be clarified before I can recommend publication:

Comment 1: *Figures 5e and 5f appear inconsistent with the rest of the manuscript. These figures are presented as measurements at the detection limit, yet when comparing Figures 3a,b with Figures 5e,f, the amplitudes and noise levels of both the direct and the 2f signals do not match. If Figures 5e and 5f indeed correspond to measurements at the detection limit, the signal characteristics should nearly overlap within the noise level. As currently presented, the comparison does not appear physically consistent, and the authors should clarify / correct these figures.*

Answer 1: We sincerely thank the reviewer for the careful reading and for pointing out this important issue. As correctly noted, for conventional absorption spectroscopy, the detection limit is typically defined by the condition where the signal amplitude becomes comparable to the noise level. This criterion, however, does not directly apply to the sensing mechanism employed in our work.

It is important to clarify that the signals presented in Figs. 3 and 5 are not absorption signals, but rather resonance mode signals of the WGM microcavity. This represents a fundamental difference from traditional absorption-based detection schemes. In our approach, the optical resonance mode always exists regardless of the gas concentration. Gas absorption induces a thermal dissipation effect, which perturbs the coupling condition of the microcavity and leads to a change in the resonance depth (or mode amplitude).

Therefore, the sensing information is not determined by the presence or absence of a signal, but by the resolvable change in the resonance amplitude. The detection limit

is thus defined by the minimum detectable variation in the mode amplitude rather than the absolute signal level. As a result, even at concentrations approaching the detection limit, or even at zero concentration, the resonance mode itself still exhibits a high signal-to-noise ratio, as shown in Figs. 5e and 5f.

Regarding the apparent inconsistency in amplitude and noise levels between Figs. 3 and 5, this arises from differences in experimental settings. To clearly resolve the small signal variations at ultra-low concentrations, the detector gain and lock-in amplifier sensitivity were increased during the measurements shown in Fig. 5. Consequently, the absolute signal amplitude and noise level are not directly comparable between these figures. Importantly, this adjustment does not affect the underlying physical mechanism or the extracted sensing results, but only improves the visualization of weak signals in different concentration regimes.

To avoid potential misunderstanding, we have added the following clarification at the end of the second paragraph in the section "Measurement Precision and Detection Sensitivity":

"To clearly resolve the weak signal variations at low concentrations, the detector gain and lock-in amplifier sensitivity were increased for the measurements in Figs. 5e and 5f. Therefore, the absolute signal amplitude and noise level are not directly comparable with those in Fig. 3. This adjustment does not affect the sensing mechanism, but is used solely to optimize signal visualization across different concentration ranges."

Comment 2: *Another issue concerns the comparison with the reference instrument. This comparison significantly strengthens the manuscript, particularly by lending credibility to the field measurements. However, regarding the quantitative comparison, I would like the authors to elaborate on the statement: "As shown, the signals remain highly consistent across different temperatures, with deviations within 1%." Is this claim supported by all data points? In particular, toward the end of the measurement sequence the discrepancy between the measurements appears larger than 1%. The authors should either correct or moderate this statement and provide a more detailed data analysis e.g. with pointing out the best and worst data match through insets. In particular, it would be useful to quantify the discrepancy (in ppm), attempt to explain it, and compare it with the laboratory measurements ~400 ppm.*

Answer 2: We appreciate the reviewer's valuable suggestions. We agree that a quantitative comparison against a reference instrument not only enhances the credibility of the field measurement results but also helps to more objectively evaluate the accuracy and applicable range of our sensor. Accordingly, we have conducted further data analysis and supplemented the results in this section to more clearly elucidate the consistency between the two measurements and the sources of the observed deviations.

First, to address the apparently large deviations observed in certain periods of Fig. 6a, we have added insets showing representative regions with both small and large discrepancies. Error bars indicating the maximum deviations are also included, allowing a more intuitive evaluation of the agreement between the two instruments. These results are presented as Fig. R1 and have been incorporated as Fig. 6a in the revised manuscript.

Based on the statistical results, the deviations for the majority of the data points are concentrated within the range of 0-5 ppm, with large deviations occurring only during a few time periods. Quantitative analysis shows that the maximum deviation between the CO_2 concentration retrieved from the raw optical mode and that measured by the commercial instrument is approximately 26 ppm, whereas the maximum deviation for the concentration retrieved from the $2f$ signal is approximately 23 ppm.

These supplementary results have been described at the beginning of the second paragraph of the "Real-Time Monitoring of Ambient CO_2" section in the revised manuscript. The revised description reads as follows:

"Most discrepancies between the proposed sensor and the commercial analyzer are within 0-5 ppm for the majority of the measurement period. Larger deviations occur only during limited time intervals, with the maximum deviation reaching approximately 26 ppm for concentrations retrieved from the raw optical mode and approximately 23 ppm for those obtained from the $2f$ signal."

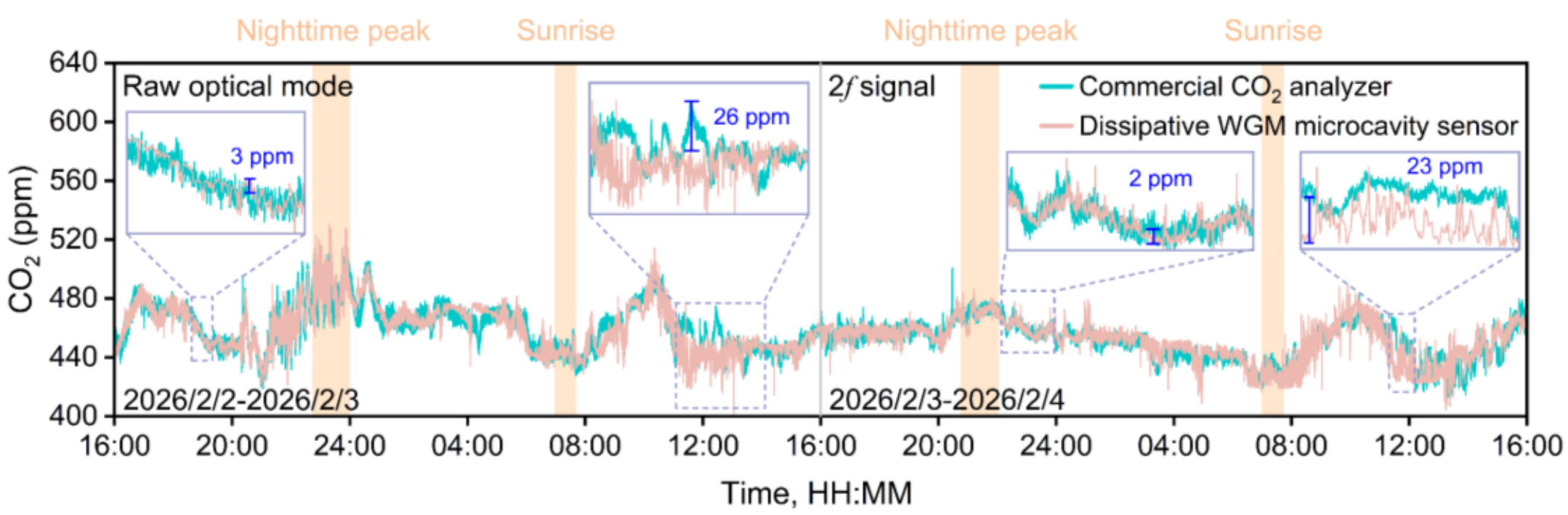

Fig. R1 | Comparative time series of ambient CO_2 concentration over ~24 h, measured simultaneously by the dissipative WGM-based sensor (raw optical mode and $2f$ signal)

and a calibrated commercial reference analyzer.

In addition, following the reviewer's suggestion, we further carried out a laboratory validation experiment using a standard gas sample. A 400 ppm CO_2-N_2 standard gas mixture was prepared using a gas mixing system, and simultaneous measurements were performed with our sensor and a commercial analyzer. The results are presented in Fig. R2 (now incorporated as Fig. 6b in the revised manuscript).

The results show that the deviations between the concentrations retrieved from the raw optical mode and the 2*f* signal and the standard value are approximately 6.4 ppm and 4.3 ppm, respectively, while the deviation of the commercial instrument is approximately 10.5 ppm. These results demonstrate that, under controlled conditions, our sensor achieves comparable or even improved accuracy relative to the commercial instrument.

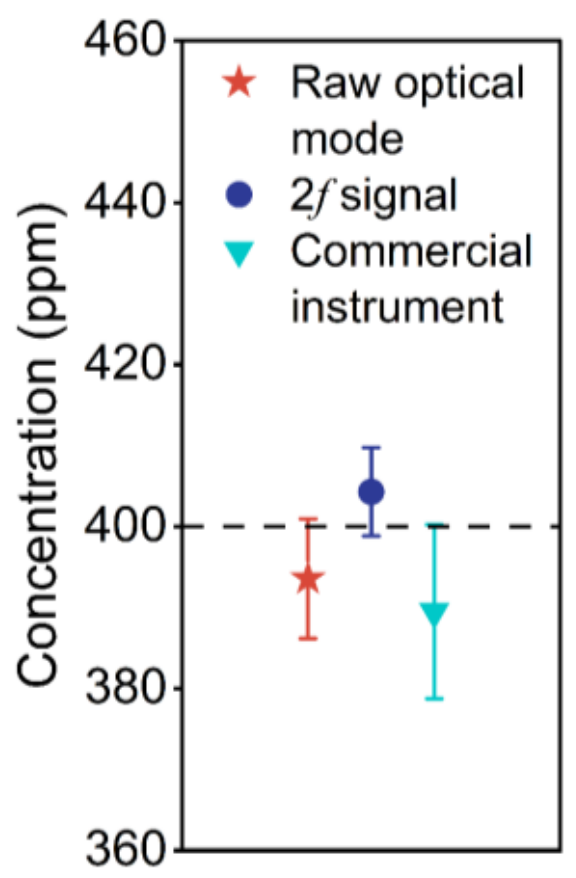

Fig. R2 | Comparison of three measurement methods at a known CO_2 concentration of 400 ppm.

These supplementary results have been described at the beginning of the second paragraph of the "Real-Time Monitoring of Ambient CO_2" section in the revised manuscript. The revised description reads as follows:

"A comparative measurement was carried out in the laboratory for a 400 ppm CO_2-N_2 standard gas sample prepared using a gas mixing system, with simultaneous detection by our sensor and a commercial instrument. The results are presented in Fig. 6b. The deviations between the concentrations determined by the raw optical mode and the 2*f* signal of our device and the standard value are approximately 6.4 ppm and 4.3 ppm, respectively, whereas the deviation of the concentration measured by the commercial instrument from the standard value is approximately 10.5 ppm."

Regarding the statement "As shown, the signals remain highly consistent across different temperatures, with deviations within 1%," we thank the reviewer for pointing out the potential ambiguity. This statement refers to Fig. S11 in the Supplementary Information rather than the comparison in Fig. 6. Fig. S11 shows the variation of the raw microcavity mode signal for a fixed 400 ppm CO_2-N_2 sample under temperature changes from 27 to 42 °C, and is intended to demonstrate the robustness of the packaged sensor against ambient temperature fluctuations. To eliminate potential misunderstanding, we have relocated the corresponding paragraph to follow Fig. 6 in the revised manuscript.

A few additional minor issues remain, and I would appreciate it if the authors could address them before publication.

Comment 3: *Page 13, line 257: The authors refer to the signal–concentration relation as exponential, whereas it should rather be described as logarithmic. Please correct this. However, I still find it somewhat difficult to describe the dependence as strictly logarithmic. In the theoretical analysis, the relationship is not strictly logarithmic, and the fit shown in Figure R3 is not particularly strong. This is visible not only in Figure R3 itself but also in the residuals, which exhibit a distinct structure rather than purely random noise. I wonder whether a higher-order polynomial fit might lead to smaller residuals than the logarithmic fit. In any case, I would recommend emphasizing that the logarithmic dependence is not directly derived from theory. For the same reason, the statement "The excellent fitting performance confirms the system's robust quantitative capability across a wide concentration range" should be moderated.*

Answer 3: We thank the reviewer for this careful and constructive comment. We agree that our previous wording was imprecise. In the manuscript, the fitting equation was written in the inverse form, $X_{CO2}=C_0\exp(kP)$, because this form was used to retrieve the gas concentration from the measured signal amplitude. However, when the sensor signal is described as a function of gas concentration, the same relation corresponds to an approximately logarithmic dependence. We have therefore corrected the text and removed the statement that the signal follows an exponential dependence on CO_2 concentration.

The sentence "According to the measured data, the signals follow an approximately logarithmic relationship with CO_2 concentration. To conveniently

retrieve gas concentrations from experimentally measured signals, we applied an inverse exponential fit to them" has been added to the second paragraph of the "CO_2 concentration measurement" section in the revised manuscript.

We also agree that the response should not be described as strictly logarithmic. The theoretical analysis does not directly yield a pure logarithmic law. Instead, it predicts a monotonic nonlinear response arising from the combined effects of absorption-induced heating, thermally induced microsphere expansion, changes in effective coupling length, and coupling-strength variation. The logarithmic function was used only as a compact semi-empirical approximation over the investigated concentration range.

Following the reviewer's suggestion, we additionally compared the logarithmic-type fits with 5th-order polynomial fits. The correlation coefficient (R^2) of the fitting results improved from 0.9947 to 0.9989. As expected, the polynomial fitting can reduce the fitting residuals because of their additional fitting parameters, which is shown in Fig. R1. It also appears as Fig. S5 in the revised supplementary note. We agree with the reviewer that a higher-order polynomial can provide smaller fitting residuals. Therefore, in the revised manuscript we no longer emphasize the logarithmic fitting for a strict physical dependence. Instead, we describe the logarithmic-type curve only as a compact phenomenological representation of the nonlinear trend, while polynomial fitting is also provided as an alternative fitting function. However, based on the theoretical analysis presented in this paper, combined with semi-empirical logarithmic fitting, the use of a logarithmic function in practical applications enables a faster retrieval of gas concentrations by utilizing fewer fitting parameters. We have replaced Fig. S5 in the supplementary note with Fig. R1 in this letter and the relevant discussions "In Fig. S5, we performed both a 5th-order polynomial fit and a logarithmic fit to Eq. S12, yielding correlation coefficients of $R^2 = 0.9989$ and $R^2 = 0.9947$, respectively. Although Eq. S12 does not analytically impose a strictly logarithmic law and does not exhibit lower fitting residuals compared to the 5th-order polynomial, the logarithmic function provides a more intuitive and useful physical picture." and "Compared to higher-order polynomials, logarithmic functions possess fewer fitting parameters, enabling the rapid retrieval of gas concentrations." has also been added to Supplementary Note 1.3.

To avoid overstatement, we have also moderated the conclusion. The previous sentence "The excellent fitting performance confirms the system's robust quantitative capability across a wide concentration range" has been replaced by: "The monotonic and reproducible fitting processing supports quantitative concentration retrieval over

the investigated concentration range; nevertheless, the fitting should be regarded as a semi-empirical approximation rather than a strict theoretical law (detailed discussion can be found in Supplementary Note 1.3).” on page 13 in the revised manuscript.

The previous sentence “Given that the relationship between δl and δR can be well-fitted by a logarithmic function, a logarithmic dependence of A on N is ultimately obtained, which is consistent with experimental observations (Fig. 4b).” has been replaced by: “Although Eq. (4) does not analytically impose a strictly logarithmic law, the calculated δl–δR dependence over the small-expansion range relevant to our experiments can be reasonably approximated by a logarithmic-type function. Therefore, an approximately logarithmic dependence of A on N is ultimately obtained, which is consistent with experimental observations (Fig. 4b).” on page 26 in the revised manuscript.

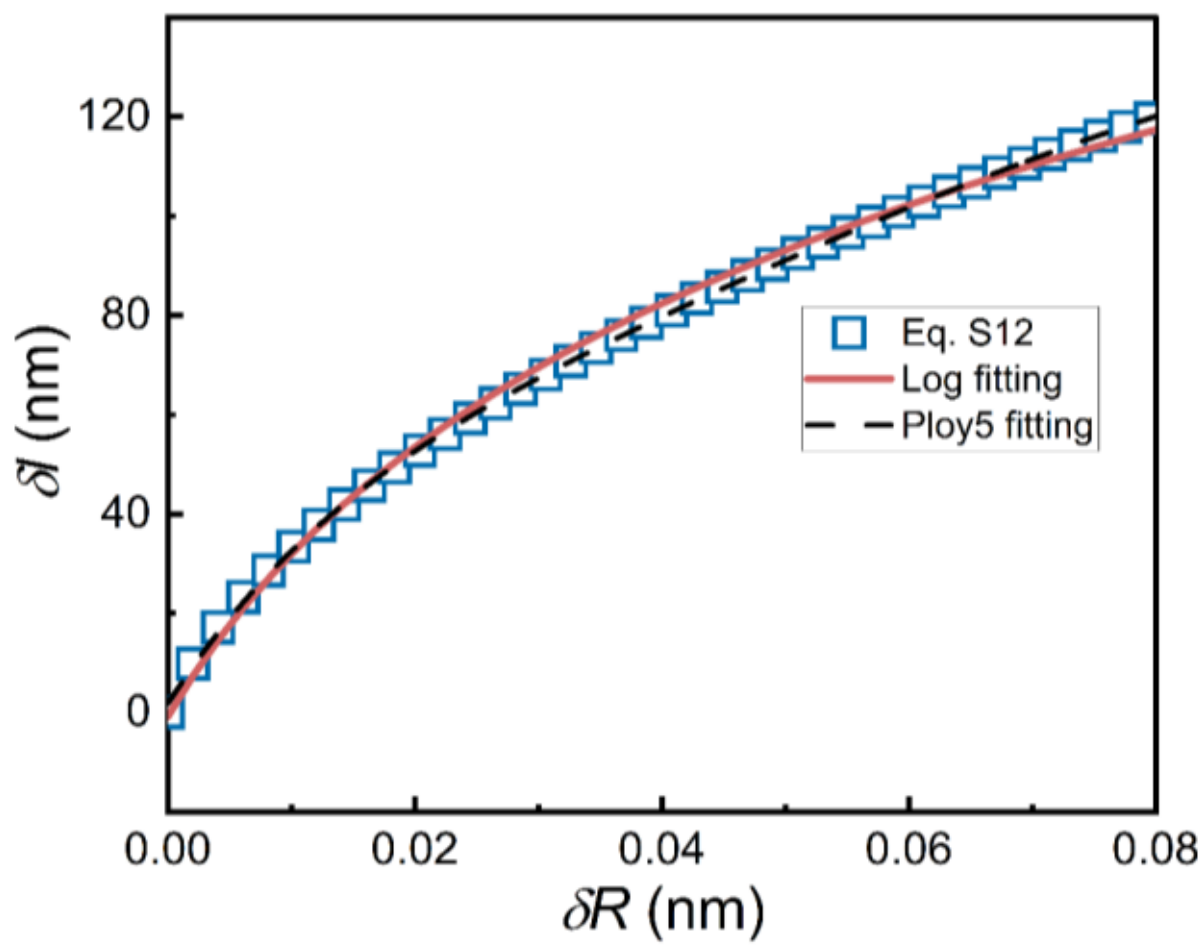

Fig. S5 | The relationship between the change in coupling length δl and the change in microsphere radius δR. Blue squares: Calculated from Eq. 12; Red curve: Logarithmic fit; Black dashed line: 5th-order polynomial fit.

On the other hand, by Taylor expansion of Eq. S12 in Supplementary Note 1.3 at the zero point, we can obtain that when δR approaches 0, δl is approximately:

$$\delta l \approx \delta l_2 = 2(R+\delta R)\cdot \arccos\frac{R-\Delta}{R+\delta R} - l_0 \approx 2\sqrt{2R(\delta R+\Delta)} - l_0 \tag{R1}$$

The derivation of the above equation uses: $\epsilon \to 0,\ \arccos(1-\epsilon) \approx \sqrt{2\epsilon}$ 。 Based on previous theoretical analysis, the sensor readout A and the gas concentration N are proportional to δl and δR, respectively. Therefore,

$$\frac{\partial A}{\partial N} \propto \frac{\partial(\delta l)}{\partial(\delta R)} \approx \sqrt{\frac{2R}{\delta R+\Delta}} \tag{R2}$$

According to Eq. R2, the sensitivity of our sensor does not increase infinitely as δR approaches zero; rather, it possesses a limit:

$$\sqrt{\frac{2R}{\Delta}}$$

When the gas concentration is sufficiently low, A exhibit a linear response to N, implying that the sensor possesses a specific limit of detection (LOD). This is attributed to the presence of the correction term Δ. As illustrated in Fig. S4, Δ is influenced by the initial coupling length, the surface roughness of the microcavity and tapered fiber, and mechanical fluctuations. Because the sensor exhibits maximum sensitivity at the LOD, the mode amplitude variations for the near-limit concentrations of 0.5 ppb and 0.2 ppb (Figs. 5e and 5f in the manuscript) remain distinguishable. Conversely, at concentrations below the LOD, the signal amplitude differences between varying concentrations fall below the noise level, thereby rendering concentration discrimination impossible.

To be clearer, we have added the discussion to Supplementary Note 1.3:

"Based on Eq. S12, δl exhibits an approximately linear dependence on δR at sufficiently low gas concentrations. Consequently, the sensitivity does not increase indefinitely as the concentration decreases, establishing a definitive limit of detection (LOD). Therefore, the sensitivity of the sensor increases as the concentration decreases, reaching a maximum at the LOD."

Comment 4: *I am also concerned about the sensor performance at higher concentration levels, as the sensitivity will decrease rapidly with increasing concentration. It should therefore be clearly stated (in the conclusion?) that the sensor performs great only for low concentration levels, and the relevant operating concentration range should be defined. I believe that even with this limitation, the device would still be suitable for many practical applications. However, could you list some of them, i.e. where sub ppl CO2 measurement is beneficial?*

Answer 4: We sincerely thank the reviewer for raising this very important point. We fully agree that, because the sensing mechanism in this work relies on changes in microcavity coupling induced by absorption-induced thermal dissipation, the sensitivity is highest in the low-concentration region, while the response curve gradually flattens with increasing concentration. As a result, the system is more suitable for high-sensitivity detection in the low-concentration range. We also agree that it is

necessary to explicitly state its optimal operating range and applicable scenarios in the manuscript. Based on the experimental results (particularly the concentration response curves shown in Fig. 4b and Fig. 4e), the sensor exhibits a higher response slope and superior concentration resolution in the low-concentration regime. Integrating the theoretical model with the experimental data, we consider that the optimal operating range of this system is primarily from the sub-ppb level up to approximately 10-20 ppm. Within this range, the mode signal exhibits high response sensitivity to concentration changes and good quantitative resolution; whereas at higher concentrations, as the response curve gradually enters a weak-gain region, the amplitude of the mode change corresponding to a unit concentration change decreases significantly, and the sensitivity therefore no longer maintains the same advantage. It should be emphasized that, although the optimal performance of the system is concentrated in the low-concentration region, we still obtained good agreement with a commercial instrument under real ambient concentration conditions (approximately 400 ppm). This indicates that the proposed method can not only realize ultra-low concentration detection, but also possesses the capability for real environmental monitoring, with its core advantages being primarily manifested in trace-level detection scenarios.

Following the reviewer's suggestion, we have further supplemented the application directions suited to the operating characteristics of this sensor and illustrated them in conjunction with typical CO_2 concentration ranges:

4.1 Semiconductor manufacturing and quality control of electronic specialty gases

In high-purity process gases such as electronic-grade N_2, Ar, and He, CO_2 typically needs to be controlled within the range of 10 ppb to 1 ppm. Trace CO_2 contamination can affect thin-film deposition, etching processes, and device yield, thus necessitating high-sensitivity detection at low concentrations.

4.2 Quality monitoring of high-purity industrial gases and specialty gases

During the production, storage, transportation, and use of high-purity nitrogen, hydrogen, oxygen, and inert gases, CO_2 often serves as a key impurity indicator, with permissible concentrations typically at the ppb to ppm level.

4.3 Low-background carbon cycle experiments and trace release studies

In scenarios such as enclosed ecological experiments, soil respiration studies, and investigations of carbon exchange kinetics, CO_2 variations are typically only at the sub-ppm to several ppm level, requiring high-resolution detection of weak concentration changes.

4.4 Isotopic CO_2 detection and expansion of tracer analysis platforms

Based on narrow-linewidth absorption lines, this method can be extended to the selective detection of $^{13}CO_2$ and $^{14}CO_2$. In related studies, the target signals are usually at extremely low abundance or trace concentration levels.

4.5 Analysis of low-pressure rarefied gas environments

In low-pressure, low-density gas environments (e.g., simulated rarefied atmosphere experiments, residual gas analysis in vacuum systems), trace CO_2 typically exists at ppm or even lower concentrations, requiring high-sensitivity detection capability.

Based on the above analysis, we have added a relevant explanation at the end of the first paragraph of the Discussion section in the revised manuscript to more precisely define the optimal applicable concentration range and application boundaries of this sensor. The newly added description reads as follows:

"Furthermore, although the system demonstrates good agreement with a commercial instrument in ambient-air CO_2 monitoring, thereby validating its reliable sensing capability under atmospheric-pressure conditions, the proposed dissipative WGM sensor exhibits superior sensitivity in the low-concentration regime (from sub-ppb levels to approximately 10-20 ppm). Within this range, the microcavity resonance response maintains a high concentration-dependent slope, enabling enhanced concentration resolution and improved sensitivity to trace-level variations. Consequently, the sensor is particularly well suited for trace CO_2 detection applications, including semiconductor manufacturing and electronic specialty gas quality control, high-purity industrial gas monitoring, low-background carbon-cycle and trace carbon-release studies, isotope-resolved CO_2 sensing, and trace gas analysis in low-pressure or rarefied environments, where its high sensitivity and resolution offer significant practical value."

Comment 5: *Finally, regarding the state of the art for on-chip laser absorption spectroscopy sensors, the following paper reports a detection limit of 20 ppm for CO2 (I referred to this in my previous review) and should be mentioned for completeness: https://doi.org/10.1364/OPTICA.533710*

Answer 5: We sincerely thank the reviewer for this insightful and important suggestion, and for recommending this representative work on on-chip laser absorption

spectroscopy sensors. This study provides a valuable reference for refining the comparison of performance among different gas sensing technologies.

We have carefully studied the recommended paper. It reports an on-chip laser absorption spectroscopy platform based on a suspended nanophotonic waveguide, achieving a CO_2 detection limit of 20 ppb and, for the first time, demonstrating stable isotope ratio ($\delta^{13}C$) measurements within an integrated on-chip architecture. This work represents a significant advance in integrated laser absorption spectroscopy and highlights the rapid progress of on-chip gas sensing technologies in terms of both sensitivity and system-level integration.

Following the reviewer's valuable suggestion, we have added a citation to this work in the revised manuscript (now Reference [50]) and incorporated it into Table 1, where the performance of the reported system is compared with that of our sensor and other representative gas sensing platforms. This addition further refines the discussion of the current state of the art and provides a more complete and balanced literature review.

50. Salaj, J. *et al.* Suspended nanophotonic waveguide for isotope-specific CO_2 detection. *Optica* **11**, 1654-1662 (2024).

Table 1. Comparison of gas sensing performance of state-of-the-art CO_2 sensors

Spectral technology	LOD[a]	Sample cell volume	Difficulty level of optical path adjustment	TSO[b]	Optimal integration time	Wavelength
Multi-pass Cell[46]	130 ppb	Large	Normal	Medium	18 s	2.004 μm
OA-CEAS[47]	1.4 ppb	Large	Hard	High	100 s	1.6 μm
CRDS[48]	160 ppb	Large	Hard	High	816 s	1.6 μm
On-chip Slot Waveguide[49]	627 ppb	Small	Easy	Low	36 s	4.23 μm
On-chip Suspended Waveguide[50]	20 ppb	Small	Easy	Low	40 s	4.345 μm
SEIRA[51]	1 ppm	Medium	Hard	Medium	N/A	6-8 μm
PAS[52]	37 ppb	Medium	Normal	Medium	307 s	4.3 μm
QEPAS[53]	35 ppb	Small	Normal	Medium	670 s	2.0 μm
MEMS[54]	580 ppt	Small	Easy	Low	49 s	4.323 μm
CEPAS[55]	4 ppm	Small	Normal	High	75 s	1.57 μm
Photothermal Spectroscopy[56]	1.5 ppb	Medium	Easy	High	1000 s	2.003 μm
LITES[57]	3.59 ppm	Large	Easy	Medium	100 s	2.00401 μm
Dispersion Spectroscopy[58]	65.78 ppb	Medium	Normal	High	218 s	4.329 μm
NDIR[59]	2 ppm	Large	Normal	Medium	N/A	4.3 μm
FTIR[60]	10 ppm	Large	Hard	High	5 s	4.25 μm

Fiber FP Interferometer[61]	480 ppb	Small	Easy	Medium	N/A	4.266 μm
Fiber Grating[62]	401 ppm	Medium	Easy	Medium	20 s	0.7 μm
Silicon Microring Resonator[63]	370 ppm	Small	Easy	High	N/A	1.56 μm
Antiresonant Fiber[64]	144 ppm	Small	Normal	Medium	1.5 s	1.574 μm
Photonic Crystals[65]	2000 ppm	Small	Easy	Medium	N/A	0.463-0.668 μm
Functionalized Dispersed WGM[66]	50 ppm	Small	Easy	Low	N/A	1.525-1.57 μm
Dispersed WGM[67]	184 ppb	Small	Easy	Low	N/A	1.55 μm
Dissipative WGM (This work)	***168 ppt***	***Small***	***Easy***	***Low***	***400 s***	***1.572 μm***

[a]LOD represents the limit of detection.

[b]TSO represents total system cost.

Reply to Reviewer #2

The authors have largely addressed my comments, and I support publication of the manuscript in its revised form. I appreciate the authors' substantial revision and the extensive additional work performed in response to the reviewer comments. The manuscript has improved significantly compared with the original submission. In particular, the added cross-sensitivity experiments, additional gas measurements, expanded supplementary analysis, and more balanced comparison with prior WGM sensing literature strengthen the technical support for the reported sensing platform.

Judith Su

We sincerely thank the reviewer for the positive evaluation and for recognizing the substantial improvements made in the revised manuscript. We greatly appreciate your constructive comments, which have helped us strengthen the technical rigor and overall quality of our work. We are particularly encouraged that the added cross-sensitivity experiments, additional gas measurements, expanded supplementary analysis, and the more balanced comparison with prior WGM sensing literature have been found to enhance the support for the proposed sensing platform. Your feedback has been invaluable in guiding these revisions.

<u>Reply to Reviewer #3</u>

The revision has improved the quality of the manuscript greatly. I think the present paper is suitable for publication.

We sincerely thank the reviewer for the positive assessment and encouraging feedback. We are pleased that the revisions have significantly improved the quality of the manuscript and that you consider it suitable for publication. Your evaluation is greatly appreciated.
